# Discovery of new Hsp90–Cdc37 protein–protein interaction inhibitors: *in silico* screening and optimization of anticancer activity†

**DOI:** 10.1039/d4ra05878j

**Published:** 2024-09-05

**Authors:** Jaka Dernovšek, Nina Gradišek, Živa Zajec, Dunja Urbančič, Jernej Cingl, Tjaša Goričan, Simona Golič Grdadolnik, Tihomir Tomašič

**Affiliations:** a Faculty of Pharmacy, University of Ljubljana Aškerčeva cesta 7 1000 Ljubljana Slovenia tihomir.tomasic@ffa.uni-lj.si; b Laboratory for Molecular Structural Dynamics, Theory Department, National Institute of Chemistry Hajdrihova 19 1001 Ljubljana Slovenia

## Abstract

The interaction between heat shock protein 90 (Hsp90) and Hsp90 co-chaperone cell-division cycle 37 (Cdc37) is crucial for the folding and maturation of several oncogenic proteins, particularly protein kinases. This makes the inhibition of this protein–protein interaction (PPI) an interesting target for developing new anticancer compounds. However, due to the large interaction surface, developing PPI inhibitors is challenging. In this work, we describe the discovery of new Hsp90–Cdc37 PPI inhibitors using a ligand-based virtual screening approach. Initial hit compounds showed Hsp90 binding, resulting in anticancer activity in the MCF-7 breast cancer cell line. To optimize their antiproliferative effect, 35 analogs were prepared. Binding affinity for Hsp90 was determined for the most promising compounds, 8c (*K*_d_ = 70.8 μM) and 13g (*K*_d_ = 73.3 μM), both of which interfered with the binding of Cdc37 to Hsp90. This resulted in anticancer activity against Ewing sarcoma (SK-N-MC), breast cancer (MCF-7), and leukemia (THP-1) cell lines *in vitro*. Furthermore, compounds 8c and 13g demonstrated the ability to induce apoptosis in the Ewing sarcoma cell line and caused a decrease in the levels of several known Hsp90 client proteins in MCF-7 cells, all without inducing the heat shock response.

## Abstract 2

The interaction between heat shock protein 90 (Hsp90) and Hsp90 co-chaperone cell-division cycle 37 (Cdc37) is crucial for the folding and maturation of several oncogenic proteins, particularly protein kinases.

## Graphical abstract

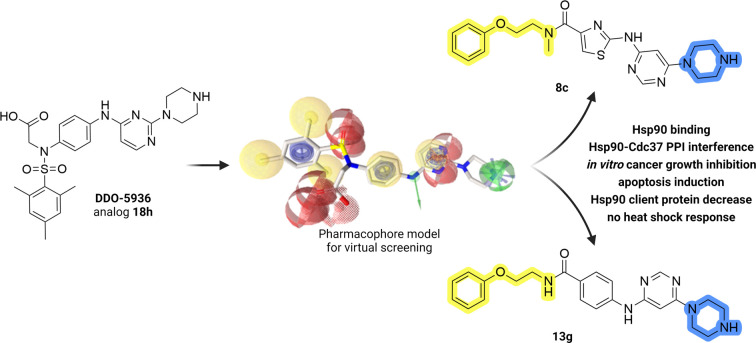

## Introduction

Proteins are among the key cellular building blocks, as their roles include structural support, immune function and signal transduction.^1^ Many proteins rely on chaperones to obtain their functional structure, especially when destabilizing mutations occur, as is often the case with various oncoproteins.^2–4^ Therefore, correct folding and stabilization of oncoproteins is necessary in cancer pathology and the role of chaperones in the development of malignancies is clear.^5,6^ Perhaps one of the most studied cancer-related chaperones is heat shock protein 90 (Hsp90),^7,8^ which is essential for the correct folding of more than 400 client proteins. Several of these are oncogenic proteins such as Cdk4, Akt, Her2 and ERα, to name a few.^8–10^

The inhibition of the chaperone cycle of Hsp90 can lead to the downregulation of oncogenic drivers, emphasizing the potential of Hsp90 inhibitors as anticancer agents. The overexpression of Hsp90 and its higher affinity for ATP in cancer cells provide a basis for selective targeting of malignantly transformed cells.^10,11^ Unfortunately, the first discovered Hsp90 inhibitor, geldanamycin,^12^ was too unstable and potentially toxic for clinical use.^13,14^ However, optimized analogs like 17-AAG and 17-DMAG have entered clinical trials. Subsequently, other structural classes with similar mechanisms of action were proposed as clinical candidates. These inhibitors primarily targeted the ATP-binding pocket of the N-terminal domain (NTD) of Hsp90.^10,13–15^ Despite potent on-target activity, these inhibitors were not as successful in the clinic. It was not until 2022 that the first N-terminal Hsp90 inhibitor, pimitespib, was approved for the treatment of gastrointestinal stromal tumors in Japan.^16,17^

Due to limited clinical success of the first Hsp90 N-terminal inhibitors, other approaches targeting various structural and functional traits of Hsp90 started to emerge.^13^ The Hsp90 chaperone family is highly conserved: a charged linker connects the ATP-binding NTD to the client-binding middle domain (MD), which then extends into the C-terminal domain (CTD), where dimerization occurs.^18^ Moreover, all three domains of Hsp90 participate in protein–protein interactions (PPI) with different co-chaperones essential for normal progression through chaperone cycle stages.^19,20^ For instance, activator of ATPase homologue 1 (Aha1), which binds to the MD and NTD, enhances the otherwise low ATPase activity of Hsp90.^21,22^ Conversely, the NTD-binding p23 inhibits ATPase activity and prolongs Hsp90's interaction with client proteins.^23,24^ p23 acts at a later stage of the chaperone cycle,^25^ while the Hsp70/Hsp90 organizing protein (Hop) binds the CTD of Hsp90 at the beginning, recruiting client proteins to Hsp90. Clients like glucocorticoid receptors are transferred from a complex with Hsp70 to Hsp90 by Hop.^26,27^ On the other hand, protein kinases and some other client proteins rely on cell division cycle 37 (Cdc37/p50) for their recruitment to Hsp90. Like Hsp90, the expression levels of Cdc37 are increased in cancer cells,^28^ making Hsp90–Cdc37 PPI particularly intriguing for the design of potential cancer growth inhibitors. Compounds blocking this PPI enable a more targeted approach towards the inhibition of folding and maturation of oncogenic Hsp90 clients, such as Cdk4, Akt, c-Raf, and the androgen receptor, while leaving others less affected.^28–32^ Disruption of Hsp90–Cdc37 PPI could result in a more selective approach to disrupting the Hsp90 chaperone cycle.

**Fig. 1:**
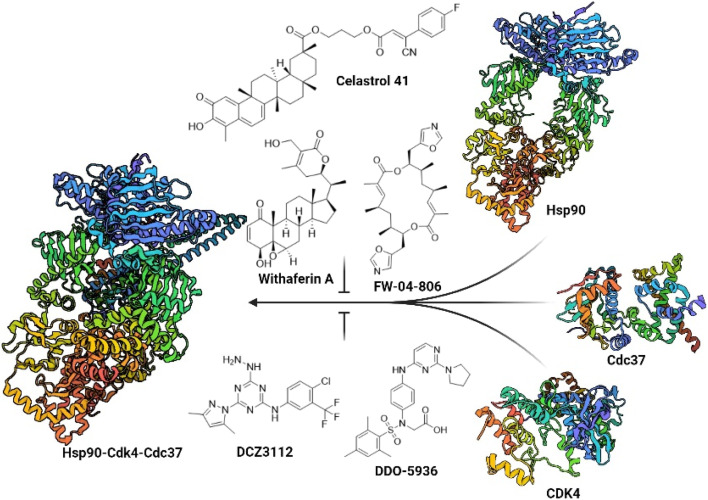
Representative natural products and celastrol derivative 41, withaferin A, FW-04-806 and rationally designed small molecules DCZ3112 and DDO-5936, which prevent the formation of the Hsp90–Cdc37 PPI. Blocking the interaction between Hsp90 (PDB ID: 8EOB) and Cdc37 (PDB ID: 2K5B) prevents the loading of Cdc37 clients such as Cdk4 (PDB ID: 2W96) to Hsp90 and thus the formation of the Hsp90-Cdk4-Cdc37 (PDB ID: 5FWP) complex.

Despite the challenging druggability of PPIs, several classes of Hsp90–Cdc37 PPI inhibitors have been described to date (Fig. 1).^33,34^ Most were derivatives of natural products or larger peptides with relatively poorly characterized binding sites on either Hsp90 or Cdc37.^35–43^ This changed in 2018 and 2019 with the discovery of DCZ311244 ^44^ and DDO-5936.^45^ Wang and colleagues found that the binding of DDO-5936 is largely dependent on Asp47 on the NTD of Hsp90. They also identified Arg167 and Gln133 as indispensable for the binding of Cdc37 to Hsp90.^45^ Subsequently, they prepared a library of analogs to establish the structure–activity relationships (SAR) of this compound class.^46,47^ The binding site of DDO-5936 on Hsp90 NTD was established through mutagenesis, protein NMR studies, and binding affinity measurements with both wild-type and mutated Hsp90. These assays identified Asp47 as a key binding determinant, as DDO-5936 showed no affinity for the Asp47Ala mutant of Hsp90 (*K*_d_ > 100 μM for the mutant *vs. K*_d_ = 3.86 μM for the wild-type). This suggests that DDO-5936 binds to a previously unknown NTD binding site adjacent to the ATP-binding site.^45^ However, recent cryo-EM structures of the full-length Hsp90β dimer in complex with Cdc37 and various client proteins^29,31,48^ indicate that interaction with Asp42 (Hsp90β amino acid numbering) might not be possible in a cellular context, as the Leu388–Met394 loop folds into the proposed binding site, possibly rendering it inaccessible.

Based on these discoveries, we decided to investigate whether our enhanced understanding of the Hsp90 N-terminal binding site^45^ and its inhibitors^46,47^ could be combined with computer-aided drug design to develop new small-molecule inhibitors of Hsp90–Cdc37 PPI. In this study, we employed ligand-based molecular modeling approach to identify hit compounds capable of inhibiting Hsp90–Cdc37 PPI with *in vitro* anticancer potency. Additionally, we introduced several structural modifications to optimize the anticancer potential and overall properties of these hits and experimentally confirmed their mode of action as Hsp90–Cdc37 PPI inhibitors.

## Results

### Virtual screening

**Fig. 2:**
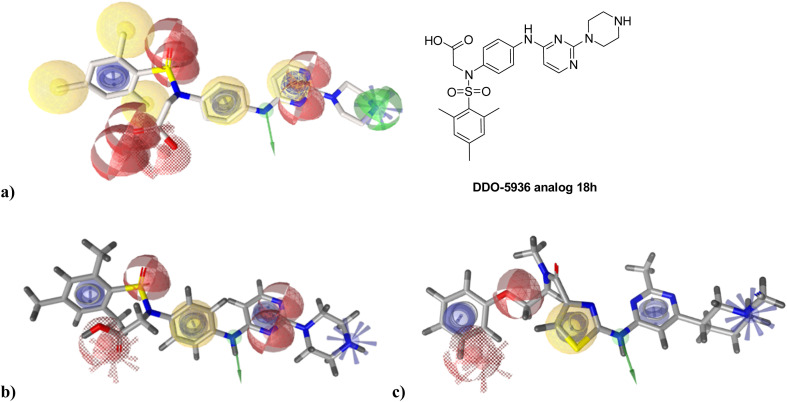
Overlay of the optimized DDO-5936 analog 18 h with (a) initial and (b) simplified ligand-based pharmacophore model. (c) Overlay of the virtual screening hit 1 with the simplified ligand-based pharmacophore model. Pharmacophore features are presented as follows: hydrogen bond acceptor – red sphere, hydrogen bond donor – green arrow or sphere, hydrophobic feature – yellow sphere, aromatic ring – blue disc, positively ionizable – blue star.

To identify new potential inhibitors of the Hsp90–Cdc37 PPI, we conducted a ligand-based virtual screening using a library of DDO-5936 analogs^45–47^ as the starting point. Four of the most potent Hsp90–Cdc37 PPI inhibitors (Table S1†), with *K*_d_ values ranging from 0.50 μM to 5.50 μM,^47^ were used as a training set to build a ligand-based pharmacophore model (LBPM) in LigandScout Expert.^49^ The initial LBPM model contained five hydrophobic features (yellow spheres), seven hydrogen bond acceptors (red spheres), two hydrogen bond donors (green arrow and sphere), three aromatic ring features (blue disc) and one positive ionizable feature (blue star) (Fig. 2a).

In the next step, the model was validated by screening a library of active (14 compounds, *K*_d_ < 20 μM) and inactive compounds (15 compounds, inhibition rate less than 50% at 100 μM).^45–47^ The model was highly selective and retrieved only six active compounds. Since the most potent compounds contained the basic piperazine moiety, neutral analogs like DDO-5936 were not identified as hits in the virtual screening. However, when we used this model in the virtual screening of the library of commercially available compounds, no hits were identified. To improve the hit rate, the initial LBPM was simplified by omitting selected hydrophobic features, hydrogen bond donors and acceptors (Fig. 2b). This decreased its specificity, as it retrieved three inactive compounds in addition to the six actives. When the simplified LBPM was used in the virtual screening, three structurally similar hits 1–3 were identified (Table 1 and Fig. 2c).

**Table 1:** Antiproliferative IC_50_ values (mean ± SD) of the virtual screening hits 1–3 determined in MCF-7 breast cancer cell line by MTS assay

Compound	Structure	MCF-7 IC_50_ (μM)
1	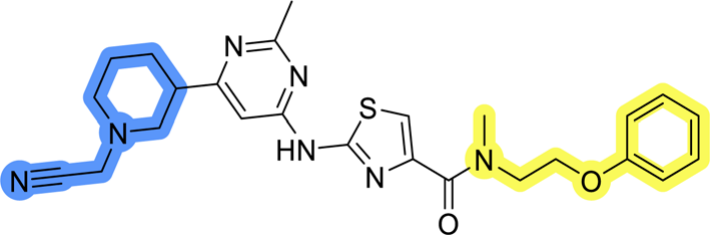	29.5 ± 5.4
2	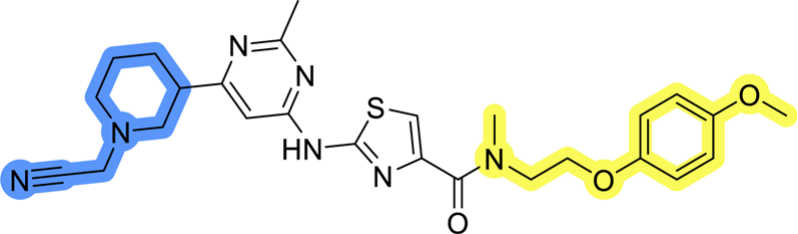	47.4 ± 0.9
3	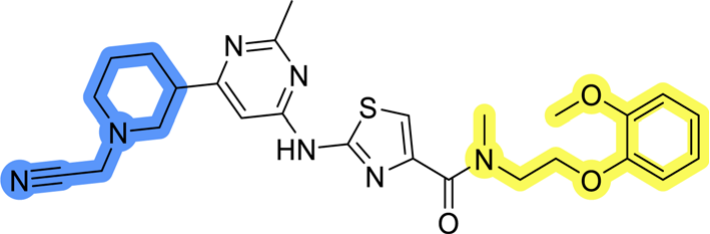	34.9 ± 2.7
DDO-5936		>50

Three compounds presented in Table 1 (1–3) were evaluated for their anticancer activity in MCF-7 breast cancer cells, and all three inhibited cell growth within the middle micromolar range (IC_50_ = 30–50 μM). Known Hsp90–Cdc37 PPI inhibitor DDO-5936 was used as a control but it did not inhibit the growth of MCF-7 cells with IC_50_ value under 50 μM (17% growth inhibition at 50 μM). Among the virtual screening hits, compound 1 demonstrated slightly higher anticancer activity than the other two. Therefore, its affinity for Hsp90 was evaluated using microscale thermophoresis (MST). Due to poor solubility of 1, its binding could only be confirmed qualitatively, as an increase in binding could be detected in concentrations above 39 μM (Fig. S1†), but the highest concentration without compound-induced protein aggregation (125 μM) was insufficient to reliably determine its *K*_d_ value. This confirmation of binding to Hsp90β, along with the antiproliferative activity of compound 1 in MCF-7 breast cancer cells, provided us with a starting point for further hit optimization.

### Design, synthesis and evaluation of library A

**Fig. 3:**
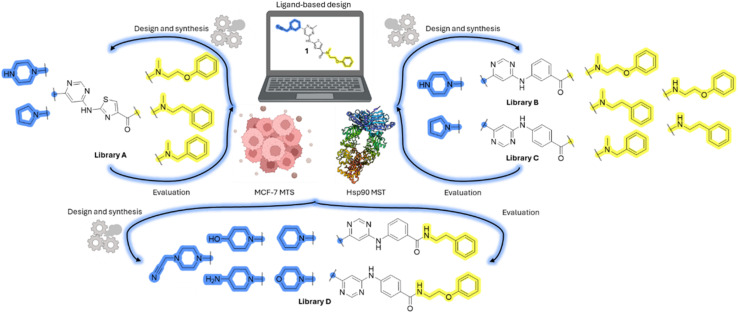
Schematic representation displaying our work plan following the discovery of hit compound 1. First, library A was designed, and the results of the biological evaluation steered the planning of libraries B and C, while library D was designed at the very end. The optimisation was aimed at the improvement of *in vitro* anticancer effects of the compounds.

In the design of library A, modifications of the piperidine moiety (colored in blue) and the linker bound phenyl ring (colored in yellow) were planned (Fig. 3). Since the cyanomethyl substituent at the piperidine ring of 1–3 seemed not to be important according to the ligand-based pharmacophore model, it was removed. Similarly, the C–C bond in compound 1 connecting piperidine and pyrimidine rings was replaced with a synthetically more accessible C–N bond. Additionally, a neutral substituent was considered in place of the piperidine, as a basic center seemed important but not crucial for activity of DDO-5936 and its analogs.^45–47^ On the other side of the molecule, the unsubstituted phenyl ring of compound 1 was retained, while linker variations were planned to reduce flexibility and investigate potential effects on antiproliferative activity. Apart from removing the methyl group on the pyrimidine of compound 1, the core 2-((pyrimidin-4-yl)amino)thiazole was kept unchanged.

**Scheme 1:**
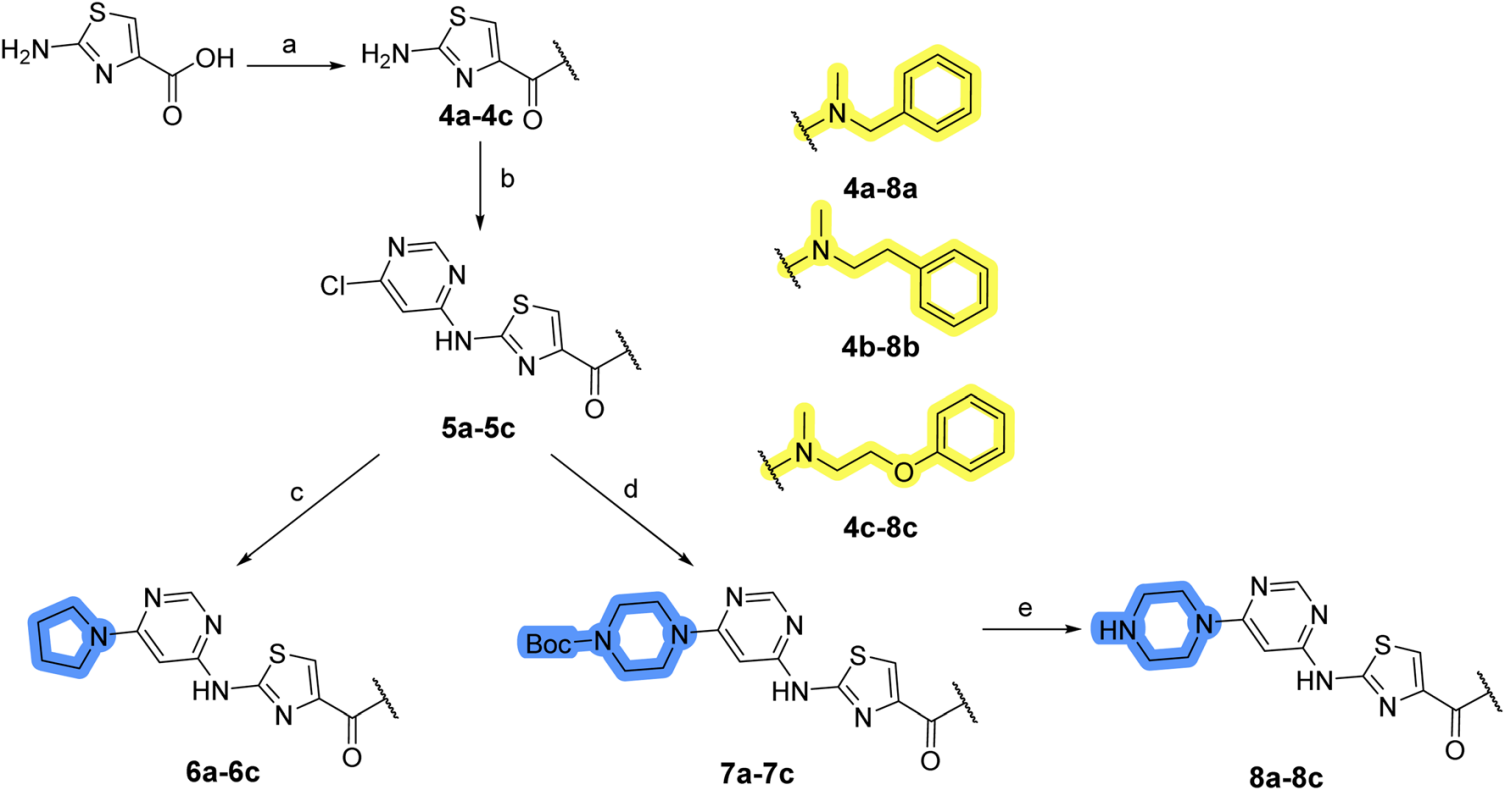
Reagents and conditions for the preparation of library A: (a) (i) EDC, HOBt, NMM, ice bath, 20 minutes; (ii) respective amine, ambient temperature, overnight; (b) 4,6-dichloropyrimidine, NaH, DMF, 0 °C to ambient temperature, overnight; (c) pyrrolidine, DMF, 80 °C, overnight; (d) *N*-Boc-piperazine, DIPEA, DMF, 80 °C, overnight; (e) TFA, DCM, ambient temperature, 20 h.

The synthesis of library A (Scheme 1) began with an amide coupling between respective amines and 2-aminothiazole-4-carboxylic acid using 1-ethyl-3-(3-dimethylaminopropyl)carbodiimide (EDC) and 1-hydroxybenzotriazole (HOBt) to produce compounds 4a–c. The resulting 2-aminothiazolamides were reacted with 4,6-dichloropyrimidine in a nucleophilic aromatic substitution to yield compounds 5a–c. To enable the reaction of nucleophilic aromatic substitution, the nucleophilicity of the slightly acidic amino group of 2-aminothiazoles 5a–c was increased by *in situ* deprotonation using sodium hydride. Nucleophilic aromatic substitution was then performed again using pyrrolidine to give final products 6a–c and *N*-Boc-piperazine to produce intermediates 7a–c. In the final step, the Boc protecting groups of 7a–c were removed by acidolysis to synthesize the final compounds 8a–c.

Compounds 6a–c and 8a–c were evaluated for their inhibitory effect on the MCF-7 breast cancer cell line (Table 2). The first set of changes resulted in a slight activity increase from 29.5 ± 5.4 μM for compound 1 to 15.2 ± 0.7 μM and 17.1 ± 1.4 μM for compounds 6b and 8a, respectively. The results also indicate that a basic center is not necessary for the antiproliferative effect in MCF-7 cell line, as both piperazine and pyrrolidine substituents show similar activities. This is in line with previous findings regarding the SAR of compounds used to set up the pharmacophore model.^46,47^ The removal of the cyanomethyl substituent, the replacement of the C–C bond with its C–N counterpart, and the changes to the linker between the core thiazole and benzene ring were well tolerated. However, no significant and consistent improvement in MCF-7 cell growth inhibition was observed for library A, regardless of the introduced changes. Therefore, we decided to replace the aminothiazole core with *meta*- and *para*-substituted benzene rings in libraries B and C, respectively.

**Table 2:** Antiproliferative IC_50_ values (mean ± SD) of the compounds 6a–c and 8a–c of library A determined in MCF-7 breast cancer cell line

Compound	Structure	MCF-7 IC_50_ (μM)
6a	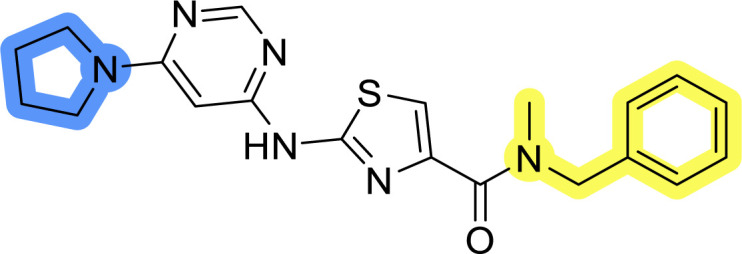	20.8 ± 2.2
6b	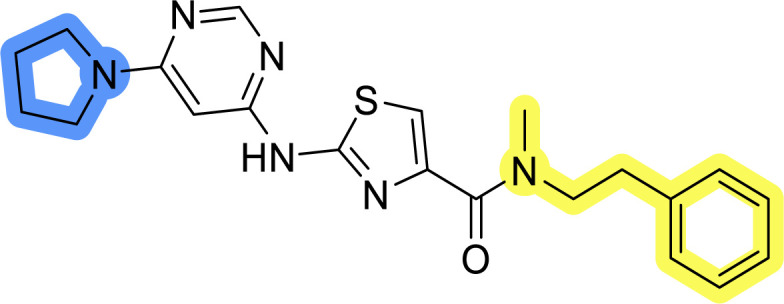	15.2 ± 0.7
6c	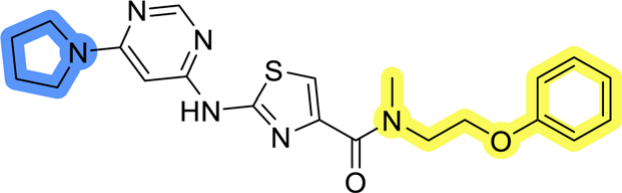	28.1 ± 1.4
8a	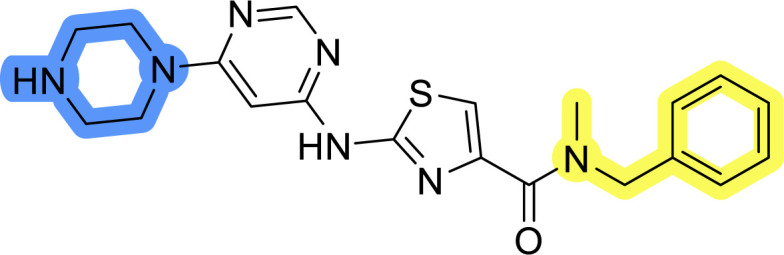	17.1 ± 1.4
8b	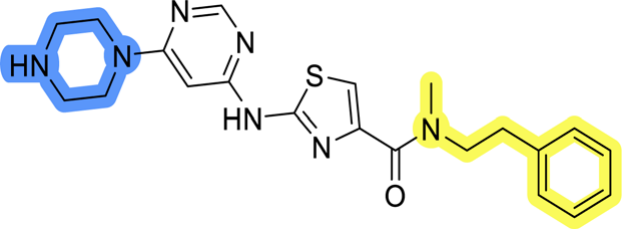	27.3 ± 2.0
8c	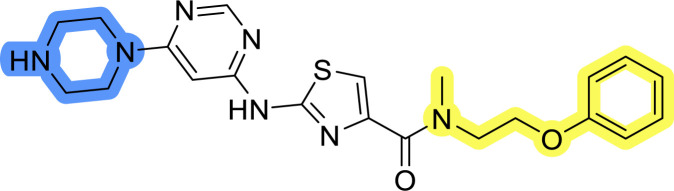	20.0 ± 1.2

### Design, synthesis and evaluation of libraries B and C

The use of 3- and 4-aminophenyl backbones in libraries B and C enabled further exploration of the chemical space around the central core (Fig. 2). 3- and 4-aminophenyl rings comparably afford a slightly different but still similar orientation at the core to that of 2-aminothiazole ring in library A. Additionally, the benzene core provided an alternative synthetic route without the need for a strong base for the activation of 2-aminothiazole, due to the greater inert nucleophilicity of the aniline. Therefore, non-methylated phenethylamine and 2-amino-1-phenoxyethane were added to the linker-to-benzene library used in amide bond formation to complement the initial three fragments used in library A. All five amines were attached to both *meta*- (library B) and *para*-aminobenzoic acid (library C) to enable a better comparison of the results.

**Scheme 2:**
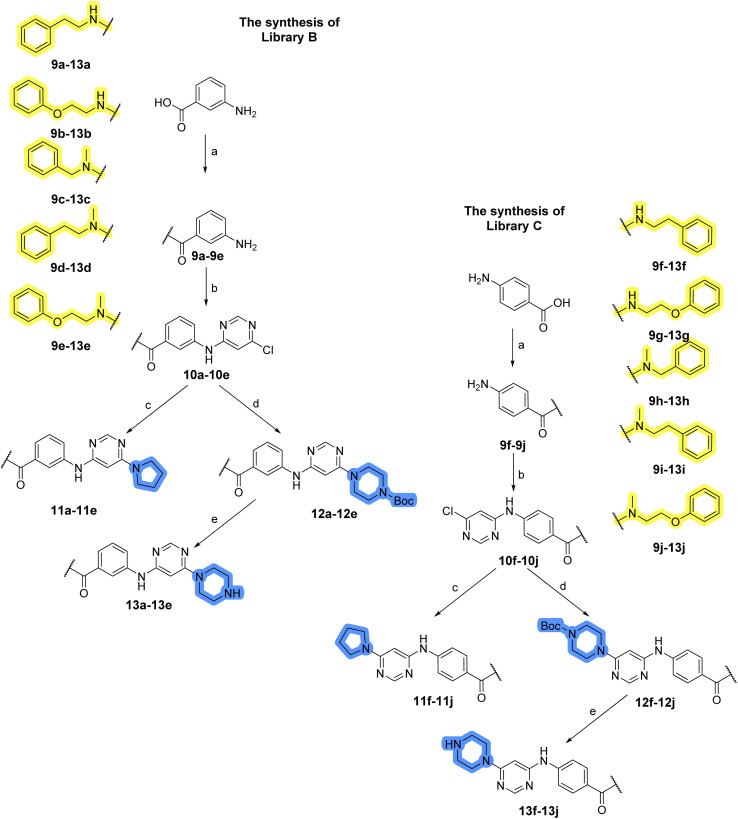
Reagents and conditions for the preparation of libraries B and C: (a) (i) EDC, HOBt, NMM, ice bath, 20 minutes; (ii) respective amine, ambient temperature, overnight; (b) 4,6-dichloropyrimidine, DIPEA, DMF, 100 °C, 2 days; (c) pyrrolidine, DMF, 80 °C, overnight; (d) *N*-Boc-piperazine, DIPEA, DMF, 80 °C; (e) TFA, DCM, ambient temperature, 20 h.

In the synthesis of libraries B and C (Scheme 2), the respective amides 9a–j were first prepared by EDC- and HOBt-promoted amide coupling. In the two consecutive nucleophilic aromatic substitutions, 9a–j were first reacted with 2,4-dichloropyrimidine to give intermediates 10a–j. Then, either pyrrolidine or *N*-Boc-piperazine were attached to yield final products 11a–j or intermediates 12a–j, respectively. This was followed by Boc-deprotection using TFA-catalyzed acidolysis to complete the synthesis of libraries B and C with final compounds 13a–j.

Upon preparation, compounds 11a–j and 13a–j were tested for their antiproliferative activities on MCF-7 cells. Unfortunately, the compounds in libraries B (Table 3) and C (Table 4) did not show significant improvement in cancer cell growth inhibition. The most potent compounds, 13h and 11b, closely followed by 13f, 11i, 11h, and 13g, exhibited similar potency to that of 6b and 8a from library A, with IC_50_ values approaching the low micromolar range. Overall, the mean IC_50_ values for active compounds in libraries B and C are 27.6 μM and 21.0 μM, respectively, indicating a trend that favors *para* substitution, though not statistically significant (*p* value = 0.087, Kolmogorov–Smirnov *t*-test). However, the *para*-substituted set includes three inactive compounds compared to only two in its *meta*-substituted counterpart. The piperazine substituent, more related to hit compounds, is slightly better tolerated with *para* substitution. Additionally, the introduction of non-methylated amides confirmed that methyl is not crucial for the antiproliferative effect of the compounds. Considering all five linkers introduced in libraries A, B, and C, the changes in activity appear to be sporadic, and no definitive conclusion regarding individual fragment combinations can be drawn. Based on accessibility, *N*-methyl-2-phenylethan-1-amine and 2-phenoxyethan-1-amine were coupled to *meta*- and *para*-aminobenzoic acid, respectively, for further analog preparation (Fig. 2).

**Table 3:** Antiproliferative IC_50_ values (mean ± SD) of the compounds 11a–e and 13a–e of library B determined in MCF-7 breast cancer cell line

Compound	Structure	MCF-7 IC_50_ (μM)
11a	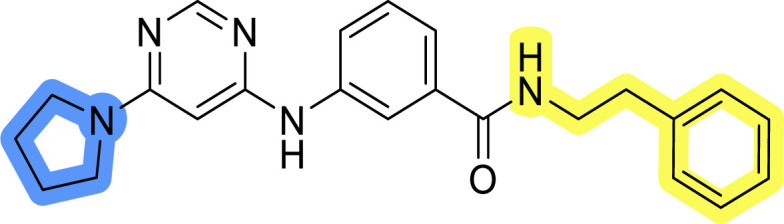	20.6 ± 3.0
11b	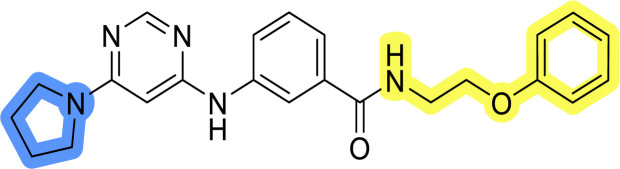	14.6 ± 4.5
11c	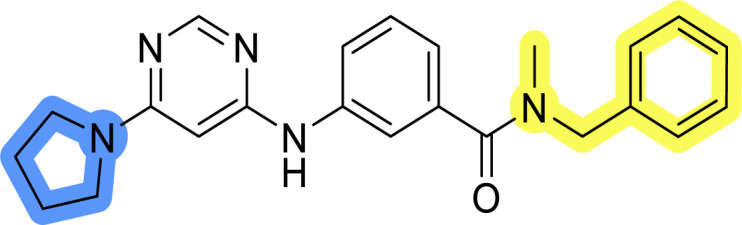	36.7 ± 6.1
11d	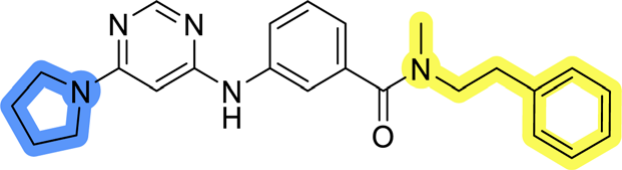	29.1 ± 5.0
11e	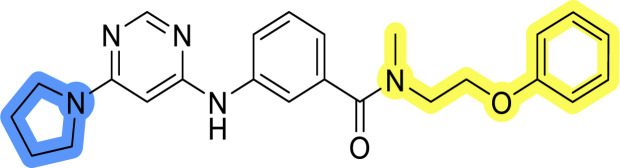	34.1 ± 2.9
13a	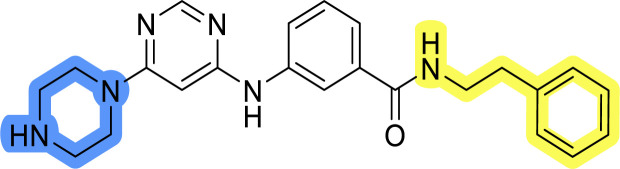	31.9 ± 3.2
13b	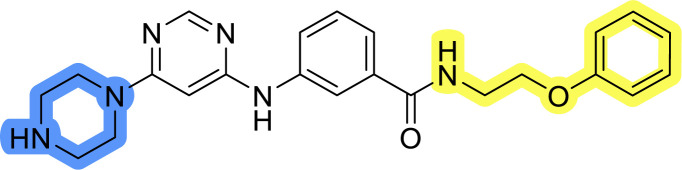	22.5 ± 3.3
13c	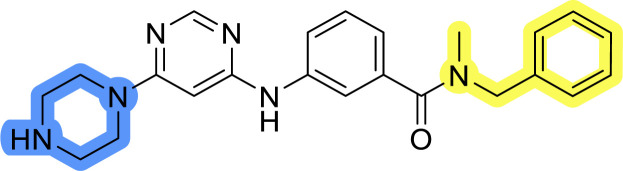	31.9 ± 2.1
13d	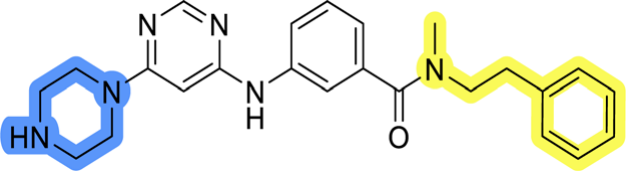	>50
13e	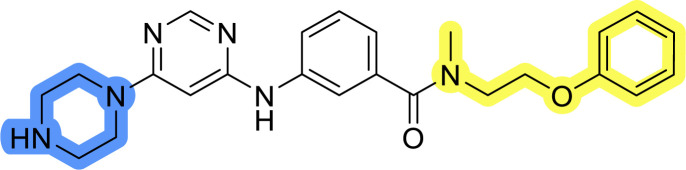	>50

**Table 4:** Antiproliferative IC_50_ values (mean ± SD) of the compounds 11f–j and 13f–j of library C determined in MCF-7 breast cancer cell line

Compound	Structure	MCF-7 IC_50_ (μM)
11f	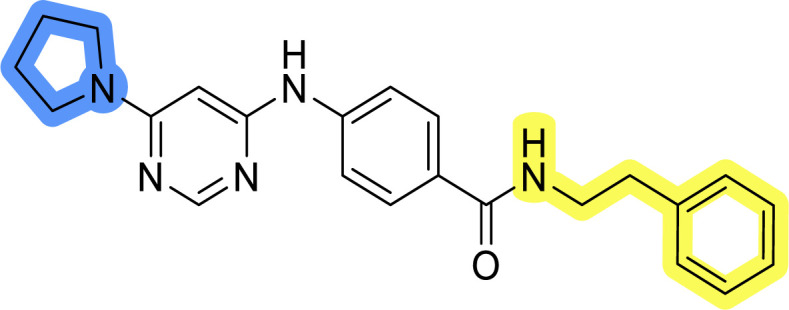	>50
11g	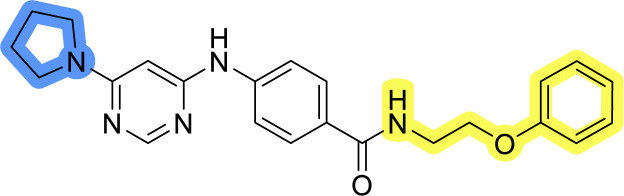	>50
11h	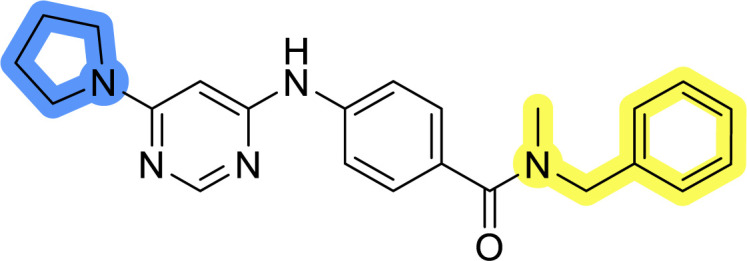	18.3 ± 7.0
11i	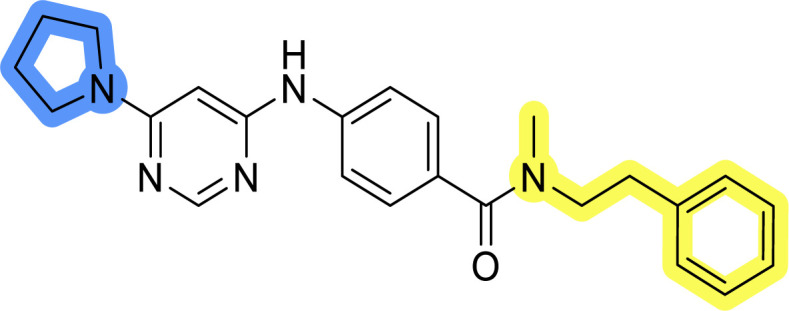	18.0 ± 1.5
11j	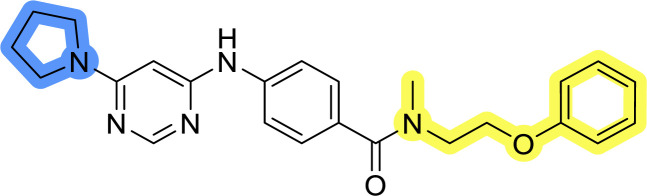	>50
13f	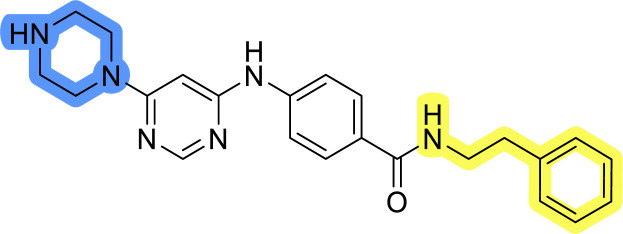	17.9 ± 0.5
13g	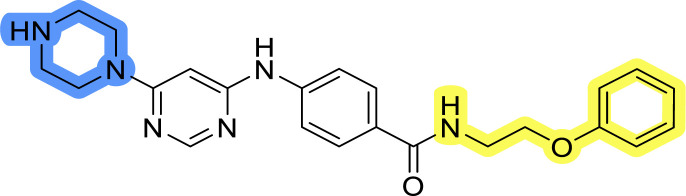	19.3 ± 2.0
13h	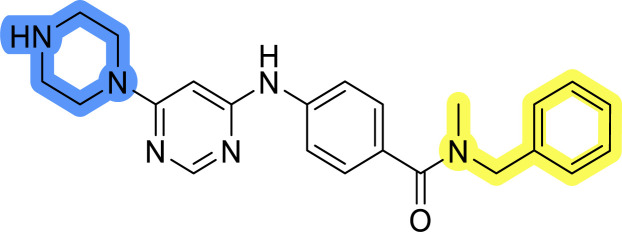	14.5 ± 4.7
13i	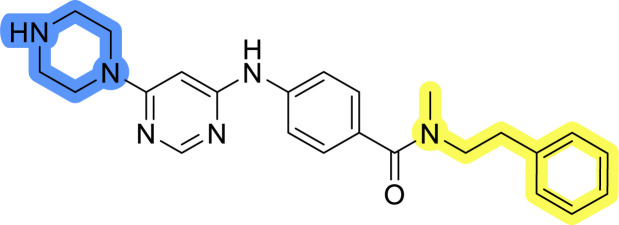	38.3 ± 4.9
13j	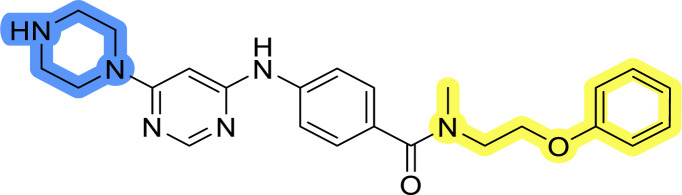	20.8 ± 2.7

### Design, synthesis and evaluation of library D

**Scheme 3:**
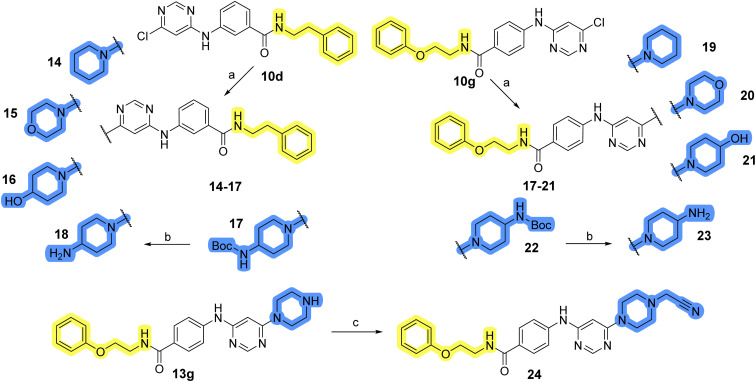
Reagents and conditions for the preparation of library D: (a) respective aliphatic amine, DMF, 90 °C, overnight; (b) TFA, DCM, ambient temperature, 20 h; (c) bromoacetonitrile, CH_3_CN : DCM = 5 : 1, ambient temperature, overnight.

The last set of changes focused on the pyrrolidine/piperidine part of the molecules, as a final attempt to optimize their antiproliferative effects. The comparison between compounds containing the piperazine and those with pyrrolidine in libraries A–C revealed that a functional group enabling ionic interaction or hydrogen bonding is not essential, with any of the central cores. To further investigate the effect of substituents at this position, piperidine, morpholine, 4-hydroxypiperidine, and 4-aminopiperidine were attached to 10d and 10g to prepare analogs 14–16, 18–21 and 23–24. In all cases, nucleophilic aromatic substitution was performed for the synthesis of compounds 14–17 and 19–22 (Scheme 3). Boc protecting group was cleaved by acidolysis to give 18 and 23. Additionally, electrophilic bromoacetonitrile was reacted with amine 13g in a nucleophilic substitution to produce compound 24, a close analog of 1, bearing the cyano group.

Compounds of library D were then evaluated for their inhibitory potency in the MCF-7 cell line (Table 5). Surprisingly, only 15 and 16 inhibited cancer cell growth with IC_50_ values lower than 50 μM, while the other compounds were inactive. The effects were only visible with the *meta*-substituted core phenyl ring, suggesting that this core allows a more favorable positioning of the potential hydrogen bond donors and acceptors, as seen in compounds with morpholine (15) and 4-hydroxypiperidine (16). Interestingly, compound 18 carrying a 4-aminopiperidine at the same position and the other analogs were not active. Overall, the inactivity of most library D analogs showed that the initial pyrrolidine and piperazine substituents were more suitable for this position. Also, the re-introduction of the cyanomethyl substituent to 13g (19.3 ± 2.0 μM) in compound 24 resulted in a loss of activity.

**Table 5:** Antiproliferative IC_50_ values (mean ± SD) of the compounds 14–16, 18–21 and 23–24 of library D determined in MCF-7 breast cancer cell line

Compound	Structure	MCF-7 IC_50_ (μM)
14	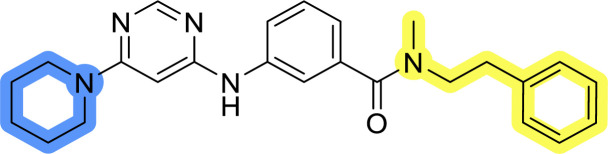	>50
15	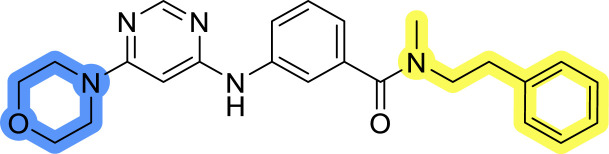	18.3 ± 7.0
16	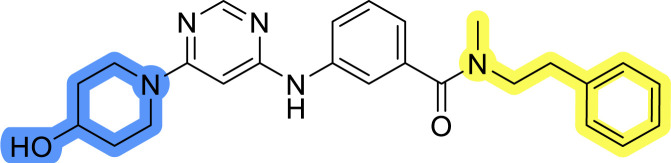	18.0 ± 1.5
18	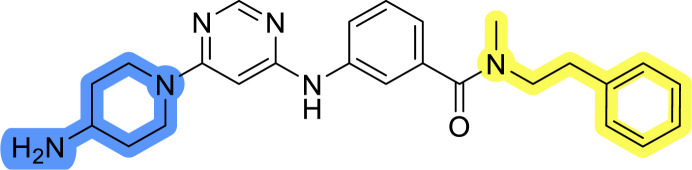	>50
19	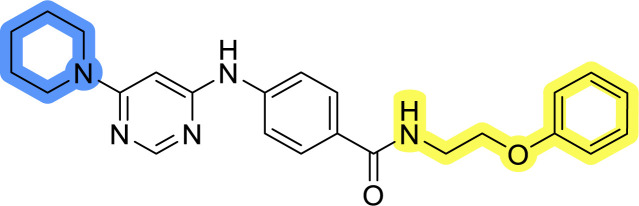	>50
20	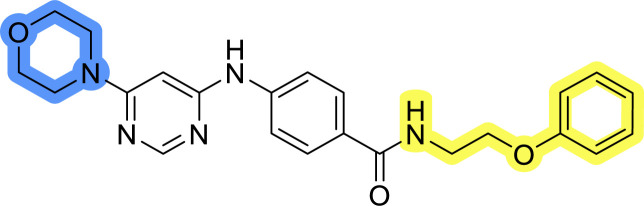	>50
21	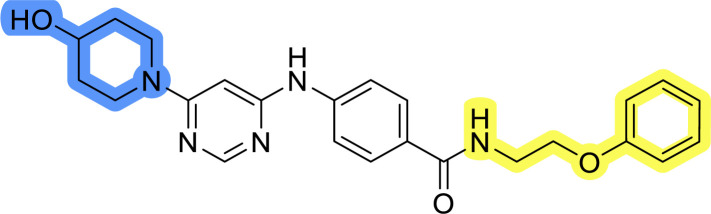	>50
23	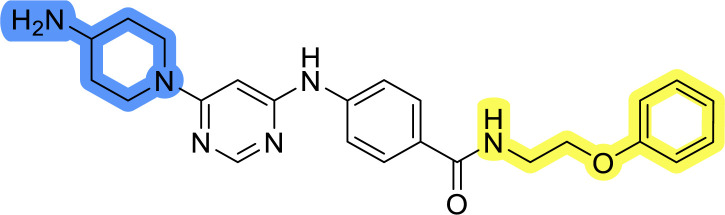	>50
24	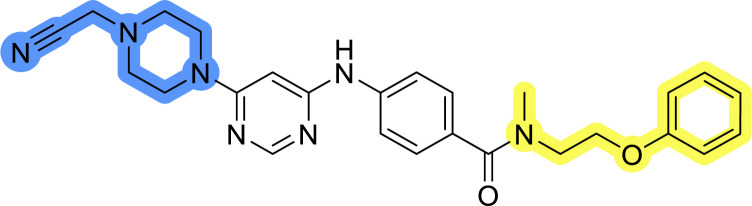	>50

Initial hit compound 1 and its representative analogs 6c, 8c, 11b, 11g, 13b, and 13g were selected for further evaluation of antiproliferative activity in the Ewing sarcoma (EwS) cell line SK-N-MC and the monocytic leukemia cell line THP-1 (Table 6). The results in EwS cell line were consistent with the MCF-7 data, as 11g was the only inactive compound, and the IC_50_ values for 1, 11b, 13b, and 13g were similar in both cell lines. In the remaining 6c and 8c pair, 6c was found to be less potent in EwS, while 8c was a stronger growth inhibitor of SK-N-MC cells than MCF-7 cells. Interestingly, only 8c and 13g retained some inhibitory activity in the leukemia cell line THP-1, while the IC_50_ values for the remaining compounds were greater than 50 μM. Surprisingly, the purchased DDO-5936 did not inhibit the growth of any of the tested cell lines with IC_50_ values lower than 50 μM. Due to the potency of 8c, the ability of both 8c and 13g to induce apoptosis in the EwS cells SK-N-MC was further evaluated by annexin V and Sytox Blue staining.

**Table 6:** Antiproliferative IC_50_ values (mean ± SD) of the compounds 1, 6c, 8c, 11b, 11g, 13b, 13g and DDO-5936 determined in Ewing sarcoma cell line SK-N-MC and leukemia cell line THP-1

Compound	Structure	SK-N-MC IC_50_ (μM)	THP-1 IC_50_ (μM)
1	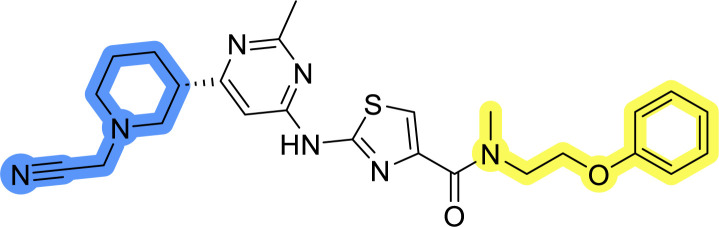	25.0 ± 0.4	>50
6c	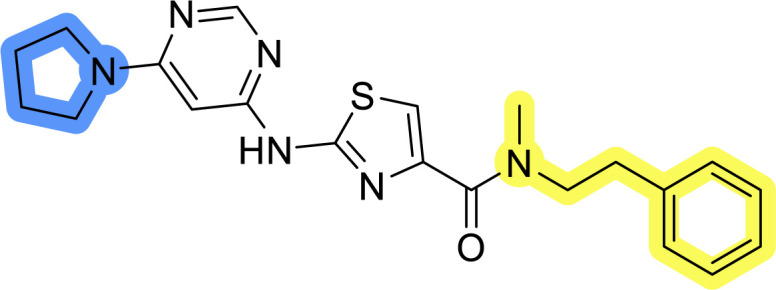	26.2 ± 0.4	>50
8c	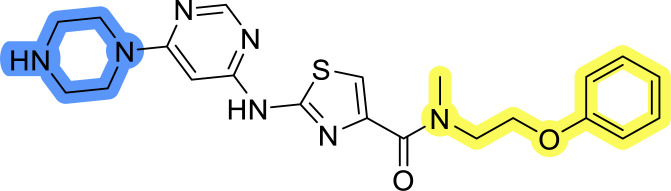	12.8 ± 0.9	33.9 ± 8.5
11b	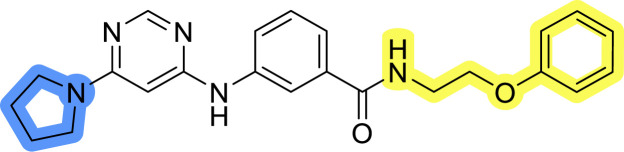	26.9 ± 1.0	>50
11g	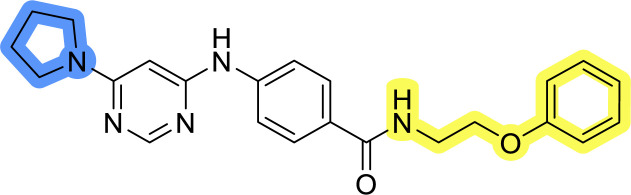	>50	>50
13b	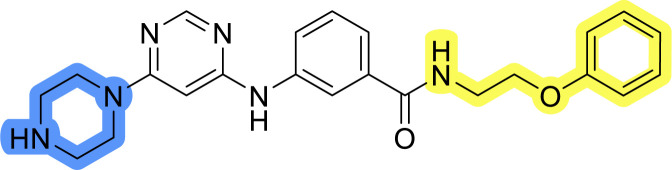	22.2 ± 0.2	>50
13g	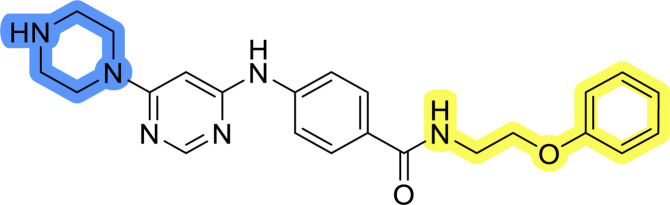	20.0 ± 1.5	41.5 ± 6.3
DDO-5936		>50	>50

**Fig. 4:**
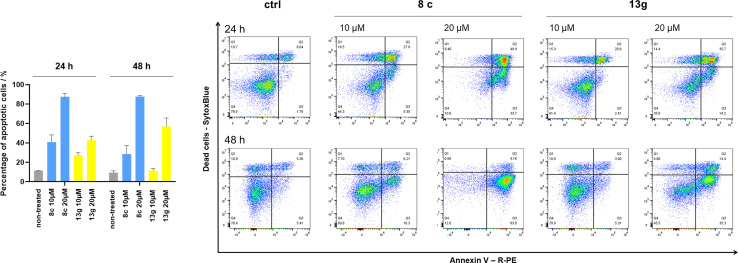
Induction of apoptosis in SK-N-MC cells by compounds 8c and 13g. SK-N-MC cells were exposed to 10 μM or 20 μM 8c or 13g. After 24 h or 48 h, apoptosis was measured by annexin V–PE/SytoxBlue staining. Percentages apoptotic cells – left, and representative scatter plots of annexin V–PE (*x*-axis) and Sytox blue (*y*-axis) – right. Data in the bar charts are presented as the means ± SD of at least three biological replicates.

Cells were exposed to compounds 8c and 13g at concentrations of 10 μM and 20 μM for 24 and 48 hours (Fig. 4). Both compounds induced apoptosis in SK-N-MC cancer cells. Similar to known inhibitors of Hsp90–Cdc37 PPI FW-04-806 and celastrol that induced apoptosis in breast^37^ and pancreatic^50^ cancer cells, respectively, an increase in apoptotic cell portion was observed with compounds 8c and 13g. Compound 8c significantly induced apoptosis at both concentrations and time-points. The proportion of apoptotic cells when exposed to 8c at 20 μM was extensive already after 24 hours (87.5% *vs.* 11.4% for the vehicle control) and remained high after 48 hours treatment (87.7% *vs.* 9.5% for the vehicle control). After treatment with 13g at 20 μM, the proportion of apoptotic cells was 42.8% and 56.8% after 24 hours and 48 hours, respectively. When treated with 10 μM compound 13g, cells initially underwent apoptotic cell death (27% of cells); however, they appeared to recover after 48 hours (proportion of apoptotic cells 11.4%), and the proportion of apoptotic cells here was not significantly different from the vehicle control. Based on these results, compound 8c is more efficient in inducing cell death. These findings are consistent with results from the proliferation assay, where 8c exhibited higher cytotoxicity compared to 13g in SK-N-MC cells.

### Investigation of the mechanism of action

**Fig. 5:**
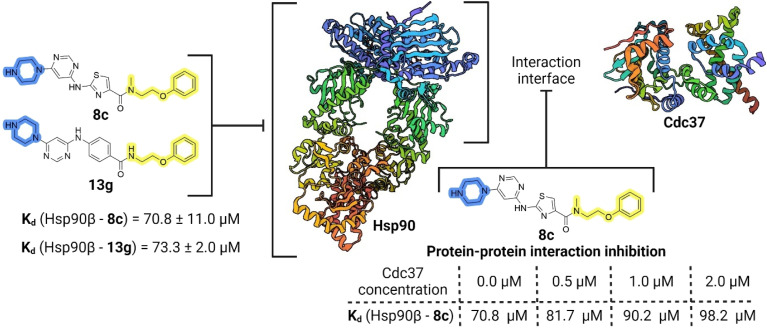
Schematic representation of binding of 8c and 13g to Hsp90 and PPI inhibition investigation for 8c.

Compounds 8c and 13g were selected to examine the mode of action of the prepared compounds, and their binding to full-length Hsp90β was studied using microscale thermophoresis (MST). Both compounds bound to Hsp90β with an affinity in the micromolar range, with *K*_d_ values of 70.8 ± 11.0 μM for 8c and 73.3 ± 2.0 μM for 13g (Fig. 5, S2A and S3A†). The binding affinity of 8c for Hsp90β was further investigated in the presence of increasing concentrations of the full-length Cdc37. In three consecutive experiments, the concentration of Cdc37 was increased from the initial 0.5 μM to 1.0 μM and finally to 2.0 μM, reaching the reported *K*_d_ range of Cdc37 for Hsp90.^45,51^ The addition of Cdc37 resulted in a concentration-dependent decrease in the affinity of 8c for Hsp90β, with *K*_d_ values increasing from 70.8 μM in the absence of Cdc37 to 81.7 μM (0.5 μM Cdc37), 90.2 μM (1 μM Cdc37), and 98.2 μM (2 μM Cdc37) (Fig. 5 and S3B–D†). This indicated that 8c binds to Hsp90β in the area of the Hsp90–Cdc37 interaction interface, thereby disrupting their PPI.^52^ Additionally, when the affinity of 13g for Hsp90β was tested in the presence of 1.0 μM Cdc37, the binding affinity within the applied concentration range was too low for quantification (Fig. S2B†). These results suggest that compounds 8c and 13g are displaced from Hsp90β by Cdc37, indicating their potential to disrupt the Hsp90–Cdc37 interaction.

**Fig. 6:**
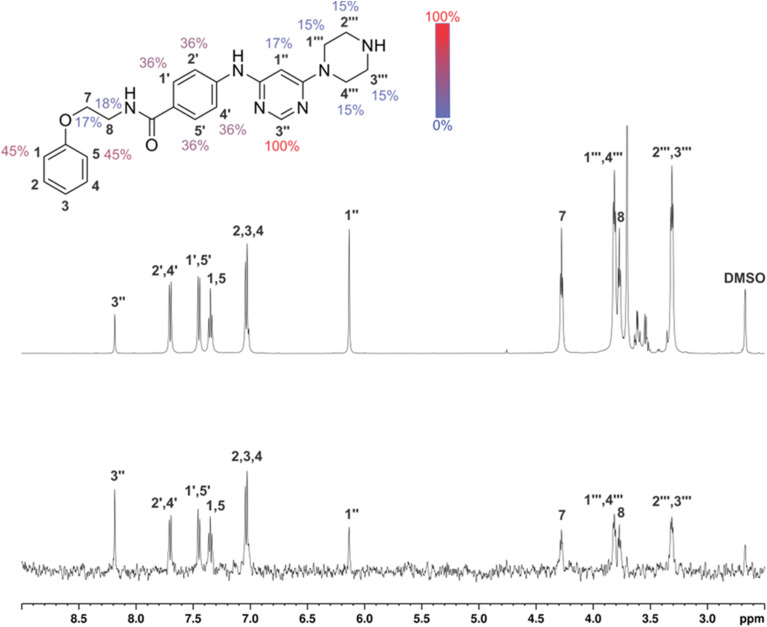
1D ^1^H STD NMR spectra for the compound 13g recorded at an Hsp90β : ligand ratio of 1 : 200 and 600 MHz. The molecular structure illustrates the proton nomenclature and the color-coded relative degrees of saturation of the individual protons. The STD amplification factors were normalized to the intensity of the signal with the largest STD effect. Reference STD spectra (top) with proton assignment and difference STD spectra (bottom) are shown. The unassigned proton signals between 3.5 and 3.8 ppm belong to the protein buffer with glycerol. The proton signals were calibrated to the DSS signal at 0.0 ppm. The spectra are not to scale.

In the absence of the experimentally determined binding site of these Hsp90–Cdc37 inhibitors, the binding of 13g to the full-length Hsp90β was explored by saturation transfer difference (STD) NMR spectroscopy under quantitative conditions. The STD amplification factors (Fig. 6) indicate that the aromatic regions of 3-(phenylamino)pyrimidine and peripheral phenyl ring of 13g are in the closest proximity with the Hsp90β binding site. Meanwhile, the contributions of the aliphatic protons in the linker between the two phenyl rings were slightly weaker. Similarly, the piperazine ring forms weaker contacts with the protein than the aromatic parts, but this part of the inhibitor also plays an important role according to the SAR of library D.

**Fig. 7:**
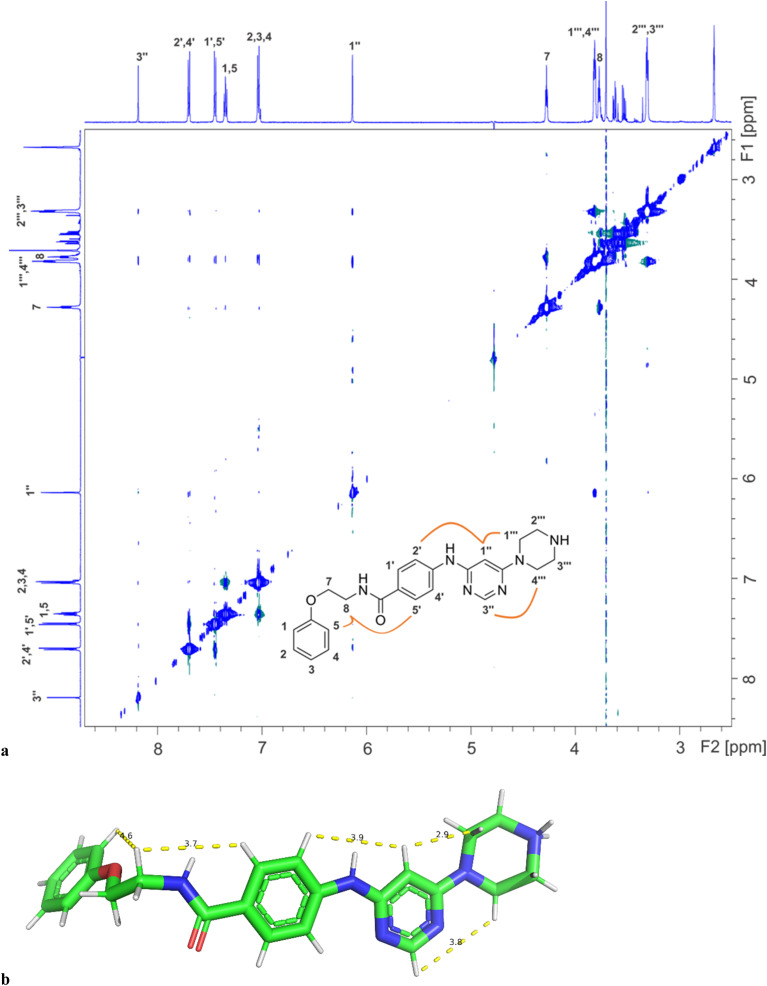
(a) The trNOESY spectrum of 13g in the presence of Hsp90β with the molecular structure illustrating the atom nomenclature and the NOE connectivities between the protons of the different molecular segments. The NOE connectivities of the magnetically equivalent protons with overlapping signals are shown schematically for one orientation of the corresponding rings only. (b) Calculated conformation of 13g based on distance constraints from trNOESY experiment (Table S4†).

Binding of inhibitors 13g to Hsp90β was further confirmed by transferred NOESY (trNOESY) experiments, as negative NOEs with the same sign as the diagonal peaks were observed between the protons of inhibitor in the presence of Hsp90β (Fig. 7a). The NOEs were observed only between adjacent molecular segments, suggesting that 13g adopts an extended conformation when bound to Hsp90β. The calculated conformation of 13g using the distance constraints from trNOESY spectrum is presented in Fig. 7b.

**Fig. 8:**
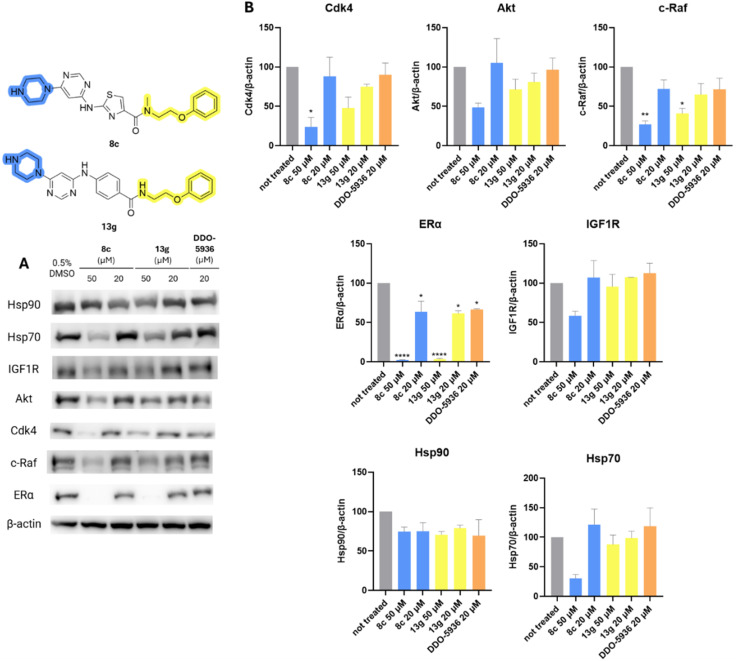
Western blot analysis of effects of DDO-5936 (20 μM), 8c and 13g (20 μM and 50 μM) in MCF-7 cell line on Hsp90 client protein levels (Cdk4, Akt, c-Raf, ERα, IGF1R) and representative heat shock proteins (Hsp70, Hsp90) after 24 hours of treatment. (A) Representative western blot images; (B) quantification results normalized on β-actin levels. The bars represent mean values with SD and Welch's *t*-test was used to evaluate statistical significance (*p < 0.05, **p < 0.01, ****p < 0.0001). Full images used for quantification are shown in ESI Fig. S4 and S5.†

The effect of Hsp90–Cdc37 PPI inhibitors 8c, 13g, and DDO-5936 on Hsp90 client protein levels was investigated in MCF-7 cells. As shown in Fig. 8, both 8c and 13g decreased the concentration of kinase clients Cdk4, c-Raf, IGF1R, and Akt at 50 μM, with varying statistical significance. The effect on client proteins was more pronounced for compound 8c, with c-Raf and Cdk4 being the most affected. Surprisingly, the decrease was most pronounced for ERα, as it was the only Hsp90 client protein significantly influenced even at the lower 20 μM concentration of 8c and 13g. ERα was also the only client protein that was downregulated by DDO-5936 with statistical significance. This was unexpected, as Cdc37 is primarily reported to be important for the kinome-related part of the Hsp90 clientele.^28,32^ However, the androgen receptor^53^ and guanylyl cyclase receptor^48^ have also been shown to rely on Cdc37 for recruitment to Hsp90, although they are not protein kinases. Additionally, Hsp70 and Hsp90 levels were examined to assess the heat shock response. Importantly, no increase in either heat shock protein was observed. On the contrary, a statistically insignificant decrease in Hsp70 levels was noted, especially at 50 μM of 8c, indicating another potential benefit of inhibiting Hsp90–Cdc37 PPI over classical N-terminal Hsp90 inhibition.^34^

## Conclusion

In the present work, we have demonstrated the viability of using ligand-based virtual screening to design new inhibitors of the Hsp90–Cdc37 PPI, and thus demonstrated that the current pool of known inhibitors enables this design approach. Our initial hit compounds exhibited anticancer potency, which we further optimized in our SAR studies. However, as observed in previous studies, firmly establishing SAR has been challenging. Additional exploration of the exact binding site of these inhibitors at the Hsp90–Cdc37 interface using full-length proteins would greatly benefit the research community. We have demonstrated that compounds 8c and 13g both bind to Hsp90 in the mid-micromolar range, interfere with the Hsp90–Cdc37 PPI, and exhibit *in vitro* anticancer activity against Ewing sarcoma, breast cancer, and leukemia cell lines. Additionally, they effectively decrease the levels of known Hsp90–Cdc37 client proteins, such as protein kinases Cdk4 and c-Raf. Importantly, compounds 8c and 13g do not induce a heat shock response, a known obstacle for N-terminal Hsp90 inhibitors in their clinical development. This absence of HSR makes the Hsp90–Cdc37 PPI and its inhibitors clinically significant and worthy of further investigation.

## Methods

### Library preparation

A library of 556 000 compounds from commercial providers was prepared based on the diversity sets from Enamine, Asinex, ChemBridge, Maybridge, LifeChemicals, Vitas-M and KeyOrganics. Libraries were downloaded in SDF format, merged and duplicates removed using the LigandScout Database Merger and Duplicates Remover nodes as implemented in the Inte : Ligand Expert KNIME Extensions.

The 14 most potent Hsp90–Cdc37 PPI inhibitors, with *K*_d_ values ranging from 0.50 μM to 20 μM, were used as a training set of active compounds for building a ligand-based pharmacophore model.^49^ In addition, we also created a library of inactive compounds (15 compounds, inhibition rate less than 50% at 100 μM).^45–47^

For each library, a maximum of 200 conformations were generated for each molecule using LigandScout's iCon algorithm with the default “BEST” settings [max. number of conformers per molecules: 200, timeout (s): 600, RMS threshold: 0.8, energy window: 20.0, max. pool size: 4000, max. fragment build time: 30]. Each library was saved in LDB (LigandScout database format) using LigandScout's idbgen algorithm with default settings (write all properties and remove duplicates).

### Ligand-based pharmacophore modeling

Four most potent Hsp90–Cdc37 PPI inhibitors^47^ were used for creation of ten ligand-based pharmacophore models in LigandScout 4.4 Expert. The models were generated using the following ligand-based pharmacophore creation settings: scoring function: pharmacophore fit and atom overlap; pharmacophore type: merged feature pharmacophore; number of omitted features for merged pharmacophore: 4; partially matching features optional, threshold (%): 10.0; feature tolerance scale factor: 1.0; maximum number of result pharmacophores: 10. Create exclusion volumes and apply custom feature definitions was ticked on in the tick box. Creation of an exclusion volumes coat around the alignment of the ligands was also enabled for each of the models. All ligands of the training set were automatically aligned to the generated pharmacophore models. The resulting ligand-based pharmacophore models were tested for their performance in the distinguishing the active and decoy compounds. The best performing model was selected for virtual screening of the commercially available compounds.

### Virtual screening

The selected ligand-based pharmacophore model was used to query the library of 556 000 commercially available diverse compounds, prepared as described using LigandScout 4.4 Expert. The settings included: scoring function: pharmacophore-fit, screening mode: match all query features; retrieval mode: stop after first matching conformation; max. number of omitted features: 0 and check exclusion volumes: true. Virtual hits were ranked according to pharmacophore fit score. Three virtual screening hits LAS 58000795 (1), LAS 58000810 (2) and LAS 58000778 (3) were purchased from Asinex.

### Synthesis and characterization

To perform the synthesis reagents and solvents used were acquired from various vendors like Sigma-Aldrich (St. Louis, MO, USA), Enamine Ltd (Kyiv, Ukraine), and Fluorochem Ltd (Derbyshire, UK), and were used without any further purification. To determine the retention factors analytical thin-layer chromatography silica gel aluminum sheets were used (0.20 mm; 60 F254; Merck, Darmstadt, Germany). To purify the compounds with flash column chromatography silica gel 60 (particle size, 230–400 mesh) was used as the stationary phase. For recording the NMR spectra (^1^H and ^13^C) to analyte the chemical compounds 400 MHz NMR spectrometer (Bruker Advance 3, Bruker, Billerica, MA, USA) was used. The splitting pattern designation was as follows: s, singlet; d, doublet; dd, double doublet; t, triplet; dt, double triplet; ddd, double of doublet of doublet; q, quartet; and m, multiplet. For the monitoring of purity of the final products ultra-high performance liquid chromatography (UHPLC) (Thermo Scientific Dionex UltiMate 3000 UHPLC modular system, Thermo Fischer Scientific Inc., Waltham, MA, USA) was used, which was equipped with a wavelength detector to perform the purity determination at 254 nm. The method used for purity determination was set up as follows: C_18_ column (Waters Acquity UPLC® HSS C18 SB column, 2.1 mm × 50 mm, 1.8 μm); temperature: 40 °C; injection volume 5 μL; flow rate: 0.3 mL min^−1^. The mobile phase consisted of varying portions of Solvent A (0.1% TFA in H_2_O) and Solvent B (MeCN), using the gradient as follows: 0 → 7 min 5% B; 7 → 8 min 95% B. Mass spectra were recorded using expression CMS^L^ mass spectrometer (Advion Inc., Ithaca, NY, USA), while to obtain high-resolution mass spectra Exactive Plus Orbitrap mass spectrometer (Thermo Scientific Inc., Waltham, MA, USA) was used. DDO-5936 was purchased from MedChemExpress.

### General procedure A

The corresponding carboxylic acid (1 equiv.) was dissolved in *N*,*N*-dimethylformamide (DMF) (20 mL). The solution was put on an ice bath and EDC (1.3 equiv.), HOBt (1.3 equiv.) and *N*-methylmorpholine (NMM, 2 equiv.) were added. The reaction was stirred at 0 °C for 20 minutes. The corresponding amine (1 equiv.) was then added, and the reaction mixture was stirred overnight at room temperature. The solvent was evaporated, and the residue was taken up in EtOAc (100 mL) and NaHCO_3_ (50 mL). The layers were separated, and the organic layer was further washed with NaHCO_3_ (2 × 50 mL) and 1% citric acid (3 × 30 mL). The organic layer was washed with brine (40 mL), then dried over Na_2_SO_4_, filtered and the solvent evaporated under reduced pressure.

### General procedure B

The corresponding 4-substituted 2-aminothiazole (1 equiv.) and 4,6-dichloropyrimidine (1 equiv.) were dissolved in dry THF (20 mL). Then 60% NaH on mineral oil (2.5 equiv.) was added on an ice bath. The temperature was gradually increased to ambient temperature and then the reaction mixture stirred for 3 hours at room temperature. Then, saturated NH_4_Cl_(aq)_ (40 mL) was added, and the mixture was concentrated under reduced pressure. EtOAc (50 mL) was added to the residue and the layers were separated. The aqueous layer was additionally extracted with EtOAc (50 mL). The combined organic phases were washed with brine (30 mL), dried over Na_2_SO_4_, filtered, and then the solvent evaporated under reduced pressure. The products were additionally purified by flash column chromatography.

### General procedure C

The corresponding 6-chloro-*N*-substitutedpyrimidin-4-amine (1 equiv.) and respective amines (1 equiv.) were dissolved in DMF (10 mL). In the case of pyrrolidine (7 equiv.), the reaction mixture was stirred at 70 °C overnight. In the case of *N*-Boc-piperazine, piperidine, morpholine, 4-hydroxypiperidine and 4-(*N*-Boc-amino)piperidine (5 equiv.), Et_3_N (2 equiv.) was also added to the reaction mixture, which was then heated to 80 °C and left to stir over three days for 4-(*N*-Boc-amino)piperidine and *N*-Boc-piperazine and overnight for pyrrolidine, morpholine and 4-hydroxypiperidine. Next, the volatiles were evaporated, and the residue was taken up in ethyl acetate (30 mL) and washed with 1% citric acid (3 × 20 mL), brine (20 mL), dried over Na_2_SO_4_, filtered, and the solvent evaporated under reduced pressure. When necessary, the crude products were additionally purified by flash column chromatography.

### General procedure D

The Boc-protected amine (1 equiv.) was dissolved in DCM (10 mL) and TFA (20 equiv.) was added. The reaction mixture was stirred overnight at room temperature. The solvent was evaporated and the product was purified by flash column chromatography.

### General procedure E

The *meta*- or *para*-substituted aminobenzamides (1 equiv.) and 4,6-dichloropyrimidine (1.5 equiv.) were dissolved in DMF (30 mL). Then, DIPEA (2 equiv.) was added, and the reaction mixture was heated to 100 °C and left to stir for 3 days. The solvent was evaporated under reduced pressure and the residue was taken up in EtOAc (50 mL) and water (50 mL). The layers were separated, and organic layer was additionally washed with water (50 mL), brine (30 mL), dried over Na_2_SO_4_, filtered, and the solvent evaporated under reduced pressure. The product was additionally purified by flash column chromatography.

#### 2-Amino-*N*-benzyl-*N*-methylthiazole-4-carboxamide (4a)

Compound 4a was prepared according to the general procedure A using 2-aminothiazole-4-carboxylic acid (609 mg, 4.23 mmol) as the starting material. Yield: 61% (637 mg); yellow solidified oil; *R*_f_ (DCM : MeOH = 9 : 1) = 0.46; ^1^H NMR (400 MHz, DMSO-*d*_6_) *δ* 7.40–7.31 (m, 2H), 7.31–7.19 (m, 3H), 7.12 (s, 2H), 7.02 (s, 1H), 5.02–4.48 (m, 2H), 3.15–2.72 (m, 3H) ppm; ^13^C NMR (101 MHz, DMSO-*d*_6_) *δ* 167.8, 164.3, 146.0, 137.8, 137.5, 128.5, 127.4, 127.2, 111.5, 110.8, 53.1, 50.3, 36.2, 33.1 ppm; MS (ESI+) C_12_H_13_N_3_OS *m*/*z*: 247.8 [M + H]^+^.

#### 2-Amino-*N*-methyl-*N*-phenethylthiazole-4-carboxamide (4b)

Compound 4a was prepared according to the general procedure A using 2-aminothiazole-4-carboxylic acid (750 mg, 5.20 mmol) as the starting material. Yield: 62% (843 mg); yellow solidified oil; *R*_f_ (DCM : MeOH = 9 : 1) = 0.44; ^1^H NMR (400 MHz, DMSO-*d*_6_) *δ* 7.35–7.03 (m, 7H), 6.94–6.70 (m, 1H), 3.73–3.51 (m, 2H), 3.09–2.91 (m, 3H), 2.88–2.80 (m, 2H) ppm; MS (ESI+) C_13_H_15_N_3_OS *m*/*z*: 261.4 [M + H]^+^.

#### 2-Amino-*N*-methyl-*N*-(2-phenoxyethyl)thiazole-4-carboxamide (4c)

Compound 4c was prepared according to the general procedure A using 2-aminothiazole-4-carboxylic acid (601 mg, 4.17 mmol) as the starting material. Yield: 68% (783 mg); yellow solidified oil; *R*_f_ (DCM : MeOH = 9 : 1) = 0.43; ^1^H NMR (400 MHz, DMSO-*d*_6_) *δ* 7.34–7.24 (m, 2H), 7.11 (s, 2H), 7.00–6.86 (m, 4H), 4.15 (t, *J* = 5.8 Hz, 2H), 3.97–3.68 (m, 2H), 3.28–2.95 (m, 3H) ppm; MS (ESI+) C_13_H_15_N_3_O_2_S *m*/*z*: 277.6 [M + H]^+^.

#### *N*-Benzyl-2-((6-chloropyrimidin-4-yl)amino)-*N*-methylthiazole-4-carboxamide (5a)

Compound 5a was prepared according to the general procedure B using 4a (340 mg, 1.37 mmol) as the starting material. DCM : MeOH = 30 : 1 was used as the eluent for flash column chromatography. Yield: 35% (174 mg); red solid; *R*_f_ (DCM : MeOH = 30 : 1) = 0.32; ^1^H NMR (400 MHz, DMSO-*d*_6_) *δ* 9.55 (s, 1H), 8.97–8.46 (m, 1H), 8.42–8.34 (m, 1H), 7.52–7.26 (m, 5H), 7.00–6.69 (m, 1H), 4.86–4.47 (m, 2H), 3.17–2.71 (m, 3H) ppm; ^13^C NMR (101 MHz, DMSO-*d*_6_) *δ* 164.1, 158.5, 158.0, 157.9, 157.4, 145.0, 137.3, 128.6, 127.6, 127.3, 117.9, 117.5, 106.2, 53.3, 50.3, 36.4, 33.2 ppm; MS (ESI+) C_16_H_14_ClN_5_OS *m*/*z*: 360.2 [M + H]^+^.

#### 2-((6-Chloropyrimidin-4-yl)amino)-*N*-methyl-*N*-phenethylthiazole-4-carboxamide (5b)

Compound 5b was prepared according to the general procedure B using 4b (400 mg, 1.53 mmol) as the starting material. DCM : MeOH = 30 : 1 was used as the eluent for flash column chromatography. Yield: 42%; red solid; *R*_f_ (DCM : MeOH = 20 : 1) = 0.19; ^1^H NMR (400 MHz, DMSO) *δ* 12.27–11.95 (m, 1H), 8.74 (d, *J* = 1.0 Hz, 1H), 7.35–7.06 (m, 7H), 3.77–3.59 (m, 2H), 3.16–2.95 (m, 3H), 2.93–2.80 (m, 2H) ppm; MS (ESI−) C_17_H_16_ClN_5_OS *m*/*z*: 372.3 [M − H]^−^.

#### 2-((6-Chloropyrimidin-4-yl)amino)-*N*-methyl-*N*-(2-phenoxyethyl)thiazole-4-carboxamide (5c)

Compound 5c was prepared according to the general procedure B using 4c (450 mg, 1.62 mmol) as the starting material. DCM : MeOH = 20 : 1 was used as the eluent for flash column chromatography. Yield: 39% (246 mg); red solid; *R*_f_ (DCM : MeOH = 20 : 1) = 0.19; ^1^H NMR (400 MHz, CDCl_3_) *δ* 9.63 (s, 1H), 8.68 (d, *J* = 1.1 Hz, 1H), 7.62–7.34 (m, 1H), 7.32–7.25 (m, 2H), 7.02–6.93 (m, 2H), 6.87 (s, 2H), 4.25 (s, 2H), 4.11–3.86 (m, 2H), 3.42–3.13 (m, 3H) ppm; MS (ESI+) C_17_H_16_ClN_5_O_2_S *m*/*z*: 389.6 [M + H]^+^.

#### *N*-Benzyl-*N*-methyl-2-((6-(pyrrolidin-1-yl)pyrimidin-4-yl)amino)thiazole-4-carboxamide (6a)

Compound 6a was prepared according to the general procedure C using 5a (45 mg, 0.125 mmol) as the starting material. DCM : MeOH = 20 : 1 was used as the eluent for flash column chromatography. Yield: 83% (41 mg); light yellow solid; *R*_f_ (DCM : MeOH = 20 : 1) = 0.17; ^1^H NMR (400 MHz, DMSO-*d*_6_) *δ* 11.29–11.15 (m, 1H), 8.30 (s, 1H), 7.46 (d, *J* = 0.9 Hz, 1H), 7.42–7.20 (m, 3H), 5.95 (d, *J* = 12.5 Hz, 1H), 4.95 (s, 1H), 4.66 (s, 1H), 3.13–2.80 (m, 3H), 1.93 (s, 4H) ppm, signal for the remaining four protons is overlapped with solvent; ^13^C NMR (101 MHz, DMSO-*d*_6_) *δ* 164.4, 162.3, 160.6, 159.9, 158.8, 156.6, 156.4, 144.7, 137.4, 128.6, 127.6, 127.4, 116.6, 115.9, 84.7, 53.3, 50.3, 46.0, 36.3, 35.8, 33.2, 30.8, 24.8, 23.8 ppm, rotamers are present in the spectrum; HRMS (ESI+) calcd for C_20_H_23_ON_6_S *m*/*z* [M + H]^+^: 395.16486, found: 395.16480; UHPLC: *t*_r_: 3.36 min (98.2% at 254 nm).

#### *N*-Methyl-*N*-phenethyl-2-((6-(pyrrolidin-1-yl)pyrimidin-4-yl)amino)thiazole-4-carboxamide (6b)

Compound 6b was prepared according to the general procedure C using 5b (60 mg, 0.160 mmol) as the starting material. DCM : MeOH = 20 : 1 was used as the eluent for flash column chromatography. Yield: 66% (43 mg); yellow solid; *R*_f_ (DCM : MeOH = 9 : 1) = 0.53; ^1^H NMR (400 MHz, DMSO-*d*_6_) *δ* 11.36–11.07 (m, 1H), 8.31 (s, 1H), 7.39–7.09 (m, 6H), 5.97 (s, 1H), 3.79–3.59 (m, 2H), 3.18–2.82 (m, 5H), 1.93 (s, 4H) ppm, signal for the remaining four protons is covered with solvent; ^13^C NMR (101 MHz, DMSO-*d*_6_) *δ* 164.6, 163.9, 159.9, 158.6, 156.6, 156.5, 144.9, 139.2, 138.8, 128.7, 128.4, 126.2, 115.7, 114.9, 84.7, 51.8, 49.2, 46.0, 36.8, 34.4, 33.4, 32.7, 24.8 ppm, rotamers are present in the spectra; HRMS (ESI+) calcd for C_21_H_25_ON_6_S *m*/*z* [M + H]^+^: 409.18051, found: 409.18036; UHPLC: *t*_r_: 3.43 min (93.1% at 254 nm).

#### *N*-Methyl-*N*-(2-phenoxyethyl)-2-((6-(pyrrolidin-1-yl)pyrimidin-4-yl)amino)thiazole-4-carboxamide (6c)

Compound 6c was prepared according to the general procedure C using 5c (60 mg, 0.154 mmol) as the starting material. DCM : MeOH = 25 : 1 was used as the eluent for flash column chromatography. Yield: 43% (28 mg); yellow solid; *R*_f_ (DCM : MeOH = 20 : 1) = 0.20; ^1^H NMR (400 MHz, DMSO-*d*_6_) *δ* 11.31–11.10 (m, 1H), 8.31 (s, 1H), 7.41 (s, 1H), 7.29–7.21 (m, 2H), 7.01–6.85 (m, 3H), 5.94 (s, 1H), 4.18 (t, *J* = 5.6 Hz, 2H), 4.05–3.74 (m, 2H), 3.31–2.99 (m, 5H), 1.93 (s, 4H) ppm, signals for the remaining two protons are covered with solvent; ^13^C NMR (101 MHz, DMSO-*d*_6_) *δ* 164.7, 164.3, 160.6, 159.9, 158.7, 158.6, 158.1, 156.6, 156.5, 156.4, 144.8, 129.5, 120.7, 116.1, 114.4, 84.7, 65.9, 65.3, 49.2, 47.1, 46.0, 38.0, 34.3, 24.8 ppm; rotamers are present in the spectrum; HRMS (ESI+) calcd for C_21_H_25_O_2_N_6_S *m*/*z* [M + H]^+^: 425.17542, found: 425.17534; UHPLC: *t*_r_: 3.46 min (99.2% at 254 nm).

#### *tert*-Butyl 4-(6-((4-(benzyl(methyl)carbamoyl)thiazol-2-yl)amino)pyrimidin-4-yl)piperazine-1-carboxylate (7a)

Compound 7a was prepared according to the general procedure C using 5a (100 mg, 0.278 mmol) as the starting material. DCM : MeOH = 20 : 1 was used as the eluent for flash column chromatography. Yield: 94% (133 mg); red solid; *R*_f_ (DCM : MeOH = 30 : 1) = 0.26; ^1^H NMR (400 MHz, DMSO-*d*_6_) *δ* 11.67–11.15 (m, 1H), 8.37 (s, 1H), 7.52–7.45 (m, 1H), 7.41–7.20 (m, 5H), 6.21 (s, 1H), 5.09–4.56 (m, 2H), 3.56–3.49 (m, 4H), 3.45–3.39 (m, 4H), 3.10–2.78 (m, 3H), 1.42 (s, 9H) ppm; MS (ESI+) C_25_H_31_N_7_O_3_S *m*/*z*: 509.8 [M + H]^+^.

#### *tert*-Butyl 4-(6-((4-(methyl(phenethyl)carbamoyl)thiazol-2-yl)amino)pyrimidin-4-yl)piperazine-1-carboxylate (7b)

Compound 7b was prepared according to the general procedure C using 5b (160 mg, 0.428 mmol) as the starting material. Yield: quantitative; red solid; *R*_f_ (DCM : MeOH = 30 : 1) = 0.05; ^1^H NMR (400 MHz, DMSO-*d*_6_) *δ* 11.31 (d, *J* = 24.7 Hz, 1H), 8.37 (s, 1H), 7.48–7.00 (m, 6H), 6.23 (s, 1H), 3.75–3.58 (m, 2H), 3.57–3.50 (m, 4H), 3.46–3.40 (m, 4H), 3.12–2.95 (m, 3H), 2.91–2.82 (m, 2H), 1.42 (s, 9H) ppm; MS (ESI+) C_26_H_33_N_7_O_3_S *m*/*z*: 523.5 [M + H]^+^.

#### *tert*-Butyl 4-(6-((4-(methyl(2-phenoxyethyl)carbamoyl)thiazol-2-yl)amino)pyrimidin-4-yl)piperazine-1-carboxylate (7c)

Compound 7c was prepared according to the general procedure C using 5c (170 mg, 0.436 mmol) as the starting material. DCM : MeOH = 25 : 1 was used as the eluent for flash column chromatography. Yield: 78% (183 mg); yellow solid; *R*_f_ (DCM : MeOH = 20 : 1) = 0.29; ^1^H NMR (400 MHz, DMSO-*d*_6_) *δ* 11.48–11.08 (m, 1H), 8.37 (s, 1H), 7.43 (d, *J* = 0.7 Hz, 1H), 7.32–7.22 (m, 2H), 6.99–6.88 (m, 3H), 6.19 (s, 1H), 4.17 (s, 2H), 4.02–3.74 (m, 2H), 3.56–3.50 (m, 4H), 3.46–3.40 (m, 4H), 3.30–2.99 (m, 3H), 1.42 (s, 9H) ppm; ^13^C NMR (101 MHz, DMSO-*d*_6_) *δ* 164.7, 161.9, 161.1, 158.5, 158.3, 158.1, 157.4, 156.6, 153.9, 153.7, 144.8, 129.5, 120.7, 116.3, 114.4, 85.0, 79.3, 79.2, 65.8, 65.3, 49.2, 47.1, 44.6, 43.2, 38.0, 34.24 28.1, 28.0 ppm; MS (ESI+) C_26_H_33_N_7_O_4_S *m*/*z*: 539.6 [M + H]^+^.

#### *N*-Benzyl-*N*-methyl-2-((6-(piperazin-1-yl)pyrimidin-4-yl)amino)thiazole-4-carboxamide (8a)

Compound 8a was prepared according to the general procedure D using 7a (90 mg, 0.177 mmol) as the starting material. DCM : MeOH : NH_4_OH = 9 : 1 : 0.1 was used as the eluent for flash column chromatography. Yield: 58% (42 mg); yellow solid; *R*_f_ (DCM : MeOH : NH_4_OH = 9 : 1 : 0.1) = 0.06; ^1^H NMR (400 MHz, DMSO-*d*_6_) *δ* 11.26 (s, 2H), 8.34 (s, 1H), 7.47 (s, 1H), 7.41–7.17 (m, 5H), 6.18 (s, 1H), 5.00–4.57 (m, 2H), 3.47–3.37 (m, 4H), 3.08–2.82 (m, 3H), 2.79–2.72 (m, 4H) ppm; ^13^C NMR (101 MHz, DMSO-*d*_6_) *δ* 164.4, 162.1, 160.8, 158.7, 157.3, 156.6, 144.7, 137.6, 137.4, 128.6, 127.6, 127.4, 116.7, 116.1, 84.8, 53.3, 50.3, 46.3, 46.1, 45.1, 44.6, 40.6, 36.3, 33.2 ppm, rotamers are present in the spectrum; HRMS (ESI+) calcd for C_20_H_24_ON_7_S *m*/*z* [M + H]^+^: 410.17576, found: 410.17554; UHPLC: *t*_r_: 2.89 min (100% at 254 nm).

#### *N*-Methyl-*N*-phenethyl-2-((6-(piperazin-1-yl)pyrimidin-4-yl)amino)thiazole-4-carboxamide (8b)

Compound 8b was prepared according to the general procedure D using 7b (100 mg, 0.191 mmol) as the starting material. DCM : MeOH : NH_4_OH = 9 : 1 : 0.1 was used as the eluent for flash column chromatography. Yield: 49% (40 mg); light yellow solid; *R*_f_ (DCM : MeOH : NH_4_OH = 8 : 1 : 0.1) = 0.14; ^1^H NMR (400 MHz, DMSO-*d*_6_) *δ* 11.31 (s, 1H), 8.37 (s, 1H), 7.42–7.08 (m, 6H), 6.23 (s, 1H), 3.74–3.47 (m, 6H), 3.12–2.82 (m, 9H) ppm; ^13^C NMR (101 MHz, DMSO-*d*_6_) *δ* 164.5, 163.9, 161.9, 158.5, 157.4, 156.6, 144.9, 138.7, 128.7, 128.6, 128.4, 126.2, 115.9, 115.1, 85.0, 51.7, 49.1, 44.1, 43.2, 36.8, 34.4, 33.4, 32.7 ppm, rotamers are present in the spectrum; HRMS (ESI+) calcd for C_21_H_26_ON_7_S *m*/*z* [M + H]^+^: 424.19141, found: 424.19100; UHPLC: *t*_r_: 2.99 min (93.9% at 254 nm).

#### *N*-Methyl-*N*-(2-phenoxyethyl)-2-((6-(piperazin-1-yl)pyrimidin-4-yl)amino)thiazole-4-carboxamide (8c)

Compound 8c was prepared according to the general procedure D using 7c (100 mg, 0.185 mmol) as the starting material. DCM : MeOH : NH_4_OH = 8 : 1 : 0.1 was used as the eluent for flash column chromatography. Yield: 60% (49 mg); light yellow solid; *R*_f_ (DCM : MeOH : NH_4_OH = 8 : 1 : 0.1) = 0.10; ^1^H NMR (400 MHz, DMSO-*d*_6_) *δ* 11.39–11.09 (m, 1H), 8.34 (s, 1H), 7.42 (s, 1H), 7.32–7.22 (m, 2H), 6.98–6.84 (m, 3H), 6.17 (d, *J* = 1.1 Hz, 1H), 4.17 (s, 2H), 4.03–3.71 (m, 2H), 3.48–3.41 (m, 4H), 3.28–3.00 (m, 3H), 2.78–2.71 (m, 4H) ppm; ^13^C NMR (101 MHz, DMSO-*d*_6_) *δ* 164.8, 164.3, 162.1, 158.6, 158.1, 157.3, 156.6, 144.8, 129.5, 120.7, 116.2, 114.4, 84.7, 65.8, 65.3, 49.2, 47.1, 45.2, 44.7, 38.0, 34.3 ppm, rotamers are present in the spectrum; HRMS (ESI+) calcd for C_21_H_26_O_2_N_7_S *m*/*z* [M + H]^+^: 440.18632, found: 440.18609; UHPLC: *t*_r_: 3.02 min (100% at 254 nm).

#### 3-Amino-*N*-phenethylbenzamide (9a)

Compound 9a was prepared according to the general procedure A using 3-aminobenzoic acid (896 mg, 6.54 mmol) as the starting material. The product was additionally purified by column chromatography using DCM : MeOH = 20 : 1 as the eluent. Yield: 31% (489 mg); light brown solid; *R*_f_ (DCM : MeOH = 20 : 1) = 0.35; ^1^H NMR (400 MHz, DMSO-*d*_6_) *δ* 8.31 (t, *J* = 5.5 Hz, 1H), 7.34–7.25 (m, 2H), 7.27–7.15 (m, 3H), 7.05 (t, *J* = 7.8 Hz, 1H), 7.00 (t, *J* = 2.0 Hz, 1H), 6.91 (dt, *J*_1_ = 7.9 Hz, *J*_2_ = 1.3 Hz, 2H), 6.67 (ddd, *J*_1_ = 7.9 Hz, *J*_2_ = 2.3 Hz, *J*_3_ = 0.8 Hz, 2H), 5.22 (s, 2H), 3.48–3.38 (m, 2H), 2.82 (t, *J* = 7.5 Hz, 2H) ppm; MS (ESI+) C_15_H_16_N_2_O *m*/*z*: 240.7 [M + H]^+^.

#### 3-Amino-*N*-(2-phenoxyethyl)benzamide (9b)

Compound 9b was prepared according to the general procedure A using 3-aminobenzoic acid (760 mg, 5.54 mmol) as the starting material. Yield: 85% (1.20 g); brown oil; *R*_f_ (DCM : MeOH = 9 : 1) = 0.61; ^1^H NMR (400 MHz, DMSO-*d*_6_) *δ* 8.44 (t, *J* = 5.5 Hz, 1H), 7.33–7.24 (m, 2H), 7.10–7.02 (m, 2H), 7.00–6.90 (m, 5H), 6.68 (ddd, *J*_1_ = 7.9 Hz, *J*_2_ = 2.3 Hz, *J*_3_ = 0.8 Hz, 1H), 5.26 (s, 2H), 4.08 (t, *J* = 6.0 Hz, 2H), 3.58 (q, *J* = 5.9 Hz, 2H) ppm; MS (ESI+) C_15_H_16_N_2_O_2_*m*/*z*: 256.6 [M + H]^+^.

#### 3-Amino-*N*-benzyl-*N*-methylbenzamide (9c)

Compound 9c was prepared according to the general procedure A using 3-aminobenzoic acid (453 mg, 3.30 mmol) as the starting material. Yield: 43% (342 mg); brown oil; *R*_f_ (DCM : MeOH = 20 : 1) = 0.33; ^1^H NMR (400 MHz, DMSO-*d*_6_) *δ* 7.42–7.34 (m, 2H), 7.33–7.25 (m, 2H), 7.23–7.11 (m, 1H), 7.10–6.98 (m, 1H), 6.64–6.55 (m, 2H), 6.55–6.45 (m, 1H), 5.26 (s, 2H), 4.69–4.42 (m, 2H), 2.81 (s, 3H) ppm; MS (ESI+) C_15_H_16_N_2_O *m*/*z*: 240.8 [M + H]^+^.

#### 3-Amino-*N*-methyl-*N*-phenethylbenzamide (9d)

Compound 9c was prepared according to the general procedure A using 3-aminobenzoic acid (500 mg, 3.65 mmol) as the starting material. Yield: 90%; brown oil; *R*_f_ (EtOAc : hexane = 3 : 1) = 0.18; ^1^H NMR (400 MHz, DMSO-*d*_6_) *δ* 7.37–7.13 (m, 4H), 7.07–6.96 (m, 2H), 6.57 (ddd, *J*_1_ = 8.1 Hz, *J*_2_ = 2.4 Hz, *J*_3_ = 1.0 Hz, 1H), 6.50–6.24 (m, 2H), 5.20 (s, 2H), 3.66–3.57 (m, 1H), 3.01–2.75 (m, 5H) ppm, signal for the missing proton is covered with solvent; MS (ESI+) C_16_H_18_N_2_O *m*/*z*: 254.5 [M + H]^+^.

#### 3-Amino-*N*-methyl-*N*-(2-phenoxyethyl)benzamide (9e)

Compound 9c was prepared according to the general procedure A using 3-aminobenzoic acid (689 mg, 5.03 mmol) as the starting material. The product was additionally purified by column chromatography using DCM : MeOH = 30 : 1 as the eluent. Yield: 37% (503 mg); brown solid; *R*_f_ (DCM : MeOH = 30 : 1) = 0.32; ^1^H NMR (400 MHz, DMSO-*d*_6_) *δ* 7.34–7.22 (m, 2H), 7.04 (t, *J* = 7.8 Hz, 1H), 7.01–6.83 (m, 3H), 6.61–6.41 (m, 3H), 5.24 (s, 2H), 4.23–3.99 (m, 2H), 3.85–3.56 (m, 2H), 2.99 (s, 3H) ppm; MS (ESI+) C_16_H_18_N_2_O_2_*m*/*z*: 270.7 [M + H]^+^.

#### 4-Amino-*N*-phenethylbenzamide (9f)

Compound 9f was prepared according to the general procedure A using 4-aminobenzoic acid (896 mg, 6.54 mmol) as the starting material. The product was additionally purified by column chromatography using DCM : MeOH = 20 : 1 as the eluent. Yield: 43% (675 mg); white solid; *R*_f_ (DCM : MeOH = 20 : 1) = 0.31; ^1^H NMR (400 MHz, DMSO-*d*_6_) *δ* 8.09 (t, *J* = 5.6 Hz, 1H), 7.57–7.51 (m, 2H), 7.32–7.26 (m, 2H), 7.25–7.16 (m, 3H), 6.55–6.49 (m, 2H), 5.58 (s, 2H), 3.45–3.37 (m, 2H), 2.84–2.76 (m, 2H) ppm; MS (ESI+) C_15_H_16_N_2_O *m*/*z*: 240.7 [M + H]^+^.

#### 4-Amino-*N*-(2-phenoxyethyl)benzamide (9g)

Compound 9g was prepared according to the general procedure A using 4-aminobenzoic acid (761 mg, 5.55 mmol) as the starting material. Yield: 75% (1.06 g); light brown solid; *R*_f_ (DCM : MeOH = 9 : 1) = 0.61; ^1^H NMR (400 MHz, DMSO-*d*_6_) *δ* 8.21 (t, *J* = 5.5 Hz, 1H), 7.61–7.56 (m, 2H), 7.32–7.24 (m, 2H), 6.99–6.89 (m, 3H), 6.55–6.50 (m, 2H), 5.62 (s, 2H), 4.06 (t, *J* = 6.1 Hz, 2H), 3.56 (q, *J* = 5.9 Hz, 2H) ppm; ^13^C NMR (101 MHz, DMSO) *δ* 166.6, 158.5, 151.7, 129.5, 128.8, 121.0, 120.6, 114.5, 112.6, 66.1, 38.7 ppm; MS (ESI+) C_15_H_16_N_2_O_2_*m*/*z*: 256.6 [M + H]^+^.

#### 4-Amino-*N*-benzyl-*N*-methylbenzamide (9h)

Compound 9h was prepared according to the general procedure A using 4-aminobenzoic acid (679 mg, 4.95 mmol) as the starting material. Yield: 43% (750 mg); brown oil; *R*_f_ (DCM : MeOH = 9 : 1) = 0.53; ^1^H NMR (400 MHz, DMSO-*d*_6_) *δ* 7.39–7.34 (m, 2H), 7.31–7.21 (m, 3H), 7.21–7.16 (m, 2H), 6.57–6.52 (m, 2H), 5.51 (s, 2H), 4.60 (s, 2H), 2.87 (s, 3H) ppm; MS (ESI+) C_15_H_16_N_2_O *m*/*z*: 240.8 [M + H]^+^.

#### 4-Amino-*N*-methyl-*N*-phenethylbenzamide (9i)

Compound 9i was prepared according to the general procedure A using 4-aminobenzoic acid (500 mg, 3.65 mmol) as the starting material. Yield: 87%; brown solid; *R*_f_ (EtOAc : Hex = 3 : 1) = 0.24; ^1^H NMR (400 MHz, DMSO-*d*_6_) *δ* 7.32–7.25 (m, 2H), 7.24–7.10 (m, 3H), 6.99 (d, *J* = 7.9 Hz, 2H), 6.50 (d, *J* = 8.6 Hz, 2H), 5.44 (s, 2H), 3.52 (t, *J* = 6.7 Hz, 2H), 2.92 (s, 3H), 2.86–2.81 (m, 2H) ppm; MS (ESI+) C_16_H_18_N_2_O *m*/*z*: 254.5 [M + H]^+^.

#### 4-Amino-*N*-methyl-*N*-(2-phenoxyethyl)benzamide (9j)

Compound 9j was prepared according to general procedure A using 4-aminobenzoic acid (689 mg, 5.03 mmol) as the starting material. Yield: 66% (898 mg); brown oil; *R*_f_ (DCM : MeOH = 20 : 1) = 0.33; ^1^H NMR (400 MHz, DMSO-*d*_6_) *δ* 7.34–7.24 (m, 2H), 7.18–7.10 (m, 2H), 6.99–6.88 (m, 3H), 6.56–6.52 (m, 2H), 5.48 (s, 2H), 4.14 (t, *J* = 5.8 Hz, 2H), 3.72 (t, *J* = 5.8 Hz, 2H), 3.03 (s, 3H) ppm; MS (ESI+) C_16_H_18_N_2_O_2_*m*/*z*: 270.7 [M + H]^+^.

#### 3-((6-Chloropyrimidin-4-yl)amino)-*N*-phenethylbenzamide (10a)

Compound 10a was prepared according to the general procedure E using 9a (448 mg, 1.86 mmol) as the starting material. DCM : MeOH = 25 : 1 was used as the eluent for flash column chromatography. Yield: 34% (224 mg); white solid, *R*_f_ (DCM : MeOH = 20 : 1) = 0.28; ^1^H NMR (400 MHz, DMSO-*d*_6_) *δ* 10.01 (s, 1H), 8.58 (t, *J* = 5.5 Hz, 1H), 8.53–8.48 (m, 1H), 8.02 (t, *J* = 1.7 Hz, 1H), 7.87–7.79 (m, 1H), 7.51 (dt, *J*_1_ = 7.9 Hz, *J*_2_ = 1.4 Hz, 1H), 7.43 (t, *J* = 7.8 Hz, 1H), 7.39–7.13 (m, 5H), 6.82 (d, *J* = 0.9 Hz, 1H), 3.52–3.45 (m, 2H), 2.85 (t, *J* = 7.4 Hz, 2H) ppm; MS (ESI+) C_19_H_17_ClN_4_O *m*/*z*: 352.4 [M + H]^+^.

#### 3-((6-Chloropyrimidin-4-yl)amino)-*N*-(2-phenoxyethyl)benzamide (10b)

Compound 10b was prepared according to the general procedure E using 9b (1.15 g, 4.49 mmol) as the starting material. DCM : MeOH = 30 : 1 was used as the eluent for flash column chromatography. Yield: 48% (792 mg); brown solid; *R*_f_ (DCM : MeOH = 30 : 1) = 0.34; ^1^H NMR (400 MHz, DMSO-*d*_6_) *δ* 10.02 (s, 1H), 8.72 (t, *J* = 5.5 Hz, 1H), 8.50 (d, *J* = 0.6 Hz, 1H), 8.07–8.03 (m, 1H), 7.87–7.82 (m, 1H), 7.56 (dt, *J*_1_ = 7.9 Hz, *J*_2_ = 1.3 Hz, 1H), 7.44 (t, *J* = 7.9 Hz, 1H), 7.32–7.25 (m, 2H), 6.99–6.90 (m, 3H), 6.82 (d, *J* = 0.9 Hz, 1H), 4.11 (t, *J* = 5.9 Hz, 2H), 3.64 (q, *J* = 5.8 Hz, 2H) ppm; MS (ESI+) C_19_H_17_ClN_4_O_2_*m*/*z*: 368.5 [M + H]^+^.

#### *N*-Benzyl-3-((6-chloropyrimidin-4-yl)amino)-*N*-methylbenzamide (10c)

Compound 10c was prepared according to the general procedure E using 9c (311 mg, 1.29 mmol) as the starting material. DCM : MeOH = 30 : 1 was used as the eluent for the first flash column chromatography and EtOAc : hexane = 3 : 1 for the second one. Yield: 35% (160 mg); light brown oil; *R*_f_ (DCM : MeOH = 20 : 1) = 0.36; ^1^H NMR (400 MHz, DMSO-*d*_6_) *δ* 10.01 (s, 1H), 8.59–8.36 (m, 1H), 7.93–7.52 (m, 2H), 7.51–7.09 (m, 7H), 6.82 (s, 1H), 4.77–4.46 (m, 2H), 2.98–2.82 (m, 3H) ppm; MS (ESI+) C_19_H_17_ClN_4_O *m*/*z*: 352.6 [M + H]^+^.

#### 3-((6-Chloropyrimidin-4-yl)amino)-*N*-methyl-*N*-phenethylbenzamide (10d)

Compound 10d was prepared according to the general procedure E using 9d (8.35 g, 3.28 mmol) as the starting material. EtOAc : hexane = 3 : 1 was used as the eluent for flash column chromatography. Yield: 71%; light orange solid; *R*_f_ (EtOAc : Hex = 3 : 1) = 0.25; ^1^H NMR (400 MHz, DMSO-*d*_6_) *δ* 9.96 (d, *J* = 14.6 Hz, 1H), 8.49 (d, *J* = 12.8 Hz, 1H), 7.70–7.49 (m, 2H), 7.43–7.08 (m, 5H), 7.04–6.76 (m, 3H), 3.72–3.62 (m, 1H), 3.45–3.38 (m, 1H), 3.05–2.78 (m, 5H) ppm; MS (ESI+) C_20_H_19_ClN_4_O *m*/*z*: 366.5 [M + H]^+^.

#### 3-((6-Chloropyrimidin-4-yl)amino)-*N*-methyl-*N*-(2-phenoxyethyl)benzamide (10e)

Compound 10e was prepared according to the general procedure E using 9e (467 mg, 1.73 mmol) as the starting material. DCM : MeOH = 20 : 1 was used as the eluent for flash column chromatography. Yield: 53% (350 mg); brown solid; *R*_f_ (DCM : MeOH = 20 : 1) = 0.31; ^1^H NMR (400 MHz, DMSO-*d*_6_) *δ* 9.99 (s, 1H), 8.48 (d, *J* = 0.9 Hz, 1H), 7.85–7.69 (m, 1H), 7.68–7.52 (m, 1H), 7.41 (t, *J* = 7.8 Hz, 1H), 7.34–7.16 (m, 2H), 7.14–7.03 (m, 1H), 7.02–6.84 (m, 3H), 6.82 (s, 1H), 4.29–4.06 (m, 2H), 3.88–3.58 (m, 2H), 3.03 (s, 3H) ppm; MS (ESI+) C_20_H_19_ClN_4_O_2_*m*/*z*: 382.5 [M + H]^+^.

#### 4-((6-Chloropyrimidin-4-yl)amino)-*N*-phenethylbenzamide (10f)

Compound 10f was prepared according to the general procedure E using 9f (651 mg, 2.71 mmol) as the starting material. DCM : MeOH = 25 : 1 was used as the eluent for flash column chromatography. Yield: 23% (220 mg); light yellow solid; *R*_f_ (DCM : MeOH = 20 : 1) = 0.27; ^1^H NMR (400 MHz, DMSO-*d*_6_) *δ* 10.10 (s, 1H), 8.55 (s, 1H), 8.48 (t, *J* = 5.5 Hz, 1H), 7.86–7.79 (m, 2H), 7.75–7.69 (m, 2H), 7.35–7.18 (m, 5H), 6.88 (d, *J* = 1.0 Hz, 1H), 3.51–3.44 (m, 2H), 2.88–2.79 (m, 2H) ppm; MS (ESI+) C_19_H_17_ClN_4_O *m*/*z*: 352.4 [M + H]^+^.

#### 4-((6-Chloropyrimidin-4-yl)amino)-*N*-(2-phenoxyethyl)benzamide (10g)

Compound 10g was prepared according to the general procedure E using 9g (1.00 g, 3.90 mmol) as the starting material. DCM : MeOH = 30 : 1 was used as the eluent for flash column chromatography. Yield: 35% (504 mg); light brown solid; *R*_f_ (DCM : MeOH = 20 : 1) = 0.31; ^1^H NMR (400 MHz, DMSO-*d*_6_) *δ* 10.11 (s, 1H), 8.62 (t, *J* = 5.5 Hz, 1H), 8.56 (d, *J* = 0.9 Hz, 1H), 7.91–7.83 (m, 2H), 7.77–7.70 (m, 2H), 7.33–7.25 (m, 2H), 7.00–6.90 (m, 3H), 6.88 (d, *J* = 0.8 Hz, 1H), 4.11 (t, *J* = 5.9 Hz, 2H), 3.63 (q, *J* = 5.8 Hz, 2H) ppm; ^13^C NMR (101 MHz, DMSO-*d*_6_) *δ* 166.00, 160.97, 158.49, 158.44, 158.14, 141.78, 129.52, 128.43, 128.24, 120.64, 119.10, 114.47, 105.83, 65.93 ppm; MS (ESI+) C_19_H_17_ClN_4_O_2_*m*/*z*: 368.5 [M + H]^+^.

#### *N*-Benzyl-4-((6-chloropyrimidin-4-yl)amino)-*N*-methylbenzamide (10h)

Compound 10h was prepared according to the general procedure E using 9h (688 mg, 2.86 mmol) as the starting material. DCM : MeOH = 30 : 1 was used as the eluent for flash column chromatography. Yield: 35% (354 mg); light brown solid; *R*_f_ (DCM : MeOH = 30 : 1) = 0.25; ^1^H NMR (400 MHz, DMSO-*d*_6_) *δ* 10.06 (s, 1H), 8.52 (s, 1H), 7.78–7.66 (m, 2H), 7.54–7.42 (m, 2H), 7.41–7.15 (m, 5H), 6.86 (s, 1H), 4.69–4.51 (m, 2H), 2.88 (s, 3H) ppm; MS (ESI+) C_19_H_17_ClN_4_O *m*/*z*: 352.6 [M + H]^+^.

#### 4-((6-Chloropyrimidin-4-yl)amino)-*N*-methyl-*N*-phenethylbenzamide (10i)

Compound 10i was prepared according to the general procedure E using 9i (787 mg, 3.09 mmol) as the starting material. EtOAc : hexane = 3 : 1 was used as the eluent for flash column chromatography. Yield: 39%; light brown solid; *R*_f_ (EtOAc : Hex = 3 : 1) = 0.21; ^1^H NMR (400 MHz, DMSO-*d*_6_) *δ* 10.03 (s, 1H), 8.53 (d, *J* = 0.8 Hz, 1H), 7.66 (s, 2H), 7.38–6.98 (m, 7H), 6.85 (s, 1H), 3.72–3.40 (m, 2H), 3.05–2.77 (m, 5H) ppm; MS (ESI+) C_20_H_19_ClN_4_O *m*/*z*: 366.5 [M + H]^+^.

#### 4-((6-Chloropyrimidin-4-yl)amino)-*N*-methyl-*N*-(2-phenoxyethyl)benzamide (10j)

Compound 10j was prepared according to the general procedure E using 9j (897 mg, 3.32 mmol) as the starting material. DCM : MeOH = 30 : 1 was used as the eluent for flash column chromatography. Yield: 46% (584 mg); light brown solid; *R*_f_ (DCM : MeOH = 20 : 1) = 0.36; ^1^H NMR (400 MHz, DMSO-*d*_6_) *δ* 10.05 (s, 1H), 8.53 (s, 1H), 7.74–7.67 (m, 2H), 7.46–7.38 (m, 2H), 7.33–7.24 (m, 2H), 7.03–6.87 (m, 3H), 6.86 (d, *J* = 0.8 Hz, 1H), 4.29–4.00 (m, 2H), 3.89–3.57 (m, 2H), 3.04 (s, 3H) ppm; MS (ESI+) C_20_H_19_ClN_4_O_2_*m*/*z*: 382.5 [M + H]^+^.

#### *N*-Phenethyl-3-((6-(pyrrolidin-1-yl)pyrimidin-4-yl)amino)benzamide (11a)

Compound 11a was prepared according to the general procedure C using 10a (45 mg, 0.142 mmol) as the starting material. Yield: 31% (18 mg); white solid; *R*_f_ (DCM : MeOH = 9 : 1) = 0.47; ^1^H NMR (400 MHz, DMSO-*d*_6_) *δ* 9.15 (s, 1H), 8.49 (t, *J* = 5.5 Hz, 1H), 8.17 (s, 1H), 8.00 (s, 1H), 7.78 (dt, *J*_1_ = 5.1 Hz, *J*_2_ = 2.4 Hz, 1H), 7.37–7.17 (m, 7H), 5.69 (s, 1H), 3.53–3.43 (m, 2H), 2.85 (t, *J* = 7.5 Hz, 2H), 1.93 (s, 4H) ppm; signals for the remaining 4 protons are overlapped with solvent; ^13^C NMR (101 MHz, DMSO-*d*_6_) *δ* 166.4, 160.1, 157.4, 141.0, 139.5, 135.3, 128.7, 128.5, 128.3, 126.1, 121.7, 119.5, 118.4, 84.0, 45.9, 40.9, 35.1, 24.8 ppm; HRMS (ESI+) calcd for C_23_H_26_N_5_O *m*/*z* [M + H]^+^: 388.21319, found: 388.21322; UHPLC: *t*_r_: 3.83 min (96.1% at 254 nm).

#### *N*-(2-Phenoxyethyl)-3-((6-(pyrrolidin-1-yl)pyrimidin-4-yl)amino)benzamide (11b)

Compound 11b was prepared according to the general procedure C using 10b (100 mg, 0.271 mmol) as the starting material. DCM : MeOH = 15 : 1 was used as the eluent for flash column chromatography. Yield: 82% (90 mg); white solid; *R*_f_ (DCM : MeOH = 9 : 1) = 0.53; ^1^H NMR (400 MHz, DMSO-*d*_6_) *δ* 9.16 (s, 1H), 8.62 (t, *J* = 5.5 Hz, 1H), 8.16 (d, *J* = 0.7 Hz, 1H), 8.03 (t, *J* = 1.9 Hz, 1H), 7.82–7.77 (m, 1H), 7.42–7.37 (m, 1H), 7.37–7.32 (m, 1H), 7.32–7.26 (m, 2H), 7.00–6.90 (m, 3H), 5.70–5.67 (m, *J* = 0.6 Hz, 1H), 4.11 (t, *J* = 5.9 Hz, 2H), 3.62 (q, *J* = 5.8 Hz, 2H), 1.96–1.87 (m, 4H) ppm; signals for the remaining 4 protons are overlapped with solvent; ^13^C NMR (101 MHz, DMSO-*d*_6_) *δ* 166.8, 160.0, 158.4, 157.3, 141.1, 135.0, 129.5, 128.5, 121.9, 120.6, 119.6, 118.4, 114.5, 84.0, 65.8, 45.9, 24.8 ppm; HRMS (ESI+) calcd for C_23_H_26_N_5_O_2_*m*/*z* [M + H]^+^: 404.20810, found: 404.20815; UHPLC: *t*_r_: 3.83 min (99.4% at 254 nm).

#### *N*-Benzyl-*N*-methyl-3-((6-(pyrrolidin-1-yl)pyrimidin-4-yl)amino)benzamide (11c)

Compound 11c was prepared according to the general procedure C using 10c (47 mg, 0.133 mmol) as the starting material. DCM : MeOH = 20 : 1 was used as the eluent for flash column chromatography. Yield: 97% (50 mg); brown solid; *R*_f_ (DCM : MeOH = 9 : 1) = 0.15; ^1^H NMR (400 MHz, DMSO-*d*_6_) *δ* 9.17 (s, 1H), 8.22–8.07 (m, 1H), 7.93–7.70 (m, 1H), 7.70–7.48 (m, 1H), 7.44–7.18 (m, 6H), 7.01–6.90 (m, 1H), 5.67 (s, 1H), 4.72–4.47 (m, 2H), 2.85 (s, 3H), 1.92 (s, 4H) ppm, signals for the remaining 4 protons are overlapped with solvent; ^13^C NMR (101 MHz, DMSO-*d*_6_) *δ* 160.0, 159.9, 157.2, 141.2, 137.5, 136.9, 136.7, 128.7, 127.6, 127.3, 126.9, 119.7, 118.8, 117.1, 84.4, 59.8, 54.1, 49.8, 45.9, 36.9, 32.4, 24.8, 20.8, 14.1 ppm, rotamers are present in the spectra; HRMS (ESI+) calcd for C_23_H_26_N_5_O *m*/*z* [M + H]^+^: 388.21319, found: 388.21313; UHPLC: *t*_r_: 3.82 min (97.7% at 254 nm).

#### *N*-Methyl-*N*-phenethyl-3-((6-(pyrrolidin-1-yl)pyrimidin-4-yl)amino)benzamide (11d)

Compound 11d was prepared according to the general procedure C using 10d (101 mg, 0.275 mmol) as the starting material. DCM : MeOH = 20 : 1 was used as the eluent for flash column chromatography. Yield: 84%; white solid; *R*_f_ (DCM : MeOH = 20 : 1) = 0.10; ^1^H NMR (400 MHz, DMSO-*d*_6_) *δ* 9.14 (s, 1H), 8.16 (d, *J* = 8.8 Hz, 1H), 7.68 (t, *J* = 1.9 Hz, 1H), 7.60–7.47 (m, 1H), 7.34–7.11 (m, 5H), 7.01 (d, *J* = 7.3 Hz, 1H), 6.84–6.62 (m, 1H), 5.67 (s, 1H), 3.66 (t, *J* = 7.7 Hz, 1H), 3.47–3.35 (m, 5H), 3.04–2.80 (m, 5H), 1.92 (s, 4H) ppm; ^13^C NMR (101 MHz, DMSO-*d*_6_) 170.7, 170.0, 160.0, 160.0, 157.3, 140.9, 139.2, 138.4, 137.2, 128.8, 128.6, 128.4, 126.3, 119.6, 119.4, 119.1, 118.7, 117.2, 116.6, 84.2, 52.3, 48.3, 45.9, 37.4, 34.0, 32.7, 32.4, 24.8 ppm, rotamers are present in the spectrum; HRMS (ESI+) calcd for C_24_H_27_N_5_O 402.7 [M + H]^+^: 402.2288, found: 402.2280; UPLC: *t*_r_: 3.84 min (99.5% at 254 nm).

#### *N*-Methyl-*N*-(2-phenoxyethyl)-3-((6-(pyrrolidin-1-yl)pyrimidin-4-yl)amino)benzamide (11e)

Compound 11e was prepared according to the general procedure C using 10e (70 mg, 0.183 mmol) as the starting material. DCM : MeOH = 20 : 1 was used as the eluent for flash column chromatography. Yield: 75% (57 mg); white solid; *R*_f_ (DCM : MeOH = 9 : 1) = 0.51; ^1^H NMR (400 MHz, DMSO-*d*_6_) *δ* 9.16 (s, 1H), 8.15 (s, 1H), 7.87–7.67 (m, 1H), 7.64–7.48 (m, 1H), 7.35–7.19 (m, 3H), 7.04–6.82 (m, 4H), 5.67 (s, 1H), 4.27–4.03 (m, 2H), 3.86–3.57 (m, 2H), 3.03 (s, 3H), 2.00–1.83 (m, 4H) ppm; signals for the remaining 4 protons are covered with solvent; ^13^C NMR (101 MHz, DMSO-*d*_6_) *δ* 171.3, 170.4, 160.0, 158.4, 158.0, 157.3, 141.1, 137.2, 136.9, 129.5, 129.5, 128.6, 120.7, 119.7, 119.4, 119.2, 117.2, 114.5, 114.3, 84.2, 65.2, 64.5, 49.6, 46.4, 45.9, 38.4, 32.6, 24.8 ppm, rotamers are present in the spectra; HRMS (ESI+) calcd for C_24_H_28_N_5_O_2_*m*/*z* [M + H]^+^: 418.22375, found: 418.22363; UHPLC: *t*_r_: 3.87 min (99.0% at 254 nm).

#### *N*-Phenethyl-4-((6-(pyrrolidin-1-yl)pyrimidin-4-yl)amino)benzamide (11f)

Compound 11f was prepared according to general procedure C using 10f (45 mg, 0.142 mmol) as the starting material. Yield: 73% (40 mg); light orange solid; *R*_f_ (DCM : MeOH = 9 : 1) = 0.44; ^1^H NMR (400 MHz, DMSO-*d*_6_) *δ* 9.29 (s, 1H), 8.37 (t, *J* = 5.6 Hz, 1H), 8.20 (s, 1H), 7.77–7.71 (m, 2H), 7.70–7.65 (m, 2H), 7.32–7.27 (m, 2H), 7.26–7.17 (m, 3H), 5.75–5.72 (m, 1H), 3.50–3.43 (m, 2H), 3.42–3.33 (m, 4H), 2.83 (t, *J* = 7.5 Hz, 2H), 1.98–1.87 (m, 4H) ppm; ^13^C NMR (101 MHz, DMSO-*d*_6_) *δ* 165.8, 160.0, 159.8, 157.3, 143.8, 139.7, 128.7, 128.3, 128.0, 126.5, 126.1, 117.7, 84.8, 45.9, 40.8, 35.3, 24.8 ppm; HRMS (ESI+) calcd for C_23_H_26_N_5_O *m*/*z* [M + H]^+^: 388.21319, found 388.21314; UHPLC: *t*_r_: 3.77 min (92.7% at 254 nm).

#### *N*-(2-Phenoxyethyl)-4-((6-(pyrrolidin-1-yl)pyrimidin-4-yl)amino)benzamide (11g)

Compound 11g was prepared according to the general procedure C using 10g (70 mg, 0.198 mmol) as the starting material. DCM : MeOH = 20 : 1 was used as the eluent for flash column chromatography. Yield: 63% (50 mg); white solid; *R*_f_ (DCM : MeOH = 20 : 1) = 0.15; ^1^H NMR (400 MHz, DMSO-*d*_6_) *δ* 9.31 (s, 1H), 8.51 (t, *J* = 5.5 Hz, 1H), 8.20 (d, *J* = 0.8 Hz, 1H), 7.82–7.75 (m, 2H), 7.72–7.66 (m, 2H), 7.32–7.25 (m, 2H), 6.99–6.90 (m, 3H), 5.73 (d, *J* = 0.8 Hz, 1H), 4.09 (t, *J* = 6.0 Hz, 2H), 3.65–3.57 (m, 2H), 3.44–3.34 (m, 4H), 1.98–1.88 (m, 4H) ppm; ^13^C NMR (101 MHz, DMSO-*d*_6_) *δ* 166.2, 160.0, 159.8, 158.4, 157.3, 143.9, 129.5, 128.1, 126.2, 120.6, 117.7, 114.5, 84.8, 66.0, 45.9, 38.8, 24.8 ppm; HRMS (ESI+) calcd for C_23_H_26_N_5_O_2_*m*/*z* [M + H]^+^: 404.20810, found: 404.20794; UHPLC: *t*_r_: 3.78 min (99.4% at 254 nm).

#### *N*-Benzyl-*N*-methyl-4-((6-(pyrrolidin-1-yl)pyrimidin-4-yl)amino)benzamide (11h)

Compound 11h was prepared according to the general procedure C using 10h (70 mg, 0.198 mmol) as the starting material. DCM : MeOH = 20 : 1 was used as the eluent for flash column chromatography. Yield: 72% (55 mg); light brown solid; *R*_f_ (DCM : MeOH = 20 : 1) = 0.23; ^1^H NMR (400 MHz, DMSO-*d*_6_) *δ* 9.25 (s, 1H), 8.17 (s, 1H), 7.68 (d, *J* = 8.2 Hz, 2H), 7.42–7.35 (m, 4H), 7.34–7.18 (m, 3H), 5.71 (s, 1H), 4.61 (s, 2H), 3.44–3.34 (m, 4H), 2.88 (s, 3H), 1.99–1.86 (m, 4H) ppm; ^13^C NMR (101 MHz, DMSO-*d*_6_) *δ* 170.9, 160.0, 159.8, 157.3, 142.4, 137.4, 128.7, 128.1, 128.0, 127.2, 118.0, 84.6, 54.2, 52.7, 50.2, 47.2, 45.9, 36.9, 32.9, 24.8 ppm, rotamers are present in the spectrum; HRMS (ESI+) calcd for C_23_H_26_N_5_O *m*/*z* [M + H]^+^: 388.21319, found: 388.21323; UHPLC: *t*_r_: 3.81 min (98.7% at 254 nm).

#### *N*-Methyl-*N*-phenethyl-4-((6-(pyrrolidin-1-yl)pyrimidin-4-yl)amino)benzamide (11i)

Compound 11i was prepared according to the general procedure C using 10i (100 mg, 0.272 mmol) as the starting material. DCM : MeOH = 20 : 1 was used as the eluent for flash column chromatography. Yield: 46%; white solid; *R*_f_ (DCM : MeOH = 20 : 1) = 0.13; ^1^H NMR (400 MHz, DMSO-*d*_6_) *δ* 9.21 (s, 1H), 8.18 (s, 1H), 7.63 (d, *J* = 7.8 Hz, 2H), 7.41–6.97 (m, 7H), 5.71 (s, 1H), 3.70–3.36 (m, 6H), 3.04–2.81 (m, 5H), 2.03–1.85 (m, 4H) ppm; ^13^C NMR (101 MHz, DMSO-*d*_6_) *δ* 170.8, 160.0, 159.9, 157.3, 142.0, 139.1, 128.7, 128.4, 127.8, 127.4, 126.2, 118.7, 117.9, 84.4, 45.9, 37.6, 33.6, 32.6, 32.4, 24.8 ppm, rotamers are present in the spectra and some signals were determined using a HSQC spectrum; HRMS (ESI+) calcd for C_24_H_27_N_5_O [M + H]^+^: 402.2288, found: 402.2278; UHPLC: *t*_r_: 3.88 min (99.8% at 254 nm).

#### *N*-Methyl-*N*-(2-phenoxyethyl)-4-((6-(pyrrolidin-1-yl)pyrimidin-4-yl)amino)benzamide (11j)

Compound 11j was prepared according to the general procedure C using 10j (100 mg, 0.261 mmol) as the starting material. DCM : MeOH = 20 : 1 was used as the eluent for flash column chromatography. Yield: 72% (78 mg); white solid; *R*_f_ (DCM : MeOH = 9 : 1) = 0.54; ^1^H NMR (400 MHz, DMSO-*d*_6_) *δ* 9.23 (s, 1H), 8.18 (s, 1H), 7.71–7.64 (m, 2H), 7.40–7.20 (m, 4H), 7.02–6.83 (m, 3H), 5.71 (d, *J* = 1.1 Hz, 1H), 4.30–3.99 (m, 2H), 3.87–3.63 (m, 2H), 3.47–3.33 (m, 4H), 3.04 (s, 3H), 2.00–1.83 (m, 4H) ppm; ^13^C NMR (101 MHz, DMSO-*d*_6_) *δ* 171.4, 160.0, 159.9, 158.2, 157.3, 142.2, 129.5, 128.0, 120.7, 118.0, 114.4, 84.5, 64.9, 49.8, 45.9, 32.9, 27.8, 24.8 ppm; HRMS (ESI+) calcd for C_24_H_28_N_5_O_2_*m*/*z* [M + H]^+^: 418.22375, found: 418.22331; UHPLC: *t*_r_: 3.90 min (98.2% at 254 nm).

#### *tert*-Butyl 4-(6-((3-(phenethylcarbamoyl)phenyl)amino)pyrimidin-4-yl)piperazine-1-carboxylate (12a)

Compound 12a was prepared according to the general procedure C using 10a (221 mg, 0.626 mmol) as the starting material. DCM : MeOH = 20 : 1 was used as the eluent for flash column chromatography. Yield: 36% (113 mg); white solid, *R*_f_ (DCM : MeOH = 9 : 1) = 0.46; ^1^H NMR (400 MHz, DMSO-*d*_6_) *δ* 9.27 ^1^H NMR (400 MHz, DMSO-*d*_6_) *δ* 9.27 (s, 1H), 8.50 (t, *J* = 5.6 Hz, 1H), 8.23 (s, 1H), 8.01–7.95 (m, 1H), 7.78 (dt, *J*_1_ = 7.0 Hz, *J*_2_ = 2.1 Hz, 1H), 7.39–7.16 (m, 7H), 5.97 (s, 1H), 3.54–3.37 (m, 10H), 2.84 (t, *J* = 7.4 Hz, 2H), 1.42 (s, 9H) ppm; MS (ESI+) C_28_H_34_N_6_O_3_*m*/*z*: 502.2 [M + H]^+^.

#### *tert*-Butyl 4-(6-((3-((2-phenoxyethyl)carbamoyl)phenyl)amino)pyrimidin-4-yl)piperazine-1-carboxylate (12b)

Compound 12b was prepared according to the general procedure C using 10b (336 mg, 0.911 mmol) as the starting material. DCM : MeOH = 20 : 1 was used as the eluent for flash column chromatography. Yield: 59% (252 mg); light brown solid; *R*_f_ (DCM : MeOH = 20 : 1) = 0.47; ^1^H NMR (400 MHz, DMSO-*d*_6_) *δ* 9.28 (s, 1H), 8.64 (t, *J* = 5.5 Hz, 1H), 8.22 (s, 1H), 8.02 (t, *J* = 2.0 Hz, 1H), 7.80 (ddd, *J*_1_ = 8.1 Hz, *J*_2_ = 2.3 Hz, *J*_3_ = 1.2 Hz, 1H), 7.42 (dt, *J*_1_ = 7.8 Hz, *J*_2_ = 1.4 Hz 1H), 7.35 (t, *J* = 7.8 Hz, 1H), 7.32–7.25 (m, 2H), 6.99–6.90 (m, 3H), 5.97 (s, 1H), 4.11 (t, *J* = 5.9 Hz, 2H), 3.62 (q, *J* = 5.8 Hz, 2H), 3.54–3.46 (m, 4H), 3.46–3.38 (m, 4H), 1.42 (s, 9H) ppm; MS (ESI+) C_28_H_34_N_6_O_4_*m*/*z*: 518.3 [M + H]^+^.

#### *tert*-Butyl 4-(6-((3-(benzyl(methyl)carbamoyl)phenyl)amino)pyrimidin-4-yl)piperazine-1-carboxylate (12c)

Compound 12c was prepared according to the general procedure C using 10c (87 mg, 0.247 mmol) as the starting material. DCM : MeOH = 20 : 1 was used as the eluent for flash column chromatography. Yield: 30% (37 mg); light brown solid; *R*_f_ (DCM : MeOH = 20 : 1) = 0.53; ^1^H NMR (400 MHz, DMSO-*d*_6_) *δ* 9.28 (s, 1H), 8.25–8.13 (m, 1H), 7.92–7.71 (m, 1H), 7.70–7.49 (m, 1H), 7.42–7.27 (m, 5H), 7.26–7.17 (m, 1H), 7.02–6.93 (m, 1H), 5.98–5.91 (m, 1H), 4.72–4.48 (m, 2H), 3.53–3.46 (m, 4H), 3.44–3.39 (m, 4H), 2.85 (s, 3H), 1.41 (s, 9H) ppm; MS (ESI+) C_28_H_34_N_6_O_3_*m*/*z*: 502.3 [M + H]^+^.

#### *tert*-Butyl 4-(6-((3-(methyl(phenethyl)carbamoyl)phenyl)amino)pyrimidin-4-yl)piperazine-1-carboxylate (12d)

Compound 12d was prepared according to the general procedure C using 10d (150 mg, 0.409 mmol) as the starting material. DCM : MeOH = 20 : 1 was used as the eluent for flash column chromatography. Yield: 68%; white solid; *R*_f_ (DCM : MeOH = 20 : 1) = 0.20; ^1^H NMR (400 MHz, DMSO-*d*_6_) *δ* 9.24 (s, 1H), 8.22 (d, *J* = 9.1 Hz, 1H), 7.70–7.64 (m, 1H), 7.60–7.49 (m, 1H), 7.36–7.10 (m, 5H), 7.01 (d, *J* = 7.3 Hz, 1H), 6.86–6.65 (m, 1H), 5.95 (s, 1H), 3.70–3.62 (m, 1H), 3.54–3.46 (m, 4H), 3.45–3.37 (m, 5H), 3.00 (s, 2H), 2.93–2.79 (m, 3H), 1.42 (s, 9H) ppm; MS (ESI+) C_29_H_36_N_6_O_3_*m*/*z*: 517.6 [M + H]^+^.

#### *tert*-Butyl 4-(6-((3-(methyl(2-phenoxyethyl)carbamoyl)phenyl)amino)pyrimidin-4-yl)piperazine-1-carboxylate (12e)

Compound 12e was prepared according to the general procedure C using 10e (180 mg, 0.470 mmol) as the starting material. DCM : MeOH = 20 : 1 was used as the eluent for flash column chromatography. Yield: 52% (130 mg); light yellow solid; *R*_f_ (DCM : MeOH = 9 : 1) = 0.35; ^1^H NMR (400 MHz, DMSO-*d*_6_) *δ* 9.27 (s, 1H), 8.21 (s, 1H), 7.85–7.69 (m, 1H), 7.63–7.50 (m, 1H), 7.34–7.20 (m, 3H), 7.03–6.84 (m, 4H), 5.95 (s, 1H), 4.26–4.18 (m, 1H), 4.12–4.03 (m, 1H), 3.86–3.78 (m, 1H), 3.68–3.60 (m, 1H), 3.53–3.45 (m, 4H), 3.45–3.37 (m, *J* = 7.1 Hz, 4H), 3.03 (s, 3H), 1.42 (s, 9H) ppm; MS (ESI+) C_29_H_36_N_6_O_4_*m*/*z*: 532.3 [M + H]^+^.

#### *tert*-Butyl 4-(6-((4-(phenethylcarbamoyl)phenyl)amino)pyrimidin-4-yl)piperazine-1-carboxylate (12f)

Compound 12f was prepared according to the general procedure C using 10f (188 mg, 0.533 mmol) as the starting material. DCM : MeOH = 20 : 1 was used as the eluent for flash column chromatography. Yield: 62%; light brown solid; *R*_f_ (DCM : MeOH = 9 : 1) = 0.54; ^1^H NMR (400 MHz, DMSO-*d*_6_) *δ* 9.40 (s, 1H), 8.39 (t, *J* = 5.6 Hz, 1H), 8.26 (s, 1H), 7.80–7.72 (m, 2H), 7.71–7.64 (m, 2H), 7.34–7.27 (m, 2H), 7.27–7.16 (m, 3H), 6.02 (s, 1H), 3.55–3.49 (m, 4H), 3.51–3.39 (m, 6H), 2.83 (t, *J* = 7.5 Hz, 2H), 1.43 (s, 9H) ppm; MS (ESI+) C_28_H_34_N_6_O_3_*m*/*z*: 502.2 [M + H]^+^.

#### *tert*-Butyl 4-(6-((4-((2-phenoxyethyl)carbamoyl)phenyl)amino)pyrimidin-4-yl)piperazine-1-carboxylate (12g)

Compound 12g was prepared according to the general procedure C using 10g (219 mg, 0.594 mmol) as the starting material. DCM : MeOH = 25 : 1 was used as the eluent for flash column chromatography. Yield: 50% (154 mg); light yellow solid; *R*_f_ (DCM : MeOH = 9 : 1) = 0.56; ^1^H NMR (400 MHz, DMSO-*d*_6_) *δ* 9.41 (s, 1H), 8.52 (t, *J* = 5.7 Hz, 1H), 8.26 (s, 1H), 7.83–7.76 (m, 2H), 7.72–7.66 (m, 2H), 7.33–7.25 (m, 2H), 7.00–6.90 (m, 3H), 6.04–6.00 (m, 1H), 4.10 (t, *J* = 6.0 Hz, 2H), 3.61 (q, *J* = 5.9 Hz, 2H), 3.54–3.48 (m, 4H), 3.46–3.38 (m, 4H), 1.42 (s, 9H) ppm; ^13^C NMR (101 MHz, DMSO-*d*_6_) *δ* 166.15, 162.01, 160.66, 158.45, 157.35, 153.91, 143.61, 129.53, 128.11, 126.55, 120.62, 117.92, 114.46, 85.20, 79.12, 65.96, 43.21, 38.82, 28.07 ppm; MS (ESI+) C_28_H_34_N_6_O_4_*m*/*z*: 518.3 [M + H]^+^.

#### *tert*-Butyl 4-(6-((4-(benzyl(methyl)carbamoyl)phenyl)amino)pyrimidin-4-yl)piperazine-1-carboxylate (12h)

Compound 12h was prepared according to the general procedure C using 10h (247 mg, 0.700 mmol) as the starting material. DCM : MeOH = 20 : 1 was used as the eluent for flash column chromatography. Yield: 49% (172 mg); light brown solid; *R*_f_ (DCM : MeOH = 20 : 1) = 0.47; ^1^H NMR (400 MHz, DMSO-*d*_6_) *δ* 9.36 (s, 1H), 8.24 (s, 1H), 7.67 (d, *J* = 8.2 Hz, 2H), 7.48–7.16 (m, 7H), 5.99 (s, 1H), 4.61 (s, 2H), 3.57–3.46 (m, 4H), 3.47–3.37 (m, 4H), 2.88 (s, 3H), 1.42 (s, 9H) ppm; MS (ESI+) C_28_H_34_N_6_O_3_*m*/*z*: 502.2 [M + H]^+^.

#### *tert*-Butyl 4-(6-((4-(methyl(phenethyl)carbamoyl)phenyl)amino)pyrimidin-4-yl)piperazine-1-carboxylate (12i)

Compound 12i was prepared according to the general procedure C using 10i (150 mg, 0.408 mmol) as the starting material. DCM : MeOH = 20 : 1 was used as the eluent for flash column chromatography. Yield: 38%; white solid; *R*_f_ (DCM : MeOH = 20 : 1) = 0.18; ^1^H NMR (400 MHz, DMSO-*d*_6_) *δ* 9.32 (s, 1H), 8.24 (s, 1H), 7.62 (d, *J* = 6.0 Hz, 2H), 7.39–7.03 (m, 7H), 5.99 (s, 1H), 3.67–3.39 (m, 10H), 3.00–2.80 (m, 5H), 1.43 (s, 9H) ppm; MS (ESI+) C_29_H_36_N_6_O_3_*m*/*z*: 517.9 [M + H]^+^.

#### *tert*-Butyl 4-(6-((4-(methyl(2-phenoxyethyl)carbamoyl)phenyl)amino)pyrimidin-4-yl)piperazine-1-carboxylate (12j)

Compound 12j was prepared according to the general procedure C using 10j (248 mg, 0.678 mmol) as the starting material. DCM : MeOH = 20 : 1 was used as the eluent for flash column chromatography. Yield: 32% (110 mg); white solid; *R*_f_ (DCM : MeOH = 9 : 1) = 0.44; ^1^H NMR (400 MHz, DMSO-*d*_6_) *δ* 9.34 (s, 1H), 8.24 (s, 1H), 7.70–7.62 (m, 2H), 7.41–7.32 (m, 2H), 7.29 (t, *J* = 7.3 Hz, 2H), 7.07–6.84 (m, 3H), 6.00 (s, 1H), 4.23–4.07 (m, 2H), 3.82–3.68 (m, 2H), 3.56–3.47 (m, 4H), 3.47–3.40 (m, 4H), 3.04 (s, 3H), 1.42 (s, 9H) ppm; MS (ESI+) C_29_H_36_N_6_O_4_*m*/*z*: 532.1 [M + H]^+^

#### *N*-Phenethyl-3-((6-(piperazin-1-yl)pyrimidin-4-yl)amino)benzamide (13a)

Compound 13a was prepared according to the general procedure D using 12a (77 mg, 0.153 mmol) as the starting material. DCM : MeOH : NH_4_OH = 9 : 1 : 0.1 was used as the eluent for flash column chromatography. Yield: 83% (51 mg); white solid; *R*_f_ (DCM : MeOH : NH_4_OH = 9 : 1 : 0.1) = 0.11; ^1^H NMR (400 MHz, DMSO-*d*_6_) *δ* 9.19 (s, 1H), 8.49 (t, *J* = 5.6 Hz, 1H), 8.20 (s, 1H), 7.97 (s, 1H), 7.84–7.74 (m, 1H), 7.41–7.15 (m, 7H), 5.94 (s, 1H), 3.53–3.37 (m, 6H), 2.84 (t, *J* = 7.4 Hz, 2H), 2.79–2.72 (m, 4H) ppm; ^13^C NMR (101 MHz, DMSO-*d*_6_) *δ* 166.3, 162.3, 160.9, 157.3, 140.9, 139.6, 135.4, 128.7, 128.5, 128.4, 126.1, 121.8, 119.7, 118.5, 84.3, 45.0, 44.4, 40.9, 35.1 ppm; HRMS (ESI+) calcd for C_23_H_27_N_6_O *m*/*z* [M + H]^+^: 403.22409, found: 403.22379; UHPLC: *t*_r_: 2.80 min (99.3% at 254 nm).

#### *N*-(2-Phenoxyethyl)-3-((6-(piperazin-1-yl)pyrimidin-4-yl)amino)benzamide (13b)

Compound 13b was prepared according to the general procedure D using 12b (92 mg, 0.177 mmol) as the starting material. DCM : MeOH : NH_4_OH = 9 : 1 : 0.1 was used as the eluent for flash column chromatography. Yield: 63% (47 mg); white solid; *R*_f_ (DCM : MeOH : NH_4_OH = 9 : 1 : 0.1) = 0.08; ^1^H NMR (400 MHz, DMSO-*d*_6_) *δ* 9.20 (s, 1H), 8.63 (t, *J* = 5.5 Hz, 1H), 8.20 (s, 1H), 8.01 (t, *J* = 2.0 Hz, 1H), 7.82–7.77 (m, 1H), 7.41 (dt, *J*_1_ = 7.8 Hz, *J*_2_ = 1.4 Hz, 1H), 7.38–7.24 (m, 3H), 7.00–6.89 (m, 3H), 5.93 (s, 1H), 4.11 (t, *J* = 5.9 Hz, 2H), 3.62 (q, *J* = 5.9 Hz, 2H), 3.47–3.37 (m, 4H), 2.88–2.70 (m, 5H) ppm; ^13^C NMR (101 MHz, DMSO-*d*_6_) *δ* 166.7, 162.3, 160.9, 158.4, 157.3, 140.9, 135.0, 129.5, 128.5, 122.0, 120.6, 119.8, 118.5, 114.5, 84.3, 65.8, 45.2, 44.7 ppm; HRMS (ESI+) calcd for C_23_H_27_N_6_O_2_*m*/*z* [M + H]^+^: 419.21900, found: 419.21860; UHPLC: *t*_r_: 2.80 min (99.6% at 254 nm).

#### *N*-Benzyl-*N*-methyl-3-((6-(piperazin-1-yl)pyrimidin-4-yl)amino)benzamide (13c)

Compound 13c was prepared according to the general procedure D using 12c (35 mg, 0.070 mmol) as the starting material. DCM : MeOH : NH_4_OH = 9 : 1 : 0.1 was used as the eluent for flash column chromatography. Yield: 33% (23 mg); white solid; *R*_f_ (MeOH : NH_4_OH = 9 : 1 : 0.1) = 0.10; ^1^H NMR (400 MHz, DMSO-*d*_6_) *δ* 9.20 (s, 1H), 8.25–8.09 (m, 1H), 7.95–7.70 (m, 1H), 7.69–7.46 (m, 1H), 7.45–7.26 (m, 5H), 7.25–6.91 (m, 2H), 5.92 (s, 1H), 4.76–4.43 (m, 2H), 3.47–3.37 (m, 4H), 2.85 (s, 3H), 2.79–2.70 (m, 4H) ppm; ^13^C NMR (101 MHz, DMSO-*d*_6_) *δ*^13^C NMR (101 MHz, DMSO) *δ* 170.9, 170.2, 162.3, 160.8, 157.3, 140.9, 137.2, 136.7, 128.8, 128.7, 127.6, 127.1, 126.9, 119.5, 119.3, 117.0, 84.5, 54.1, 49.8, 45.2, 44.6, 36.9, 32.5 ppm, rotamers are present in the spectrum; HRMS (ESI+) calcd for C_23_H_27_N_6_O *m*/*z* [M + H]^+^: 403.22409, found: 403.22385; UHPLC: *t*_r_: 2.80 min (99.7% at 254 nm).

#### *N*-Methyl-*N*-phenethyl-3-((6-(piperazin-1-yl)pyrimidin-4-yl)amino)benzamide (13d)

Compound 13d was prepared according to the general procedure D using 12d (60 mg, 0.117 mmol) as the starting material. DCM : MeOH : NH_4_OH = 9 : 1 : 0.1 was used as the eluent for flash column chromatography. Yield: 76%; white solid; *R*_f_ (DCM : MeOH : NH_4_OH = 9 : 1 : 0.1) = 0.10; ^1^H NMR (400 MHz, DMSO-*d*_6_) *δ* 9.16 (s, 1H), 8.19 (d, *J* = 8.9 Hz, 1H), 7.66 (s, 1H), 7.60–7.49 (m, 1H), 7.36–7.11 (m, 5H), 7.01 (d, *J* = 6.4 Hz, 1H), 6.86–6.60 (m, 1H), 5.92 (s, 1H), 3.70–3.62 (m, 1H), 3.54–3.37 (m, 5H), 3.04–2.79 (m, 5H) 2.78–2.71 (m, 4H) ppm; ^13^C NMR (101 MHz, DMSO) *δ* 170.7, 170.0, 162.2, 160.9, 157.3, 140.8, 139.2, 138.4, 137.2, 128.8, 128.6, 128.4, 126.3, 119.7, 119.5, 119.3, 119.0, 117.3, 116.8, 84.5, 52.3, 50.7, 48.3, 45.0, 44.3, 43.5, 37.4, 34.0, 32.7, 32.4 ppm, rotamers are present in the spectrum; HRMS (ESI+) calcd for C_24_H_28_N_6_O_2_ [M + H]^+^: 417.2397, found: 417.2386; UPLC: *t*_r_: 2.84 min (99.5% at 254 nm).

#### *N*-Methyl-*N*-(2-phenoxyethyl)-3-((6-(piperazin-1-yl)pyrimidin-4-yl)amino)benzamide (13e)

Compound 13e was prepared according to the general procedure D using 12e (70 mg, 0.131 mmol) as the starting material. DCM : MeOH : NH_4_OH = 9 : 1 : 0.1 was used as the eluent for flash column chromatography. Yield: 74% (42 mg); white solid; *R*_f_ (MeOH : NH_4_OH = 9 : 1 : 0.1) = 0.11; ^1^H NMR (400 MHz, DMSO-*d*_6_) *δ* 9.20 (s, 1H), 8.19 (s, 1H), 7.86–7.67 (m, 1H), 7.66–7.48 (m, 1H), 7.37–7.19 (m, 3H), 7.05–6.81 (m, 4H), 5.93 (s, 1H), 4.30–4.01 (m, 2H), 3.89–3.54 (m, 2H), 3.51–3.38 (m, 4H), 3.03 (s, 3H), 2.88–2.69 (m, 4H) ppm; ^13^C NMR (101 MHz, DMSO-*d*_6_) *δ* 171.3, 170.4, 162.2, 160.8, 158.4, 158.0, 157.3, 140.9, 137.2, 136.9, 129.5, 129.5, 128.7, 120.8, 119.8, 119.4, 117.4, 114.5, 114.3, 84.5, 65.1, 64.5, 49.6, 46.4, 45.1, 44.6, 38.5, 32.6 ppm, rotamers are present in the spectrum; HRMS (ESI+) calcd for C_24_H_29_N_6_O_2_*m*/*z* [M + H]^+^: 433.23465, found: 433.23438; UHPLC: *t*_r_: 2.86 min (99.0% at 254 nm).

#### *N*-Phenethyl-4-((6-(piperazin-1-yl)pyrimidin-4-yl)amino)benzamide (13f)

Compound 13f was prepared according to the general procedure D using 12f (75 mg, 0.149 mmol) as the starting material. DCM : MeOH : NH_4_OH = 9 : 1 : 0.1 was used as the eluent for flash column chromatography. Yield: 49% (32 mg); white solid; *R*_f_ (DCM : MeOH : NH_4_OH = 9 : 1 : 0.1) = 0.10; ^1^H NMR (400 MHz, DMSO-*d*_6_) *δ* 9.35 (s, 1H), 8.39 (t, *J* = 5.5 Hz, 1H), 8.25 (s, 1H), 7.79–7.73 (m, 2H), 7.71–7.65 (m, 2H), 7.34–7.28 (m, 2H), 7.27–7.17 (m, 3H), 6.01 (s, 1H), 3.51–3.42 (m, 6H), 2.87–2.78 (m, 6H) ppm; ^13^C NMR (101 MHz, DMSO-*d*_6_) *δ* 165.8, 162.3, 160.7, 157.3, 143.6, 139.7, 128.7, 128.4, 128.0, 126.8, 126.1, 117.9, 85.0, 45.1, 44.5, 40.9, 35.3 ppm; HRMS (ESI+) calcd for C_23_H_27_N_6_O *m*/*z* [M + H]^+^: 403.22409, found: 403.22388; UHPLC: *t*_r_: 2.82 min (96.7% at 254 nm).

#### *N*-(2-Phenoxyethyl)-4-((6-(piperazin-1-yl)pyrimidin-4-yl)amino)benzamide (13g)

Compound 13g was prepared according to the general procedure D using 12g (52 mg, 0.100 mmol) as the starting material. DCM : MeOH : NH_4_OH = 9 : 1 : 0.1 was used as the eluent for flash column chromatography. Yield: 83% (35 mg); white solid; *R*_f_ (MeOH : NH_4_OH = 9 : 1 : 0.1) = 0.11; ^1^H NMR (400 MHz, DMSO-*d*_6_) *δ* 9.34 (s, 1H), 8.51 (t, *J* = 5.4 Hz, 1H), 8.24 (s, 1H), 7.85–7.75 (m, 2H), 7.72–7.64 (m, 2H), 7.34–7.22 (m, 2H), 7.01–6.89 (m, 3H), 5.99 (s, 1H), 4.10 (t, *J* = 5.9 Hz, 2H), 3.61 (q, *J* = 5.9 Hz, 2H), 3.51–3.40 (m, 4H), 2.83–2.74 (m, 4H) ppm; ^13^C NMR (101 MHz, DMSO-*d*_6_) *δ* 166.2, 162.3, 160.6, 158.4, 157.3, 143.7, 129.5, 128.1, 126.4, 120.6, 117.8, 114.5, 85.0, 66.0, 45.0, 44.4, 38.8 ppm; HRMS (ESI+) calcd for C_23_H_27_N_6_O_2_*m*/*z* [M + H]^+^: 419.21900, found: 419.21865; UHPLC: *t*_r_: 2.82 min (99.1% at 254 nm).

#### *N*-Benzyl-*N*-methyl-4-((6-(piperazin-1-yl)pyrimidin-4-yl)amino)benzamide (13h)

Compound 13h was prepared according to the general procedure D using 12h (90 mg, 0.179 mmol) as the starting material. DCM : MeOH : NH_4_OH = 9 : 1 : 0.1 was used as the eluent for flash column chromatography. Yield: 56% (40 mg); white solid; *R*_f_ (MeOH : NH_4_OH = 9 : 1 : 0.1) = 0.11; ^1^H NMR (400 MHz, DMSO-*d*_6_) *δ* 9.30 (s, 1H), 8.22 (s, 1H), 7.67 (d, *J* = 8.4 Hz, 2H), 7.46–7.15 (m, 7H), 5.98 (s, 1H), 4.81–3.94 (m, 2H), 3.52–3.41 (m, 4H), 2.88 (s, 3H), 2.84–2.73 (m, 4H) ppm; ^13^C NMR (101 MHz, DMSO-*d*_6_) *δ* 170.9, 170.6, 162.2, 160.7, 157.3, 142.2, 137.4, 128.7, 128.4, 128.0, 128.0, 127.2, 118.2, 84.8, 54.3, 54.2, 50.1, 44.8, 44.1, 37.1, 32.8 ppm; HRMS (ESI+) calcd for C_23_H_27_N_6_O *m*/*z* [M + H]^+^: 403.22409, found: 403.22389; UHPLC: *t*_r_: 2.80 min (98.3% at 254 nm).

#### *N*-Methyl-*N*-phenethyl-4-((6-(piperazin-1-yl)pyrimidin-4-yl)amino)benzamide (13i)

Compound 13i was prepared according to the general procedure D using 12i (69 mg, 0.136 mmol) as the starting material. DCM : MeOH : NH_4_OH = 9 : 1 : 0.1 was used as the eluent for flash column chromatography. Yield: 99%; white solid; *R*_f_ (DCM : MeOH : NH_4_OH = 9 : 1 : 0.1) = 0.68; ^1^H NMR (400 MHz, DMSO-*d*_6_) *δ* 9.31 (s, 1H), 8.25 (s, 1H), 7.62 (d, *J* = 6.4 Hz, 2H), 7.36–7.00 (m, 7H), 6.36 (s, 1H), 6.01 (s, 1H), 3.66–3.43 (m, 6H), 3.03–2.79 (m, 9H) ppm; ^13^C NMR (101 MHz, DMSO-*d*_6_) 169.8, 162.0, 160.8, 158.1, 157.8, 157.5, 157.4, 141.7, 129.1, 128.8, 128.5, 128.4, 127.6, 126.3, 118.8, 118.2, 115.8, 85.0, 49.2, 44.4, 43.9, 42.8, 38.1, 34.2, 33.2, 32.9 ppm, rotamers are present in the spectra and some signals were determined using a HSQC spectrum; HRMS (ESI+) calcd for C_24_H_28_N_6_O_2_ [M + H]^+^: 417.2400, found: 417.2386; UPLC: *t*_r_: 2.91 min (99.7% at 254 nm).

#### *N*-Methyl-*N*-(2-phenoxyethyl)-4-((6-(piperazin-1-yl)pyrimidin-4-yl)amino)benzamide (13j)

Compound 13j was prepared according to the general procedure D using 12j (36 mg, 0.068 mmol) as the starting material. DCM : MeOH : NH_4_OH = 9 : 1 : 0.1 was used as the eluent for flash column chromatography. Yield: 82% (24 mg); white solid; *R*_f_ (MeOH : NH_4_OH = 9 : 1 : 0.1) = 0.10; ^1^H NMR (400 MHz, DMSO-*d*_6_) *δ* 9.26 (s, 1H), 8.22 (s, 1H), 7.69–7.63 (m, 2H), 7.39–7.24 (m, 4H), 7.02–6.86 (m, 3H), 5.96 (s, 1H), 4.26–4.03 (m, 3H), 3.83–3.62 (m, 2H), 3.47–3.38 (m, 4H), 3.04 (s, 3H), 2.81–2.70 (m, 4H) ppm; ^13^C NMR (101 MHz, DMSO-*d*_6_) *δ* 171.1, 162.3, 160.7, 158.2, 157.3, 143.0, 129.5, 128.0, 120.8, 118.1, 114.4, 84.7, 64.8, 49.9, 46.8, 45.2, 44.7, 32.8 ppm; HRMS (ESI+) calcd for C_24_H_29_N_6_O_2_*m*/*z* [M + H]^+^: 433.23465, found: 433.23444; UHPLC: *t*_r_: 2.93 min (98.1% at 254 nm).

#### *N*-Methyl-*N*-phenethyl-3-((6-(piperidin-1-yl)pyrimidin-4-yl)amino)benzamide (14)

Compound 14 was prepared according to the general procedure C using 10d (100 mg, 0.271 mmol) as the starting material. DCM : MeOH = 20 : 1 was used as the eluent for flash column chromatography. Yield: 50%; white solid; *R*_f_ (DCM : MeOH = 20 : 1) = 0.16; ^1^H NMR (400 MHz, DMSO-d6) *δ* 9.20 (s, 1H), 8.26 (d, *J* = 8.4 Hz, 1H), 7.74 (s, 1H), 7.67–7.56 (m, 1H), 7.43–7.19 (m, 5H), 7.11–7.05 (m, 1H), 6.92–6.70 (m, 1H), 3.73 (t, *J* = 7.5 Hz, 1H), 3.58 (t, *J* = 5.3 Hz, 4H), 3.11–2.82 (m, 5H), 1.73–1.64 (m, 2H), 1.63–1.56 (m, 4H) ppm, signal for the remaining proton is overlapped with solvent; ^13^C NMR (101 MHz, DMSO-*d*_6_) 170.7, 170.0, 161.8, 160.9, 157.4, 140.8, 139.2, 138.4, 137.2, 128.8, 128.6, 128.4, 126.3, 119.6, 119.4, 119.2, 118.8, 117.2, 116.7, 84.3, 52.3, 48.3, 44.6, 37.4, 34.0, 32.7, 32.4, 24.9, 24.2 ppm, rotamers are present in the spectrum; HRMS (ESI+) calcd for C_25_H_29_N_5_O [M + H]^+^: 416.2445, found: 416.2433; UPLC: *t*_r_: 4.14 min (100% at 254 nm).

#### *N*-Methyl-3-((6-morpholinopyrimidin-4-yl)amino)-*N*-phenethylbenzamide (15)

Compound 15 was prepared according to the general procedure C using 10d (81 mg, 0.221 mmol) as the starting material. DCM : MeOH = 20 : 1 was used as the eluent for flash column chromatography. Yield: 20%; white solid; *R*_f_ (DCM : MeOH = 20 : 1) = 0.19; ^1^H NMR (400 MHz, DMSO-*d*_6_) *δ* 9.24 (s, 1H), 8.22 (d, *J* = 9.0 Hz, 1H), 7.66 (s, 1H), 7.60–7.49 (m, 1H), 7.35–7.11 (m, 5H), 7.01 (d, *J* = 7.2 Hz, 1H), 6.87–6.66 (m, 1H), 5.95 (s, 1H), 3.71–3.63 (m, 5H), 3.51–3.35 (m, 5H), 3.04–2.79 (m, 5H) ppm; ^13^C NMR (101 MHz, DMSO-*d*_6_) 170.7, 169.9, 162.4, 160.9, 157.3, 140.7, 139.2, 138.4, 137.2, 128.8, 128.6, 128.4, 126.3, 119.8, 119.6, 119.4, 119.1, 117.4, 116.9, 84.8, 65.8, 52.3, 48.3, 43.9, 37.4, 36.7, 34.0, 32.7, 32.4 ppm, rotamers are present in the spectrum; HRMS (ESI+) calcd for C_24_H_27_N_5_O_2_ [M + H]^+^: 418.2238, found: 418.2228; UPLC: *t*_r_: 3.60 min (99.6% at 254 nm).

#### 3-((6-(4-Hydroxypiperidin-1-yl)pyrimidin-4-yl)amino)-*N*-methyl-*N*-phenethylbenzamide (16)

Compound 16 was prepared according to the general procedure C using 10d (80 mg, 0.219 mmol) as the starting material. DCM : MeOH = 15 : 1 was used as the eluent for flash column chromatography. Yield: 87%; white solid; *R*_f_ (DCM : MeOH = 15 : 1) = 0.20; ^1^H NMR (400 MHz, DMSO-*d*_6_) *δ* 9.16 (s, 1H), 8.20 (d, *J* = 8.3 Hz, 1H), 7.66 (s, 1H), 7.60–7.48 (m, 1H), 7.35–7.12 (m, 5H), 7.01 (d, *J* = 7.3 Hz, 1H), 6.86–6.65 (m, 1H), 5.97 (s, 1H), 4.76 (s, 1H), 3.93 (d, *J* = 13.2 Hz, 2H), 3.77–3.63 (m, 2H), 3.14 (t, *J* = 11.5 Hz, 2H), 3.04–2.79 (m, 5H), 1.82–1.72 (m, 2H), 1.33 (q, *J* = 10.8 Hz, 2H) ppm; ^13^C NMR (101 MHz, DMSO-*d*_6_): 170.7, 170.0, 161.8, 160.9, 157.4, 140.8, 139.2, 138.4, 137.2, 128.8, 128.6, 128.4, 126.3, 119.7, 119.5, 119.2, 118.9, 117.3, 116.7, 84.4, 66.0, 54.9, 52.3, 48.3, 41.4, 37.4, 34.0, 33.6, 32.7, 32.4 ppm, rotamers are present in the spectrum; HRMS (ESI+) calcd for C_25_H_29_N_5_O_2_ [M + H]^+^: 432.2394, found: 432.2383; UPLC: *t*_r_: 3.41 min (99.8% at 254 nm).

#### *tert*-Butyl(1-(6-((3-(methyl(phenethyl)carbamoyl)phenyl)amino)pyrimidin-4-yl)piperidin-4-yl)carbamate (17)

Compound 17 was prepared according to the general procedure C using 10d (120 mg, 0.327 mmol) as the starting material. DCM : MeOH = 20 : 1 was used as the eluent for flash column chromatography. Yield: 71%; white solid; *R*_f_ (DCM : MeOH = 20 : 1) = 0.13; ^1^H NMR (400 MHz, DMSO-*d*_6_) *δ* 9.15 (s, 1H), 8.19 (d, *J* = 8.2 Hz, 1H), 7.66 (s, 1H), 7.59–7.48 (m, 1H), 7.35–7.12 (m, 5H), 7.01 (d, *J* = 7.3 Hz, 1H), 6.87 (d, *J* = 7.9 Hz, 1H), 6.87–6.67 (m, 1H), 5.96 (s, 1H), 4.14 (d, *J* = 13.2 Hz, 2H), 3.71–3.63 (m, 1H), 3.58–3.35 (m, 2H), 3.03–2.79 (m, 7H), 1.77 (d, *J* = 11.8 Hz, 2H), 1.39 (s, 9H), 1.36–1.19 (m, 2H) ppm; MS (ESI+) C_30_H_38_N_6_O_3_*m*/*z*: 531.9 [M + H]^+^.

#### 3-((6-(4-Aminopiperidin-1-yl)pyrimidin-4-yl)amino)-*N*-methyl-*N*-phenethylbenzamide (18)

Compound 18 was prepared according to the general procedure D using 17 (60 mg, 0.113 mmol) as the starting material. DCM : MeOH : NH_4_OH = 9 : 1 : 0.1 was used as the eluent for flash column chromatography. Yield: 82%; white solid; *R*_f_ (DCM : MeOH : NH_4_OH = 9 : 1 : 0.1) = 0.10; ^1^H NMR (400 MHz, DMSO-*d*_6_) *δ* 9.13 (s, 1H), 8.19 (d, *J* = 8.4 Hz, 1H), 7.66 (s, 1H), 7.60–7.46 (m, 1H), 7.35–7.11 (m, 5H), 7.01 (d, *J* = 7.3 Hz, 1H), 6.85–6.60 (m, 1H), 5.96 (d, *J* = 1.0 Hz, 1H), 4.10 (d, *J* = 13.2 Hz, 2H), 3.70–3.62 (m, 1H), 3.05–2.77 (m, 8H), 2.01–1.81 (m, 2H), 1.79–1.68 (m, 2H), 1.23–1.08 (m, 2H) ppm, signals for the remaining proton is overlapped with solvent; ^13^C NMR (101 MHz, DMSO-*d*_6_) 171.2, 170.5, 162.2, 161.4, 157.9, 141.3, 139.6, 138.9, 137.6, 129.3, 129.1, 128.8, 126.7, 120.1, 119.9, 119.7, 119.3, 117.7, 117.1, 84.9, 52.7, 48.7, 43.0, 37.9, 34.9, 34.5, 33.2, 32.9 ppm, rotamers are present in the spectrum; HRMS (ESI+) calcd for C_25_H_30_N_6_O [M + H]^+^: 431.2554, found: 431.2543; UPLC: *t*_r_: 2.89 min (100% at 254 nm).

#### *N*-(2-Phenoxyethyl)-4-((6-(piperidin-1-yl)pyrimidin-4-yl)amino)benzamide (19)

Compound 19 was prepared according to the general procedure C using 10g (100 mg, 0.271 mmol) as the starting material. DCM : MeOH = 9 : 1 was used as the eluent for flash column chromatography. Yield: 70% (79 mg); off-white solid; *R*_f_ (DCM : MeOH = 9 : 1) = 0.48; ^1^H NMR (400 MHz, DMSO-*d*_6_) *δ* 9.31 (s, 1H), 8.55–8.47 (m, 1H), 8.23 (s, 1H), 7.83–7.75 (m, 3H), 7.72–7.64 (m, 3H), 7.34–7.24 (m, 3H), 7.00–6.89 (m, 5H), 6.01 (s, 1H), 4.10 (t, *J* = 6.0 Hz, 2H), 3.61 (q, *J* = 5.9 Hz, 2H), 3.52 (t, *J* = 5.5 Hz, 4H), 1.66–1.58 (m, 1H), 1.57–1.48 (m, 2H) ppm; ^13^C NMR (101 MHz, DMSO-*d*_6_) *δ* 166.2, 161.9, 160.7, 158.5, 157.4, 143.8, 129.5, 128.1, 126.3, 120.6, 117.8, 114.5, 84.8, 66.0, 44.6, 38.8, 24.9, 24.2 ppm; HRMS (ESI+) calcd for C_24_H_28_N_5_O_2_*m*/*z* [M + H]^+^: 418.22281, found: 418.22281; UHPLC: *t*_r_: 3.83 min (99.5% at 254 nm).

#### 4-((6-Morpholinopyrimidin-4-yl)amino)-*N*-(2-phenoxyethyl)benzamide (20)

Compound 20 was prepared according to the general procedure C using 10g (80 mg, 0.217 mmol) as the starting material. DCM : MeOH = 15 : 1 was used as the eluent for flash column chromatography. Yield: 80% (73 mg); off-white solid; *R*_f_ (DCM : MeOH = 15 : 1) = 0.14; ^1^H NMR (400 MHz, DMSO-*d*_6_) *δ* 9.40 (s, 1H), 8.52 (t, *J* = 5.6 Hz, 1H), 8.27 (s, 1H), 7.80 (dt, *J* = 7.1, 2.3 Hz, 2H), 7.75–7.65 (m, 2H), 7.34–7.24 (m, 2H), 7.01–6.89 (m, 3H), 6.02 (d, *J* = 1.0 Hz, 1H), 4.10 (t, *J* = 6.0 Hz, 2H), 3.71–3.64 (m, 4H), 3.61 (q, *J* = 5.9 Hz, 2H), 3.46 (t, *J* = 5.0 Hz, 4H) ppm; ^13^C NMR (101 MHz, DMSO-*d*_6_) *δ* 166.2, 162.5, 160.7, 158.5, 157.3, 143.6, 129.5, 128.1, 126.6, 120.6, 118.0, 114.5, 85.2, 66.0, 65.8, 43.9, 38.8 ppm; HRMS (ESI+) calcd for C_23_H_26_N_5_O_3_*m*/*z* [M + H]^+^: 420.20302, found: 420.20202; UHPLC: *t*_r_: 3.33 min (100% at 254 nm).

#### 4-((6-(4-Hydroxypiperidin-1-yl)pyrimidin-4-yl)amino)-*N*-(2-phenoxyethyl)benzamide (21)

Compound 21 was prepared according to the general procedure C using 10g (80 mg, 0.217 mmol) as the starting material. Yield: 73% (69 mg); light yellow solid; *R*_f_ (DCM : MeOH = 15 : 1) = 0.08; ^1^H NMR (400 MHz, DMSO-*d*_6_) *δ* 9.31 (s, 1H), 8.51 (t, *J* = 5.6 Hz, 1H), 8.24 (d, *J* = 0.9 Hz, 1H), 7.83–7.75 (m, 2H), 7.73–7.64 (m, 2H), 7.34–7.24 (m, 2H), 7.01–6.89 (m, 3H), 6.03 (d, *J* = 1.0 Hz, 1H), 4.75 (d, *J* = 2.9 Hz, 1H), 4.10 (t, *J* = 6.0 Hz, 2H), 3.98–3.90 (m, 2H), 3.73 (s, 1H), 3.61 (q, *J* = 5.9 Hz, 2H), 3.15 (ddd, *J*_1_ = 13.2 Hz, *J*_2_ = 9.8 Hz, *J*_3_ = 3.3 Hz, 2H), 1.80–1.74 (m, 2H), 1.40–1.26 (m, 2H) ppm; ^13^C NMR (101 MHz, DMSO-*d*_6_) *δ* 166.2, 161.8, 160.7, 158.5, 157.4, 143.8, 129.5, 128.1, 126.4, 120.6, 117.8, 114.5, 84.9, 66.0, 65.9, 41.5, 38.8, 33.6 ppm; HRMS (ESI+) calcd for C_24_H_28_N_5_O_3_*m*/*z* [M + H]^+^: 434.21867, found: 434.21782; UHPLC: *t*_r_: 3.13 min (97.7% at 254 nm).

#### *tert*-Butyl (1-(6-((4-((2-phenoxyethyl)carbamoyl)phenyl)amino)pyrimidin-4-yl)piperidin-4-yl)carbamate (22)

Compound 22 was prepared according to the general procedure C using 10g (150 mg, 0.407 mmol) as the starting material. Yield: quantitative; white solid; *R*_f_ (DCM : MeOH = 20 : 1) = 0.06; ^1^H NMR (400 MHz, DMSO-*d*_6_) *δ* 9.36 (s, 1H), 8.52 (t, *J* = 5.5 Hz, 1H), 8.24 (s, 1H), 7.83–7.76 (m, 2H), 7.70–7.64 (m, 2H), 7.34–7.24 (m, 2H), 7.00–6.84 (m, 5H), 6.03 (s, 1H), 4.20–4.08 (m, 4H), 3.61 (q, *J* = 6.0 Hz, 2H), 3.56–3.48 (m, 1H), 2.96 (t, *J* = 11.7 Hz, 2H), 1.81–1.74 (m, 2H), 1.45–1.21 (m, 11H) ppm; MS (ESI+) C_29_H_36_N_6_O_4_*m*/*z*: 539.9 [M + H]^+^.

#### 4-((6-(4-Aminopiperidin-1-yl)pyrimidin-4-yl)amino)-*N*-(2-phenoxyethyl)benzamide (23)

Compound 23 was prepared according to the general procedure D using 22 (120 mg, 0.225 mmol) as the starting material. DCM : MeOH : NH_4_OH = 9 : 1 : 0.1 was used as the eluent for flash column chromatography. The residue after evaporation was dissolved in DCM and MeOH, then EtOAc and hexane were added to precipitate the product. Yield: 77% (75 mg); light yellow solid; *R*_f_ (DCM : MeOH : NH_4_OH = 9 : 1 : 0.1) = 0.03; ^1^H NMR (400 MHz, DMSO-*d*_6_) *δ* 9.33 (s, 1H), 8.53 (t, *J* = 5.7 Hz, 1H), 8.24 (d, *J* = 0.7 Hz, 1H), 7.84–7.76 (m, 2H), 7.75–7.65 (m, 2H), 7.34–7.24 (m, 2H), 7.03–6.89 (m, 3H), 6.04 (d, *J* = 1.0 Hz, 1H), 4.16–4.06 (m, 4H), 3.62 (q, *J* = 5.9 Hz, 2H), 3.02–2.89 (m, 2H), 2.87–2.77 (m, 1H), 1.80–1.70 (m, 2H), 1.23–1.09 (m, 2H) ppm; ^13^C NMR (101 MHz, DMSO-*d*_6_) *δ* 166.2, 161.8, 160.7, 158.5, 157.5, 143.8, 129.6, 128.1, 126.3, 120.6, 117.8, 114.5, 84.9, 66.0, 48.3, 42.5, 38.8, 34.4 ppm; HRMS (ESI+) calcd for C_24_H_29_N_6_O_2_*m*/*z* [M + H]^+^: 433.23465, found: 433.23387; UHPLC: *t*_r_: 2.78 min (99.6% at 254 nm).

#### 4-((6-(4-(Cyanomethyl)piperazin-1-yl)pyrimidin-4-yl)amino)-*N*-(2-phenoxyethyl)benzamide (24)

Compound 13g (100 mg, 239 mmol) was dissolved in a mixture of MeCN (5 mL) and DCM (1 mL). Then, bromoacetonitrile (20 μL, 1.2 equiv.) was added and the reaction was stirred overnight at room temperature. A precipitate that formed was filtered off, and dried to give a clean product. Yield: 62% (68 mg); white solid; *R*_f_ (DCM : MeOH : NH_4_OH = 9 : 1) = 0.42; ^1^H NMR (400 MHz, DMSO-*d*_6_) *δ* 9.39 (s, 1H), 8.52 (t, *J* = 5.5 Hz, 1H), 8.27 (s, 1H), 7.83–7.76 (m, 2H), 7.72–7.65 (m, 2H), 7.34–7.24 (m, 2H), 7.00–6.89 (m, 3H), 6.05 (d, *J* = 1.0 Hz, 1H), 4.10 (t, *J* = 6.0 Hz, 2H), 3.80 (s, 2H), 3.61 (q, *J* = 5.8 Hz, 2H), 3.58–3.54 (m, 4H), 2.57–2.52 (m, 4H) ppm; ^13^C NMR (101 MHz, DMSO-*d*_6_) *δ* 165.2, 161.1, 159.7, 157.5, 156.4, 142.6, 128.5, 127.1, 125.6, 119.6, 116.9, 114.7, 113.5, 84.3, 65.0, 49.7, 44.1, 42.2, 37.8 ppm; HRMS (ESI+) calcd for C_25_H_28_N_7_O_2_*m*/*z* [M + H]^+^: 458.22990, found: 458.22894; UHPLC: *t*_r_: 3.37 min (95.2% at 254 nm).

### Cell culture

Hormone-positive breast cancer cell line MCF-7 (ATCC-HTB-22; ATCC) was grown in Dulbecco's Modified Eagle's Medium (DMEM), while Ewing sarcoma cell line SK-N-MC (a kind gift from Beat Schäfer) and THP-1 (ATCC-TIB-202; ATCC) cells were grown in Roswell Park Memorial Institute (RPMI) 1640. The medium was supplemented with 10% heat-inactivated fetal bovine serum (Gibco, Thermo Fisher Scientific, Waltham, MA, USA), 100 U per mL penicillin, 100 μg per mL streptomycin, and 2 mM l-glutamine (all from Sigma-Aldrich, St. Louis, MO, USA). All cell lines were incubated at 37 °C in a 5% CO_2_ atmosphere.

### Cell viability assay

The antiproliferative activity of the compounds was evaluated against MCF-7 (ATCC-HTB-22; ATCC), SK-N-MC (a kind gift from Beat Schäfer) and THP-1 (ATCC-TIB-202; ATCC) cancer cell lines using the MTS assay (Promega, Madison, WI, USA). Cells were seeded in 96-well plates at a density of 2 × 10^4^ cells per mL in 100 μL of growth medium for THP-1 and were treated with compounds and controls directly, while 3 × 10^3^ cells per mL for MCF-7 and SK-N-MC were seeded in 100 μL of growth medium and allowed to adhere overnight following the treatment. In all cases the cells were treated with respective newly prepared compounds, positive controls (17-DMAG for MCF-7 and SK-N-MC; PU-H71 for THP-1) and a vehicle control (0.5% DMSO). The incubation time after treatment was 72 hours, then CellTiter96^®^ Aqueous One Solution Reagent (Promega, Madison, WI, USA) was added to each well. Following a 3 hours incubation, absorbance was measured at 492 nm using BioTek's Synergy™ 4 Hybrid Microplate Reader (Winooski, VT, USA). The measurements were performed in two biological repetitions and each time the experiment was performed in a triplicate. The IC_50_ values, representing the concentration of inhibitor required for half-maximal inhibition, are the average of three independent experiments and were calculated using GraphPad Prism 9.5.0 software (San Diego, CA, USA).

### Apoptosis assay

Apoptosis of SK-N-MC cells was evaluated by detecting phosphatidylserines using R-phycoerythrin–annexin V conjugate (R-PE annexin V; Invitrogen, Carlsbad, CA, USA) and nucleic acids in dead cells using SytoxBlue Dead Cell Stain (Invitrogen, Carlsbad, CA, USA), according to the manufacturer's instructions. Briefly, 2.5 × 10^5^ SK-N-MC cells were seeded in six-well plates. After 24 hours, cells were washed with PBS and treated with 8c and 13g in 10 and 20 μM concentrations for 24 and 48 hours. The medium with detached cells was then collected, and attached cells were harvested and combined with the detached cells. Following two washing steps with cold PBS, cells were resuspended in 100 μL annexin-binding buffer (Invitrogen, Carlsbad, CA, USA) containing 2.5 μL annexin V–R-PE solution and 750 nM SytoxBlue, and incubated in the dark for 15 minutes at room temperature. Before measurement, 200 μL of annexin-binding buffer was added. A minimum of 10 000 events were collected using a flow cytometer (Attune NxT; Invitrogen, Carlsbad, CA, USA). Annexin V (ANV)-/SYTOX Blue (SB)-indicates viable cells, ANV+/SB− indicates early apoptotic cells, ANV+/SB+ indicates late apoptotic cells, and ANV−/SB+ indicates necrotic cells.

### Western blot

MCF-7 cells were treated with compounds 8c (20 μM and 50 μM), 13g (20 μM and 50 μM) and DDO-5936 (20 μM) or 0.5% DMSO as a negative control. The cells were incubated with the compounds or negative control for 24 hours. After incubation, the cells were rinsed with PBS (Gibco, Thermo Fisher Scientific, Waltham, MA, USA) and lysed using RIPA buffer containing 50 mM Tris–HCl pH 7.4, 150 mM NaCl, 1% NP-40, 0.5% sodium deoxycholate, and 1 mM EDTA. The RIPA buffer was further supplemented with Halt™ Protease Inhibitor Cocktail and Halt™ Phosphatase Inhibitor Cocktail (both from Thermo Fisher Scientific, Waltham, MA, USA) at a 1 : 100 dilution. The lysates were then frozen for at least 24 hours. Upon thawing, the lysates were sonicated and centrifuged at 15 000 rpm for 20 minutes at 4 °C. Only the supernatants were collected, and protein concentration was measured using the DC protein assay (Bio-Rad, Hercules, California, USA). Proteins (20 μg) were separated by SDS-PAGE on a 10% acrylamide/bisacrylamide gel, running at 80 V for 15 minutes followed by 130 V for 60 minutes. The separated proteins were then transferred onto a nitrocellulose membrane using the iBlot 2 Dry Blotting System (Thermo Fisher Scientific, Waltham, MA, USA). The membranes were blocked with 5% BSA for 1 hour at room temperature to prevent nonspecific binding. Primary antibodies were then added, and the membranes were incubated overnight at 4 °C. The primary antibodies (Cell Signaling, Danvers, MA, USA) used included anti-β-ActinMouse mAb (1 : 5000), anti-Hsp90 Rabbit mAb (1 : 1000), anti-Hsp70 Mouse mAb (1 : 1000), anti-IGF1R Rabbit (1 : 1000), anti-CDK4 Rabbit (1 : 1000), anti-ERα Mouse mAb (1 : 1000), anti-Akt Rabbit mAb (1 : 1000), and anti-c-Raf Rabbit mAb (1 : 1000). Secondary antibodies included anti-rabbit IgG, HRP-linked antibody (1 : 10 000), and anti-mouse IgG, HRP-linked antibody (1 : 10 000), which were incubated with the membranes for 1 hour at room temperature. Following washes, SuperSignal™ West Femto Maximum Sensitivity Substrate (Thermo Fisher Scientific, Waltham, MA, USA) was applied. Visualization of the blots was performed using the UVITEC Cambridge Imaging System (UVITEC, Cambridge, UK), and densitometric analysis of the western blot bands was conducted using NineAlliance software. Relative protein densities were calculated in relation to β-actin, used as a loading control.

### Expression and purification of the full-length Hsp90β

For the protein expression of full-length Hsp90β, we received plasmids as a kind gift from Dr Asta Zubriené at the Institute of Biotechnology, Vilnius University, Lithuania. Hsp90β, tagged with N-terminal 6×His-tag, was expressed in *Escherichia coli* strain Rosetta. The cells were cultured in LB media supplemented with kanamycin (50 μg mL^−1^) and chloramphenicol (34 μg mL^−1^) at 37 °C, and protein expression was initiated with 0.5 mM isopropyl β-d-1-thiogalactopyranoside (IPTG) when the OD_600_ reached 0.8. Following induction, the cells were incubated for 16 hours at 18 °C before being harvested by centrifugation. The cells were resuspended in a lysis buffer containing 40 mM potassium phosphate pH 8.0, 400 mM KCl, 10 mM imidazole, and protease inhibitors (Sigma), and then lysed by sonication. After centrifugation, the proteins were initially purified using a Ni^2+^-affinity HisTrap column (GE Healthcare). Impurities were washed away with lysis buffer containing 20–40 mM imidazole, and Hsp90β was subsequently eluted with lysis buffer containing 300 mM imidazole. The purification process continued with size exclusion chromatography (SEC) using a Superdex-200 (16/600) column (GE Healthcare) and a running buffer of 50 mM Tris pH 7.5 at room temperature, 300 mM KCl. The purity of the fractions was confirmed by SDS-PAGE, and they were then concentrated. For the NMR experiments, Hsp90β was dialyzed against NMR buffer (50 mM potassium phosphate pD 7.5, 100 mM KCl, 2 mM DTT (98%, D10) in D_2_O) and subsequently frozen in liquid nitrogen.

### Microscale thermophoresis

The full-length Hsp90β was labeled using the Monolith His-Tag Labeling Kit RED-tris-NTA following the manufacturer's instructions (NanoTemper Technologies GmbH, Munich, Germany). The proteins were first diluted to a concentration of 8 nM in assay buffer (50 mM Tris–HCl, pH 7.4, containing 150 mM NaCl, 5% ethanol, and 10 mM MgCl_2_). To determine the *K*_d_ values, the proteins were mixed with the test compounds in a 1 : 1 ratio as stated in the manufacturers protocol. The final concentration of Hsp90β was 4 nM, with compounds added in the following ranges: 1000 μM to 5.86 μM for 8c; 312.5 μM to 0.15 μM for 13g and 125 μM to 3.91 μM for 1. Two independent *K*_d_ determinations were performed for compounds 8c and 13g. Higher compound concentrations both with and without Cdc37 resulted in aggregation due to insufficient solubility, so these measurements were excluded (MST curves colored in grey). Next, Cdc37 (AR09008PUN, OriGene Technologies, Inc., Rockville, MD, USA) was acquired for the purpose of PPI inhibition evaluation between full-length Hsp90β and Cdc37. The *K*_d_ values of compounds 8c and 13g with Hsp90β (4 nM) were reevaluated in the presence of Cdc37 (0.5 μM, 1 μM and 2 μM for 8c and 1 μM for 13g) using the following concentration ranges of inhibitors: 500 μM to 5.86 μM for 8c and 312.5 μM to 1.95 μM for 13g. In all experiments the compounds were incubated with the proteins for 15 minutes in the dark at room temperature. The mixtures were then loaded into Monolith NT.115 Premium Capillaries (NanoTemper Technologies GmbH, Munich, Germany). Thermophoresis was induced at 1475 ± 15 nm and measured using a Monolith NT.115 pico instrument (NanoTemper Technologies GmbH, Munich, Germany). Measurements were conducted at ambient temperature (24–25 °C), with excitation power set to 20% and MST power set to 40%, and a laser on time of 5 seconds. To calculate the *K*_d_ values, the average fluorescence responses for each concentration were plotted against the logarithm of compound concentrate on using GraphPad Prism software (GraphPad Software, Inc., La Jolla, CA).

### Ligand-observed protein NMR studies

High-resolution NMR spectra of 13g were recorded using a Bruker Avance Neo 600 MHz spectrometer with a cryoprobe at 25 °C. Data collection employed pulse sequences from the Bruker library, and analysis was conducted using Bruker Topspin 4.2.0. The residual water signal was suppressed by excitation sculpting^54^ with a 5 ms selective pulse, and a T_1_ρ filter of 100 ms was used to remove background protein resonances. The ^1^H spectral widths were set at 5263 Hz. NMR samples were prepared in a buffer containing 50 mM potassium phosphate (pD 7.5), 100 mM KCl in D_2_O, 5 mM MgSO_4_, 2 mM DTT-*d*_10_, 0.02% NaN_3_, and 2% DMSO-*d*_6_. Full proton assignment (see ESI Fig. S6 and Table S2†) was achieved using TOCSY, NOESY, ROESY, and HSQC spectra. The ^1^H STD spectra were recorded with a protein : ligand ratio of 1 : 200, with protein and ligand concentrations of 1.5 μM and 3 mM.

For the ^1^H STD ligand epitope mapping experiments,^55^ 65 536 data points (6.23 s and acquisition time) were used, with a relaxation delay of 1.63 s and 5120 scans. A short protein saturation time of 0.5 s was utilized to minimize the effect of relaxation on the STD amplification factors.^56^ Selective on-resonance saturation of Hsp90β was performed at −0.827 ppm, with the transmitter offset referenced to 4.70 ppm. Off-resonance irradiation for the reference spectrum was applied at 30 ppm. The spectra were zero-filled and apodized using an exponential line-broadening function of 3 Hz. Errors in the STD amplification factor were estimated using the specified formula:^57^
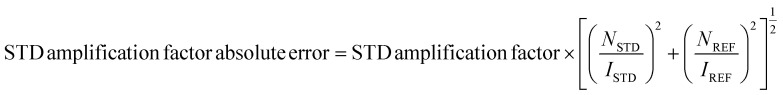
*N*_STD_ and *N*_REF_ are noise levels in STD and reference spectra. *I*_STD_ and *I*_REF_ are signal intensities in STD and reference spectra. The relative errors of the STD amplification factors (Tables S3 and S6†) for all protons are below 4%.

The trNOESY^58^ spectra were recorded with 4096 data points in *t*_2_, 64 scans, 360 complex points in *t*_1_, and a relaxation delay of 1.5 s. A mixing time of 150 ms was selected according to the binding affinity of the derivatives to compromise between sufficient signal-to-noise ratio and reduced spin diffusion. The spectra were apodized with a squared sine bell function shifted by π/2 in both dimensions. Distances for 13g were calculated from the cross-peak volumes, assuming the integrated intensity of proton pairs 2′, 4′ and 1′, 5′ in the *para*-substituted phenyl ring with a distance of 2.5 Å and that the distances of the protons in pyrimidine ring are the same.

### Conformation of compound 8c and 13g from NMR experiments

Conformation of 13g were calculated in Schrodinger Suite using distance constraints from trNOESY experiments (Table S4†). For conformational search systematic torsional sampling method with default settings was used.

## Data availability

The data supporting this article have been included as part of the ESI.†

## Conflicts of interest

The authors declare that they have no known competing financial interests or personal relationships that could have appeared to influence the work reported in this paper.

## Supplementary Material

RA-014-D4RA05878J-s001
