# Supplementary material for: Discovery of new Hsp90–Cdc37 protein–protein interaction inhibitors: in silico screening and optimization of anticancer activity
Source: RSC Adv. 2024 Sep 5;14(39):28347–75. doi: 10.1039/d4ra05878j (PMC11375794; doi:10.1039/d4ra05878j)
Supplement: RA-014-D4RA05878J-s001 [file RA-014-D4RA05878J-s001.pdf]

Supplementary Information (SI) for RSC Advances.
This journal is © The Royal Society of Chemistry 2024

Discovery of new Hsp90-Cdc37 protein-protein interaction inhibitors: *In silico* screening and optimization of anticancer activity

Jaka Dernovšek[a], Nina Gradišek[a], Živa Zajec[a], Dunja Urbančič[a], Jernej Cingl[a], Tjaša Goričan[b], Simona Golič Grdadolnik[b], Tihomir Tomašič[a,*]

[a] *Faculty of Pharmacy, University of Ljubljana, Aškerčeva cesta 7, 1000 Ljubljana, Slovenia*

[b] *Laboratory for Molecular Structural Dynamics, Theory Department, National Institute of Chemistry, Hajdrihova 19, 1001 Ljubljana, Slovenia*

Table of Contents

1. Virtual screening 2
2. Representative MST and K_d curves 3
3. Western blot images used for quantification 5
4. NMR with Hsp90β - supporting data 7
5. Representative HPLC traces of 8c and 13g 9
6. NMR spectra 10

Literature 51

1. Virtual screening

Table S1. Compounds used for building the ligand-based pharmacophore model. Original numbering from the publication is used.[1]

cpd	SMILES
17h	O=S(N(CC(OC)=O)C1=CC=C(NC2=NC(N3CCNCC3)=NC=C2)C=C1)(C4=C(C)C=C(C)C=C4C)=O
18h	O=S(N(CC(O)=O)C1=CC=C(NC2=NC(N3CCNCC3)=NC=C2)C=C1)(C4=C(C)C=C(C)C=C4C)=O
19	O=S(N(CC(NO)=O)C1=CC=C(NC2=NC(N3CCNCC3)=NC=C2)C=C1)(C4=C(C)C=C(C)C=C4C)=O
21	O=S(N(CC(C1=NNN=N1)=O)C2=CC=C(NC3=NC(N4CCNCC4)=NC=C3)C=C2)(C5=C(C)C=C(C) C=C5C)=O

2. Representative MST and K_d curves

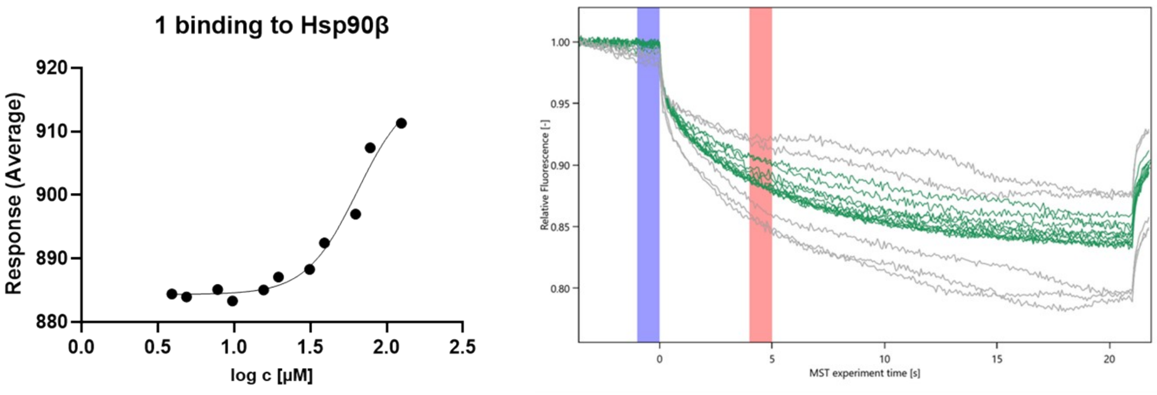

Figure S1. MST curves (colored in green) used for calculation of average response that was plotted against the logarithm of concentration of **1** in an attempt to evaluate the binding compound. Concentration range without the presence of aggregation was insufficient for K_d determination.

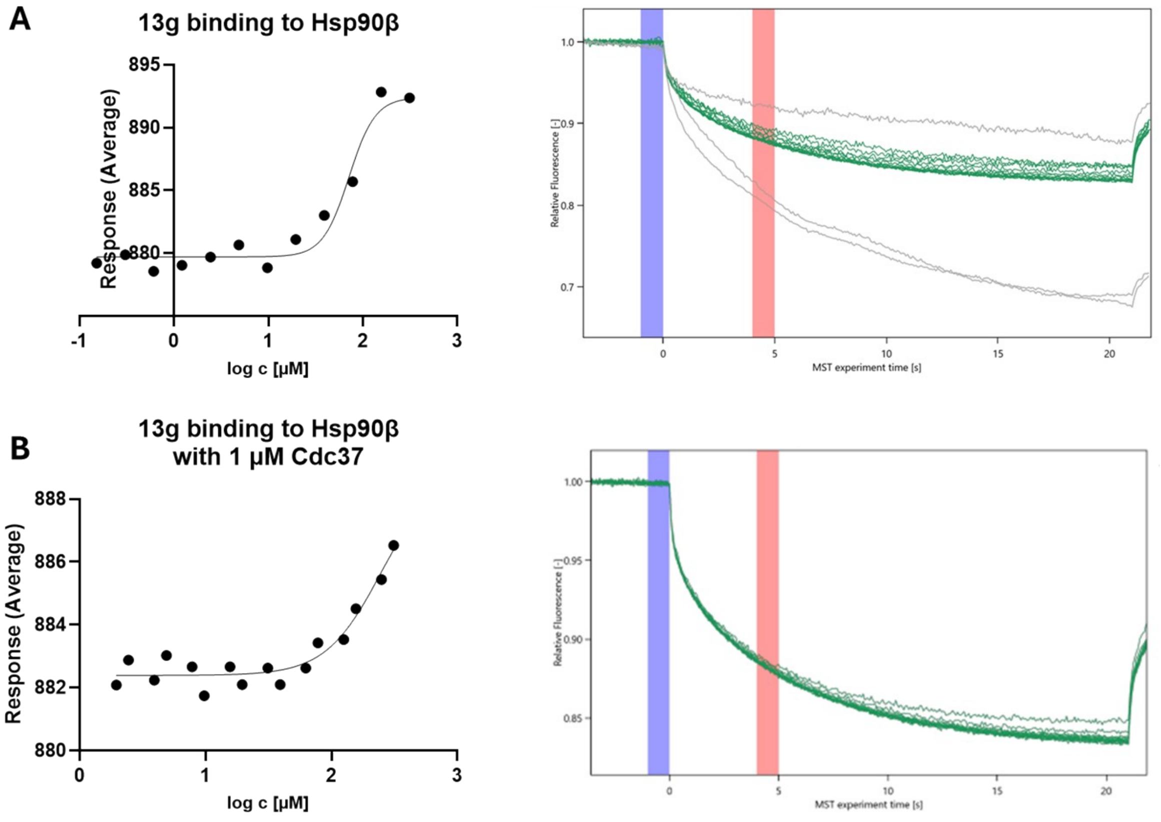

Figure S2. Representative MST curves (colored in green) used for calculation of average response that was plotted against the logarithm of concentration of **13g** to determine the K_d values of this compound with Hsp90β (**A**). In an attempt to determine the K_d of **13g** with Hsp90β in the presence of 1 µM Cdc37 (**B**) the concentration range used was insufficient to enable quantification.

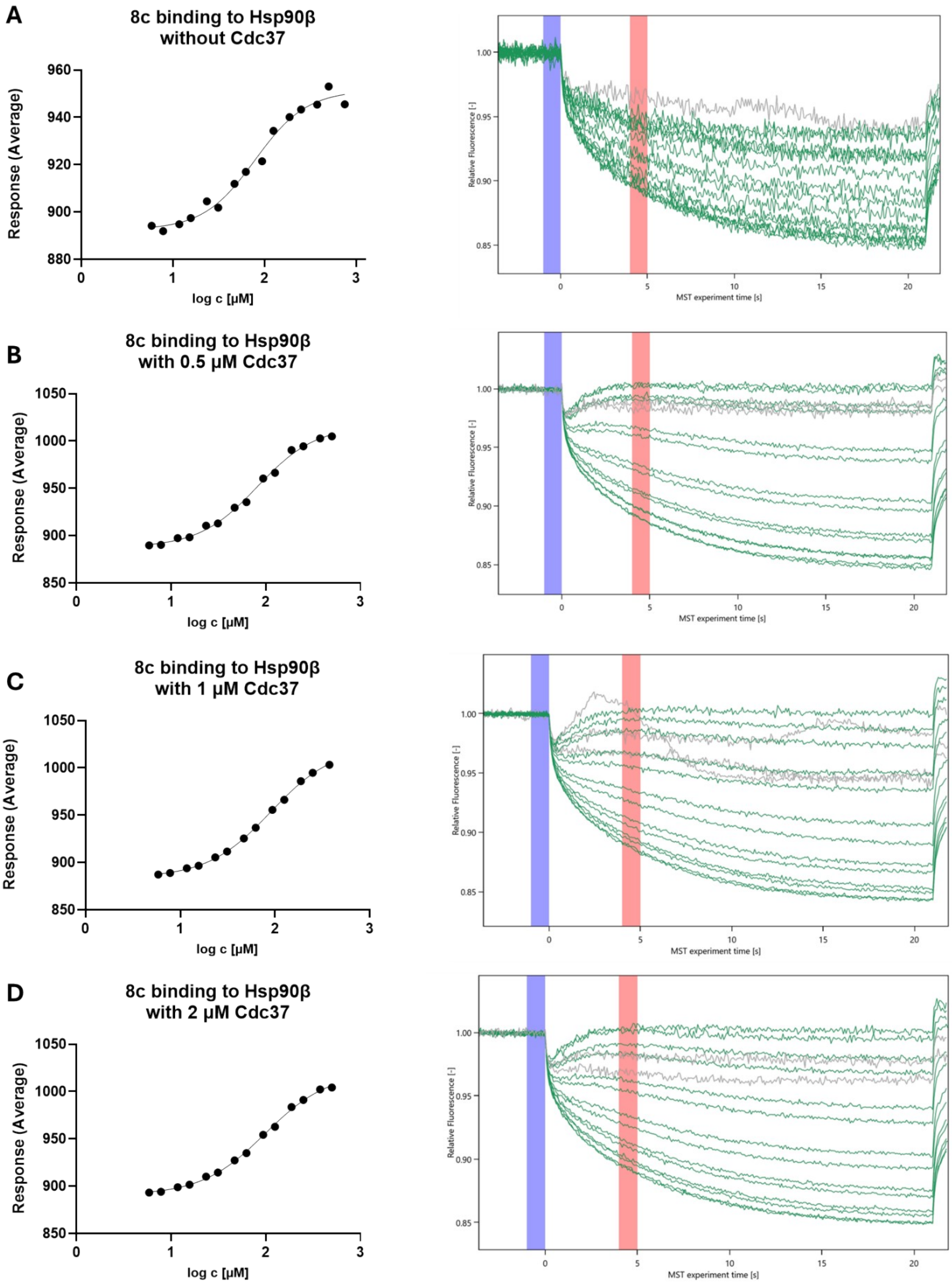

Figure S3. Representative MST curves (colored in green) used for calculation of average response that was plotted against the logarithm of concentration of **8c** to determine the K_d values of this compound with Hsp90β in the presence of 0 (**A**), 0.5 (**B**), 1 (**C**) and 2 (**D**) μM of Cdc37.

3. Western blot images used for quantification

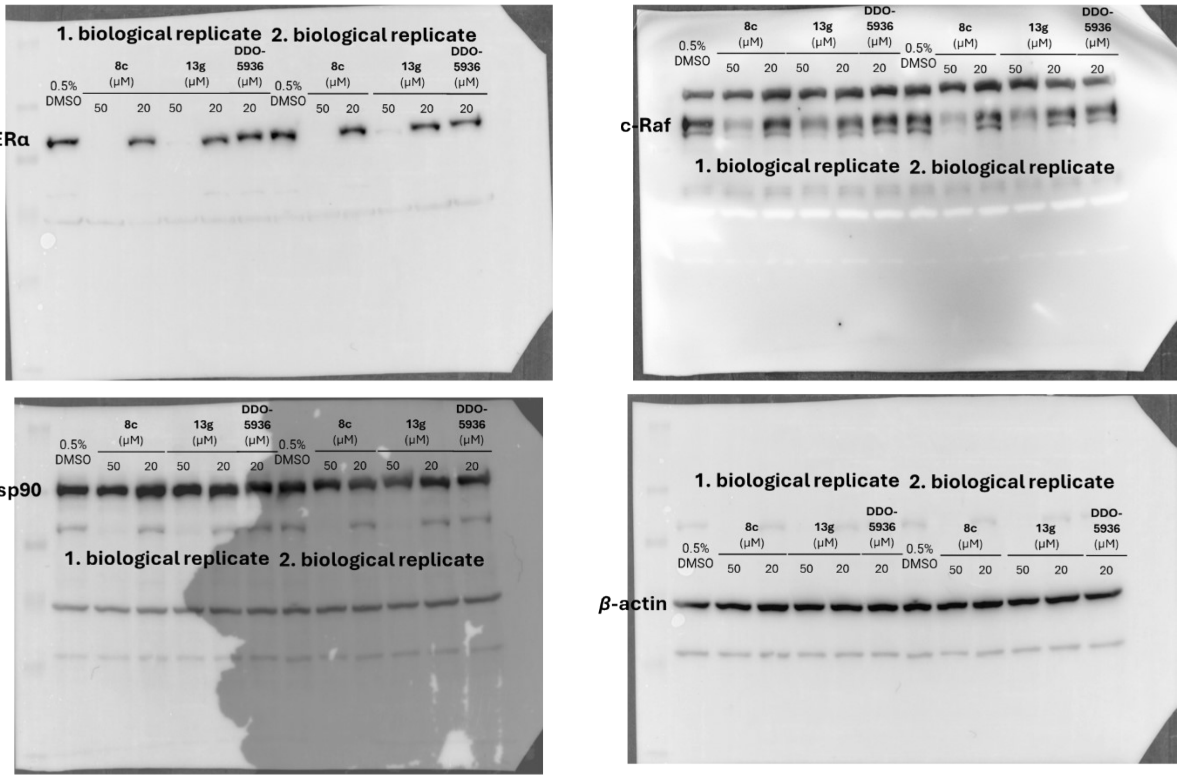

Figure S4. Western blot pictures used for quantification of protein in MCF-7 lysates for first and second biological repetitions of ERα, Hsp90 and c-Raf along with β-actin which was used as a standard.

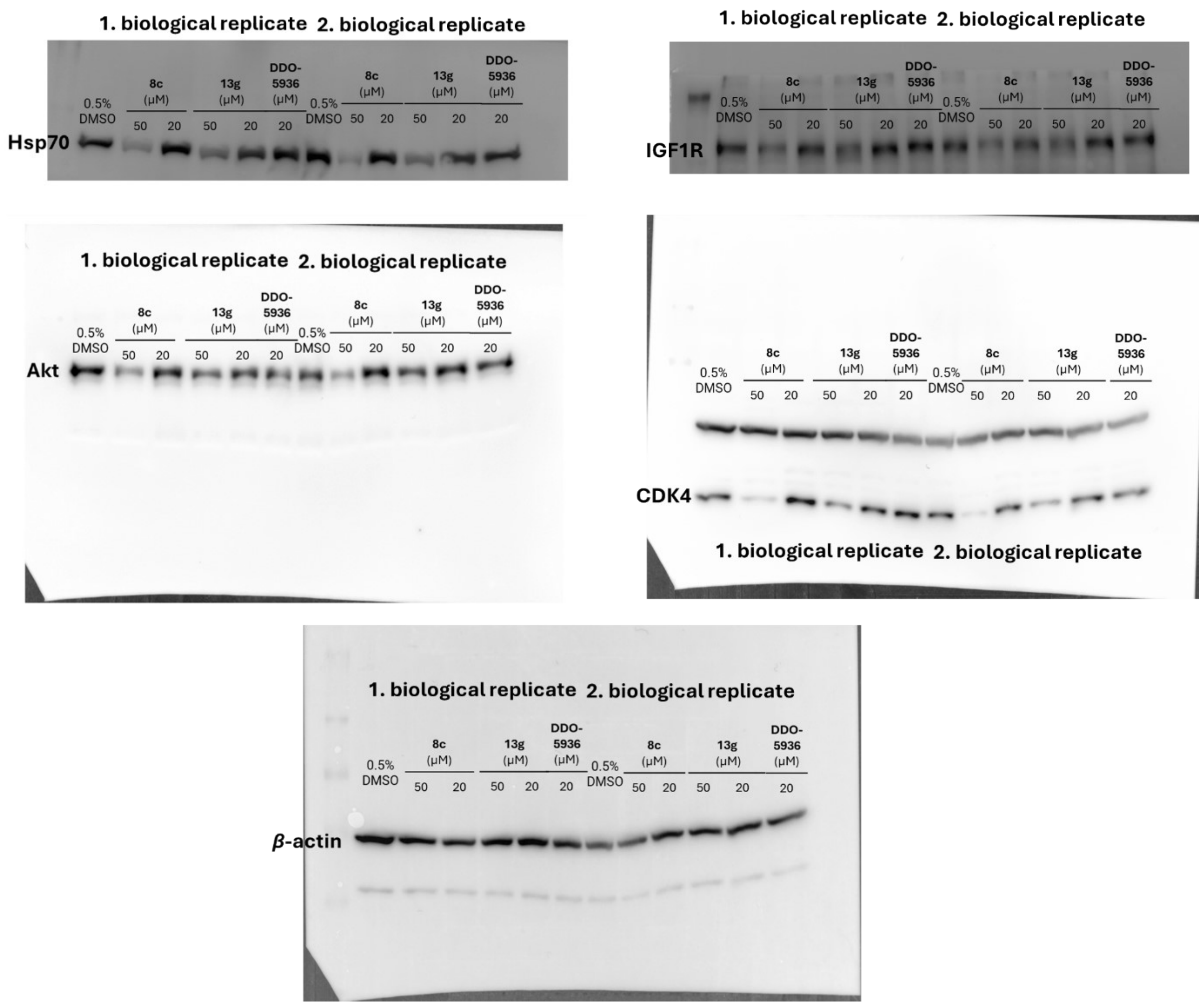

Figure S5. Western blot pictures used for quantification of protein in MCF-7 lysates for first and second biological repetitions of IGF1R, Hsp70, Akt and CDK-4 along with β-actin which was used as a standard.

4. NMR with Hsp90β - supporting data

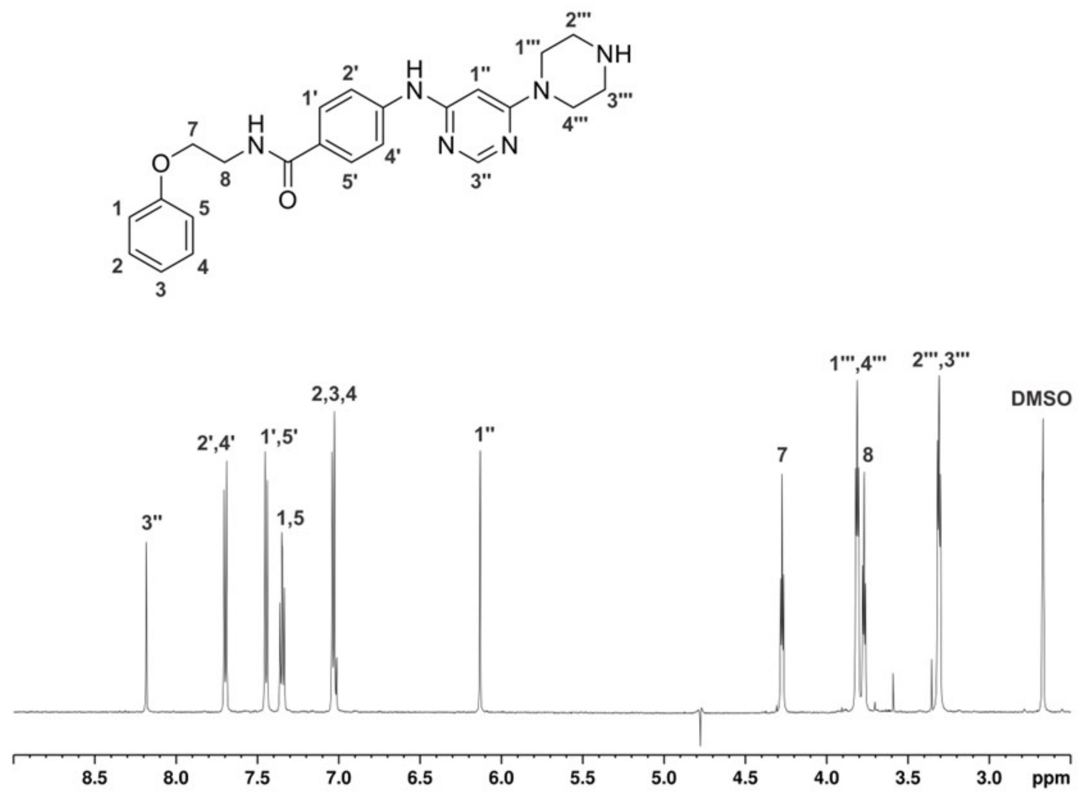

Figure S6. ^{1}H NMR spectrum for the compound **13g** recorded at a concentration of 0.3 mM in 50 mM K-phosphate buffer (pD 7.5), 100 mM KCl, 2 % DMSO-d_6 in D_2O. The proton signals were calibrated to the DSS signal at 0.0 ppm.

Table S2. Chemical shifts in ppm of the assigned protons of compound **13g** in phosphate buffer (pD 7.5); referenced to DSS.

Proton	^{1}H chemical shift [ppm]
3''	8.172
2', 4'	7.697
1', 5'	7.456
1, 5	7.334
2, 3, 4	7.020
1''	6.144
7	4.279
1''', 4'''	3.831
8	3.759
2''', 3'''	3.294

Table S3. STD amplification factors (AMP) and their absolute and relative errors of the assigned protons of compound **13g**.

Proton	AMP	AMP absolute error	AMP relative error
3''	4.88	0.09	1.9
2', 4'	1.78	0.03	1.4
1', 5'	1.75	0.02	1.4
1, 5	2.18	0.03	1.2
2,3,4	2.14	0.02	0.8
1''	0.83	0.03	3.5

Table S4. NOE connectivities of the compound **13g** with calculated distances.

NOE connectivity	Distance [Å]	
	2 NOE interactions	4 NOE interactions
3'' - (1''', 4''')	3.7	/
(1', 5') - 8	/	3.7
(1, 5) - 8	/	4.6
1'' - (1''', 4''')	/	2.9
(2', 4') - 1''	3.9	/

5. Representative HPLC traces of 8c and 13g

HPLC trace of **8c**, UHPLC: t_r: 3.02 min (100% at 254 nm).

Chromatogram

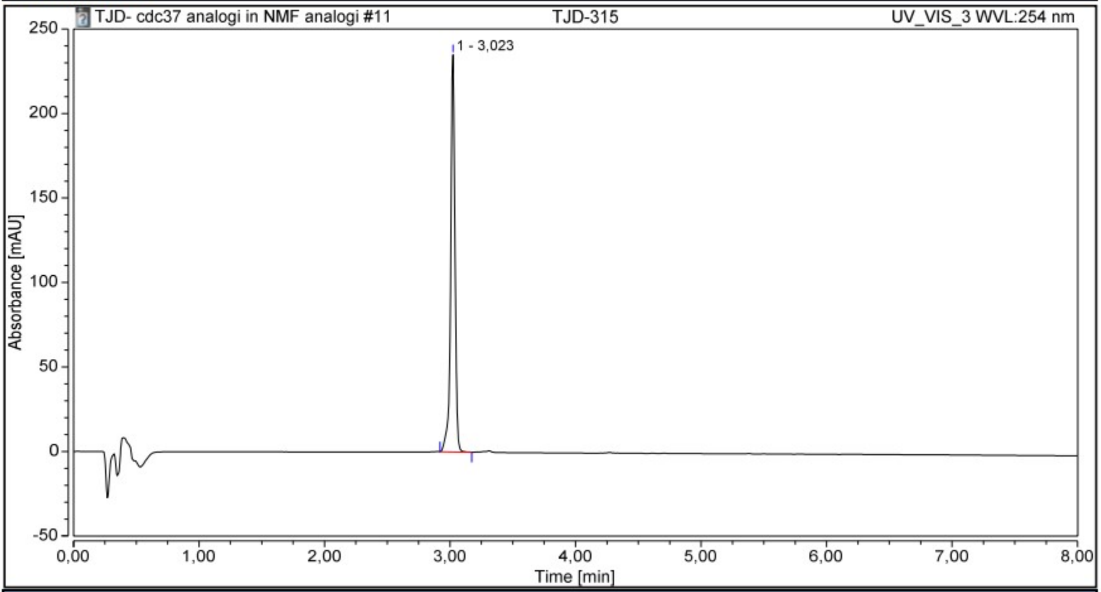

Integration Results

No.	Peak Name	Retention Time min	Area mAU*min	Height mAU	Relative Area %	Relative Height %	Amount n.a.
1		3,023	9,323	235,017	100,00	100,00	n.a.
**Total:**			**9,323**	**235,017**	**100,00**	**100,00**	

HPLC trace of **13g**, UHPLC: t_r: 2.82 min (99.1% at 254 nm).

Chromatogram

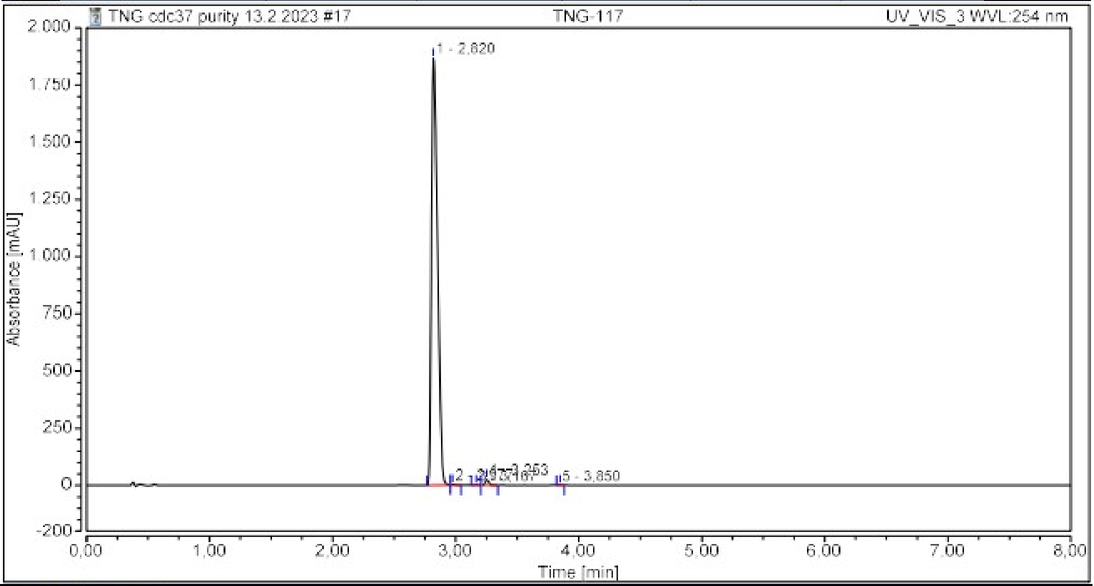

Integration Results

No.	Peak Name	Retention Time min	Area mAU*min	Height mAU	Relative Area %	Relative Height %	Amount n.a.
1		2,820	107,862	1865,879	99,05	98,38	n.a.
2		2,977	0,109	2,315	0,10	0,12	n.a.
3		3,167	0,050	1,509	0,05	0,08	n.a.
4		3,253	0,833	25,547	0,76	1,35	n.a.
5		3,850	0,038	1,358	0,04	0,07	n.a.
**Total:**			**108,893**	**1896,608**	**100,00**	**100,00**	

6. NMR spectra

Compound **4a** ^{1}H NMR (400 MHz, DMSO-d_6):

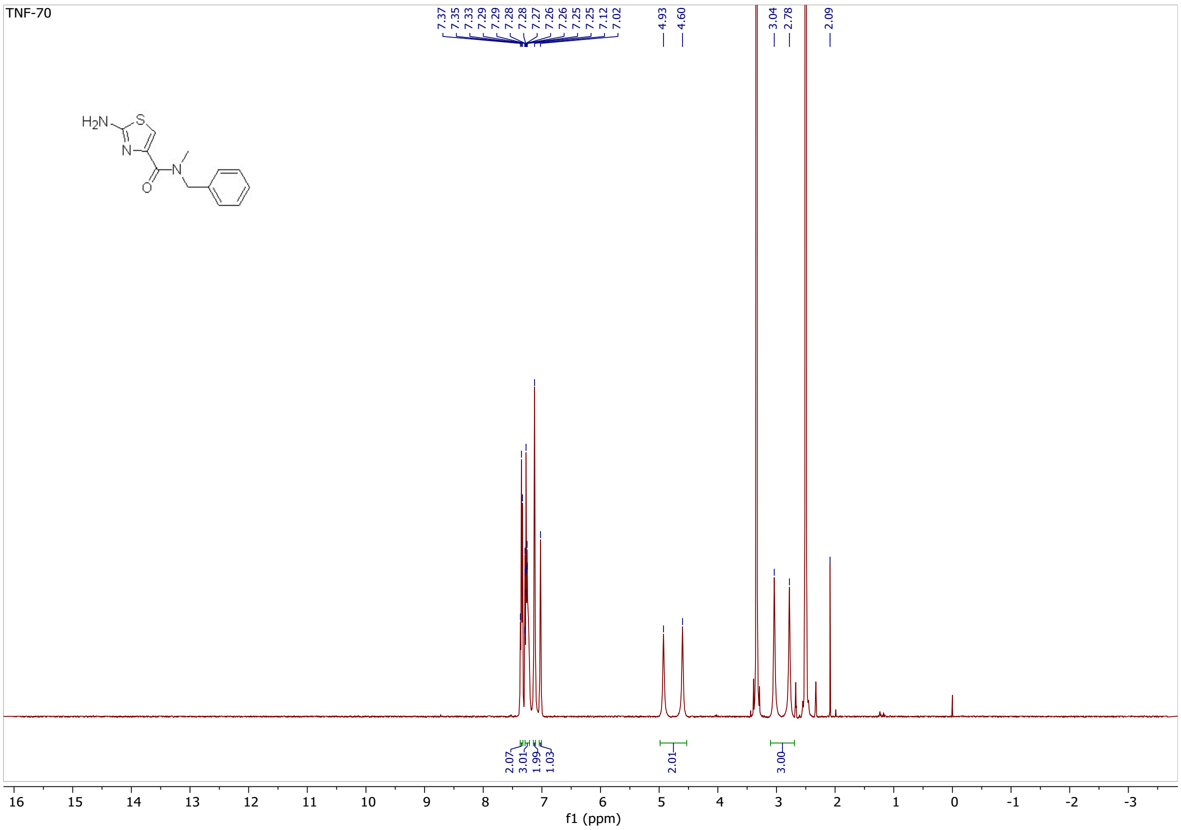

Compound **4a** ^{13}C NMR (101 MHz, DMSO-d_6):

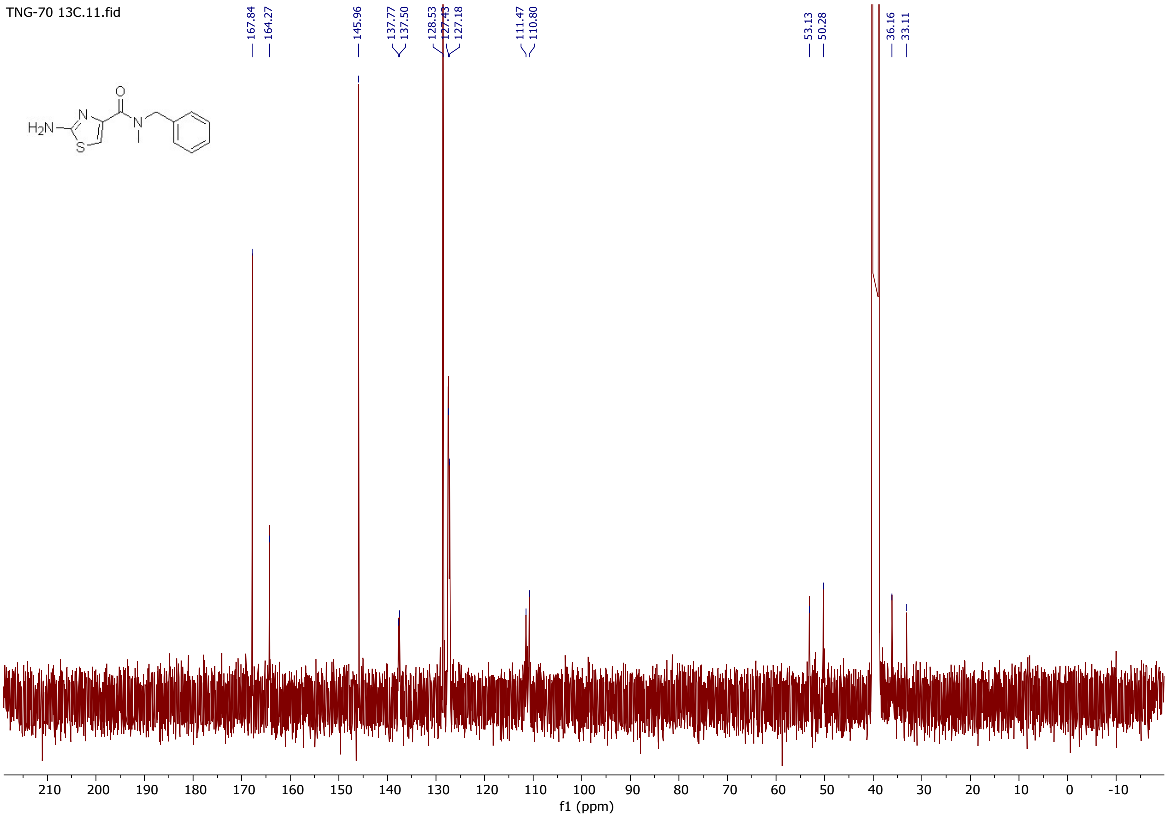

Compound **5a** ^{1}H NMR (400 MHz, DMSO-d_6):

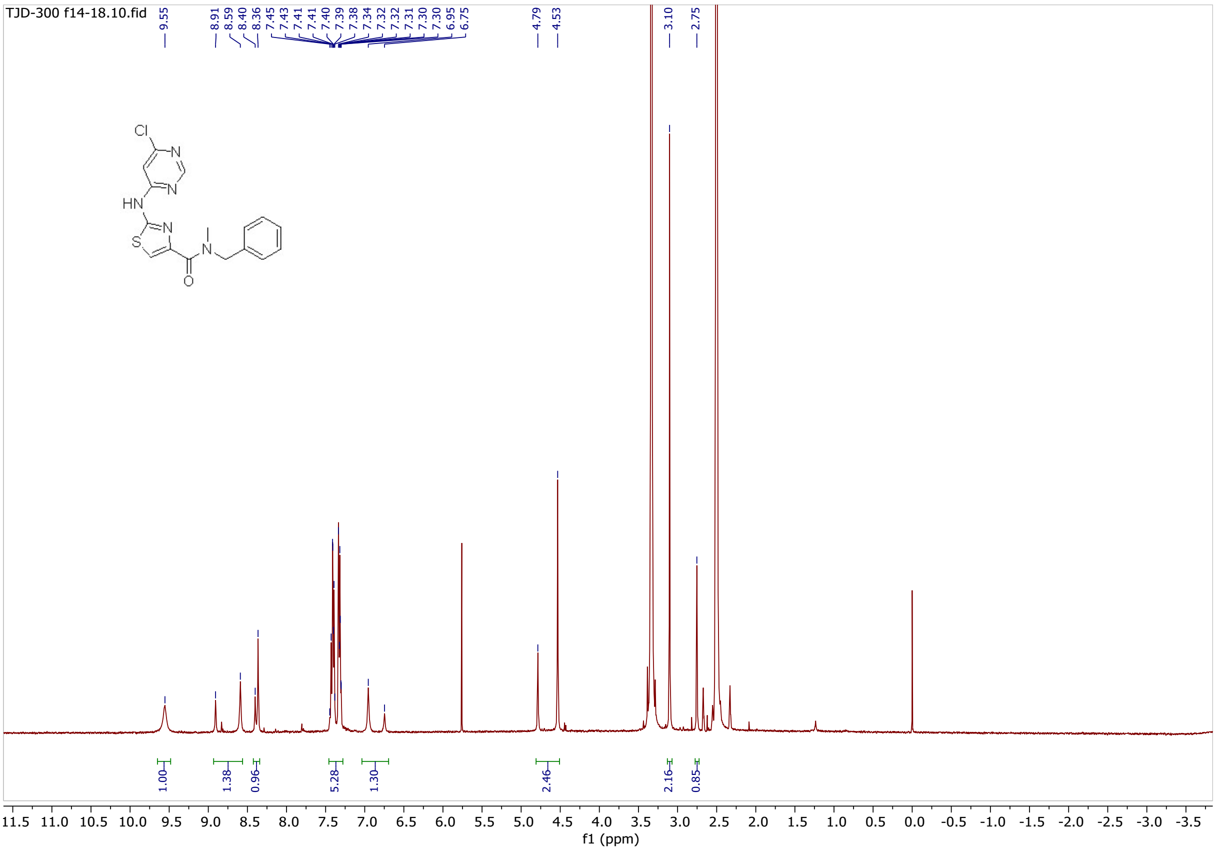

Compound **5a** ^{13}C NMR (101 MHz, DMSO-d_6):

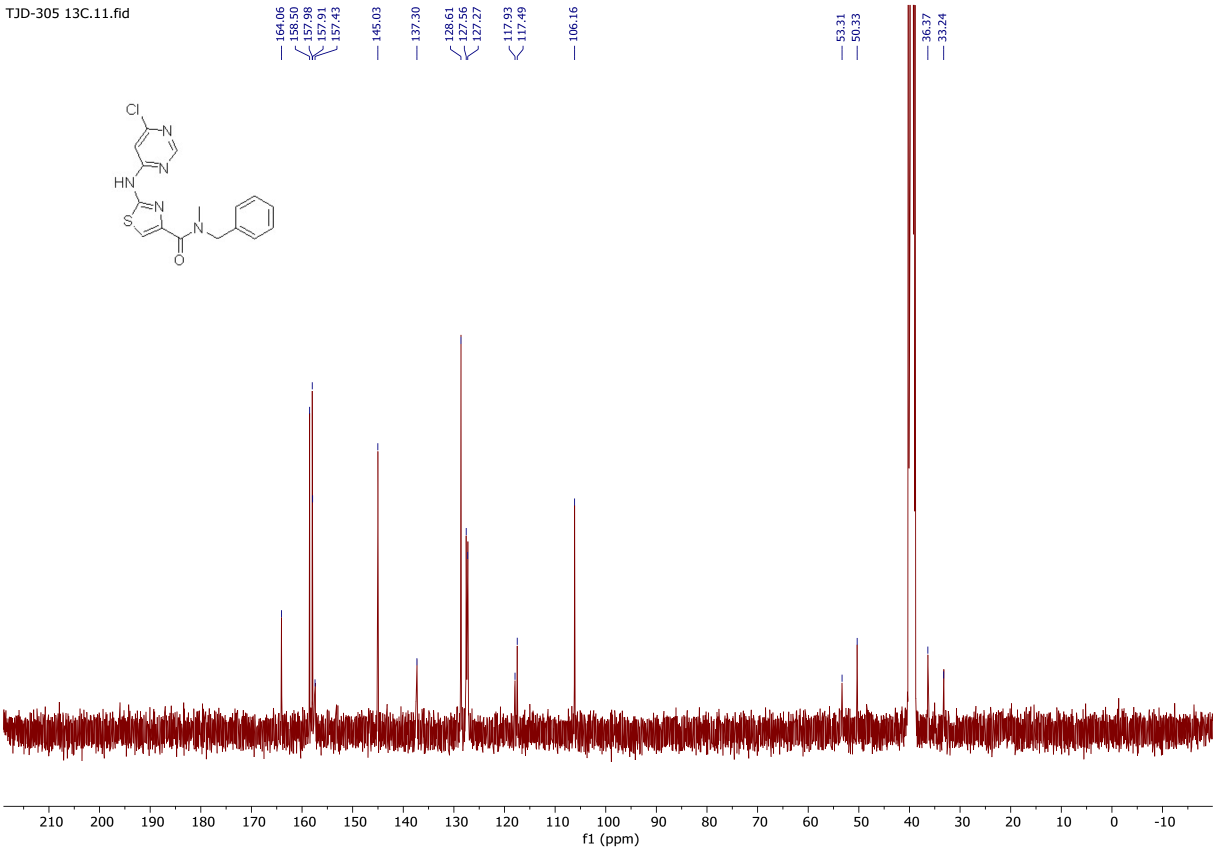

Compound **6a** ^{1}H NMR (400 MHz, DMSO-d_6):

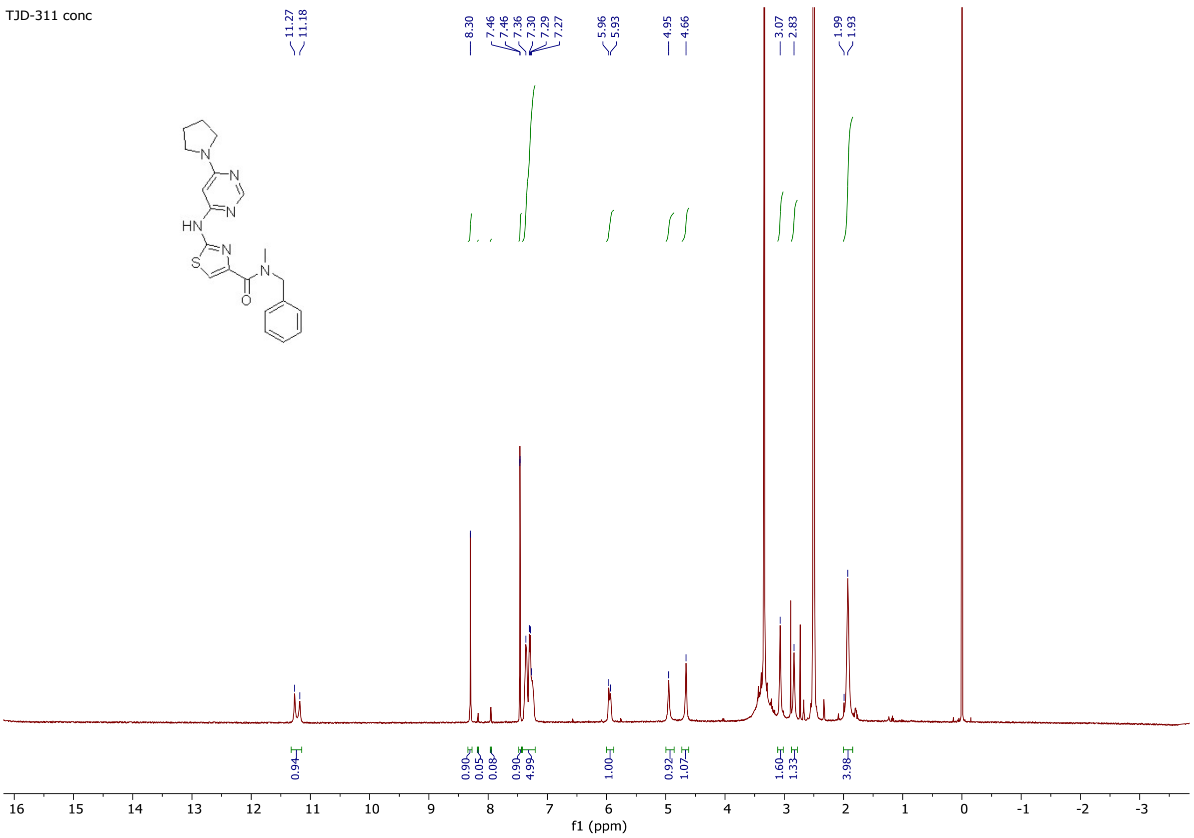

Compound **6a** ^{13}C NMR (101 MHz, DMSO-d_6):

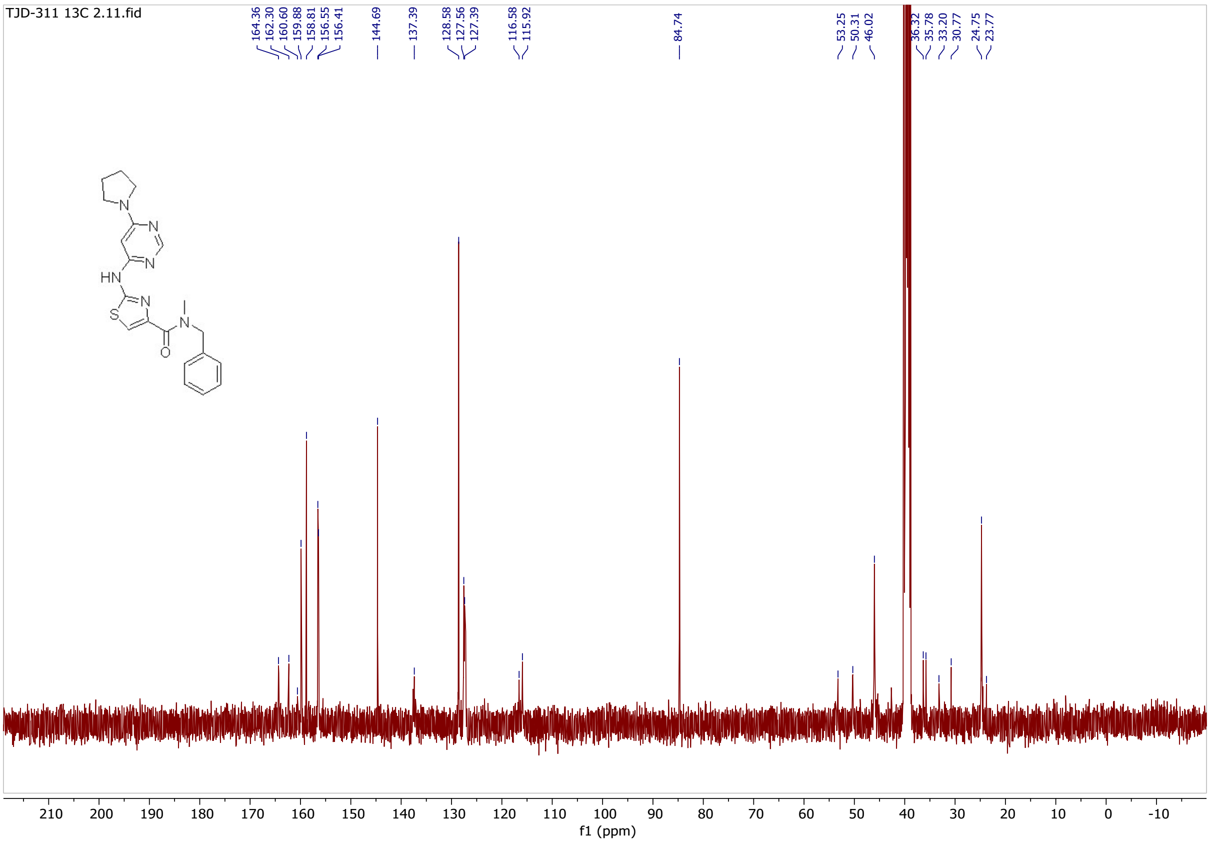

Compound **6b** ^{1}H NMR (400 MHz, DMSO-d_6):

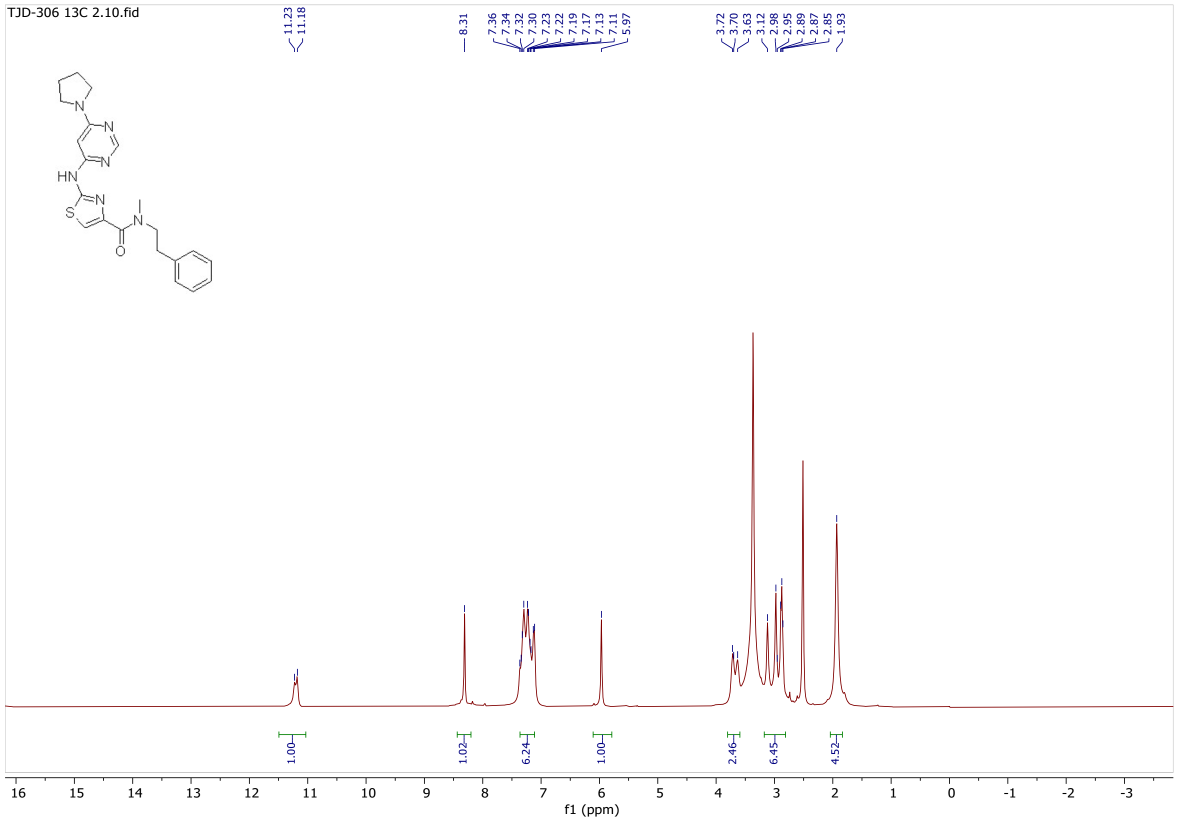

Compound **6b** ^{13}C NMR (101 MHz, DMSO-d_6):

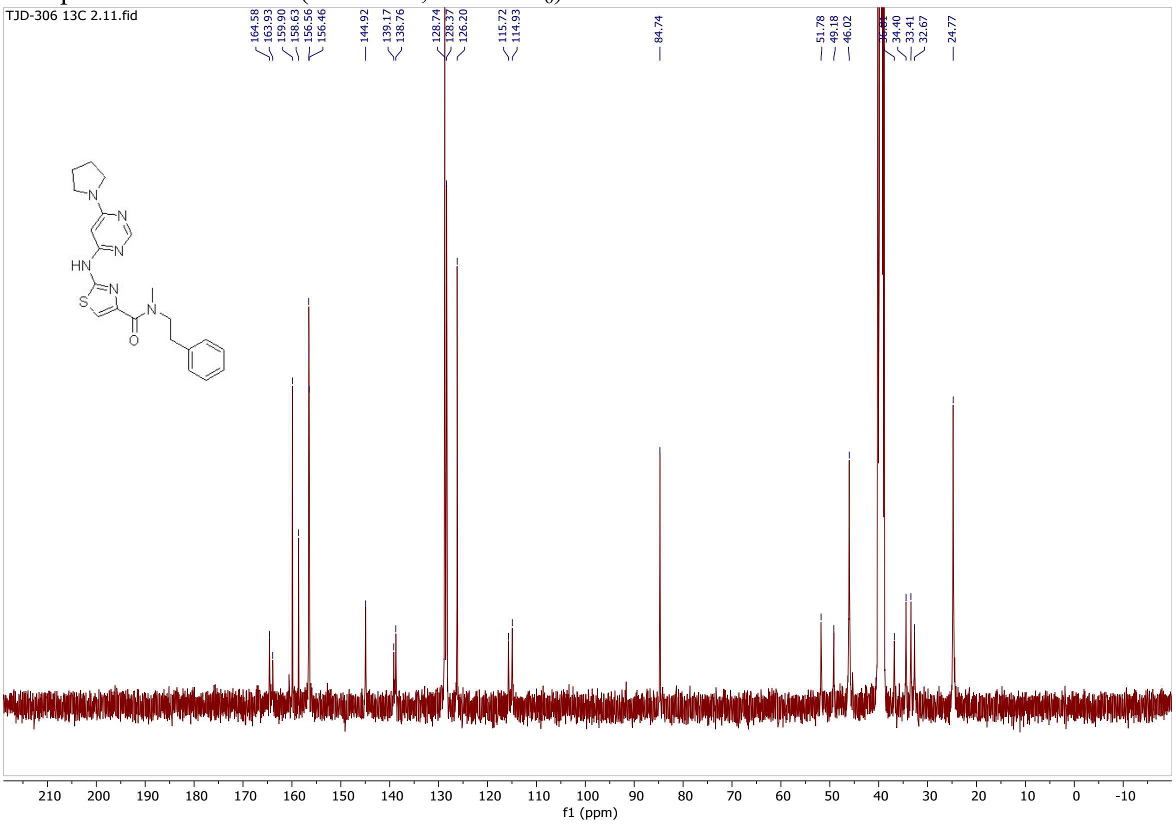

Compound **6c** ^{1}H NMR (400 MHz, DMSO-d_6):

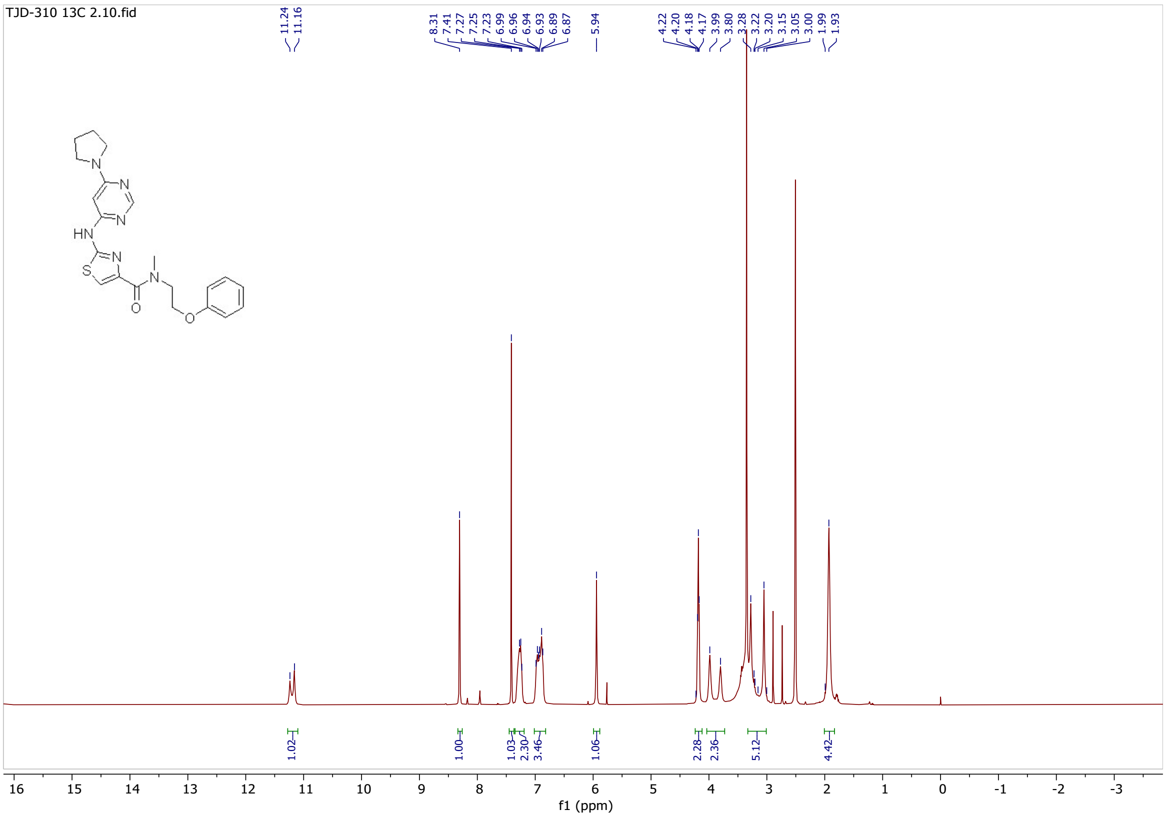

Compound **6c** ^{13}C NMR (101 MHz, DMSO-d_6):

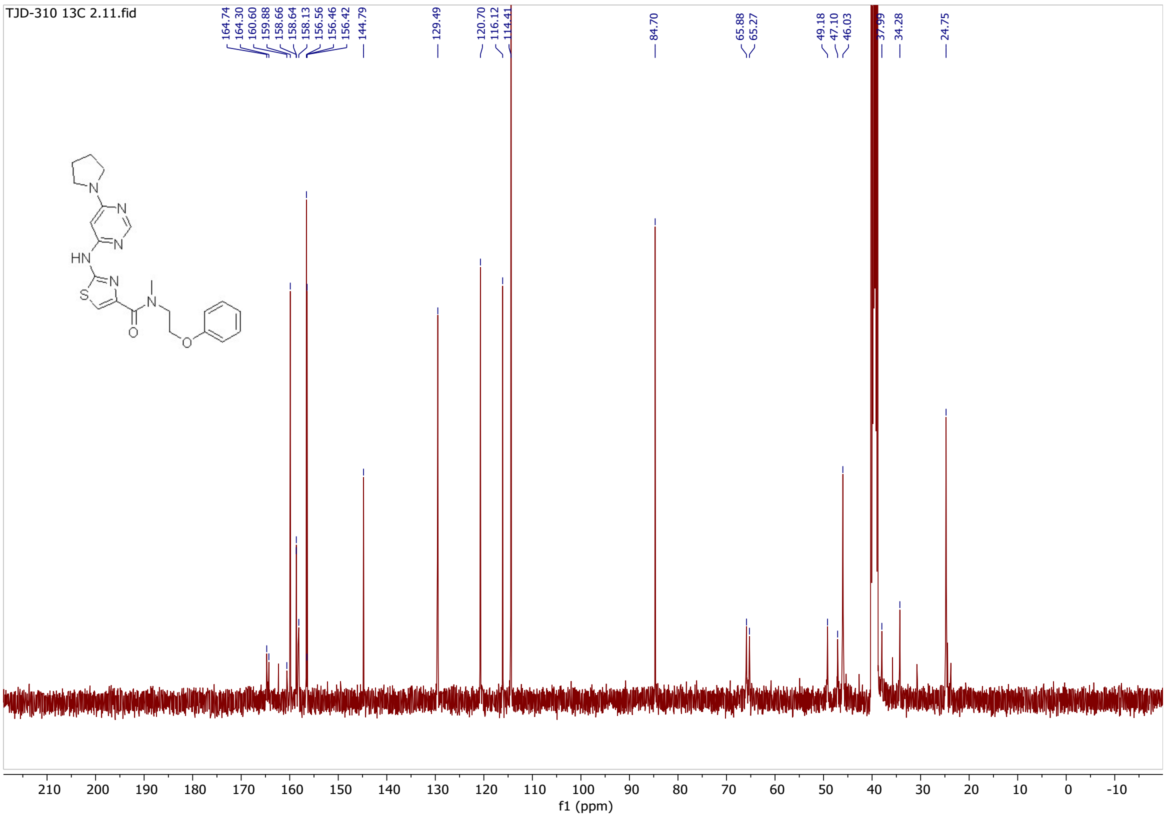

Compound **7c** ^{1}H NMR (400 MHz, DMSO-d_6):

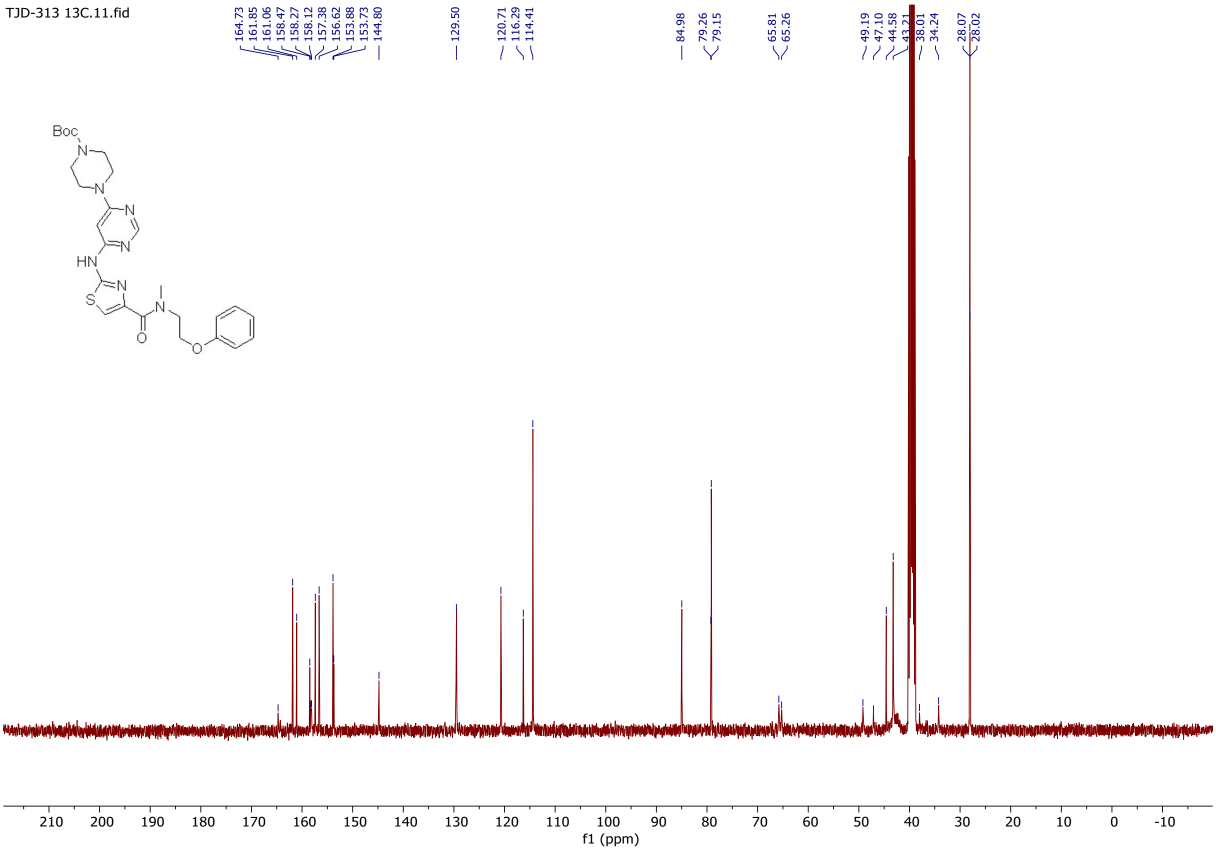

Compound **7c** ^{13}C NMR (101 MHz, DMSO-d_6):

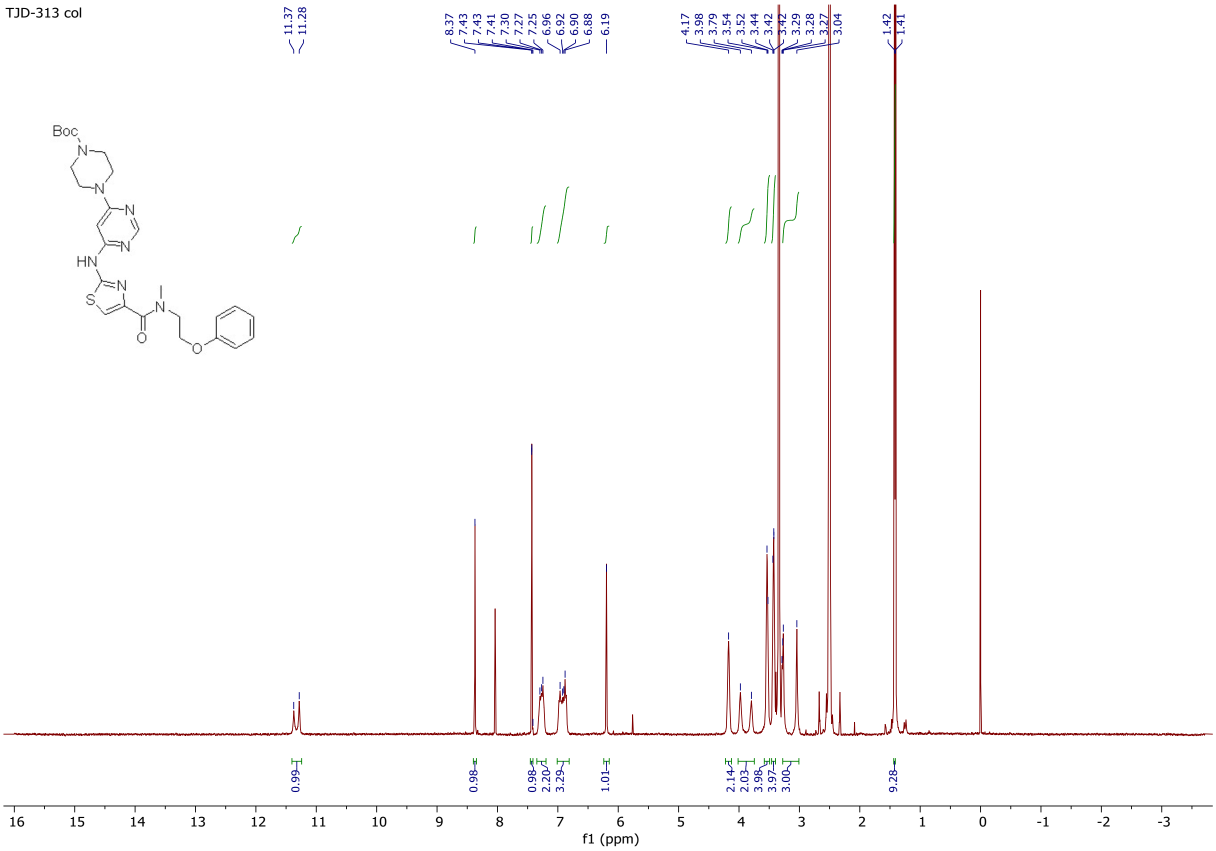

Compound **8a** ^{1}H NMR (400 MHz, DMSO-d_6):

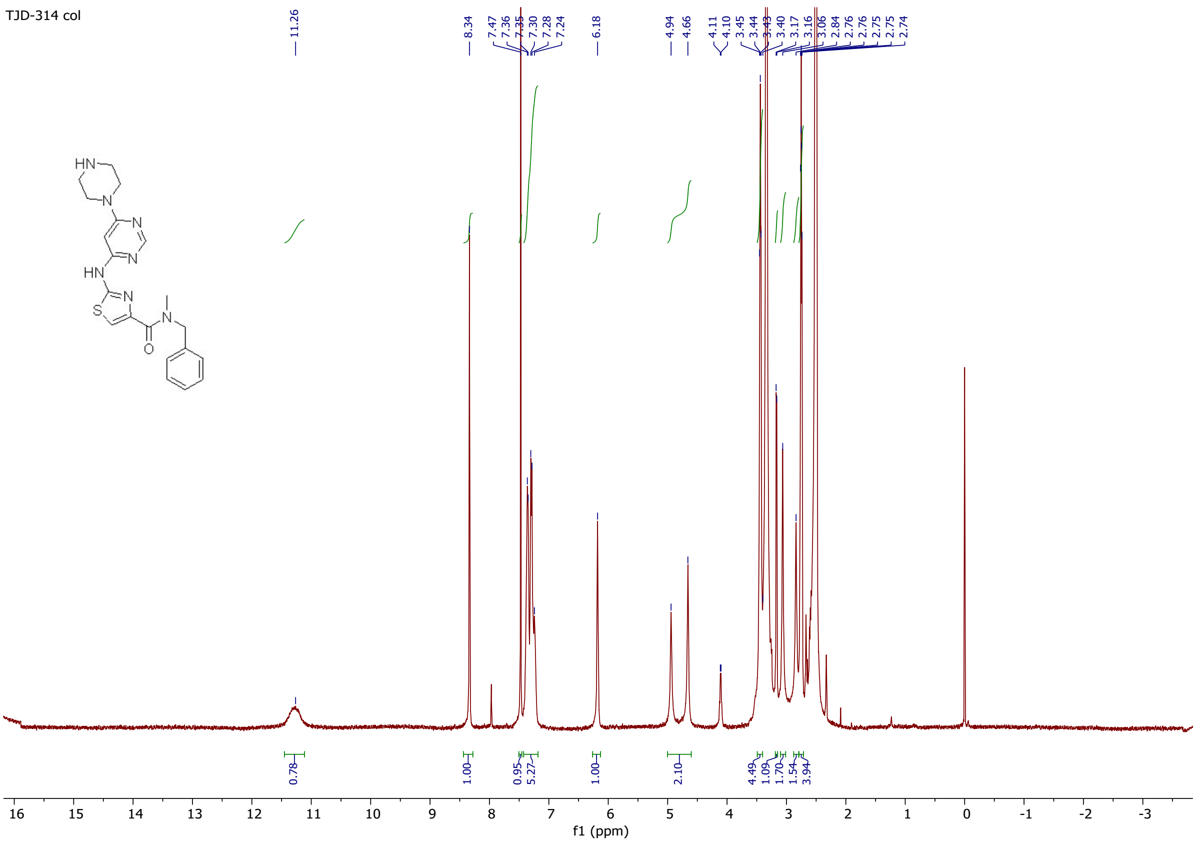

Compound **8a** ^{13}C NMR (101 MHz, DMSO-d_6):

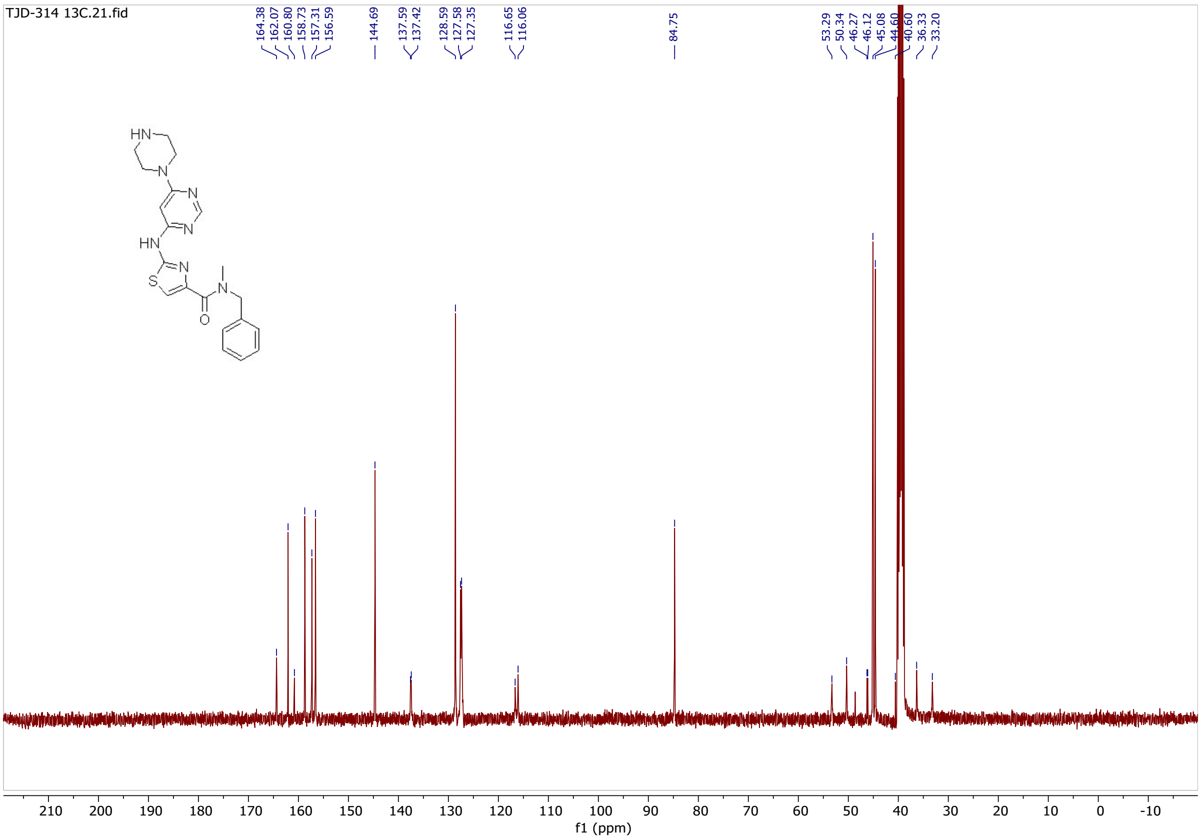

Compound **8b** ^{1}H NMR (400 MHz, DMSO-d_6):

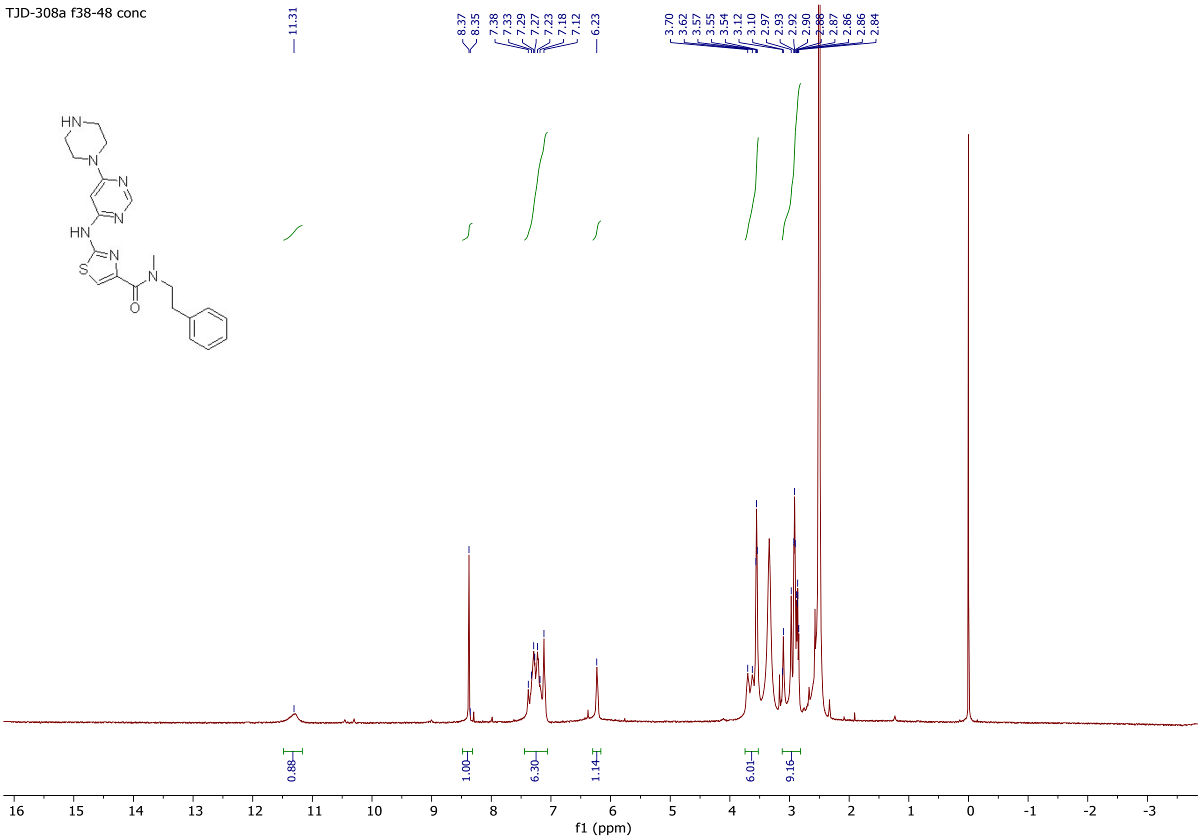

Compound **8b** ^{13}C NMR (101 MHz, DMSO-d_6):

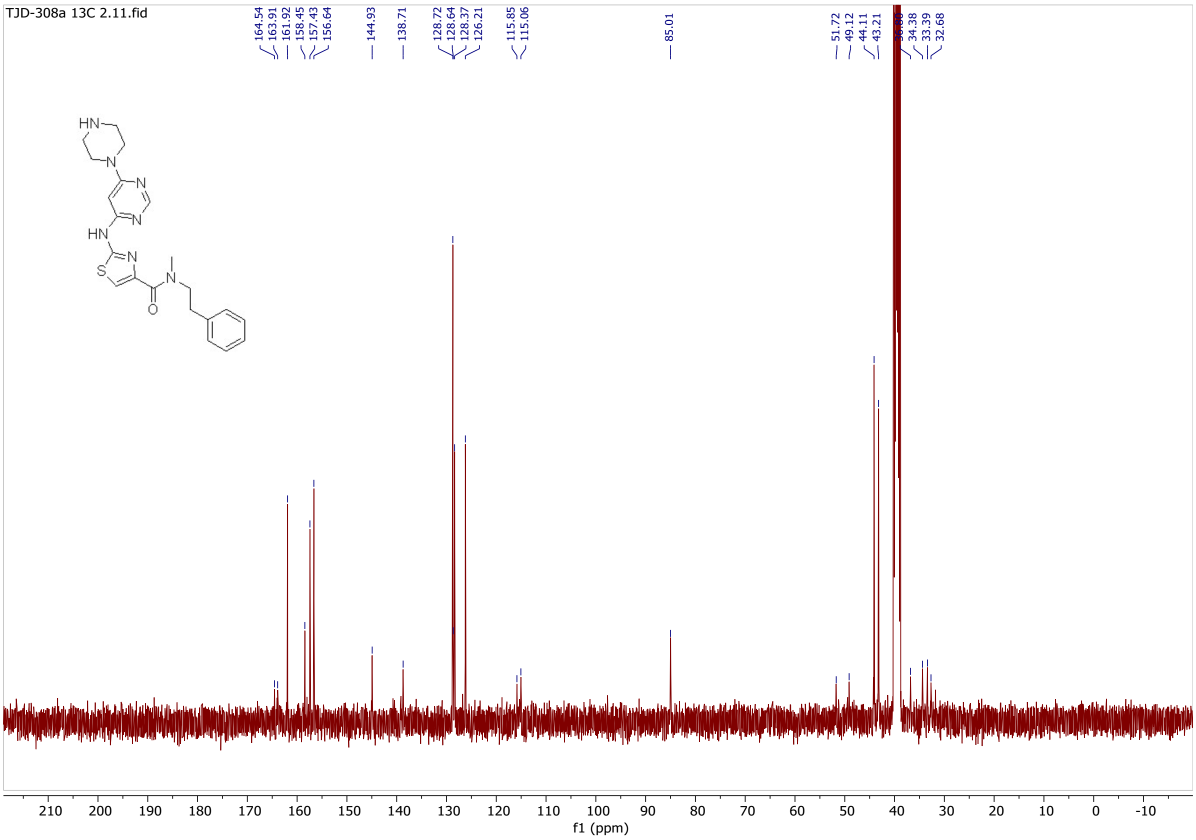

Compound **8c** ^{1}H NMR (400 MHz, DMSO-d_6):

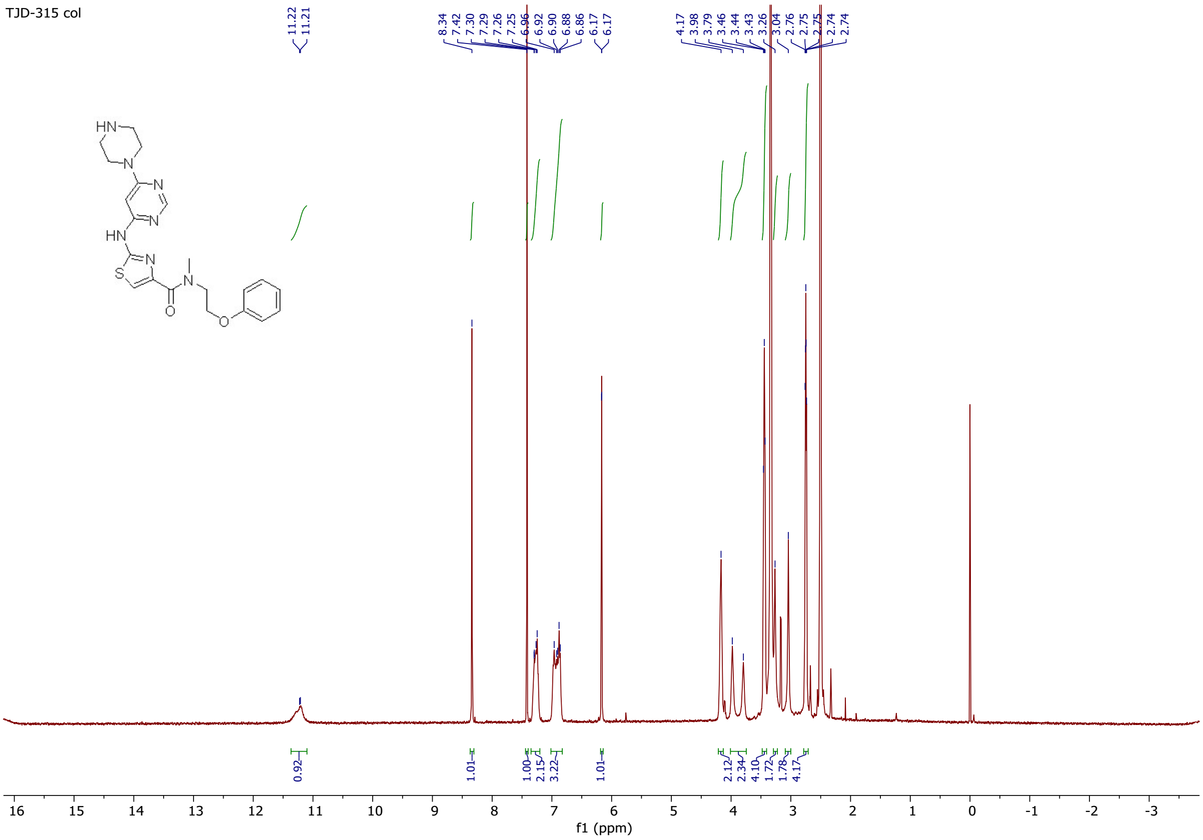

Compound **8c** ^{13}C NMR (101 MHz, DMSO-d_6):

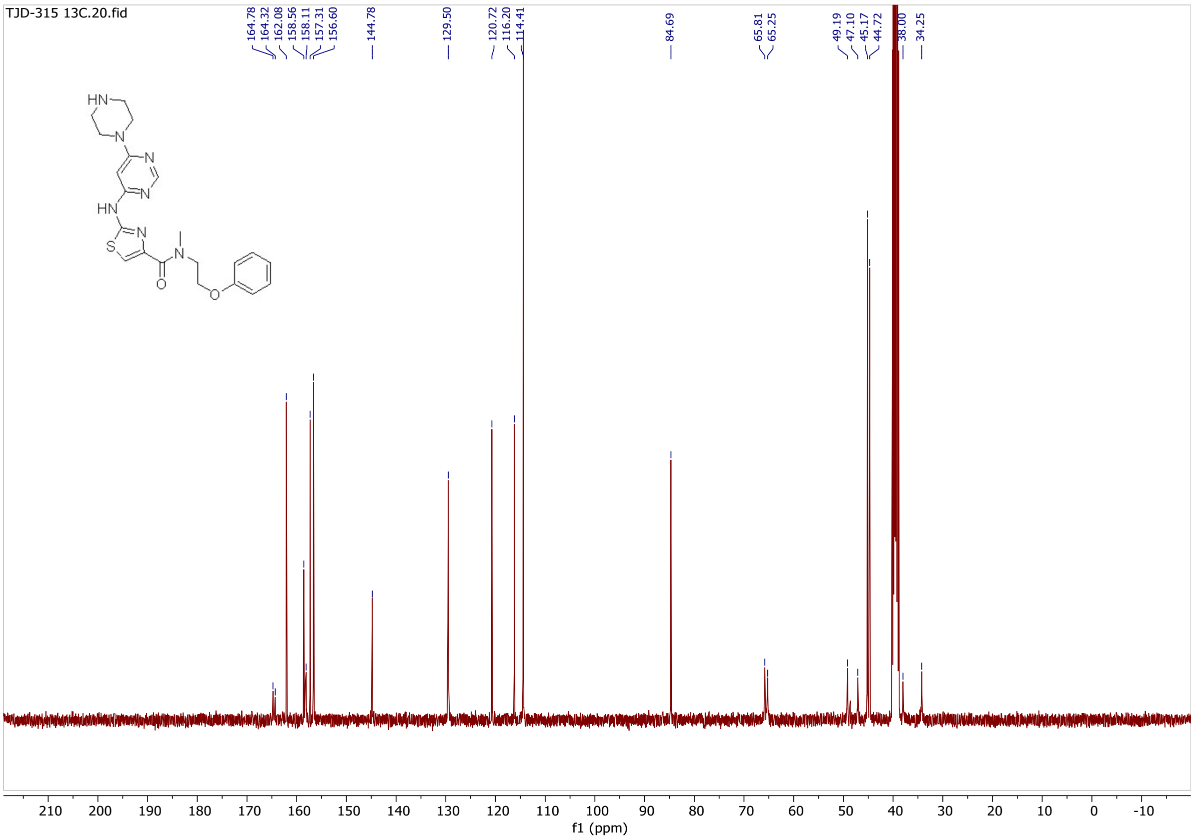

Compound **9g** ^{1}H NMR (400 MHz, DMSO-d_6):

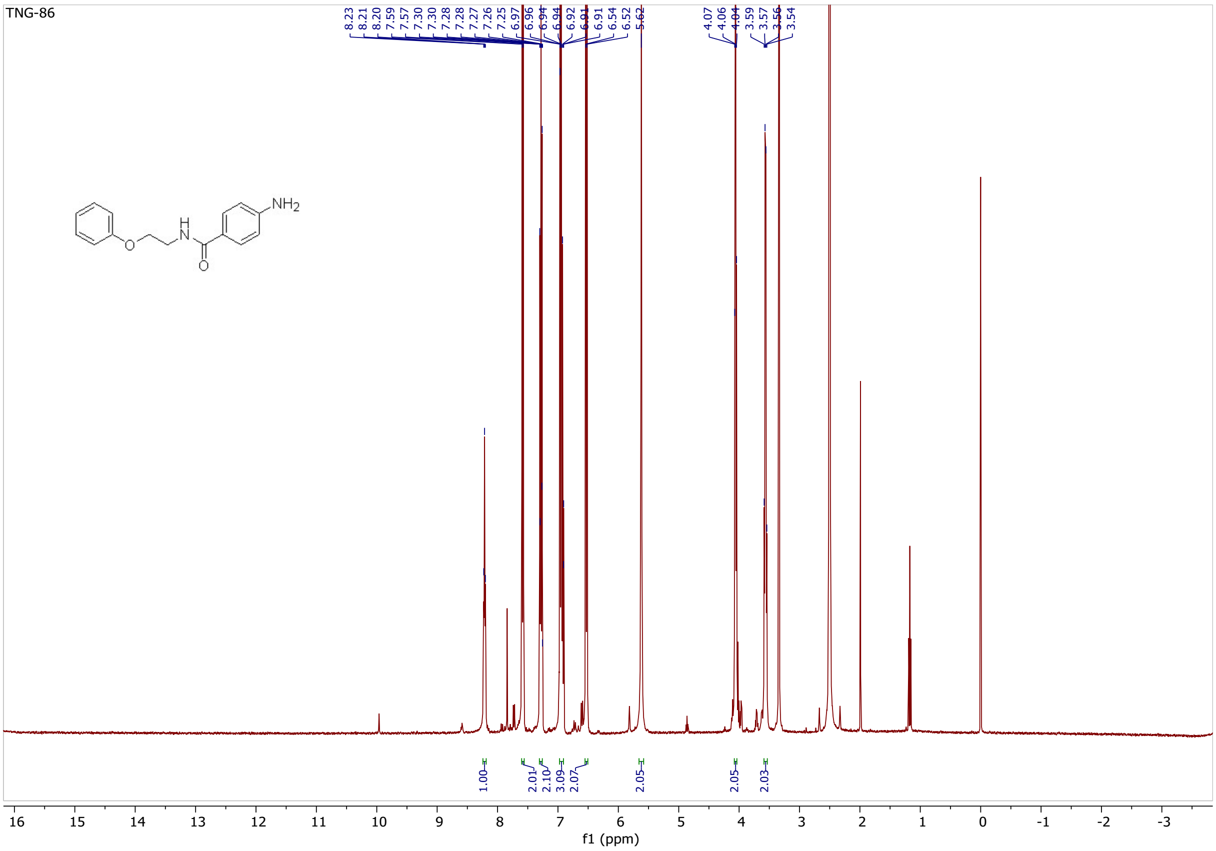

Compound **9g** ^{13}C NMR (101 MHz, DMSO-d_6:

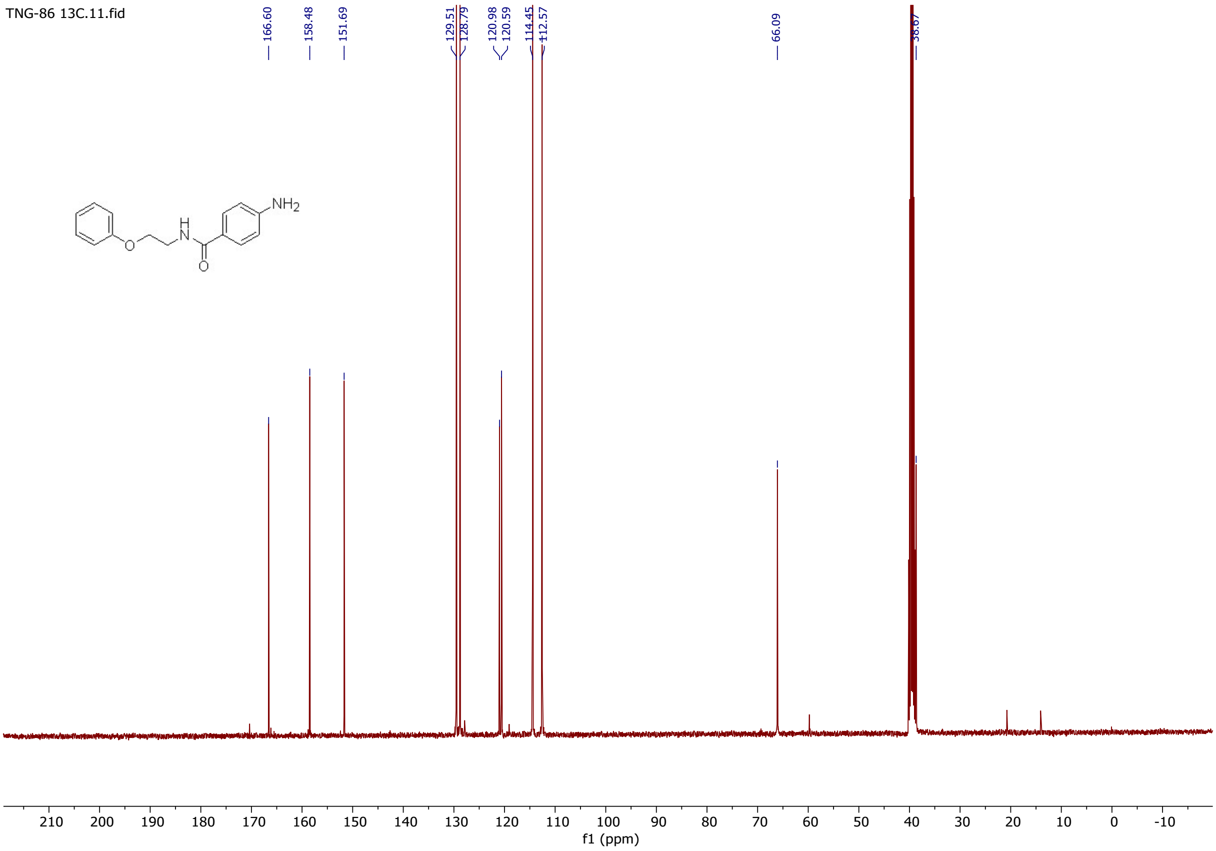

Compound **10g** ^{1}H NMR (400 MHz, DMSO-d_6):

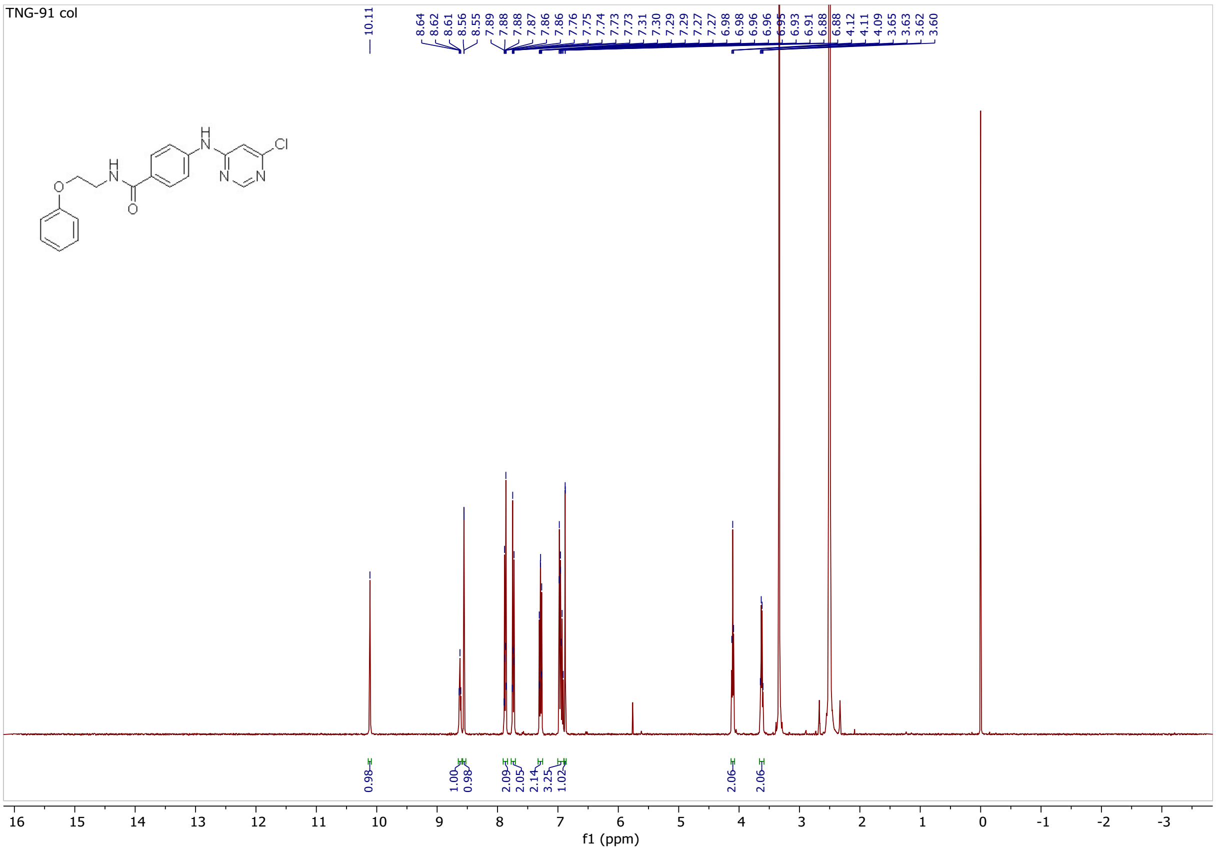

Compound **10g** ^{13}C NMR (101 MHz, DMSO-d_6):

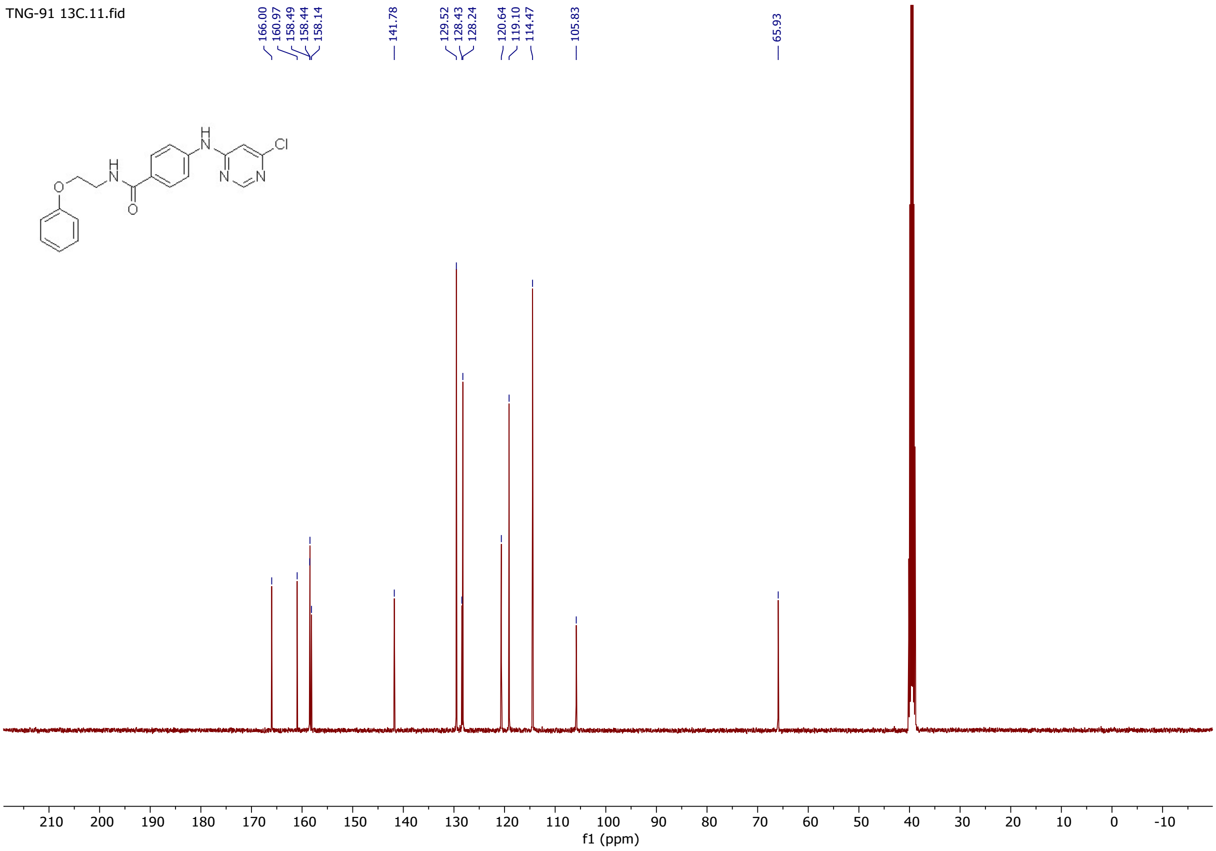

Compound **11a** ^{1}H NMR (400 MHz, DMSO-d_6):

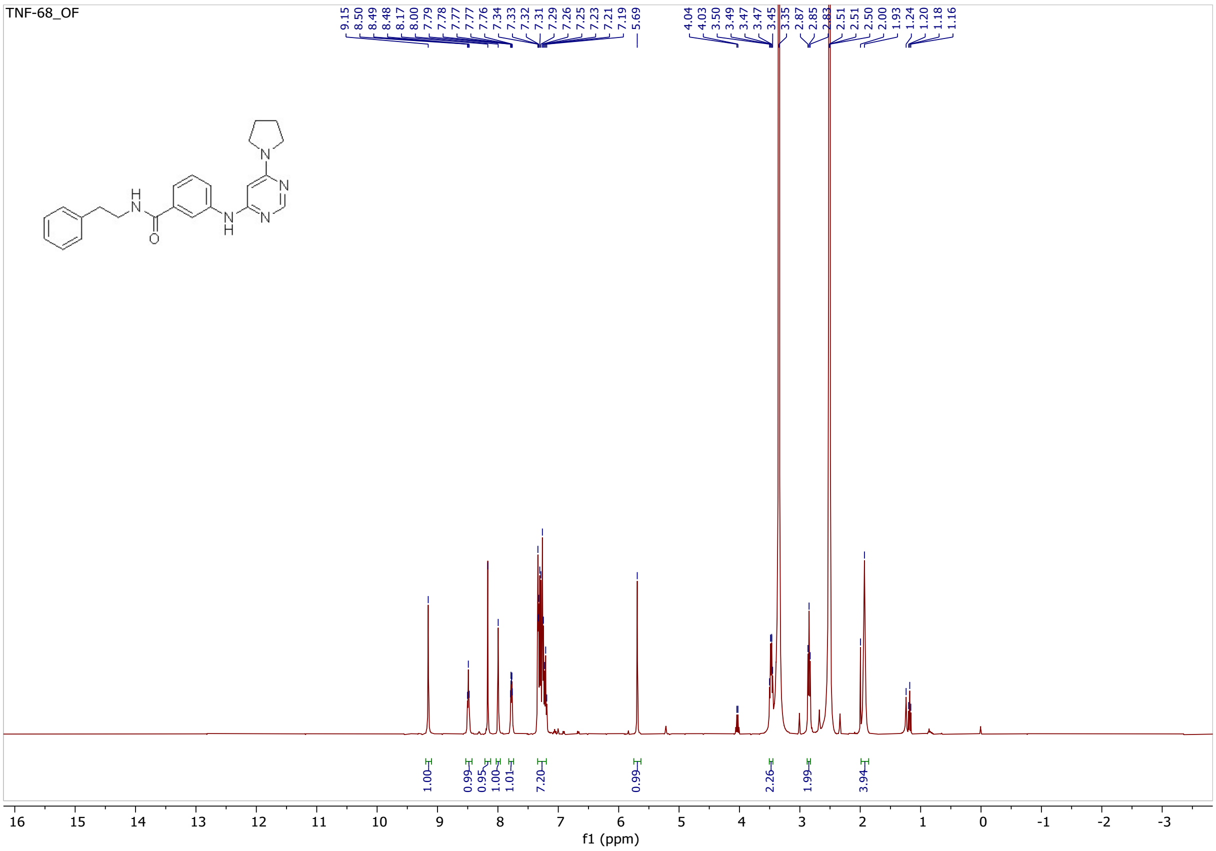

Compound **11a** ^{13}C NMR (101 MHz, DMSO-d_6):

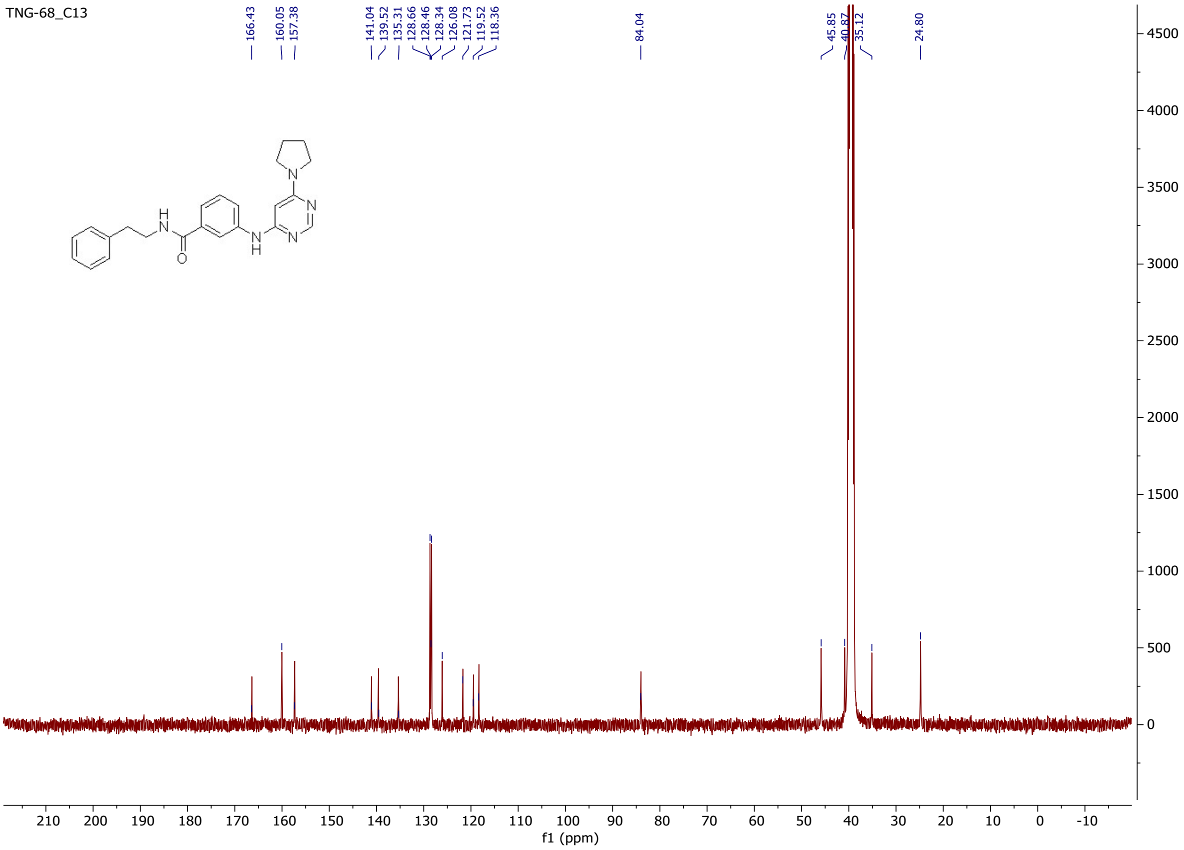

Compound **11b** ^{1}H NMR (400 MHz, DMSO-d_6):

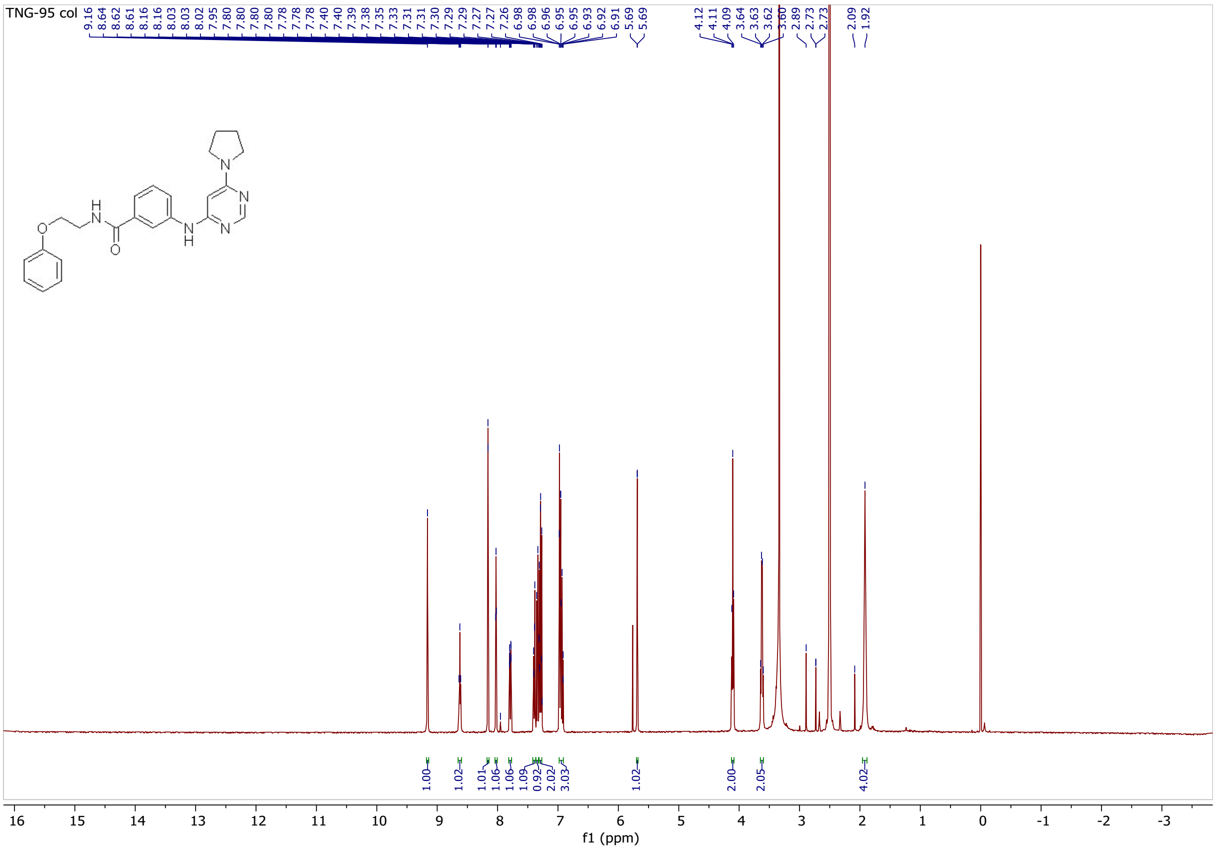

Compound **11b** ^{13}C NMR (101 MHz, DMSO-d_6):

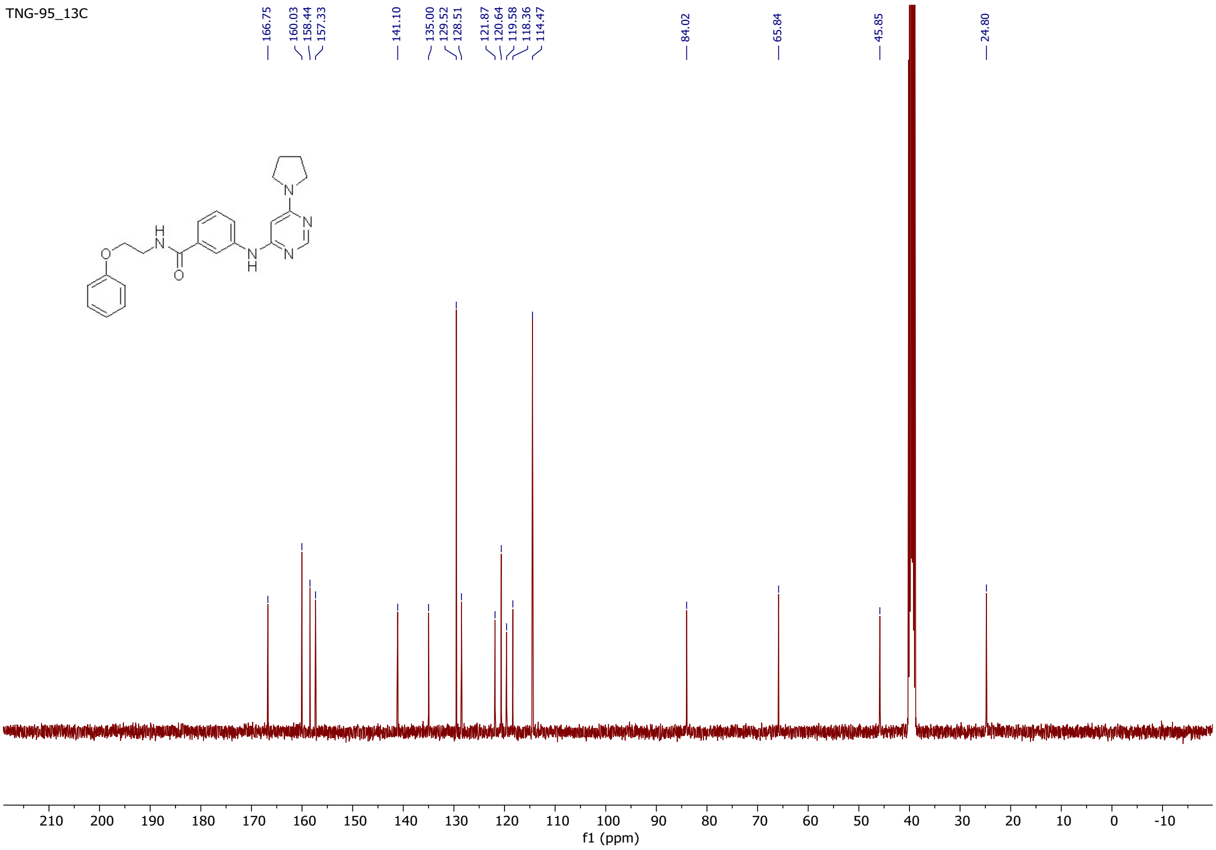

Compound **11c** ^{1}H NMR (400 MHz, DMSO-d_6):

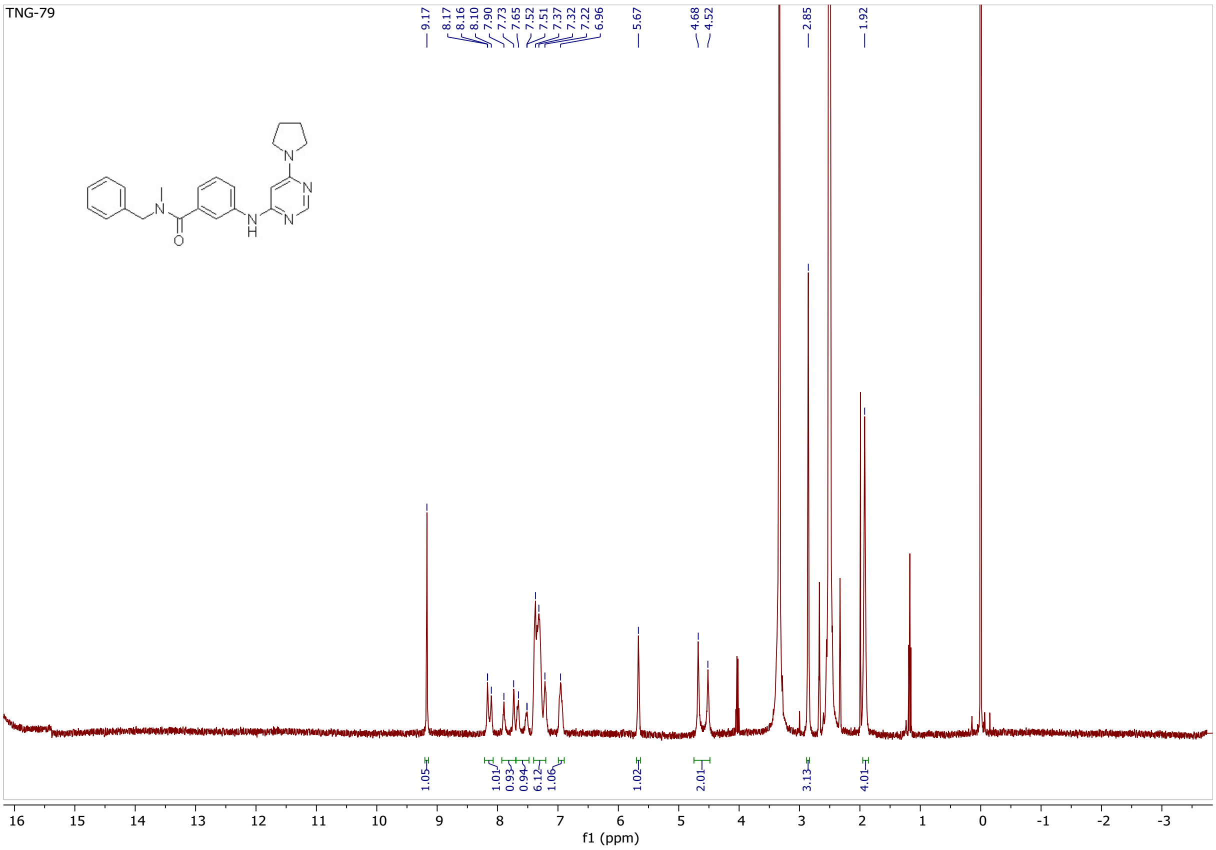

Compound **11c** ^{13}C NMR (101 MHz, DMSO-d_6):

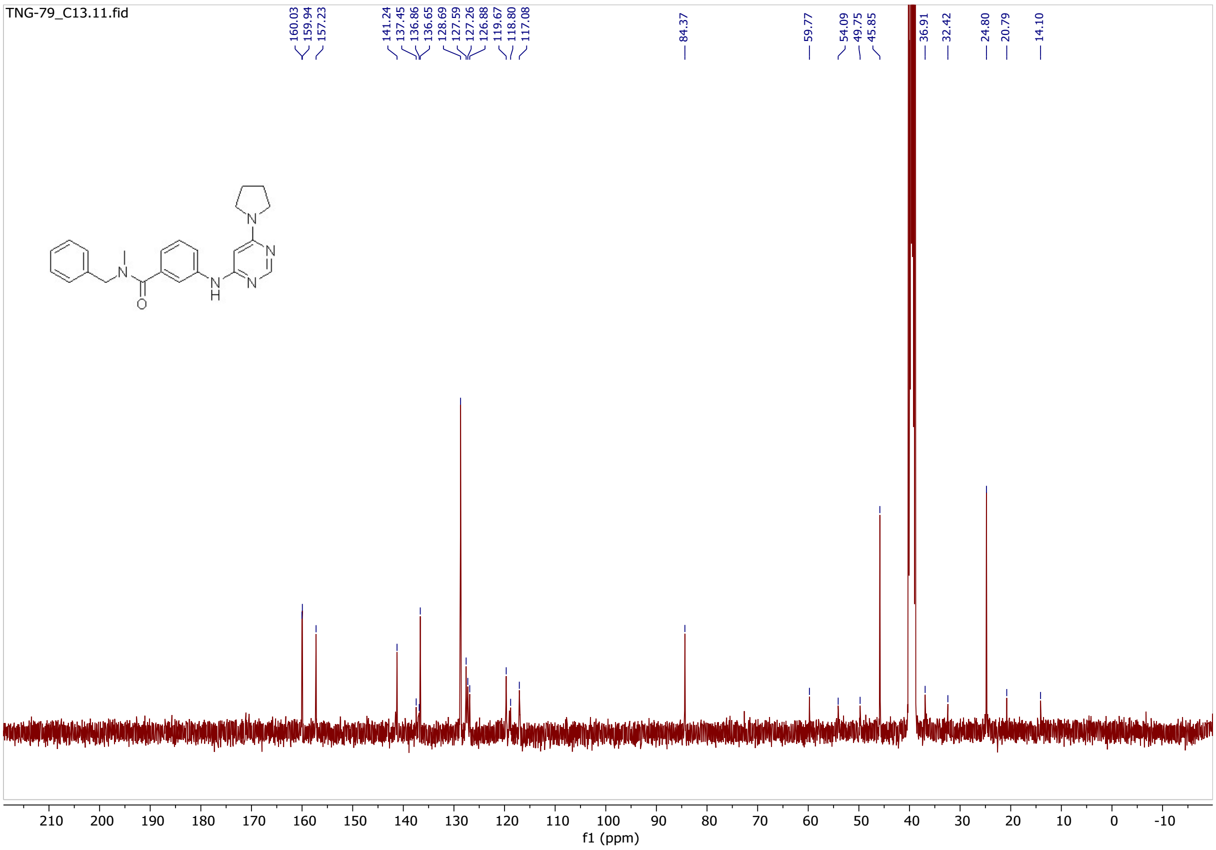

Compound **11d** ^{1}H NMR (400 MHz, DMSO-d_6):

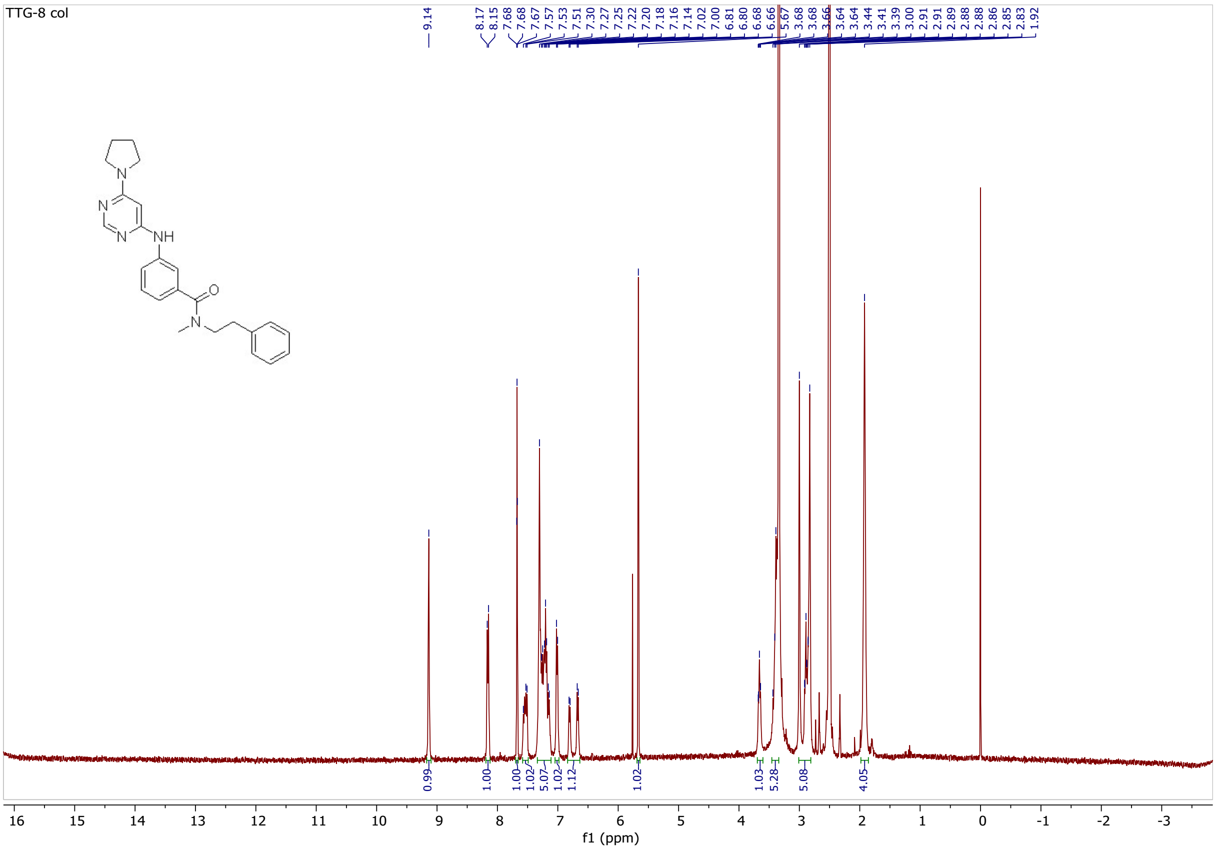

Compound **11d** ^{13}C NMR (101 MHz, DMSO-d_6):

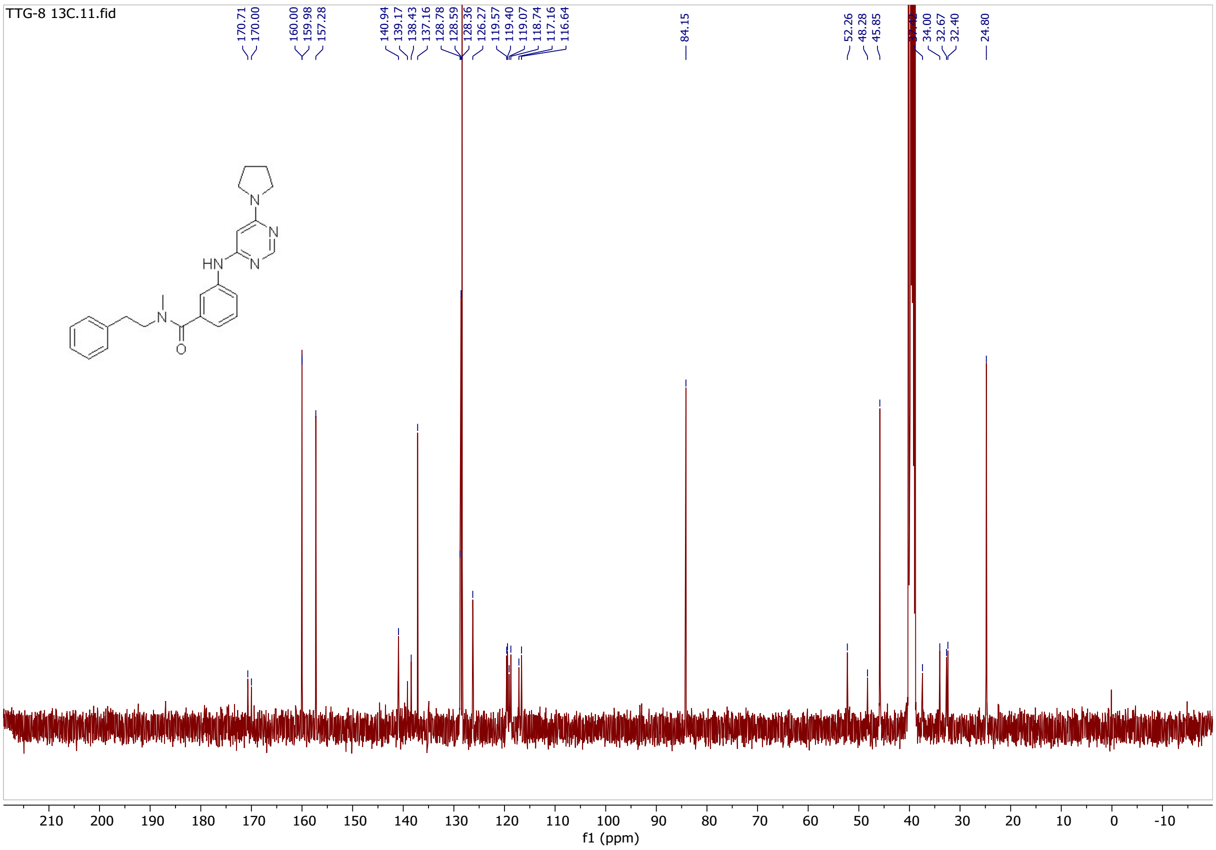

Compound **11e** ^{1}H NMR (400 MHz, DMSO-d_6):

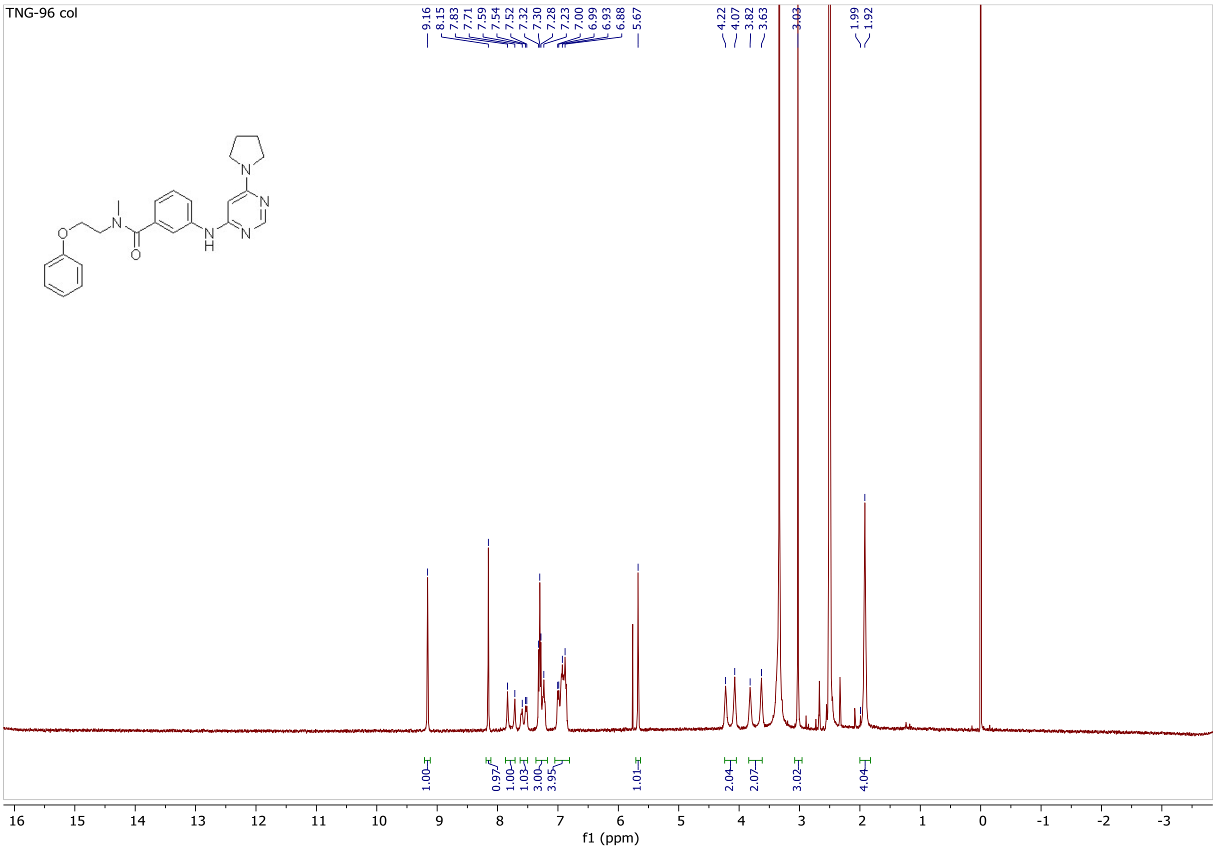

Compound **11e** ^{13}C NMR (101 MHz, DMSO-d_6):

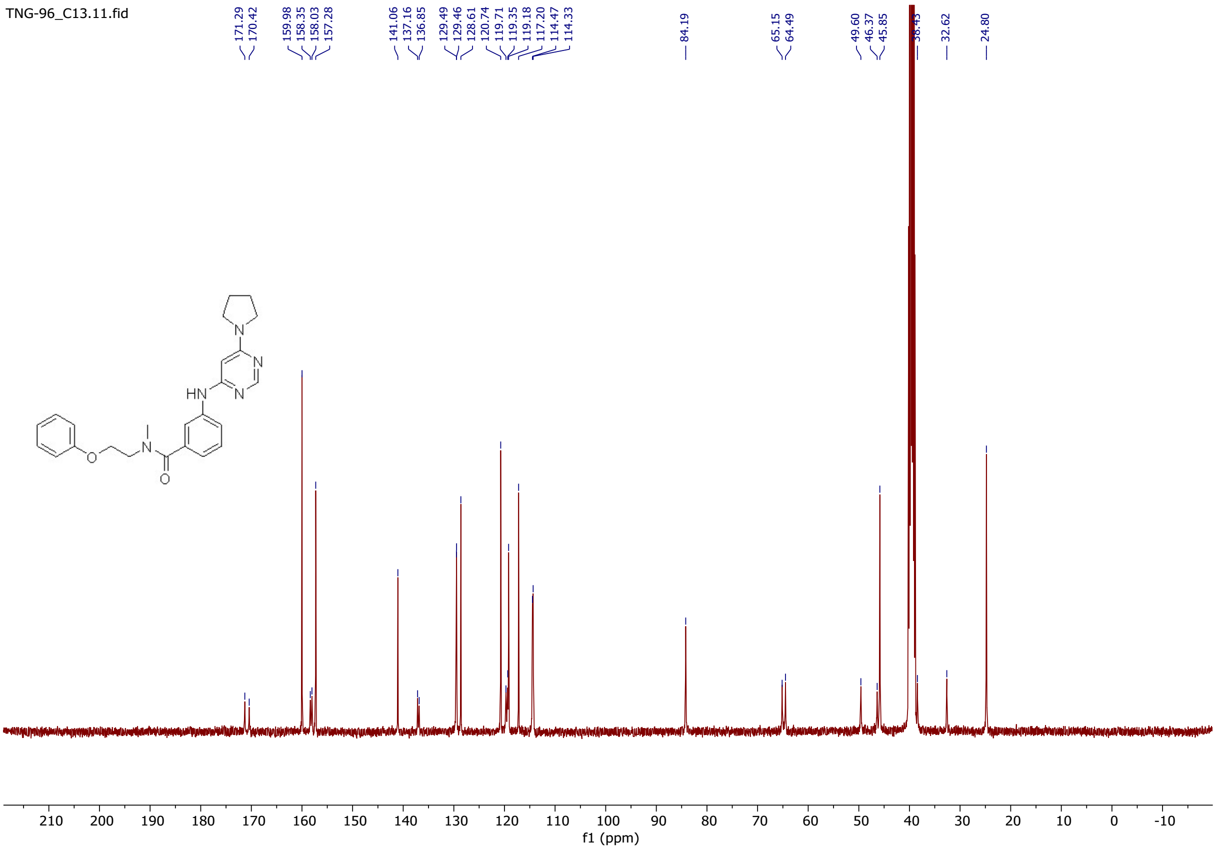

Compound **11f** ^{1}H NMR (400 MHz, DMSO-d_6):

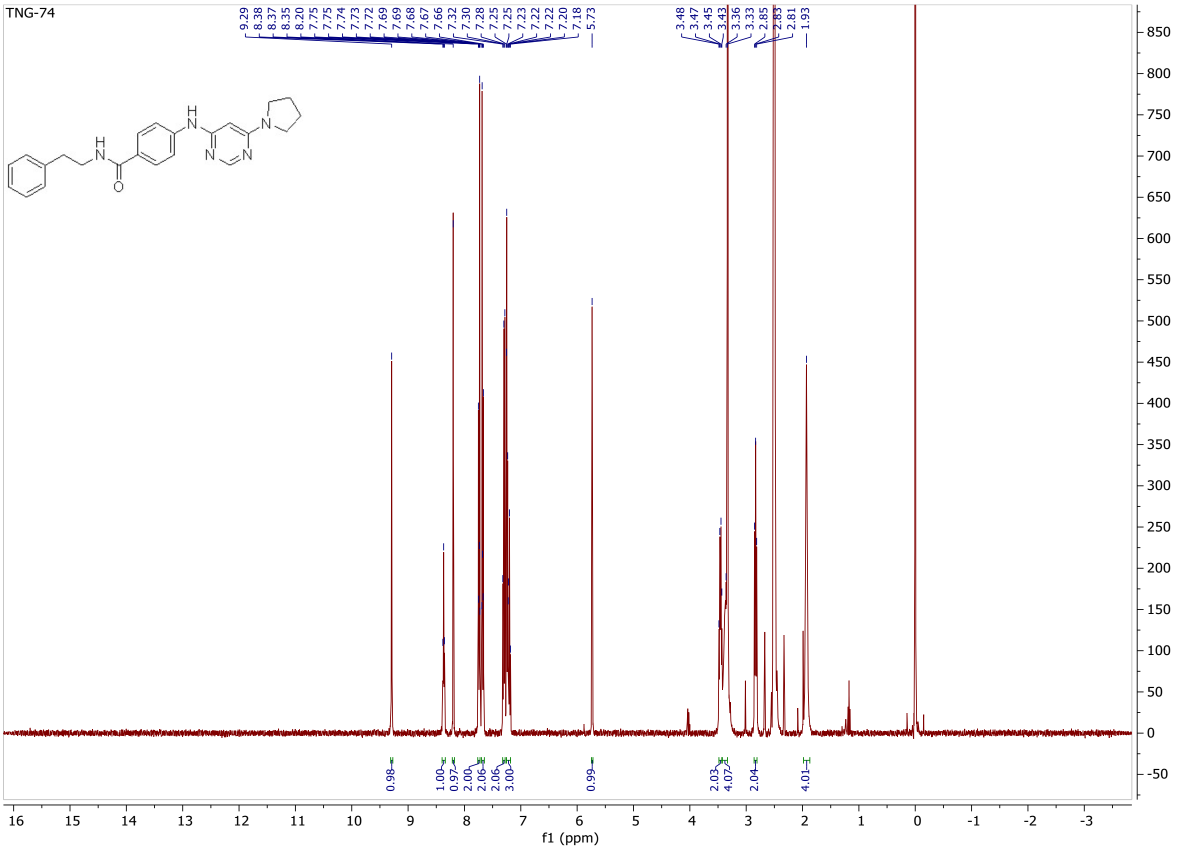

Compound **11f** ^{13}C NMR (101 MHz, DMSO-d_6):

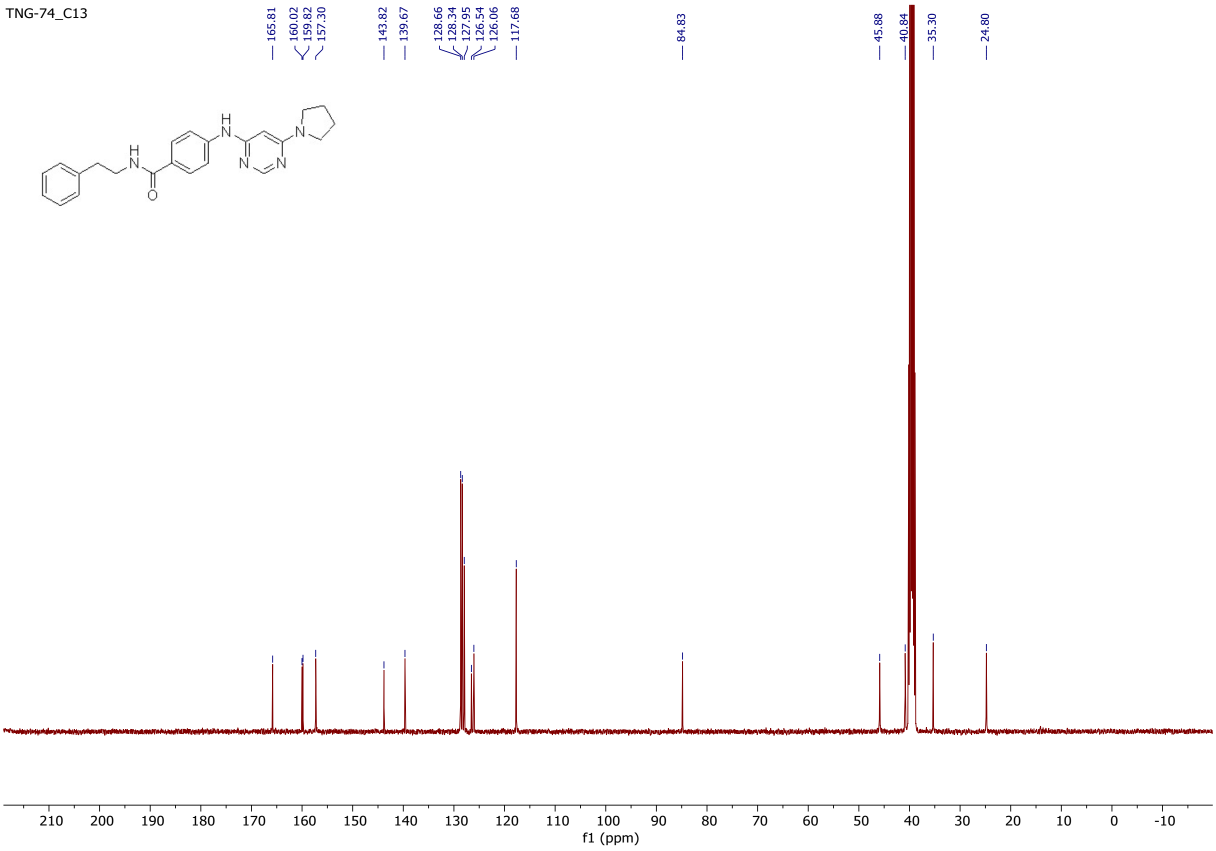

Compound **11g** ^{1}H NMR (400 MHz, DMSO-d_6):

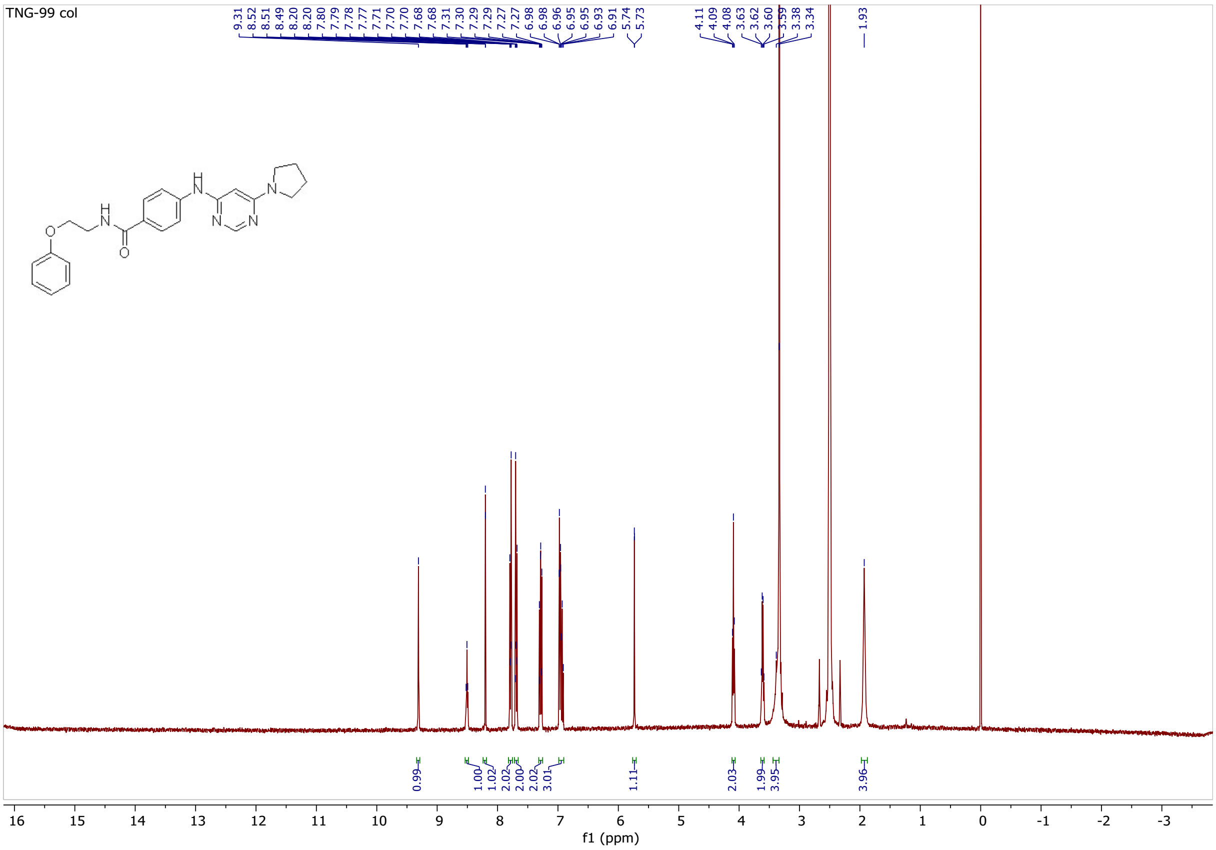

Compound **11g** ^{13}C NMR (101 MHz, DMSO-d_6):

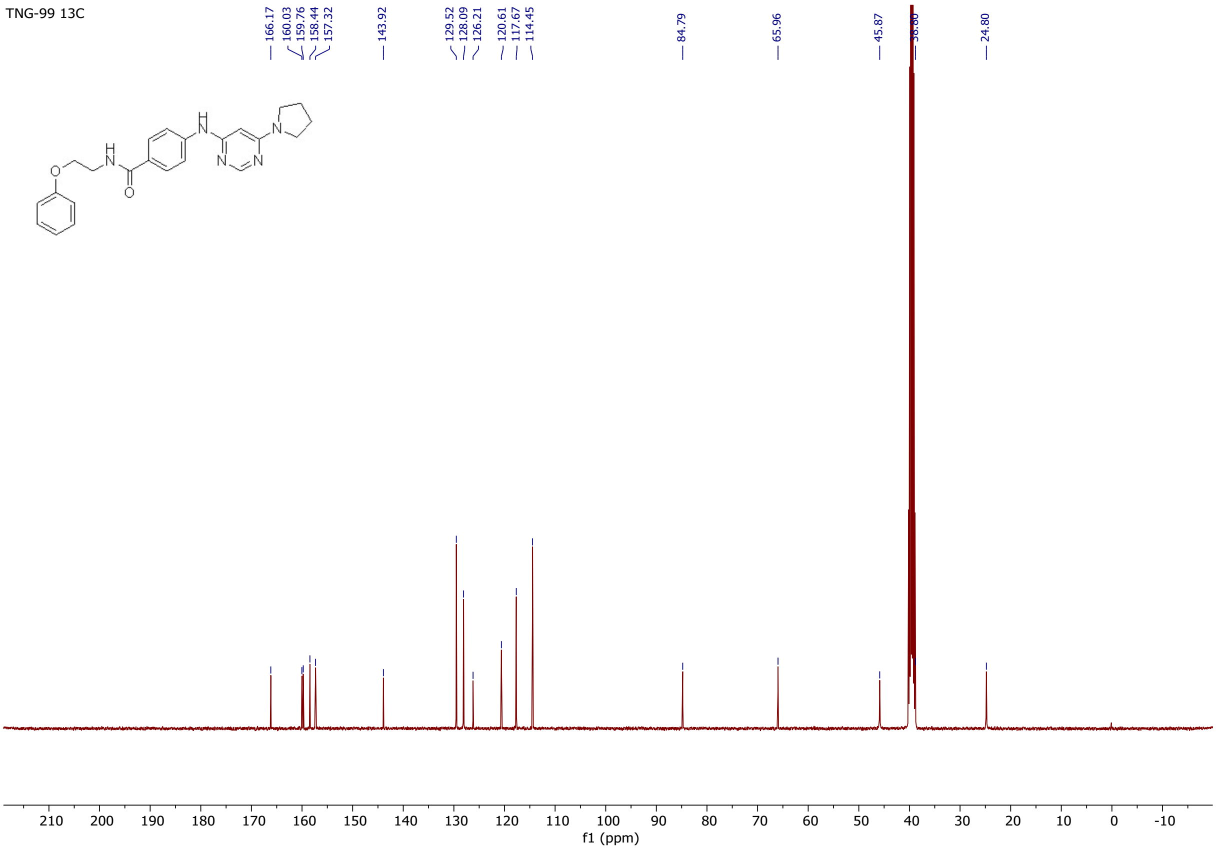

Compound **11h** ^{1}H NMR (400 MHz, DMSO-d_6):

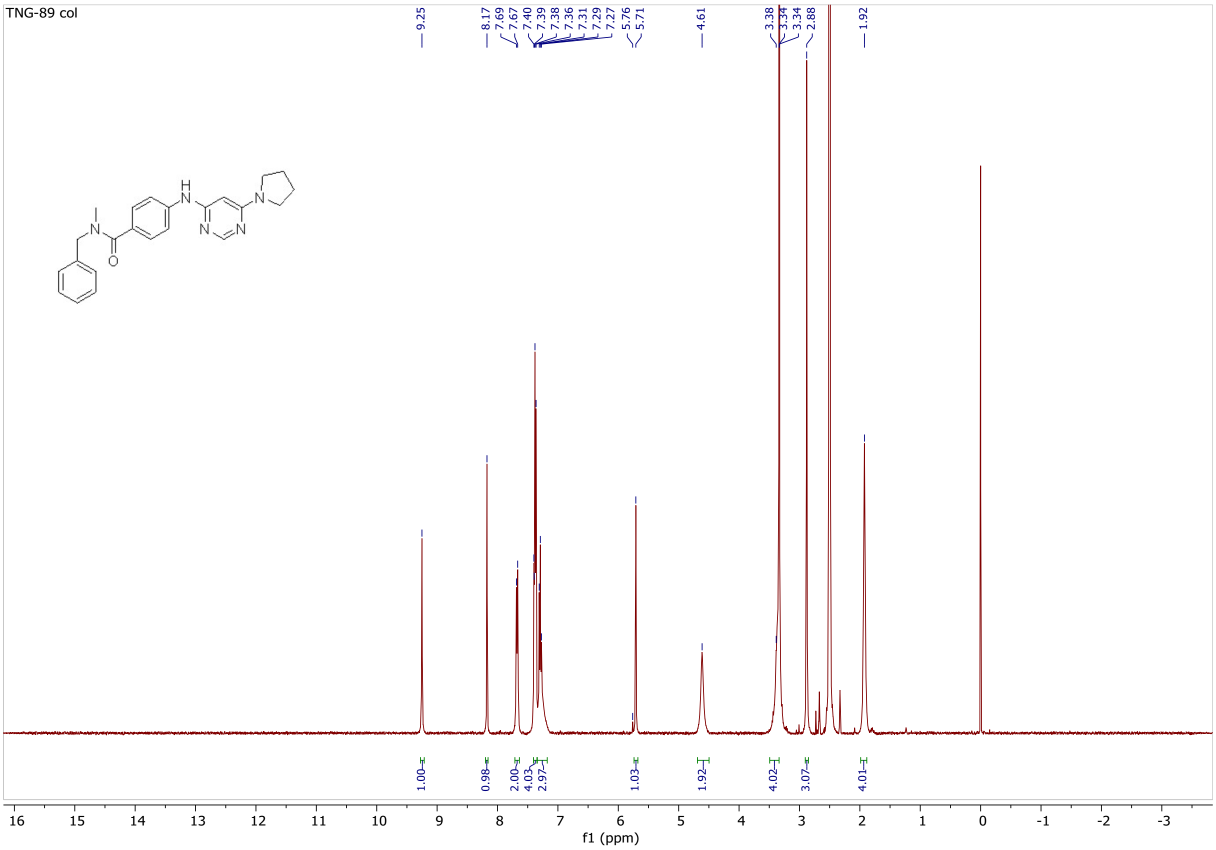

Compound **11h** ^{13}C NMR (101 MHz, DMSO-d_6):

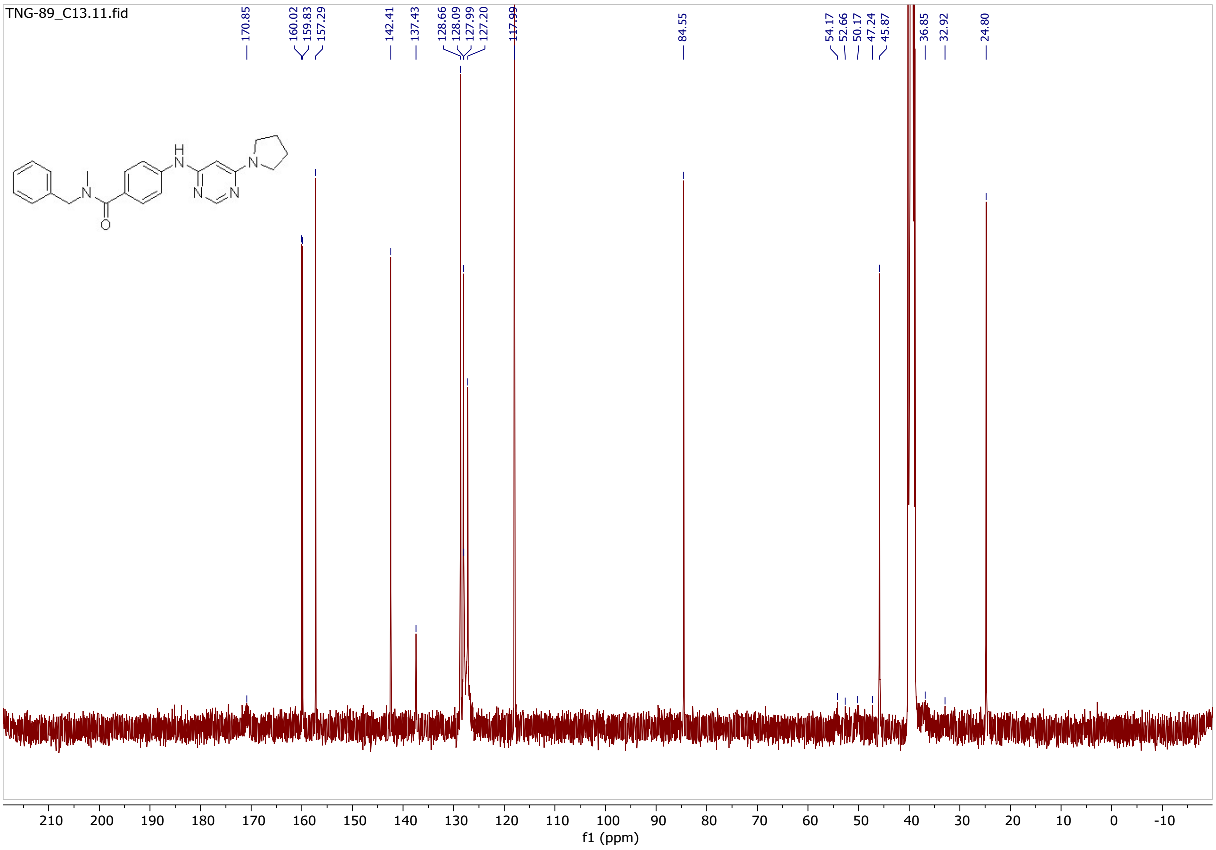

Compound **11i** ^{1}H NMR (400 MHz, DMSO-d_6):

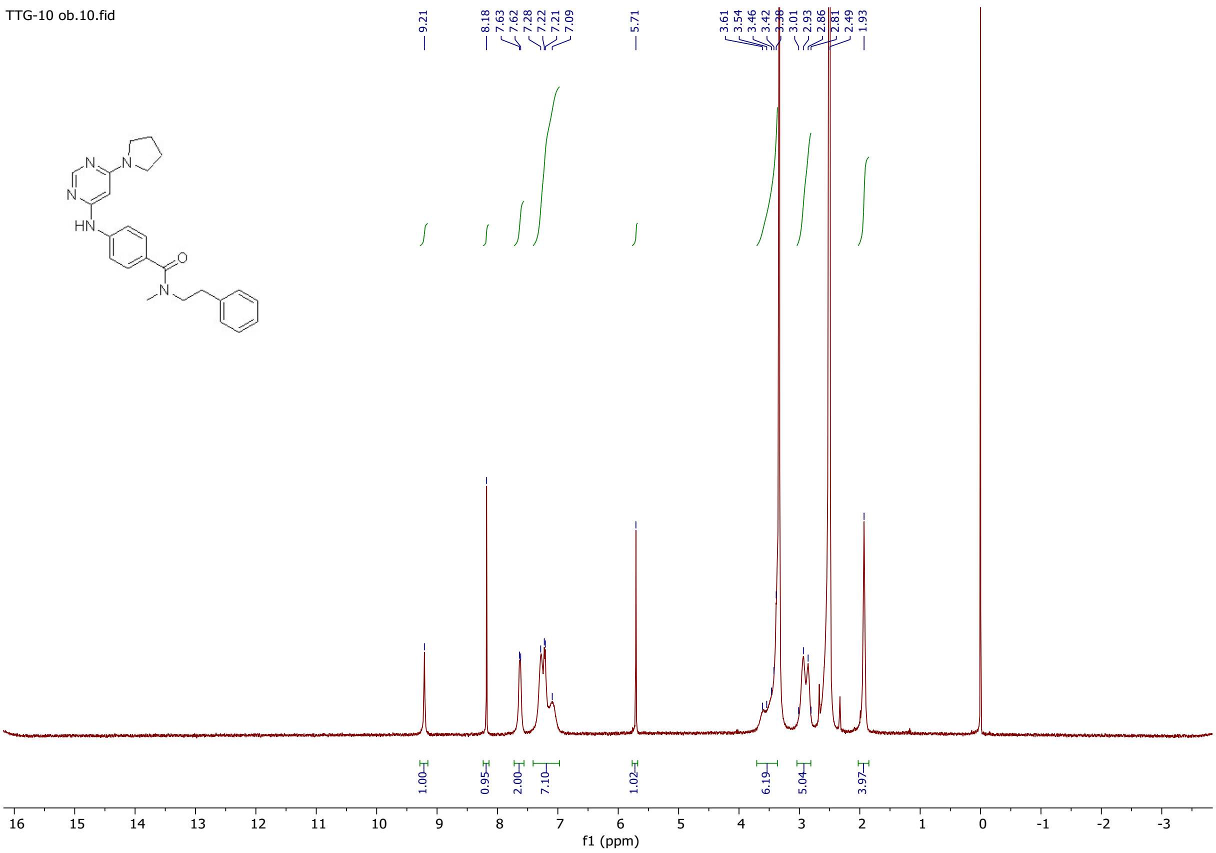

Compound **11i** ^{13}C NMR (101 MHz, DMSO-d_6):

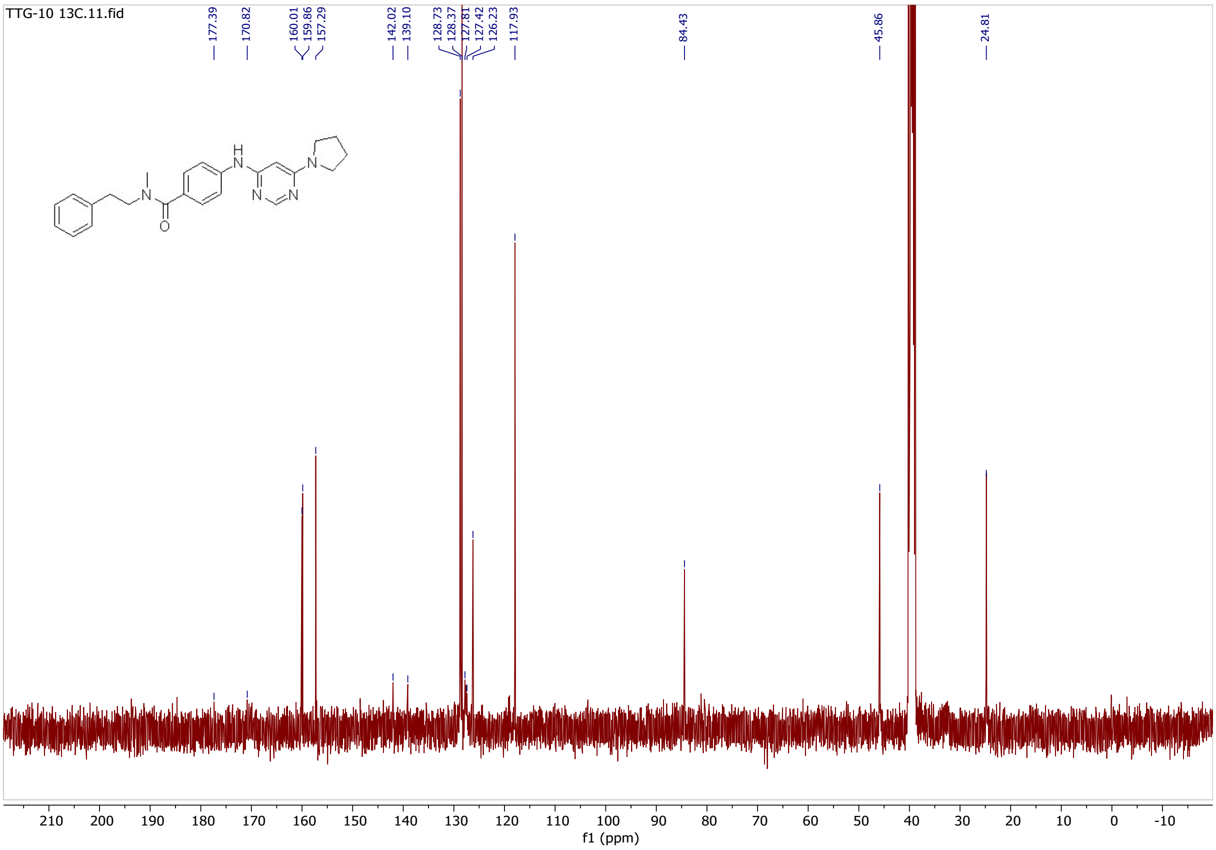

Compound **11j** ^{1}H NMR (400 MHz, DMSO-d_6):

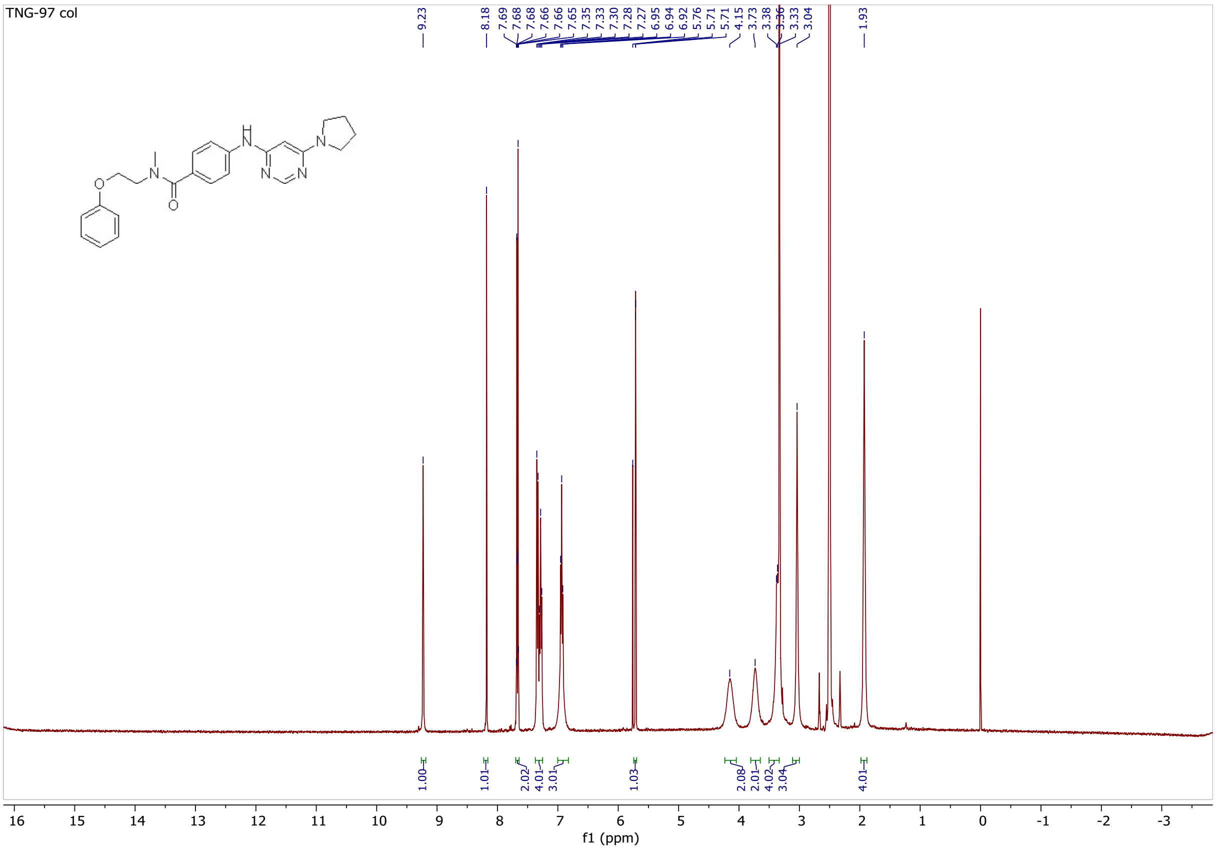

Compound **11j** ^{13}C NMR (101 MHz, DMSO-d_6):

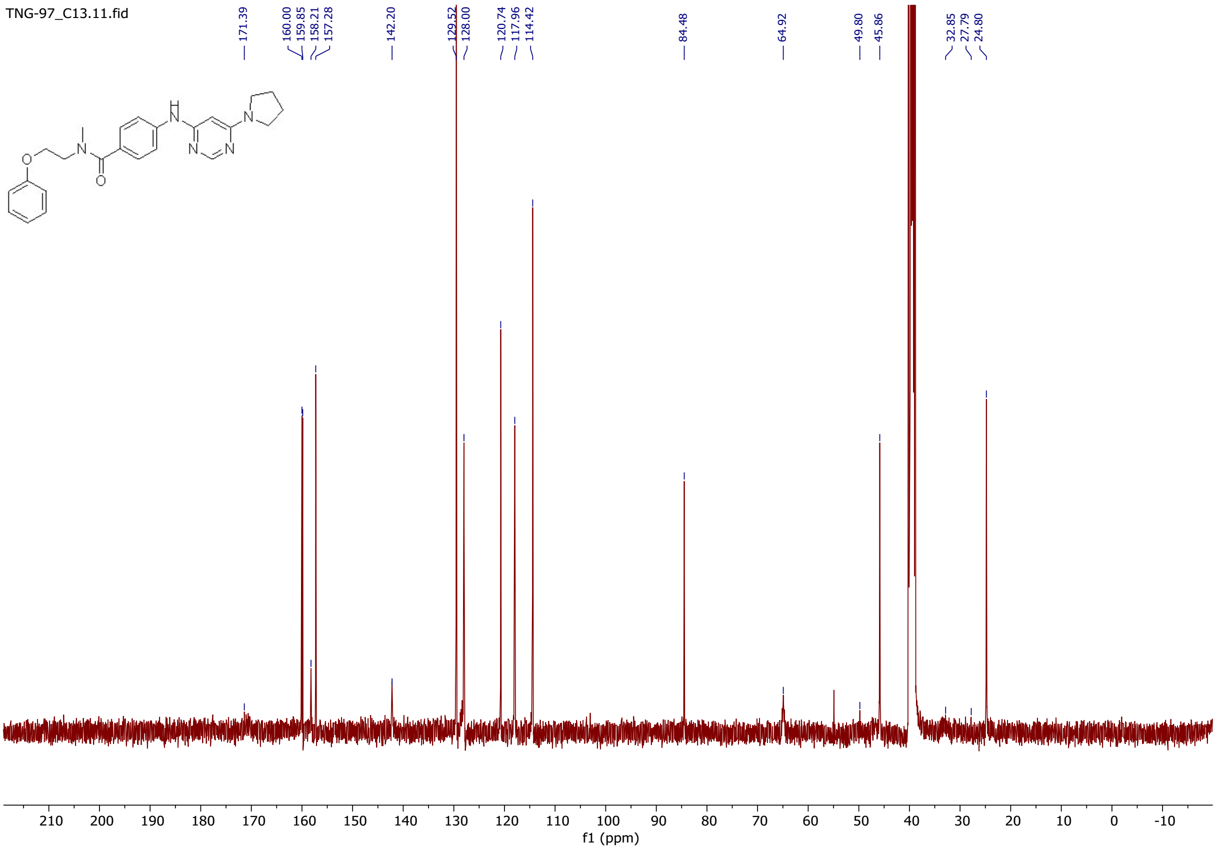

Compound **12g** ^{1}H NMR (400 MHz, DMSO-d_6):

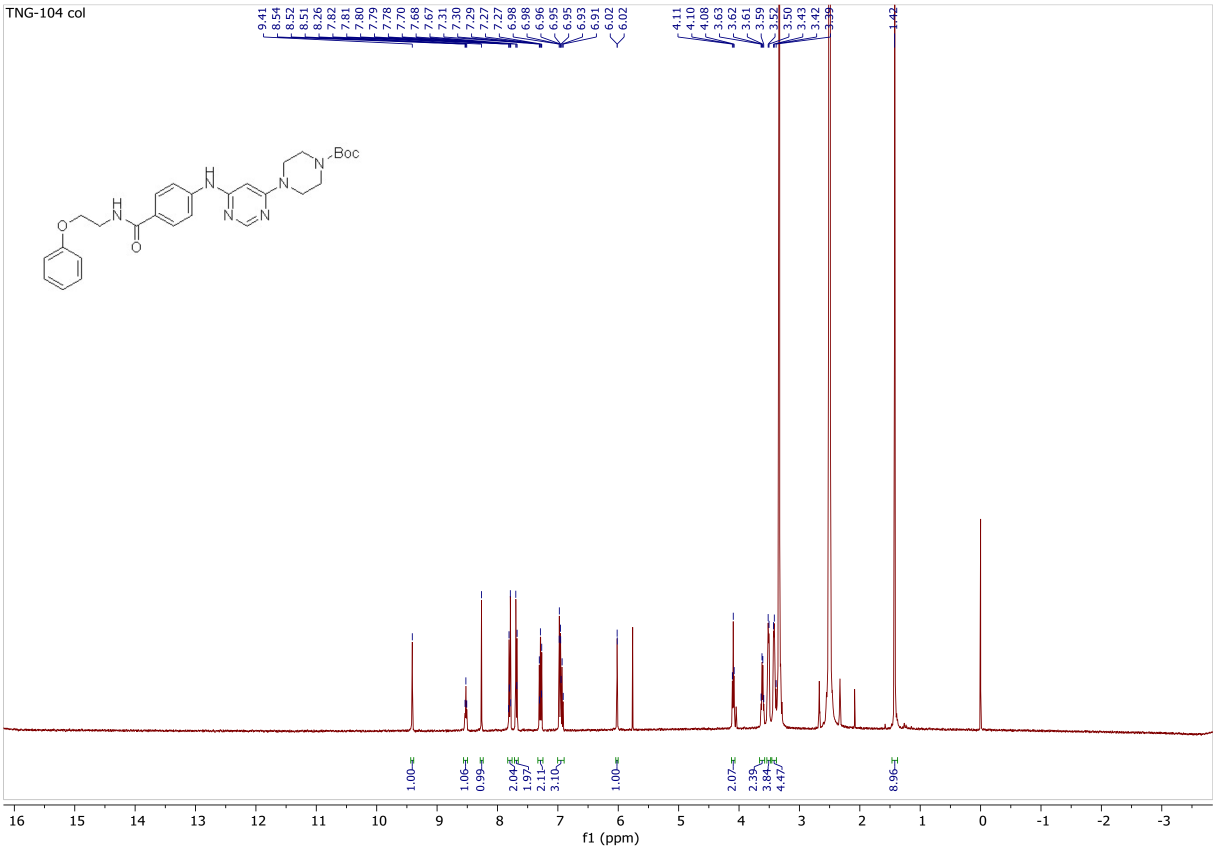

Compound **12g** ^{13}C NMR (101 MHz, DMSO-d_6):

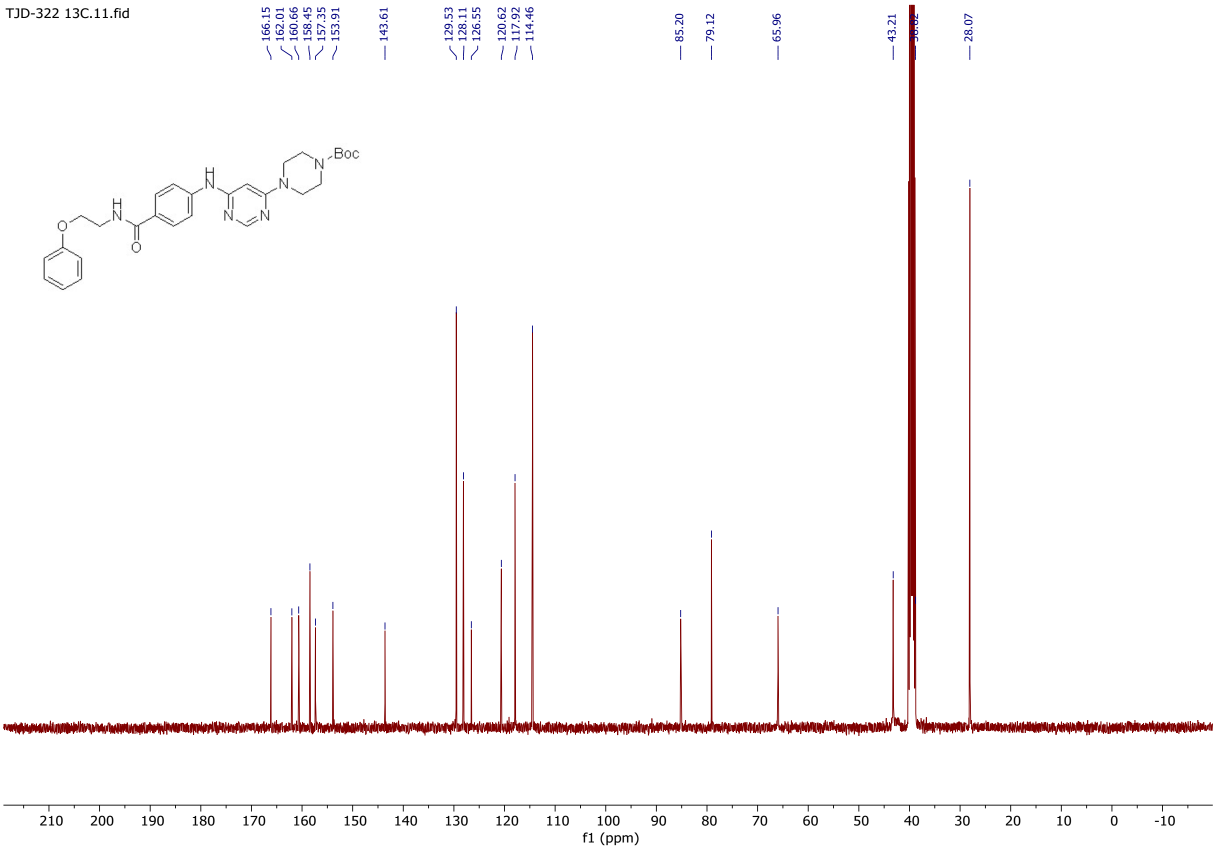

Compound **13a** ^{1}H NMR (400 MHz, DMSO-d_6):

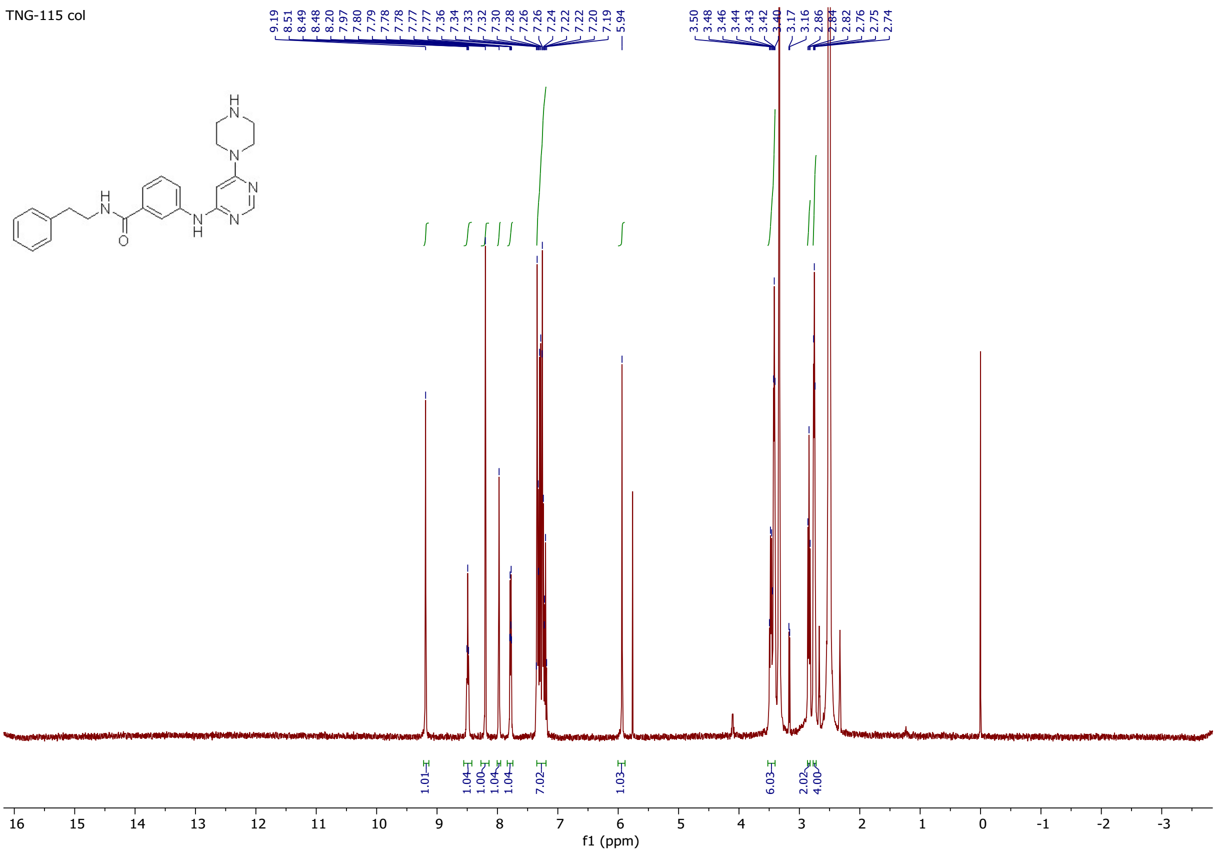

Compound **13a** ^{13}C NMR (101 MHz, DMSO-d_6):

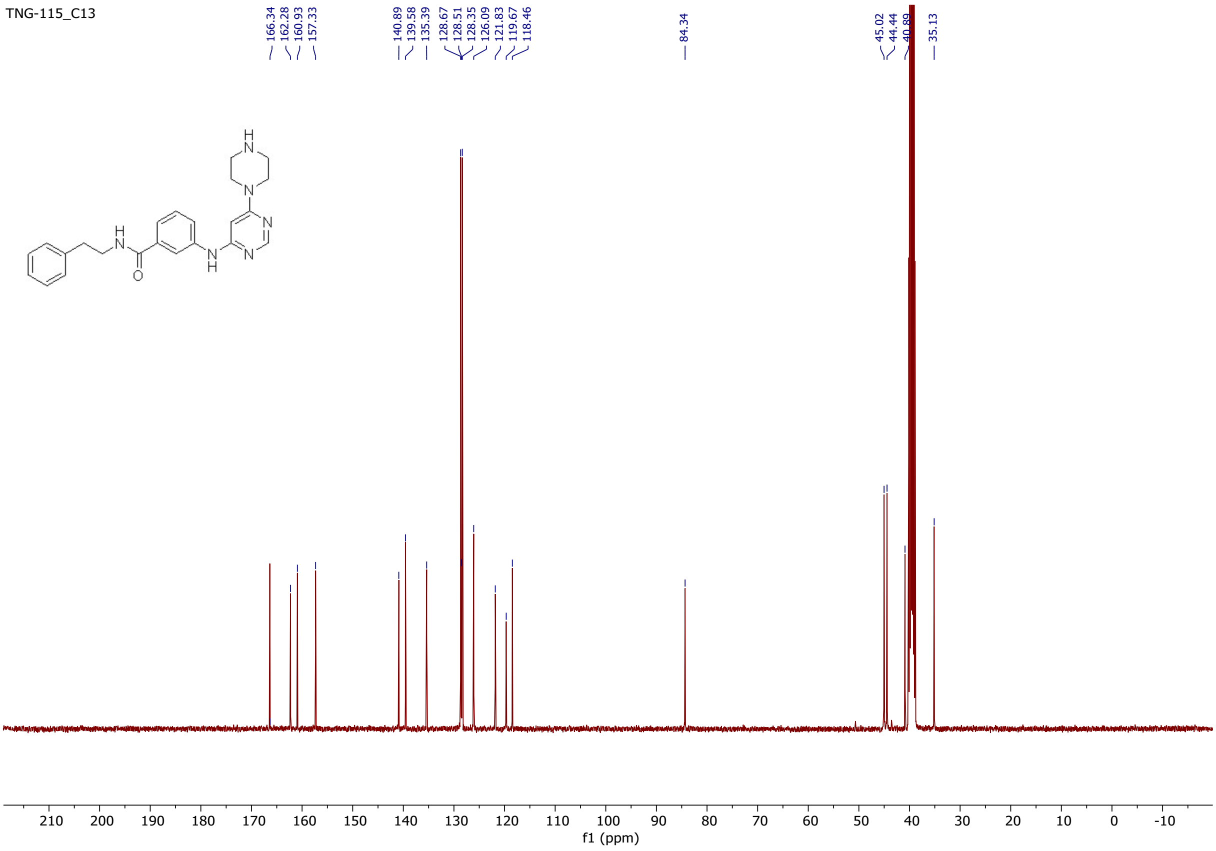

Compound **13b** ^{1}H NMR (400 MHz, DMSO-d_6):

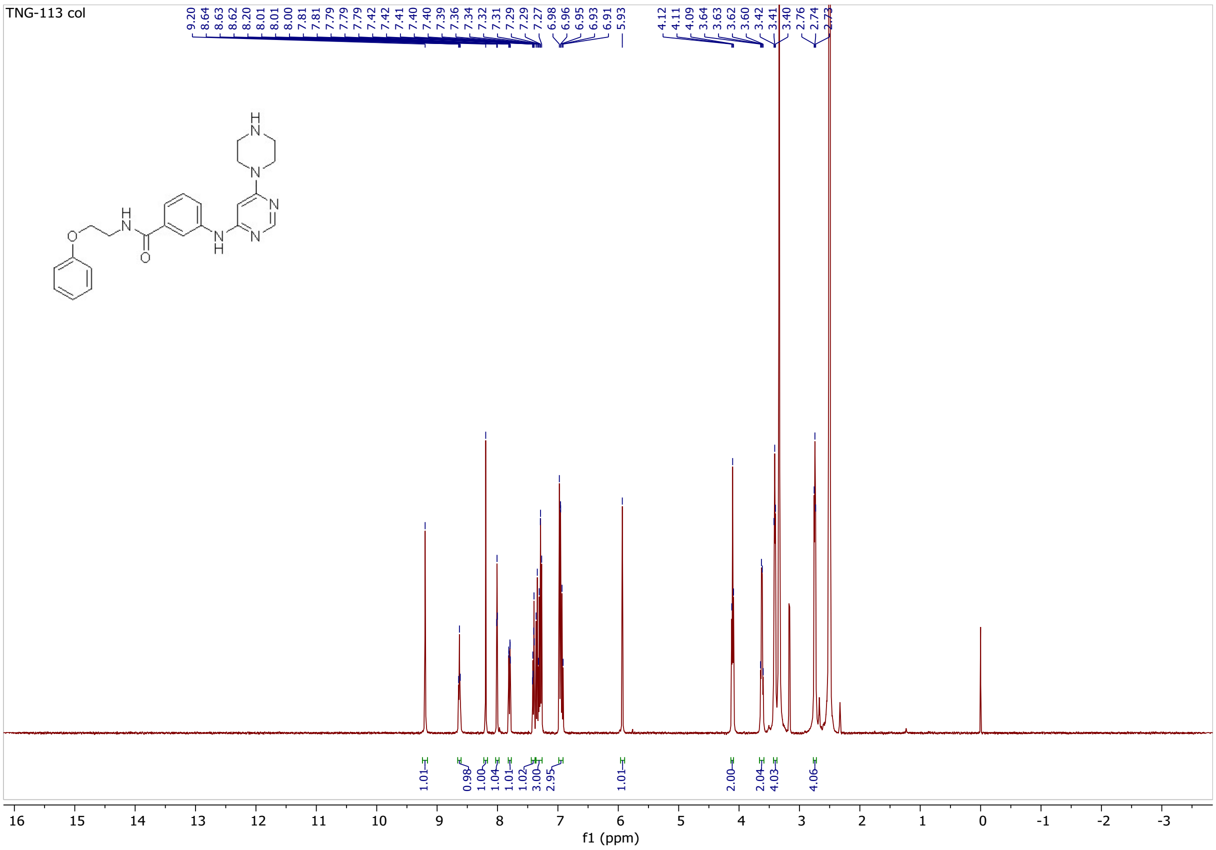

Compound **13b** ^{13}C NMR (101 MHz, DMSO-d_6):

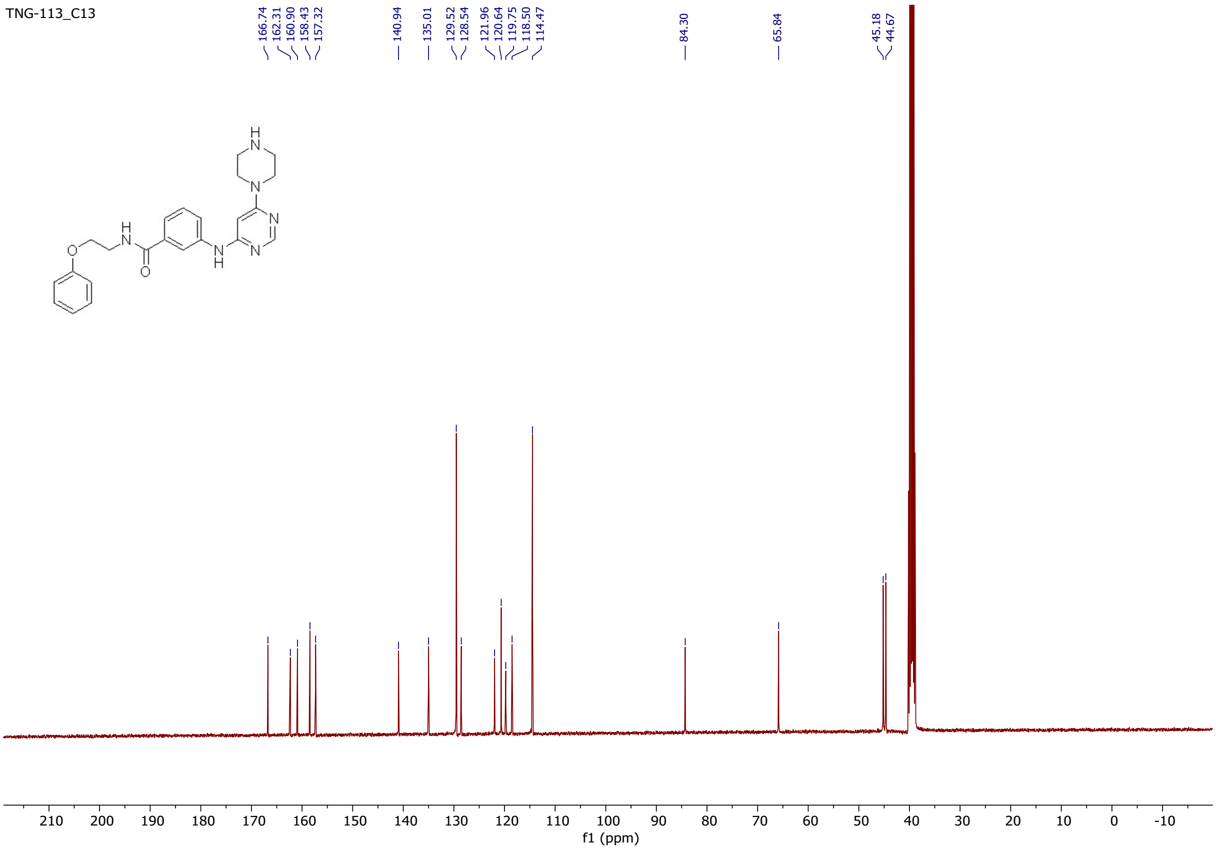

Compound **13c** ^{1}H NMR (400 MHz, DMSO-d_6):

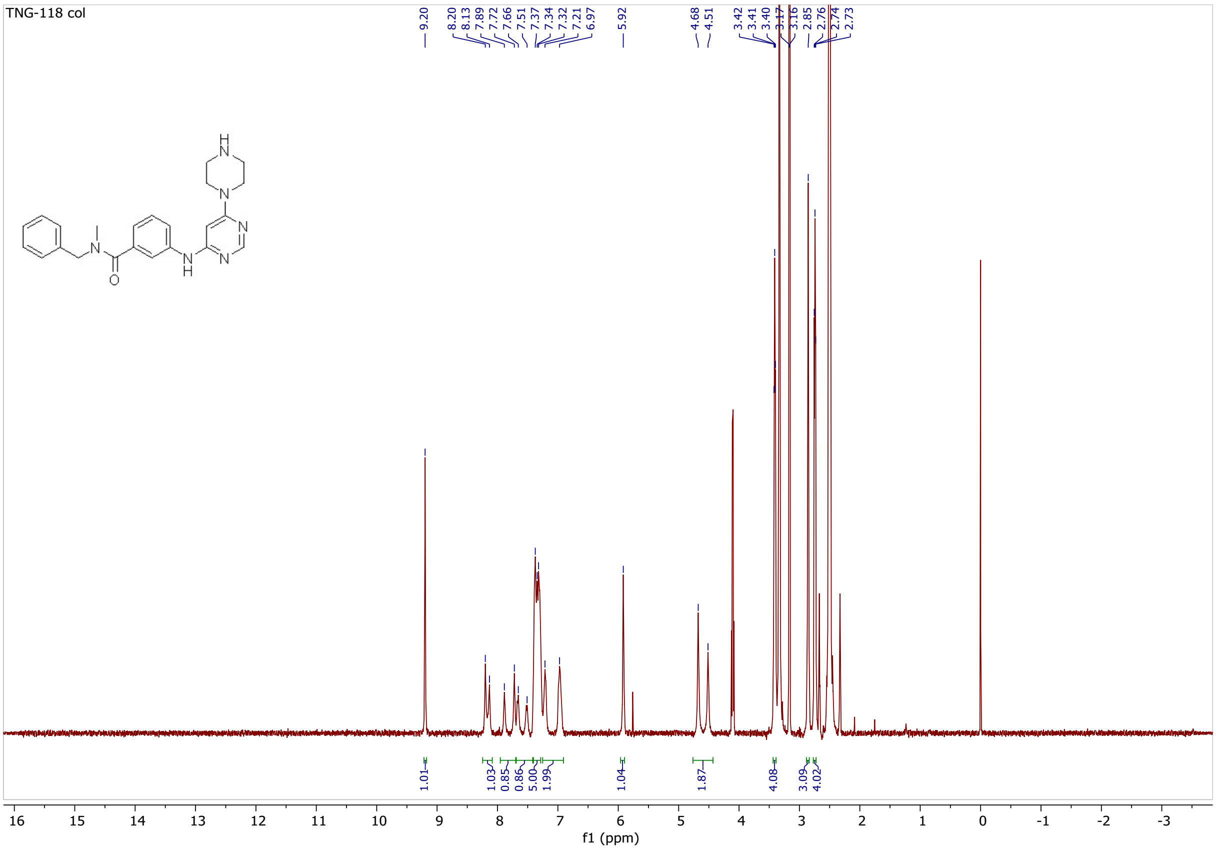

Compound **13c** ^{13}C NMR (101 MHz, DMSO-d_6):

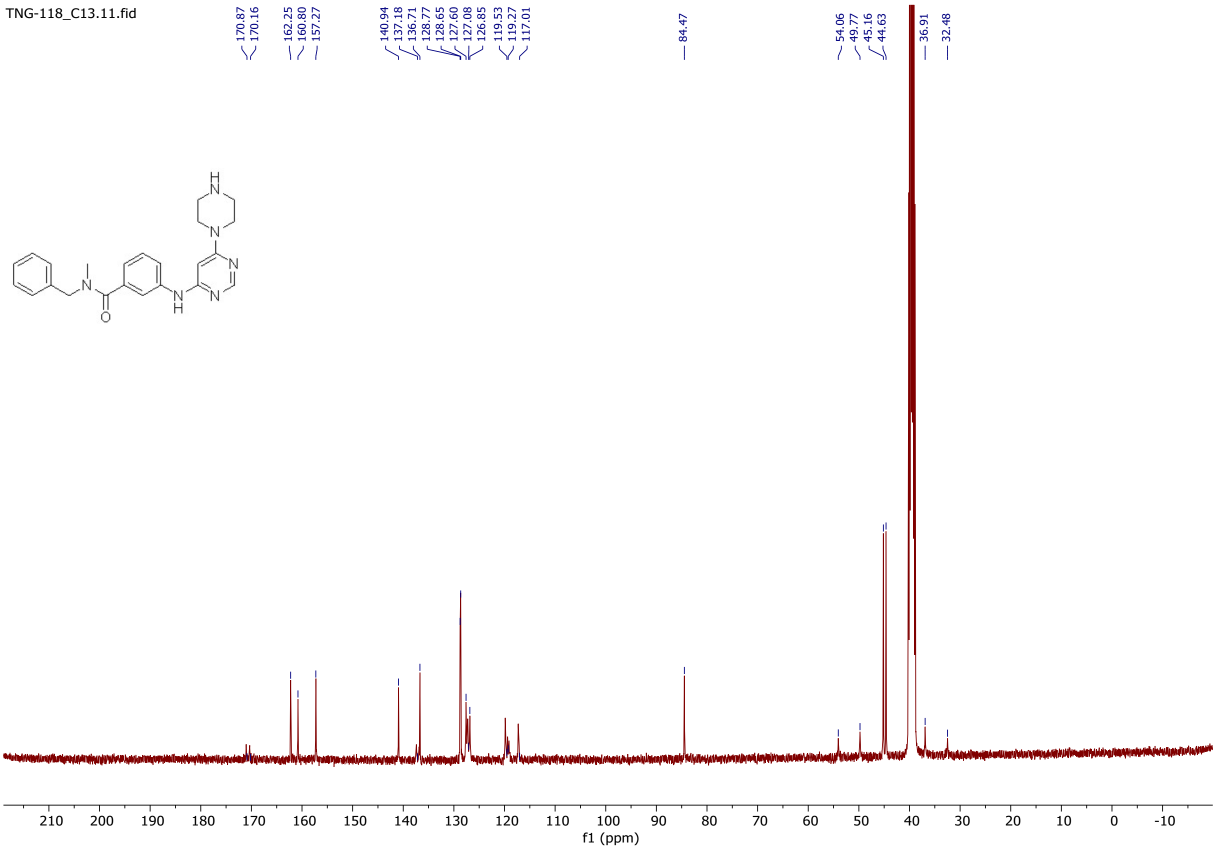

Compound **13d** ^{1}H NMR (400 MHz, DMSO-d_6):

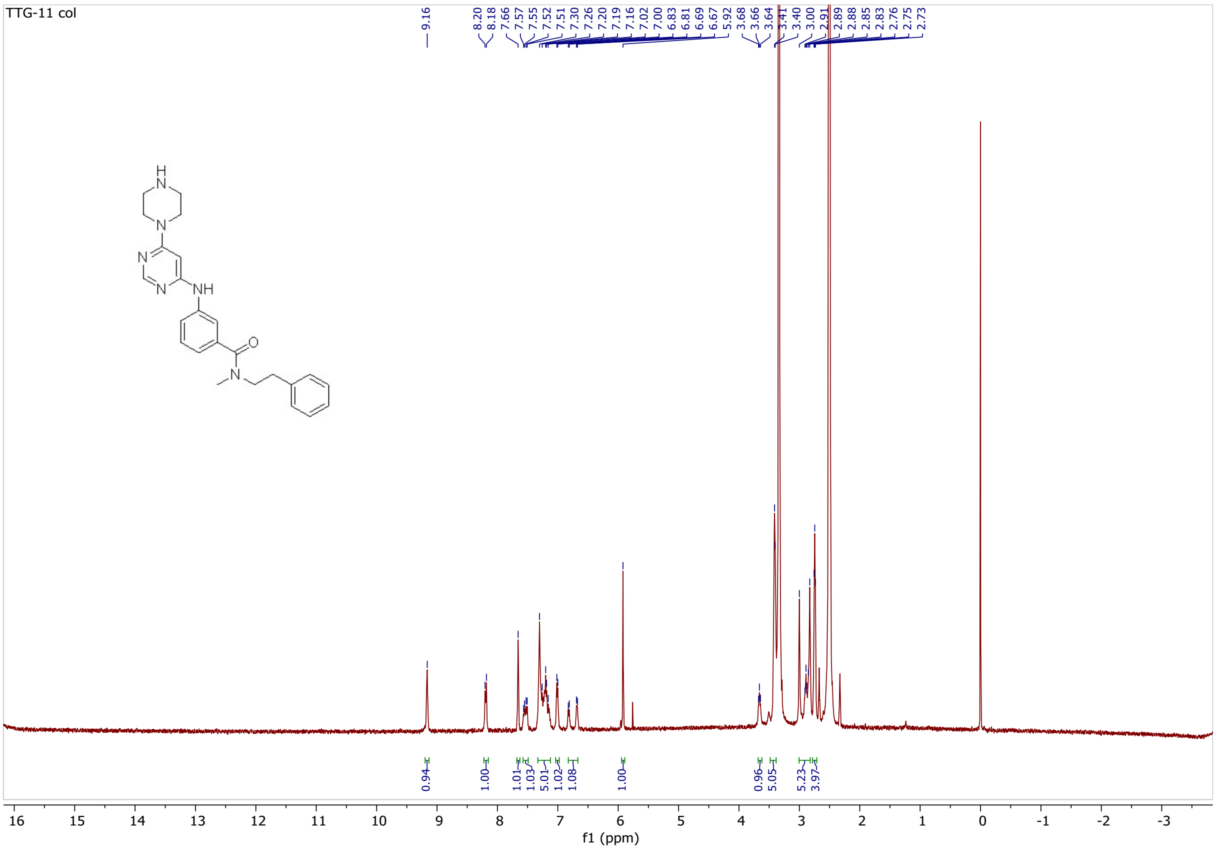

Compound **13d** ^{13}C NMR (101 MHz, DMSO-d_6):

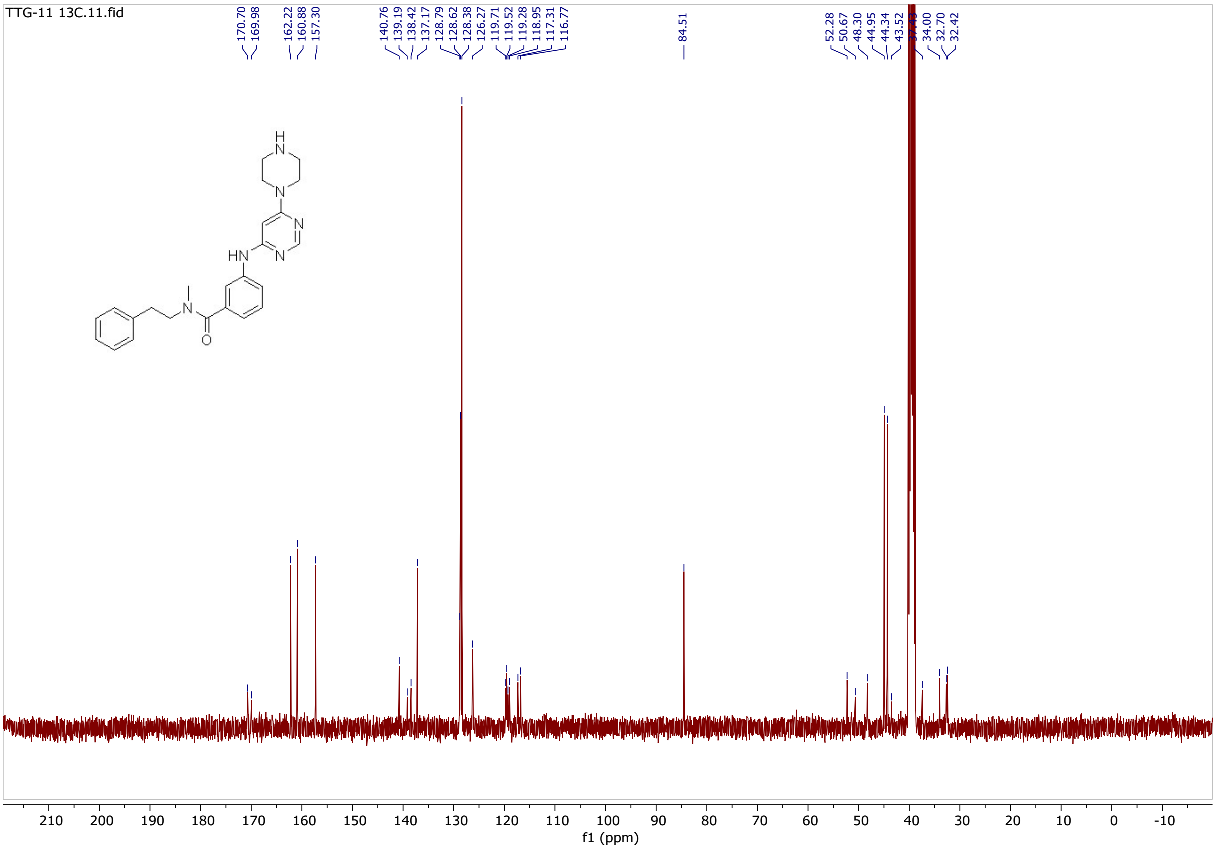

Compound **13e** ^{1}H NMR (400 MHz, DMSO-d_6):

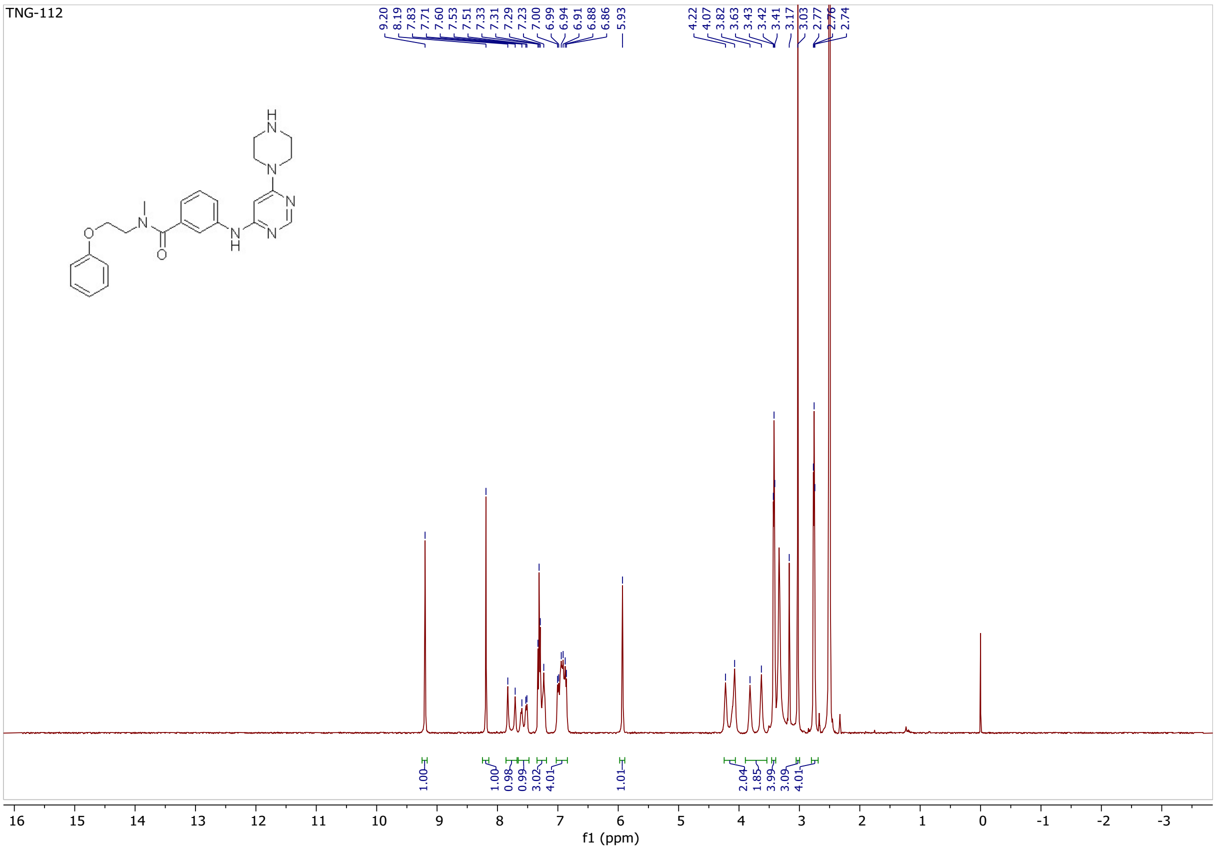

Compound **13e** ^{13}C NMR (101 MHz, DMSO-d_6):

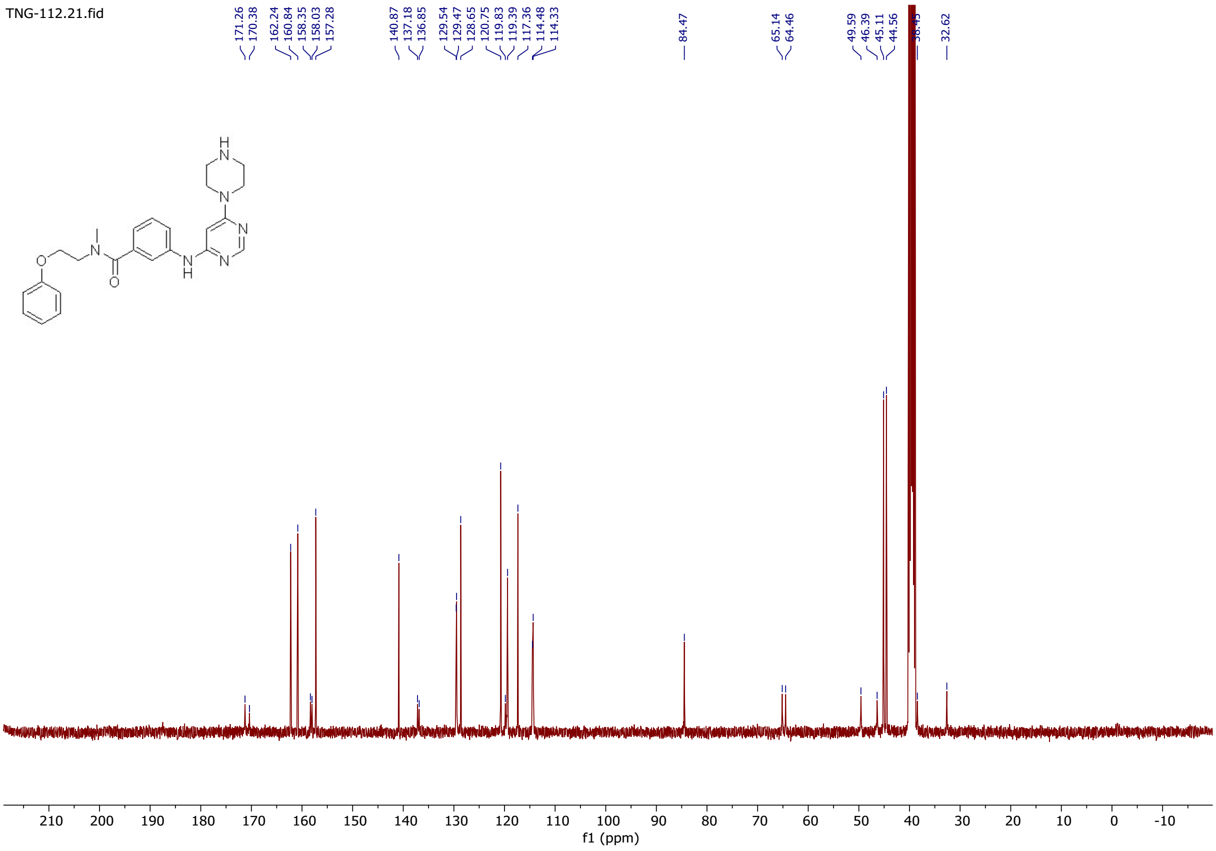

Compound **13f** ^{1}H NMR (400 MHz, DMSO-d_6):

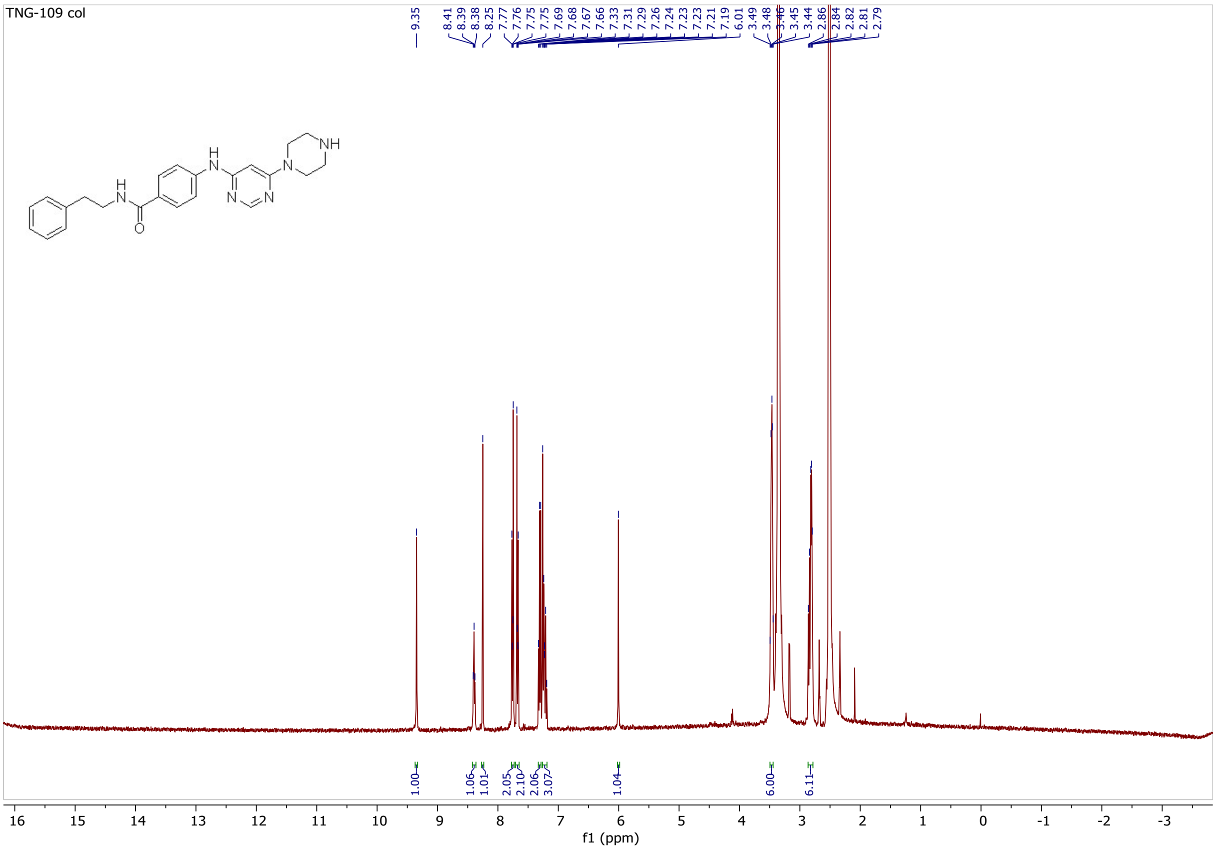

Compound **13f** ^{13}C NMR (101 MHz, DMSO-d_6):

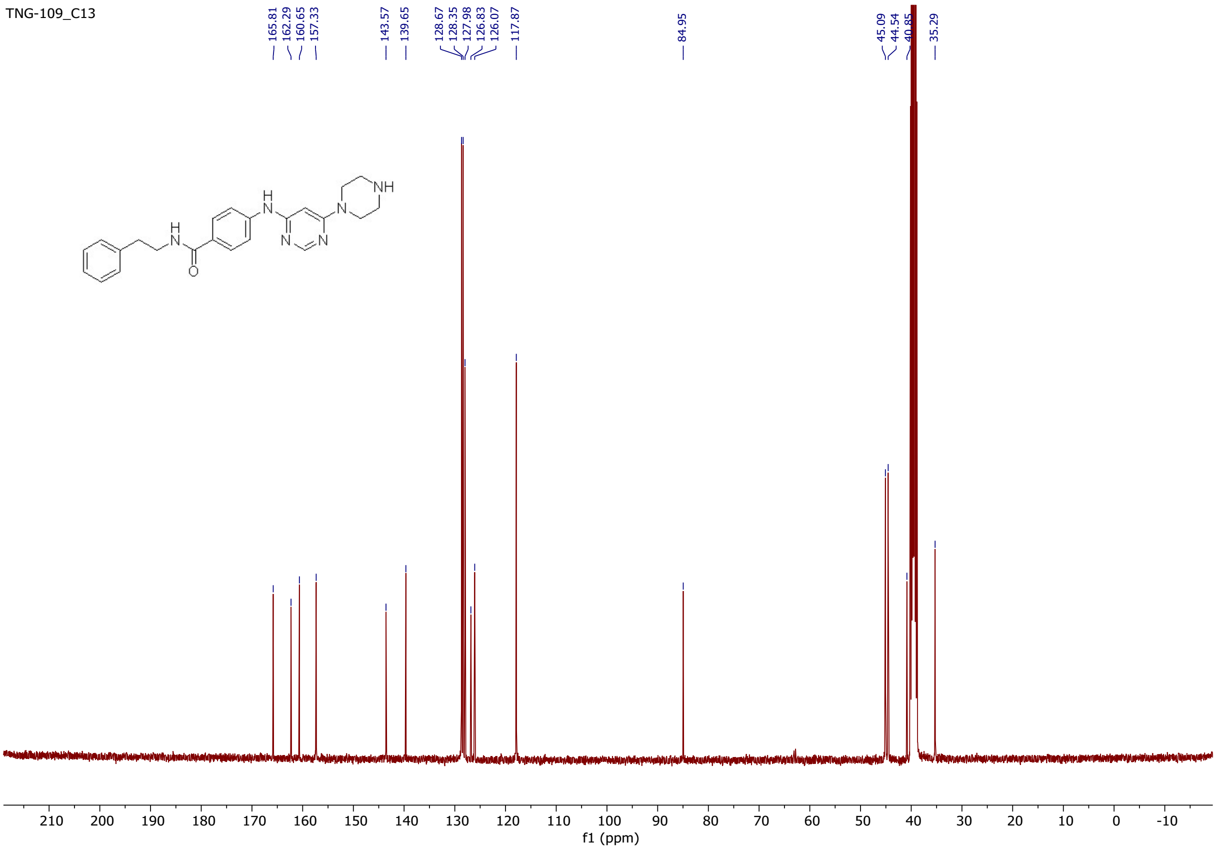

Compound **13g** ^{1}H NMR (400 MHz, DMSO-d_6):

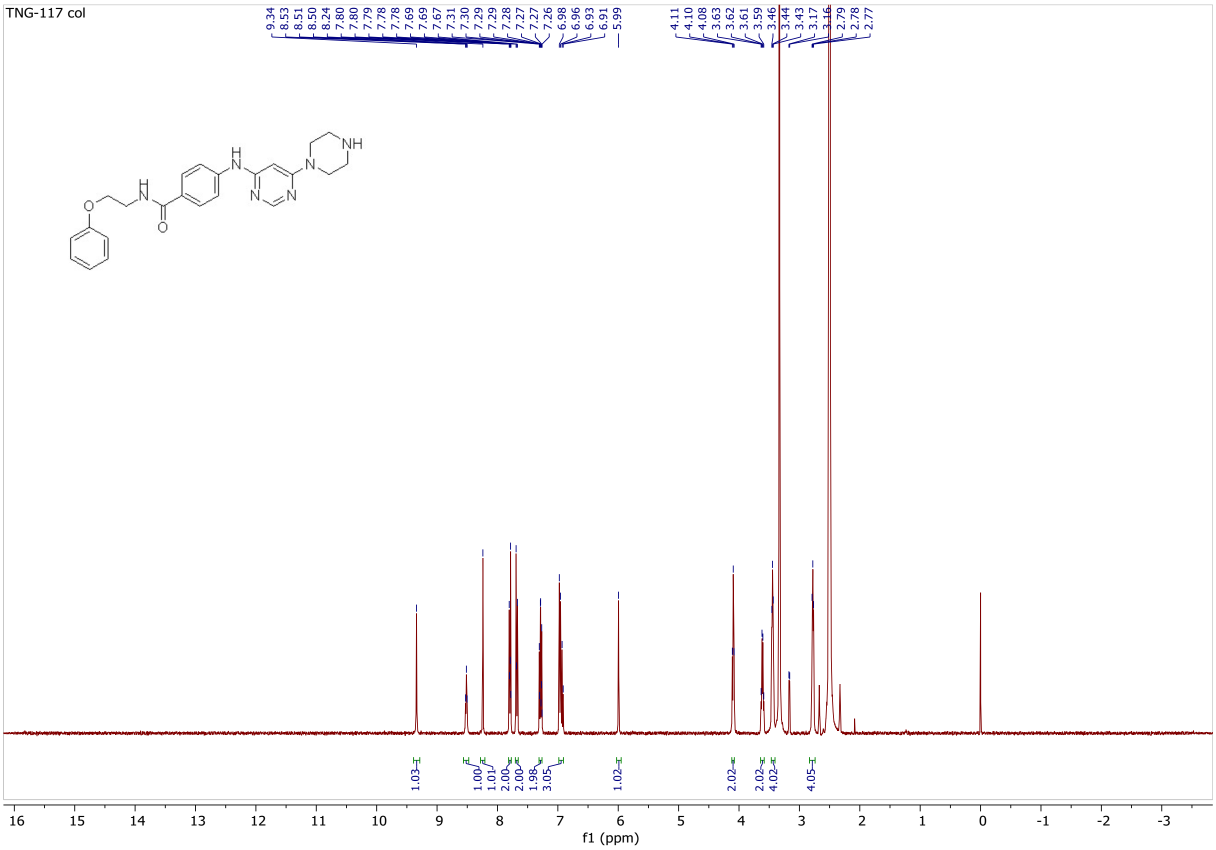

Compound **13g** ^{13}C NMR (101 MHz, DMSO-d_6):

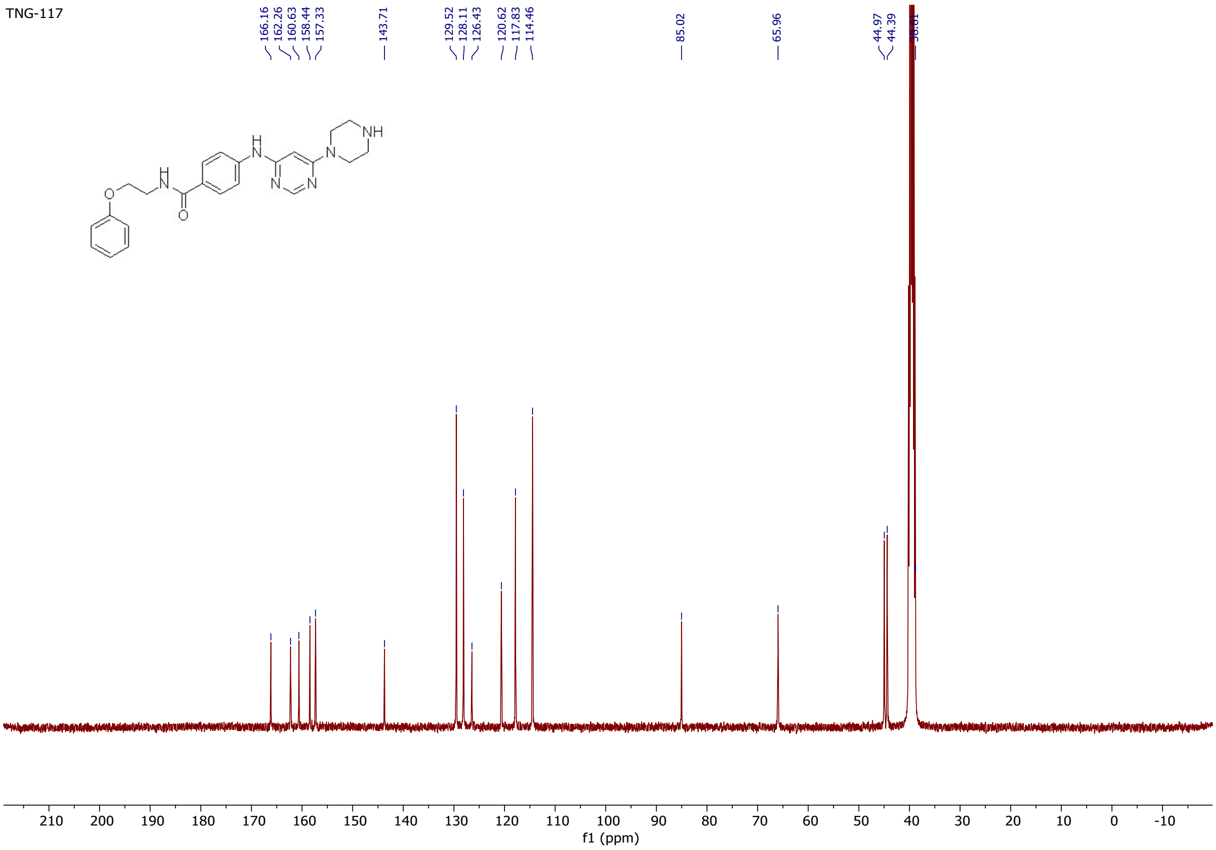

Compound **13h** ^{1}H NMR (400 MHz, DMSO-d_6):

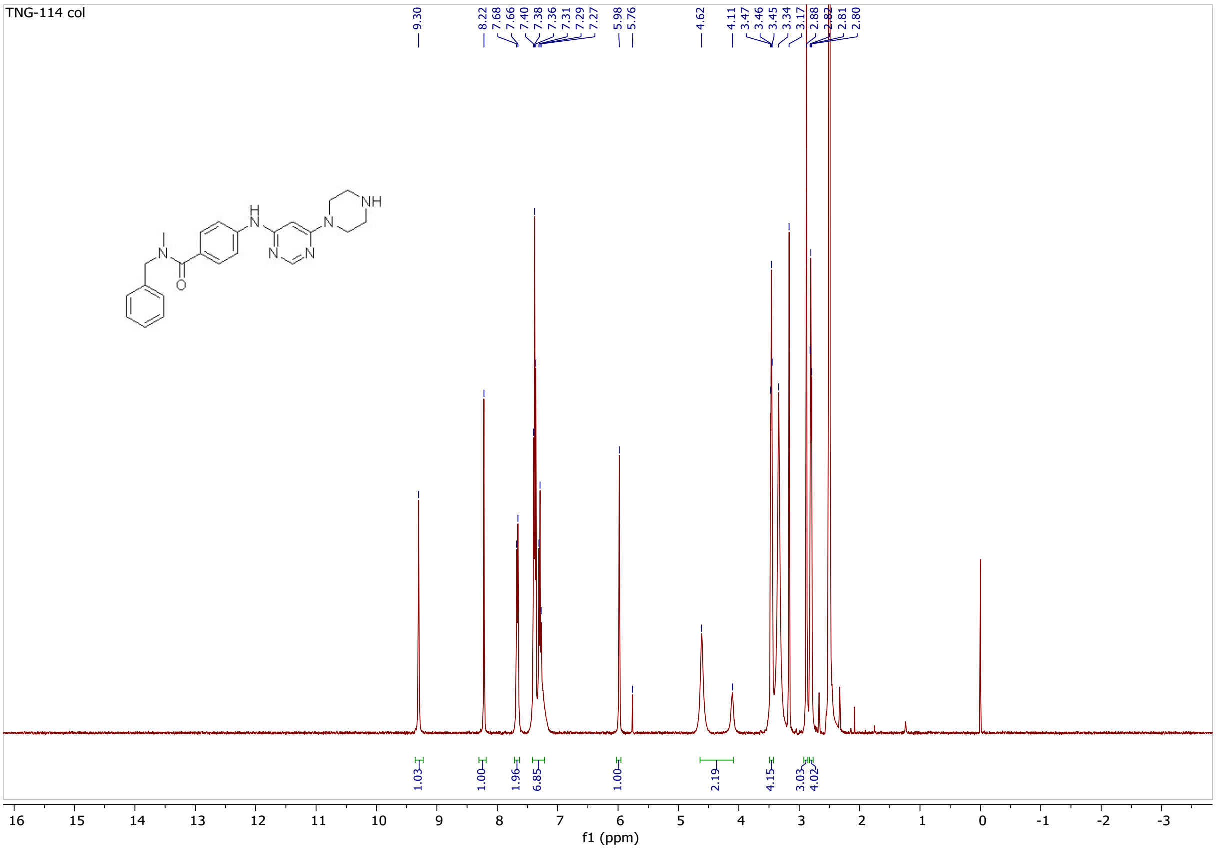

Compound **13h** ^{13}C NMR (101 MHz, DMSO-d_6):

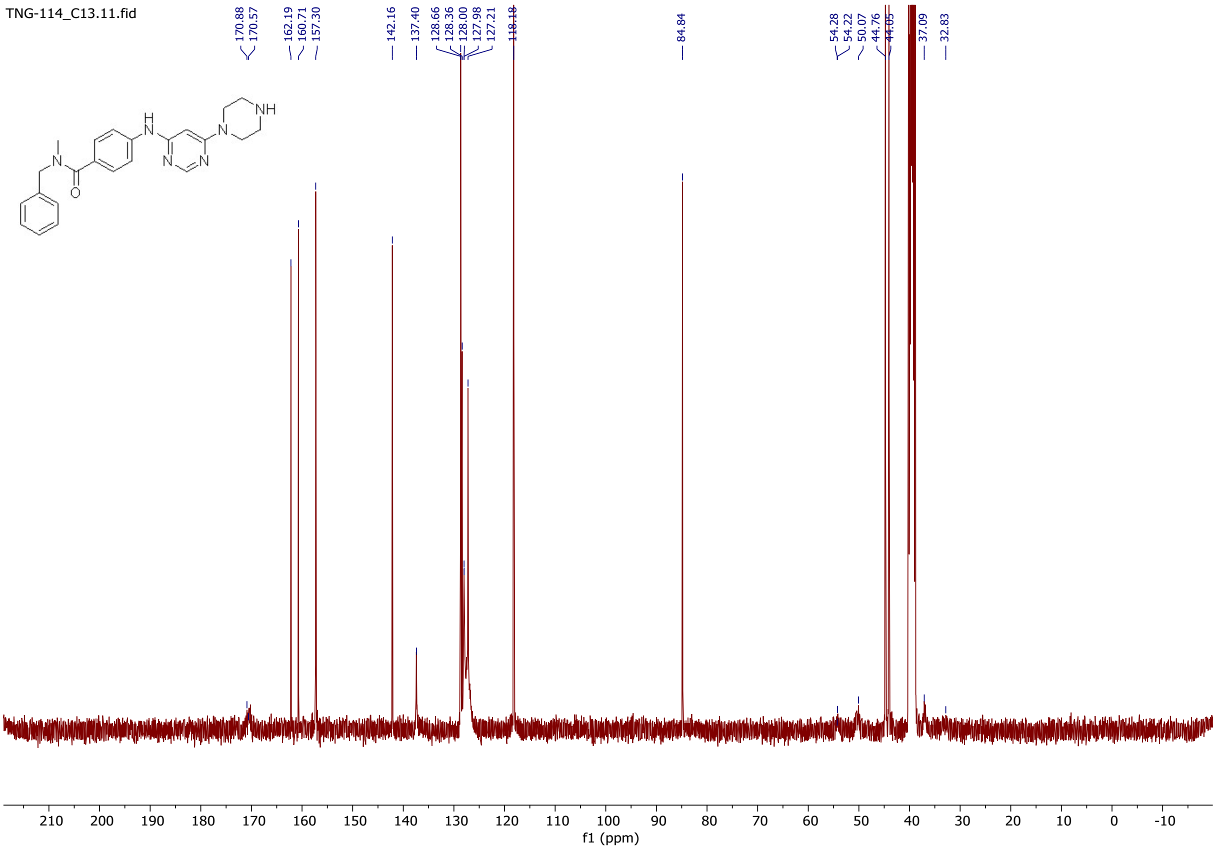

Compound **13i** ^{1}H NMR (400 MHz, DMSO-d_6):

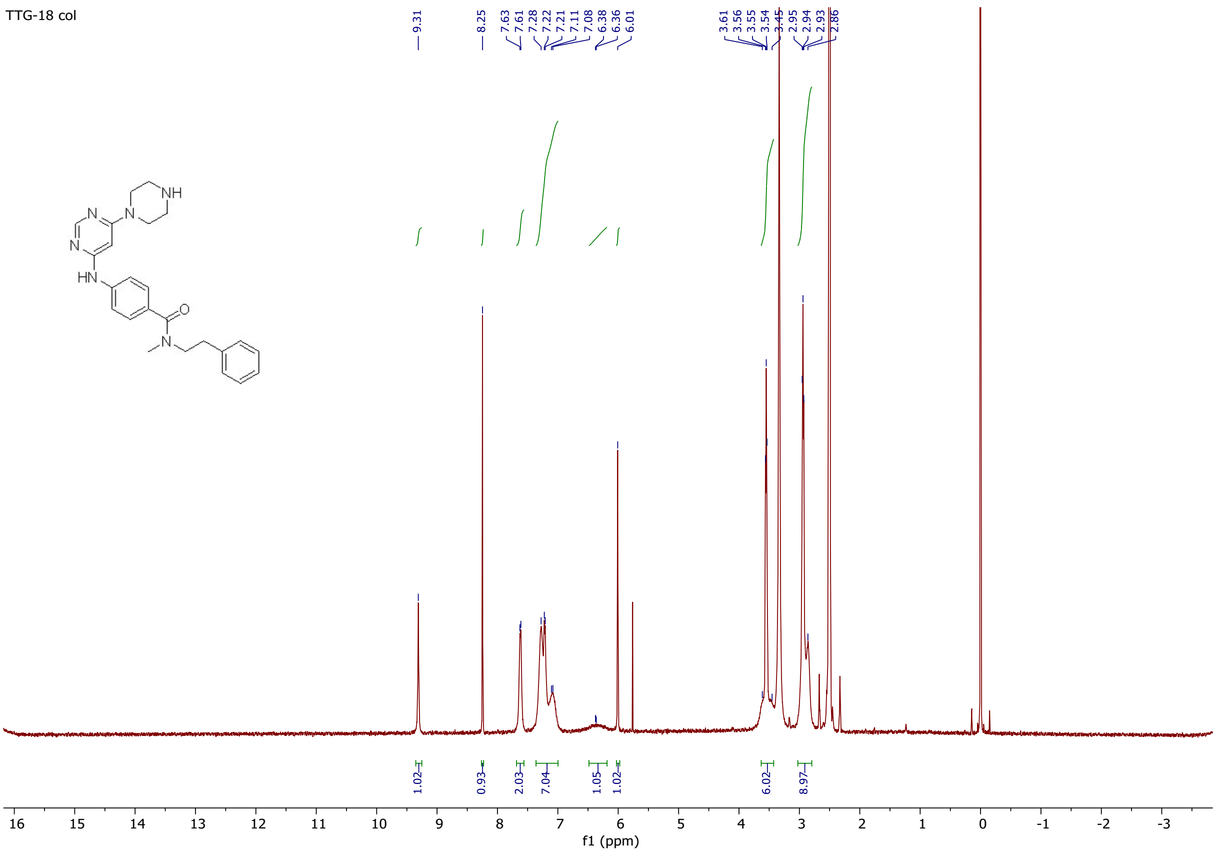

Compound **13i** ^{13}C NMR (101 MHz, DMSO-d_6):

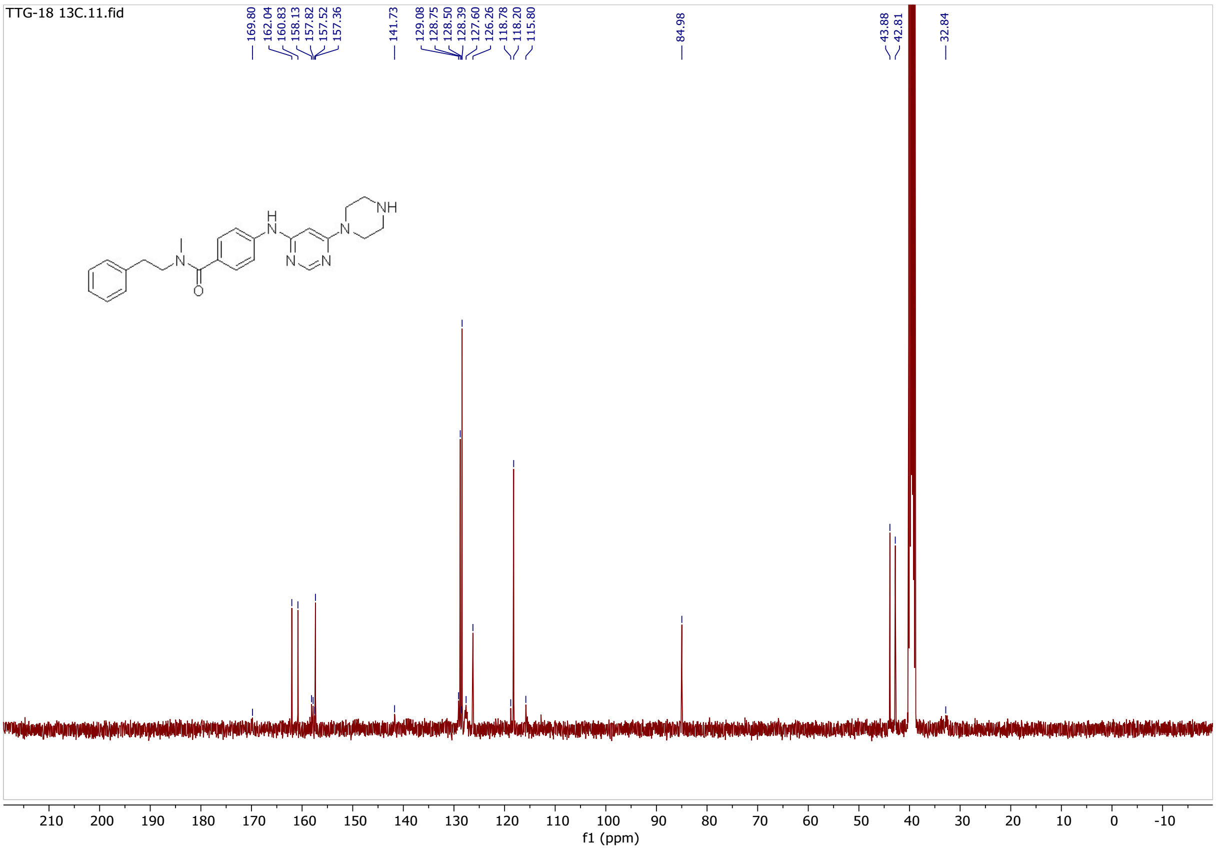

Compound **13j** ^{1}H NMR (400 MHz, DMSO-d_6):

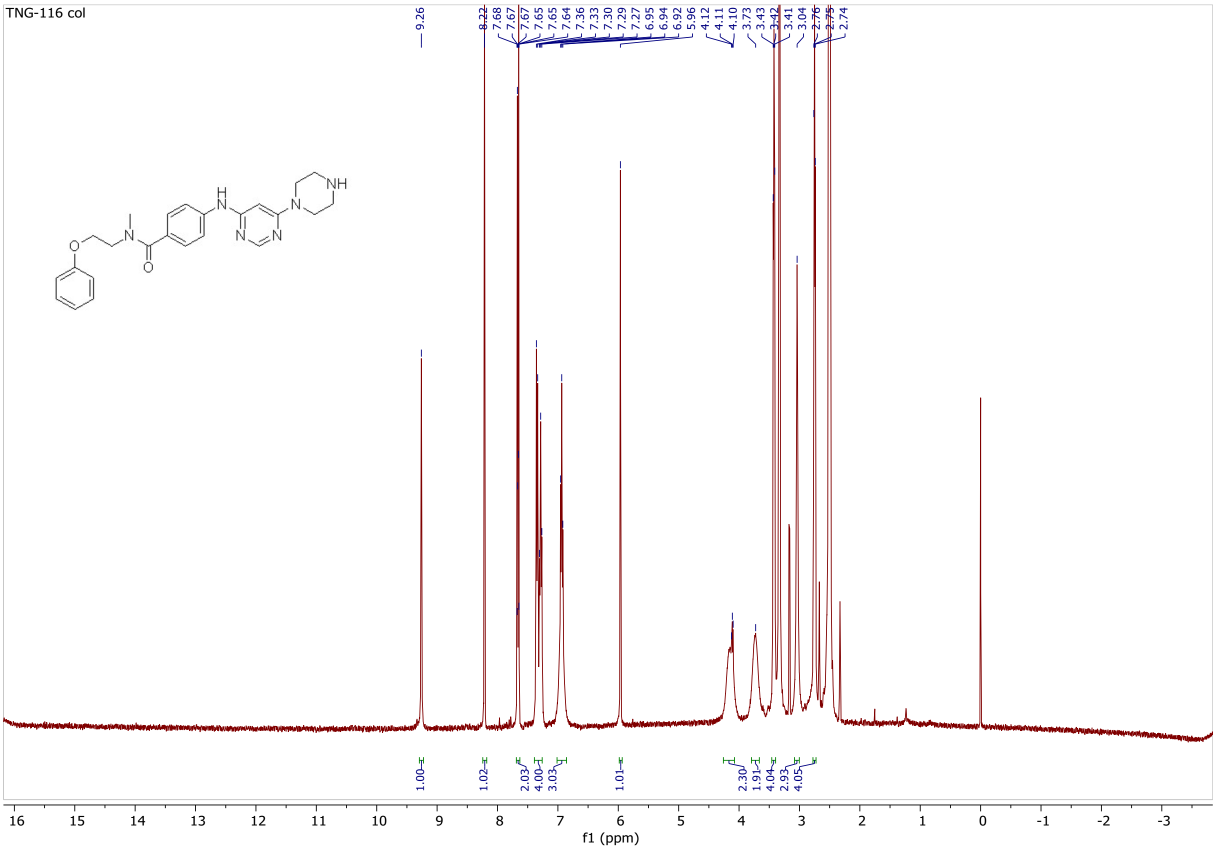

Compound **13j** ^{13}C NMR (101 MHz, DMSO-d_6):

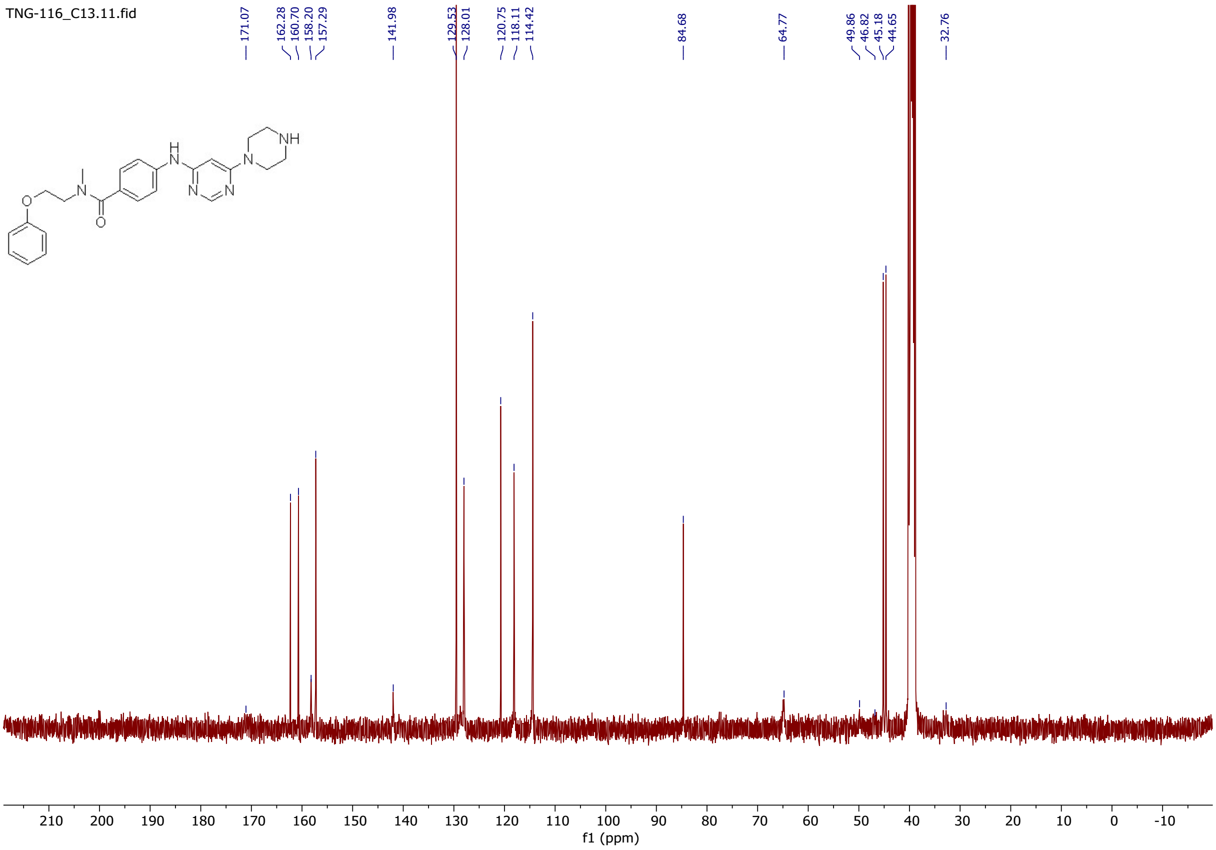

Compound **14** ^{1}H NMR (400 MHz, DMSO-d_6):

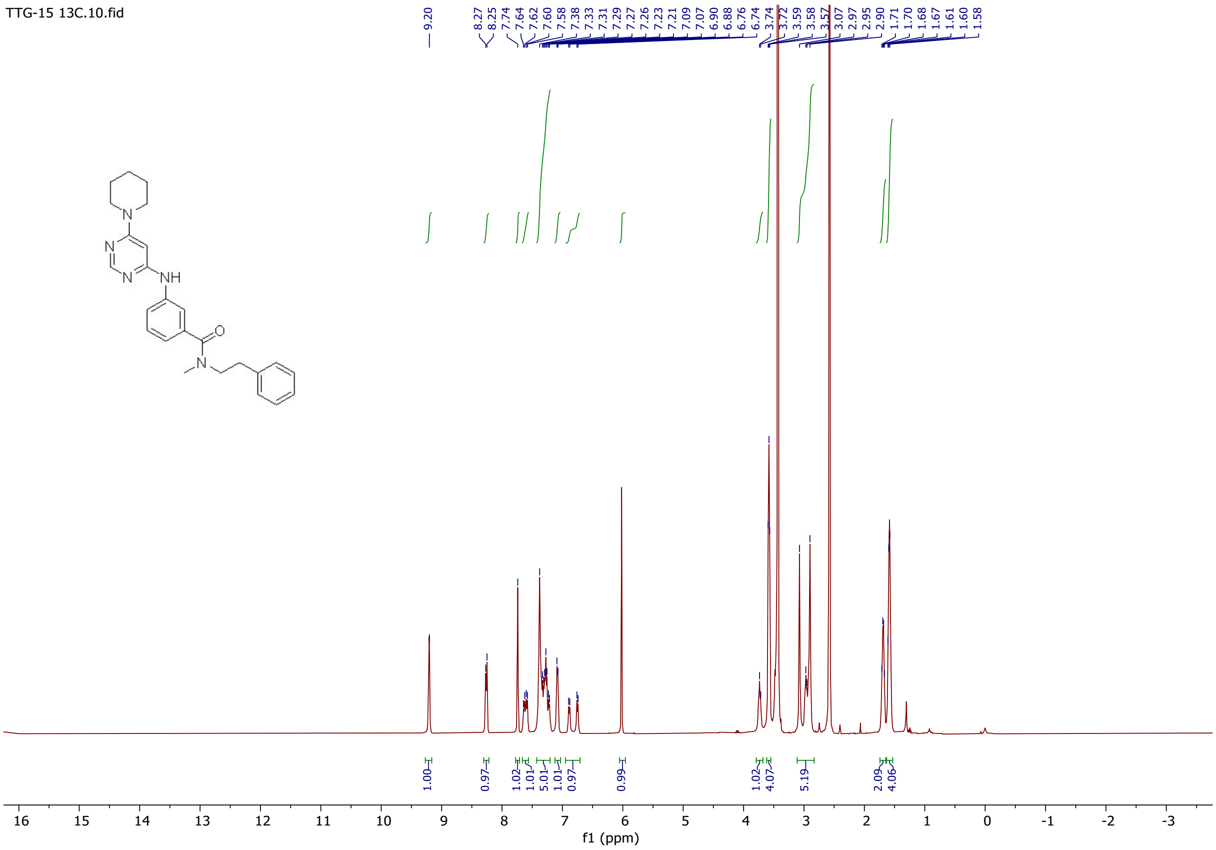

Compound **14** ^{13}C NMR (101 MHz, DMSO-d_6):

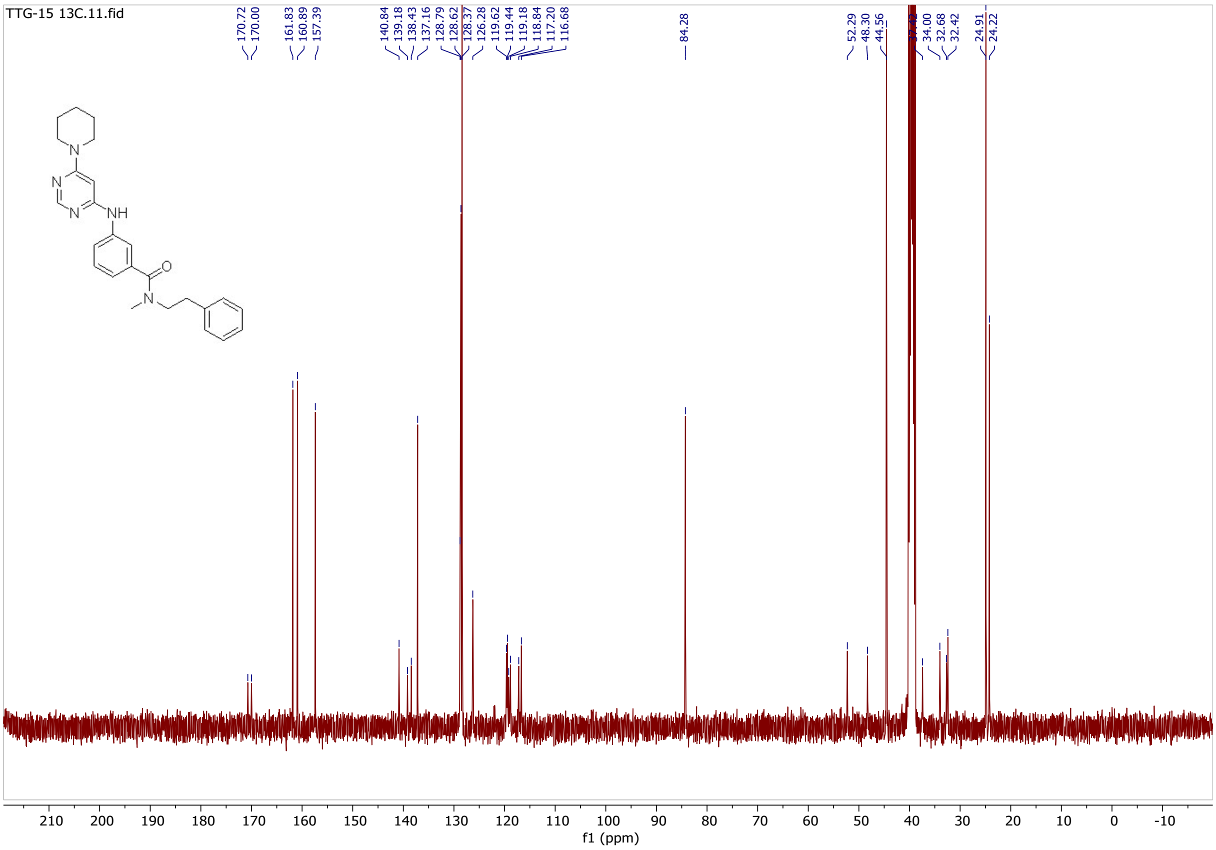

Compound **15** ^{1}H NMR (400 MHz, DMSO-d_6):

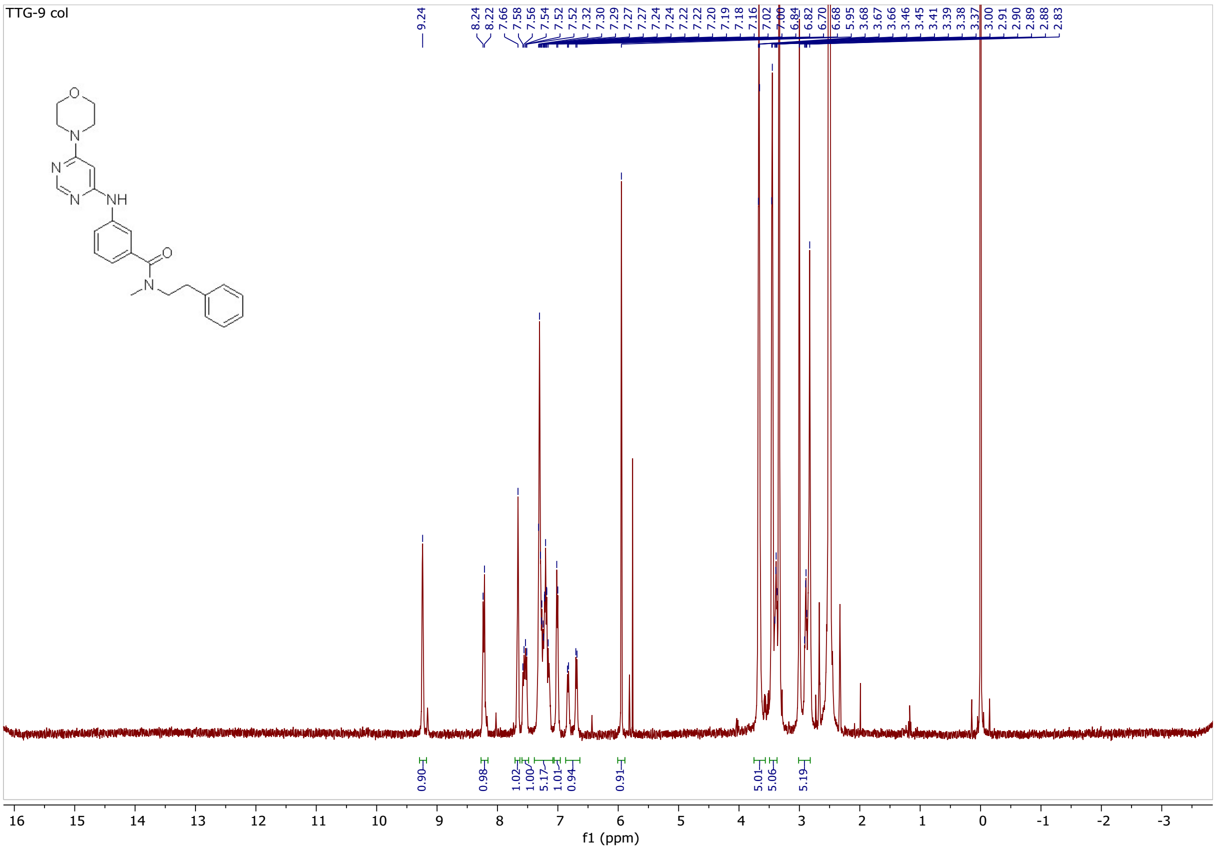

Compound **15** ^{13}C NMR (101 MHz, DMSO-d_6):

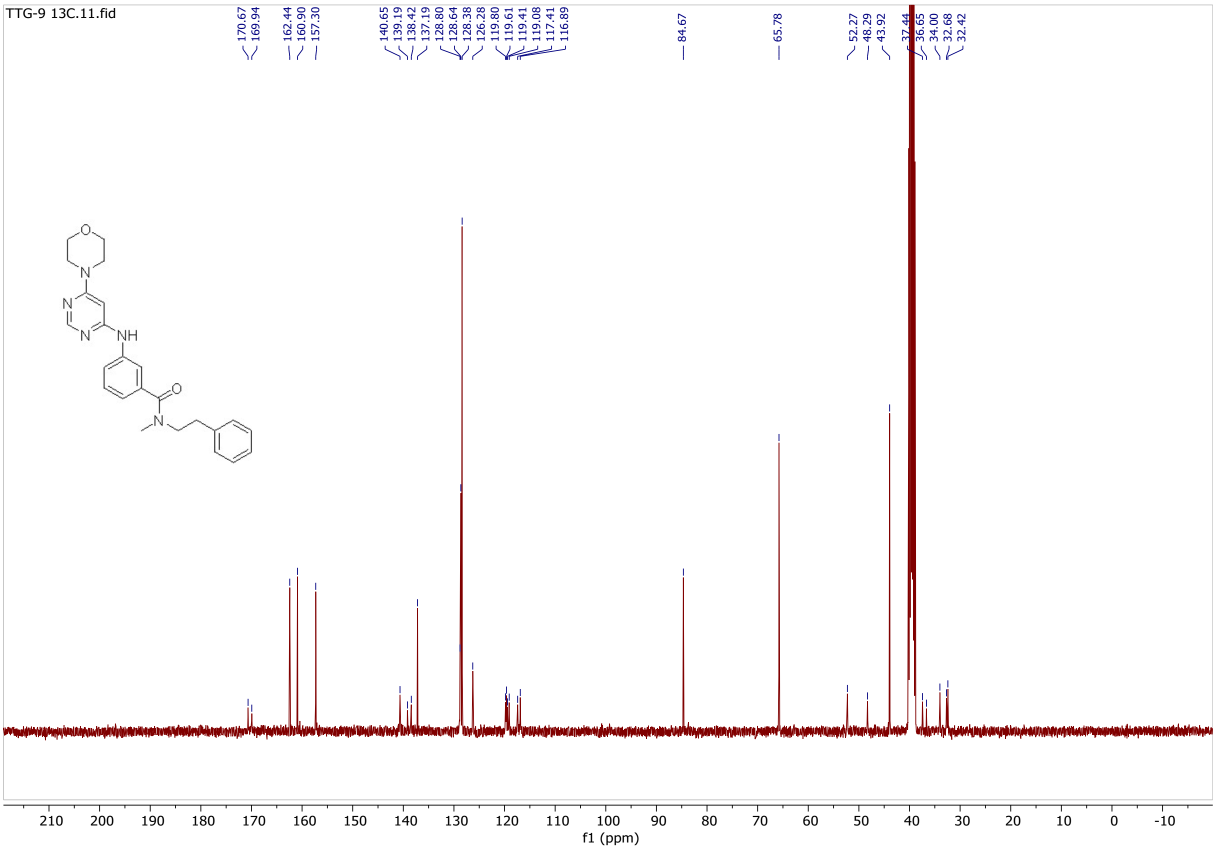

Compound **16** ^{1}H NMR (400 MHz, DMSO-d_6):

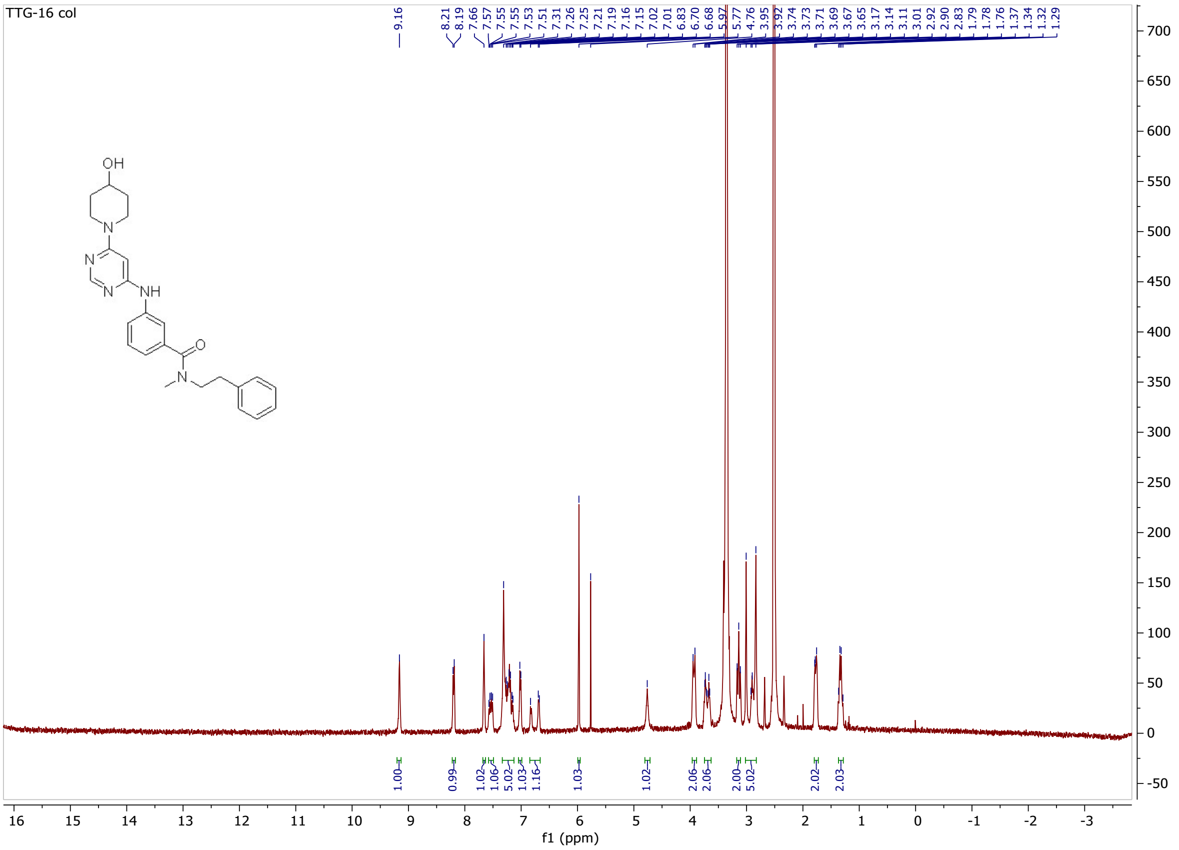

Compound **16** ^{13}C NMR (101 MHz, DMSO-d_6):

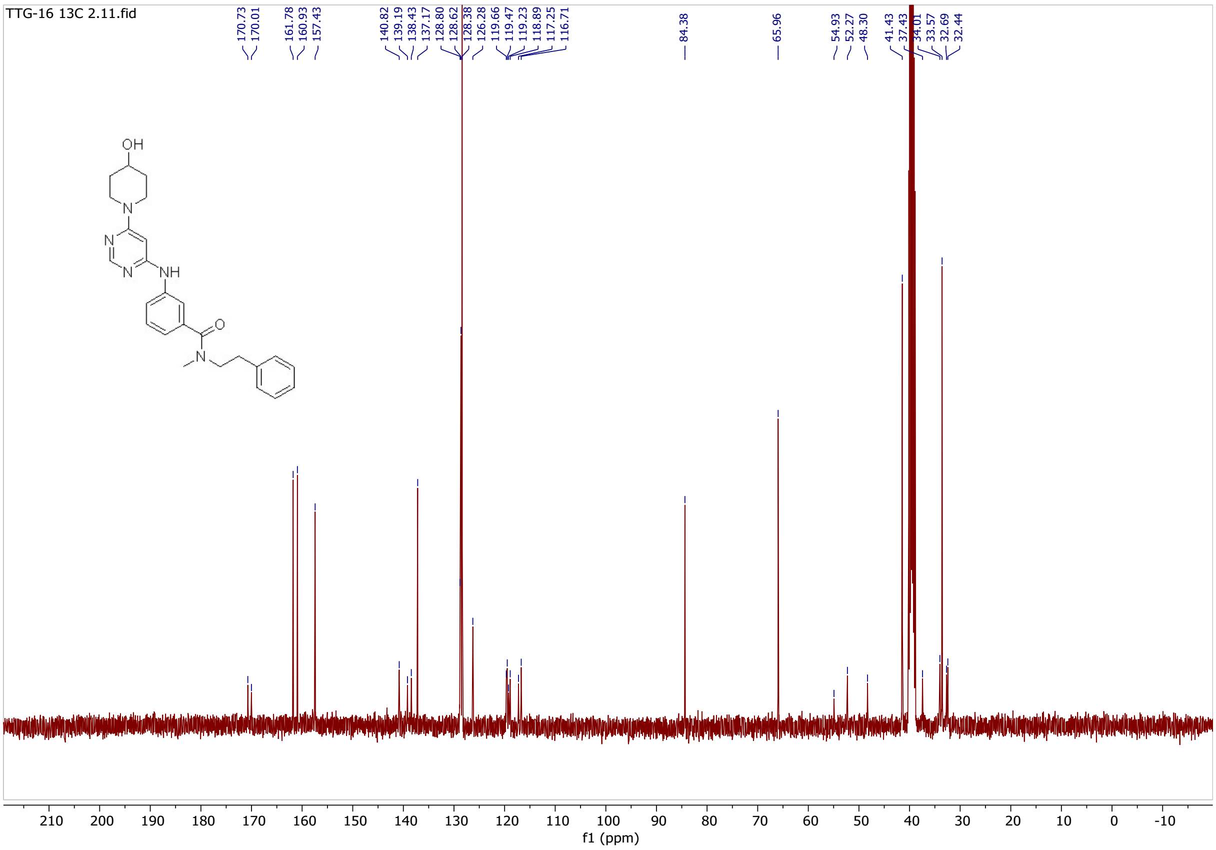

Compound **18** ^{1}H NMR (400 MHz, DMSO-d_6):

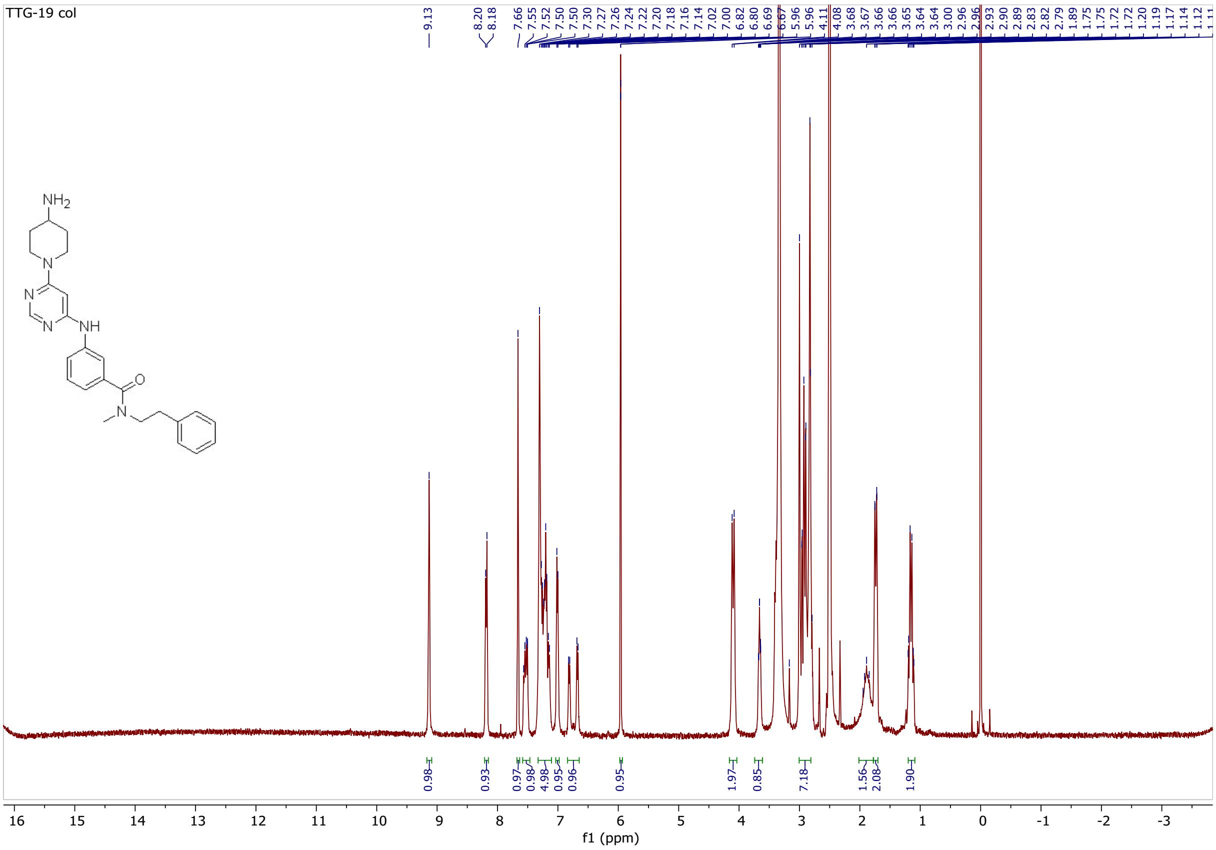

Compound **18** ^{13}C NMR (101 MHz, DMSO-d_6):

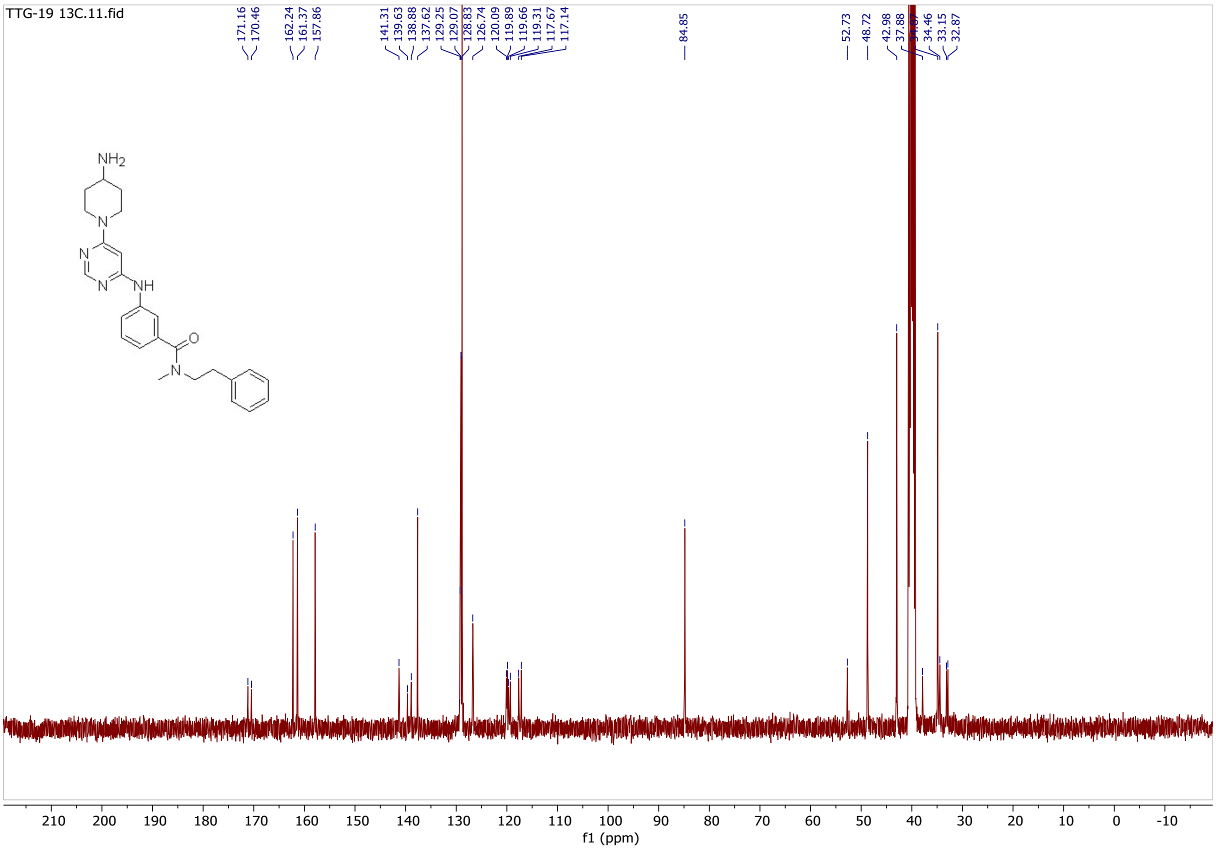

Compound **19** ^{1}H NMR (400 MHz, DMSO-d_6):

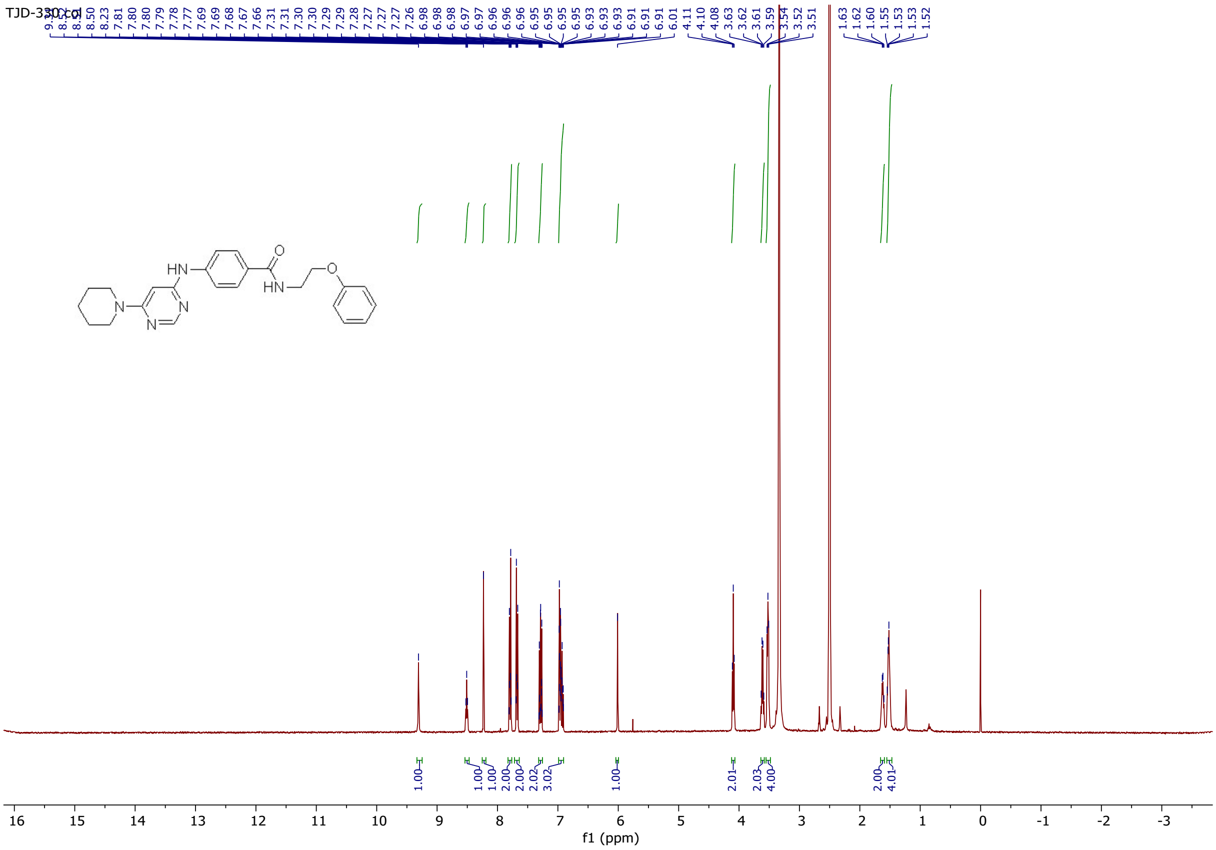

Compound **19** ^{13}C NMR (101 MHz, DMSO-d_6):

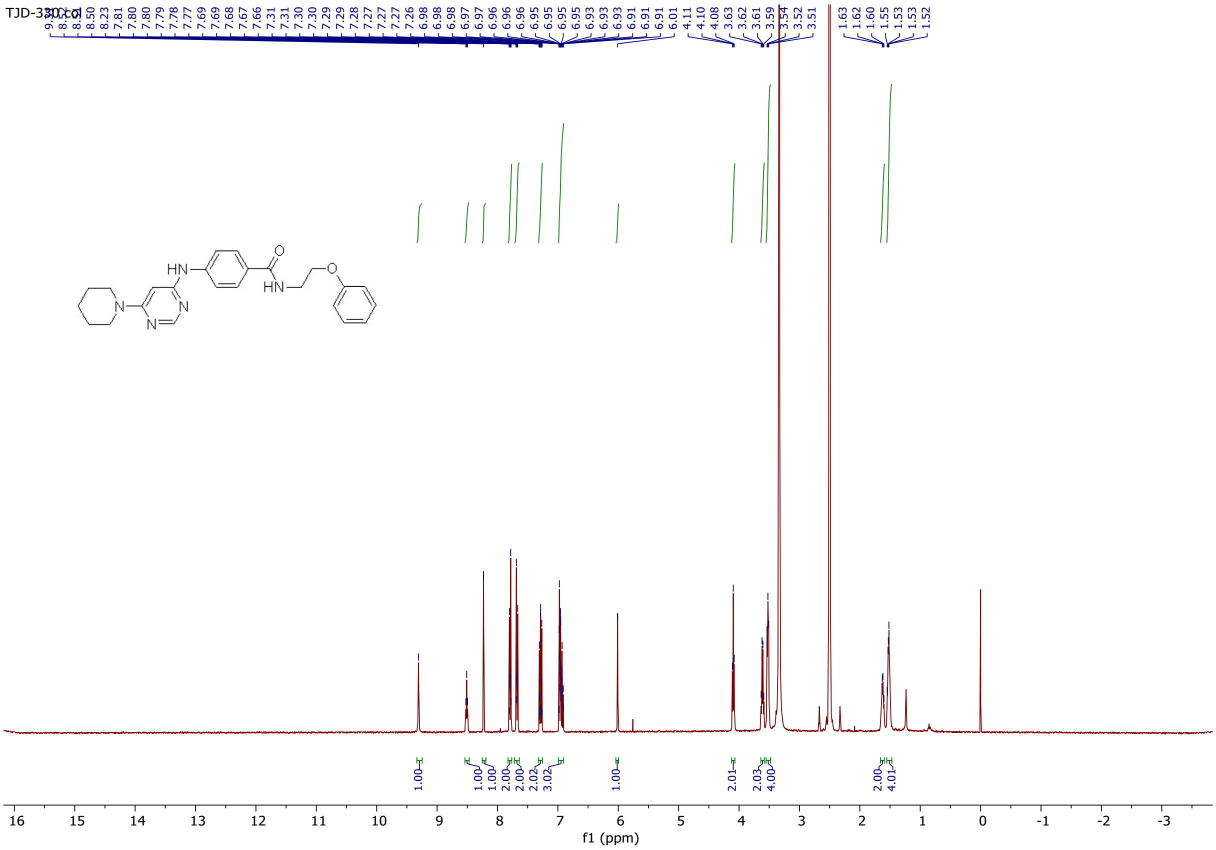

Compound **20** ^{1}H NMR (400 MHz, DMSO-d_6):

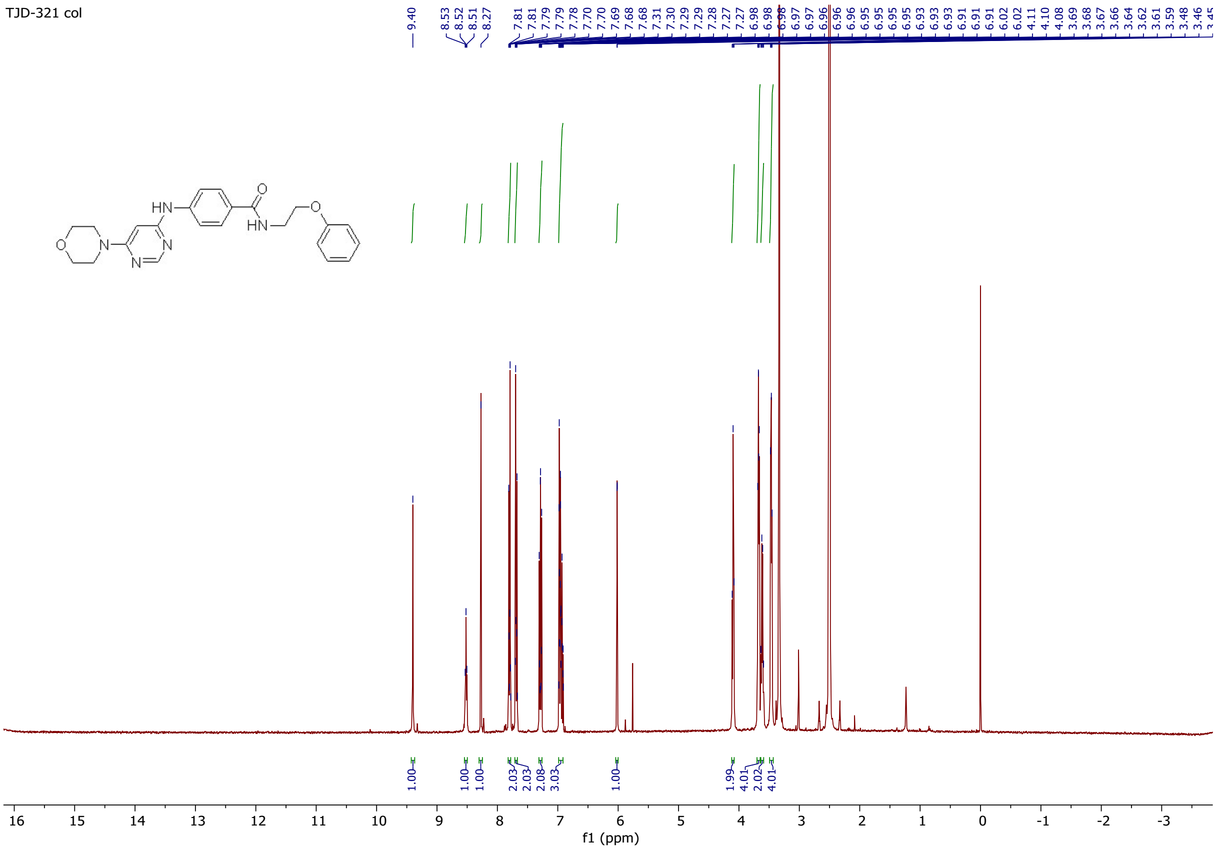

Compound **20** ^{13}C NMR (101 MHz, DMSO-d_6):

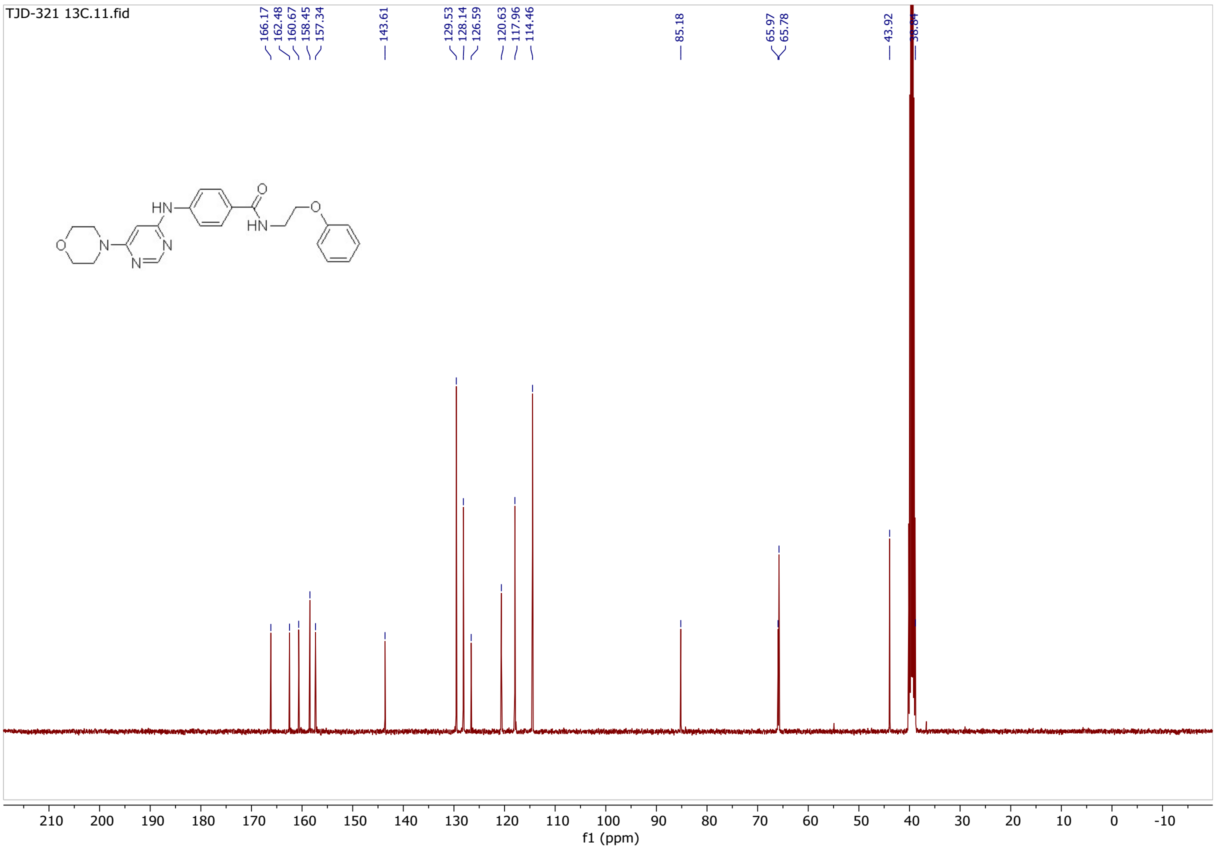

Compound **21** ^{1}H NMR (400 MHz, DMSO-d_6):

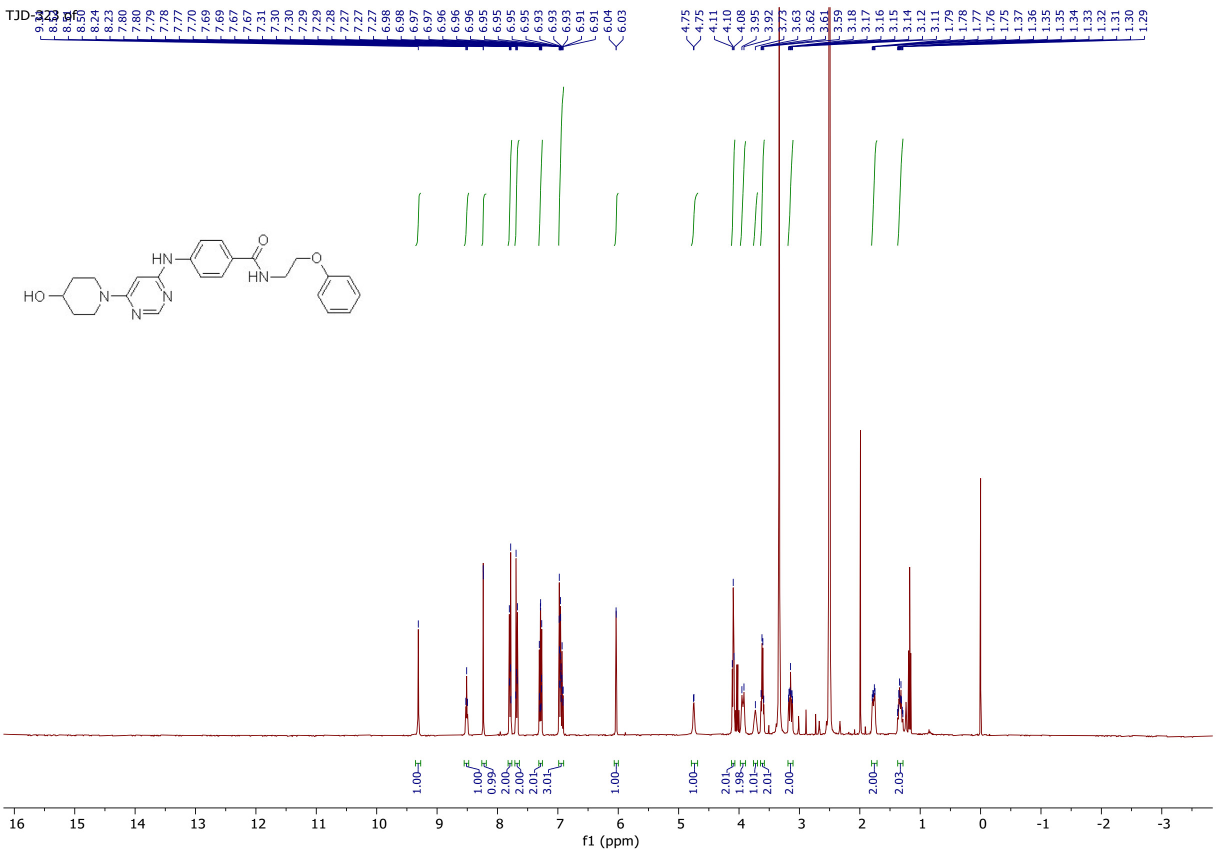

Compound **21** ^{13}C NMR (101 MHz, DMSO-d_6):

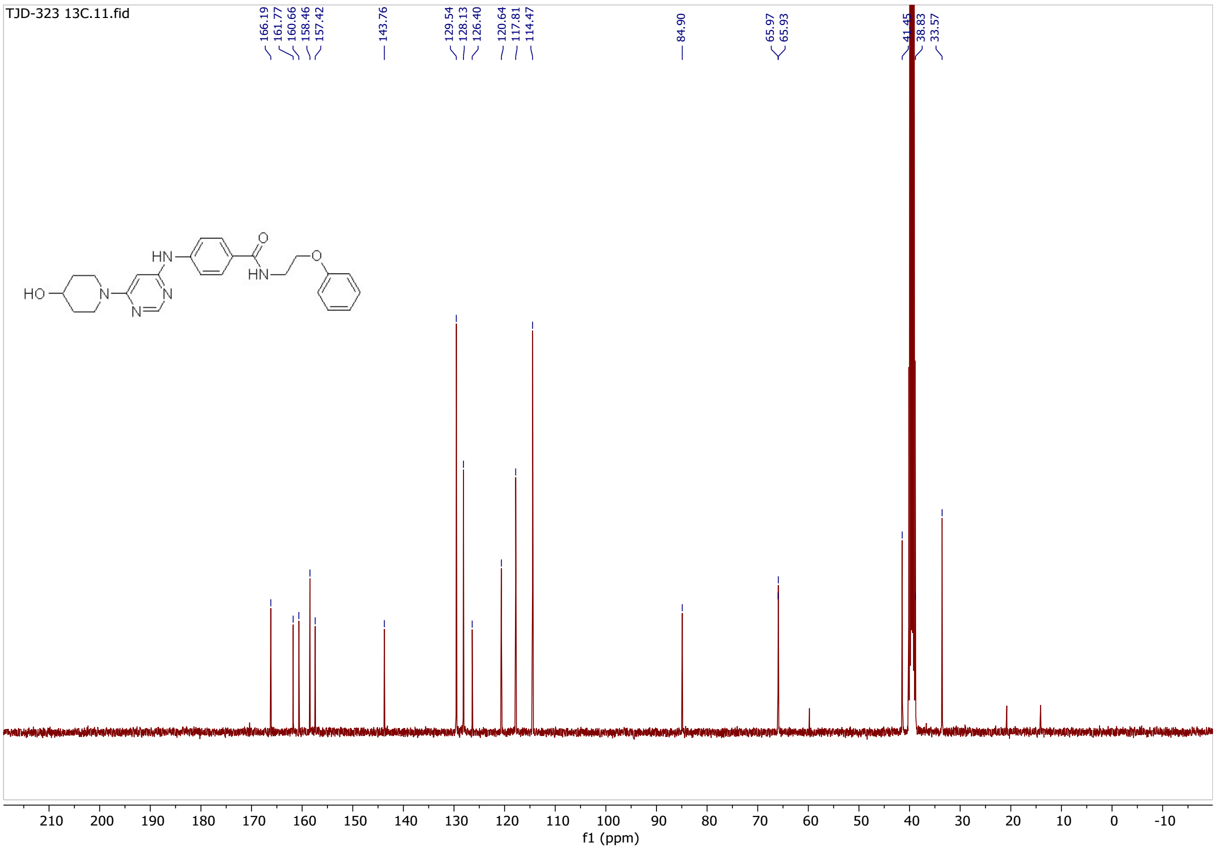

Compound **23** ^{1}H NMR (400 MHz, DMSO-d_6):

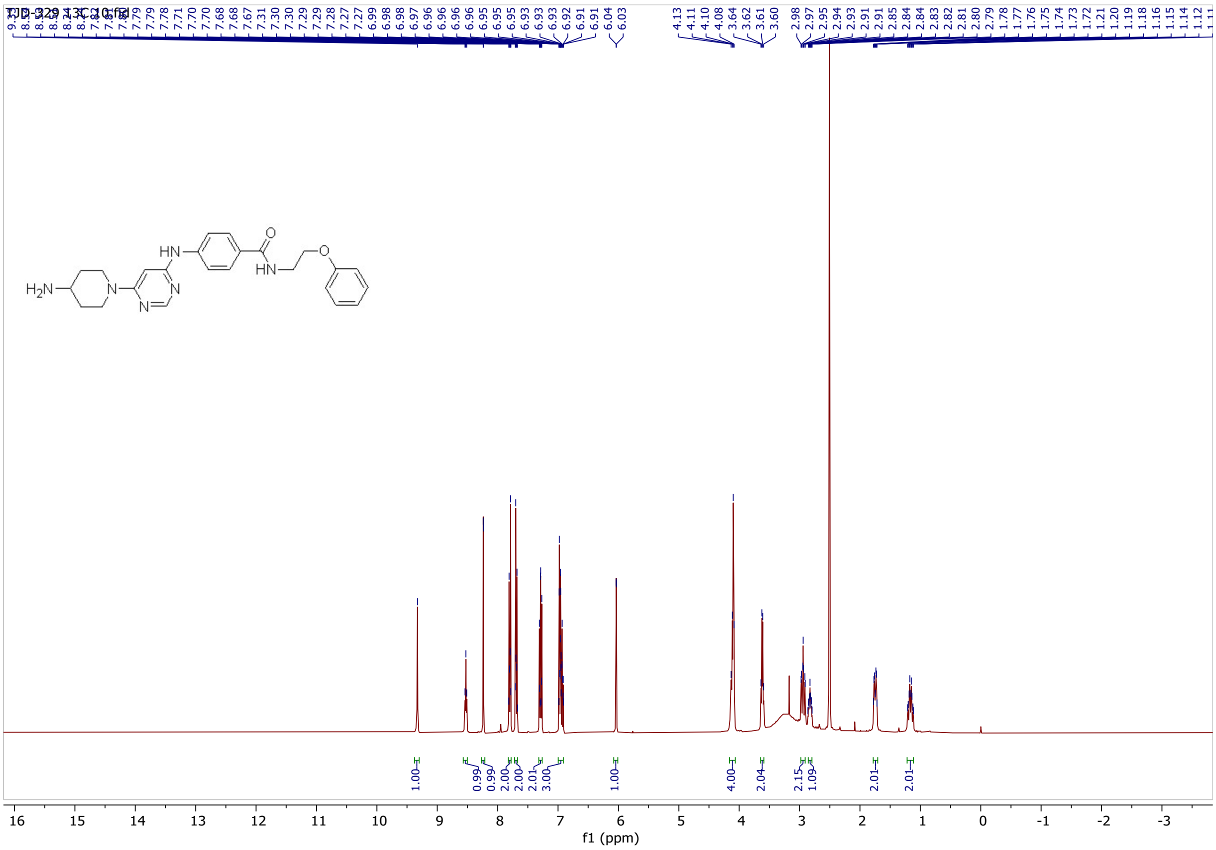

Compound **23** ^{13}C NMR (101 MHz, DMSO-d_6):

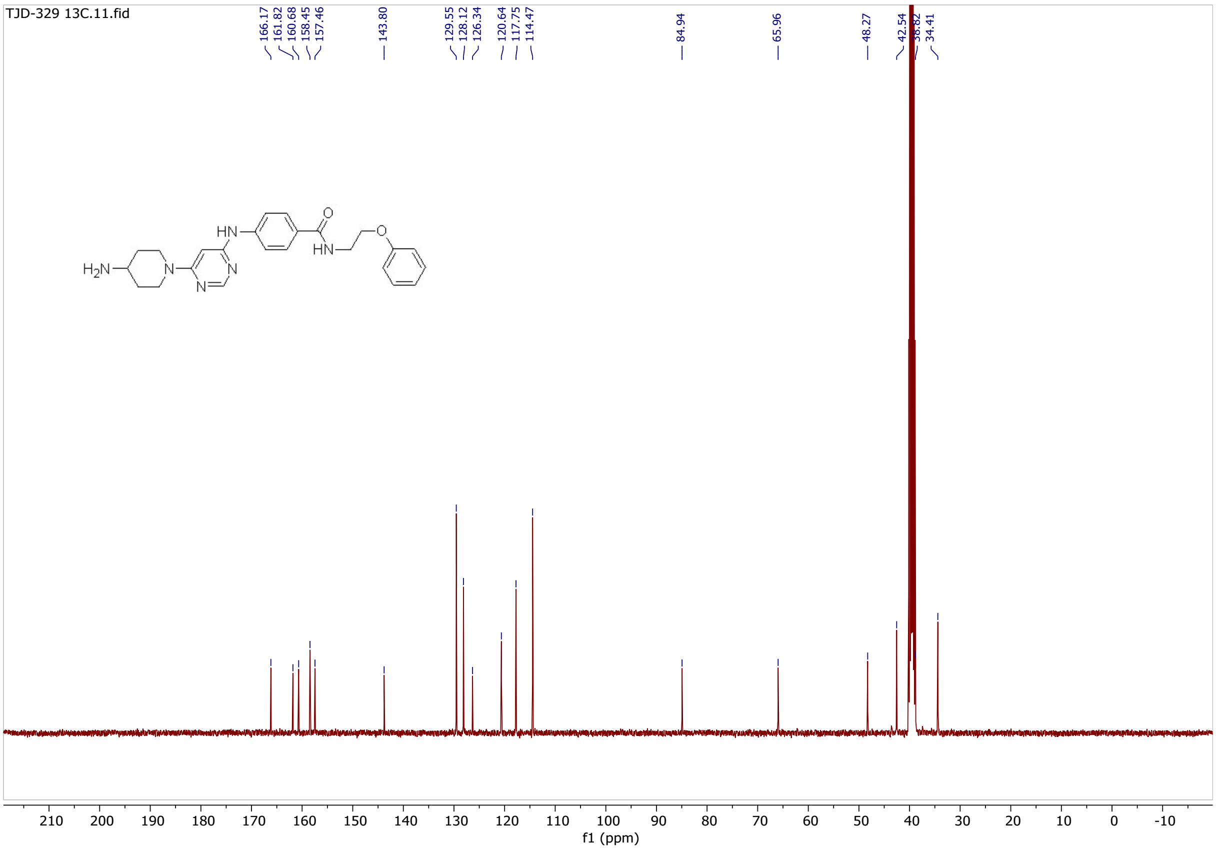

Compound **24** ^{1}H NMR (400 MHz, DMSO-d_6):

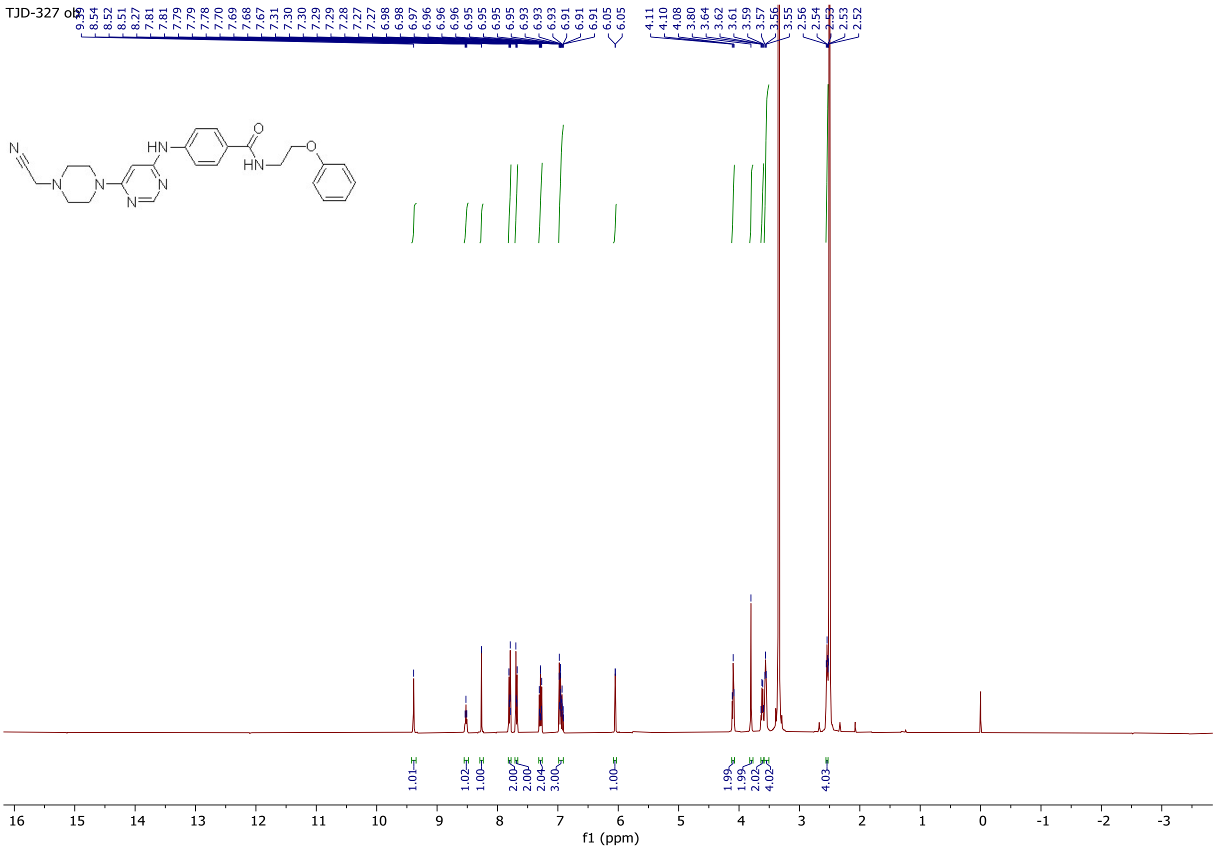

Compound **24** ^{13}C NMR (101 MHz, DMSO-d_6):

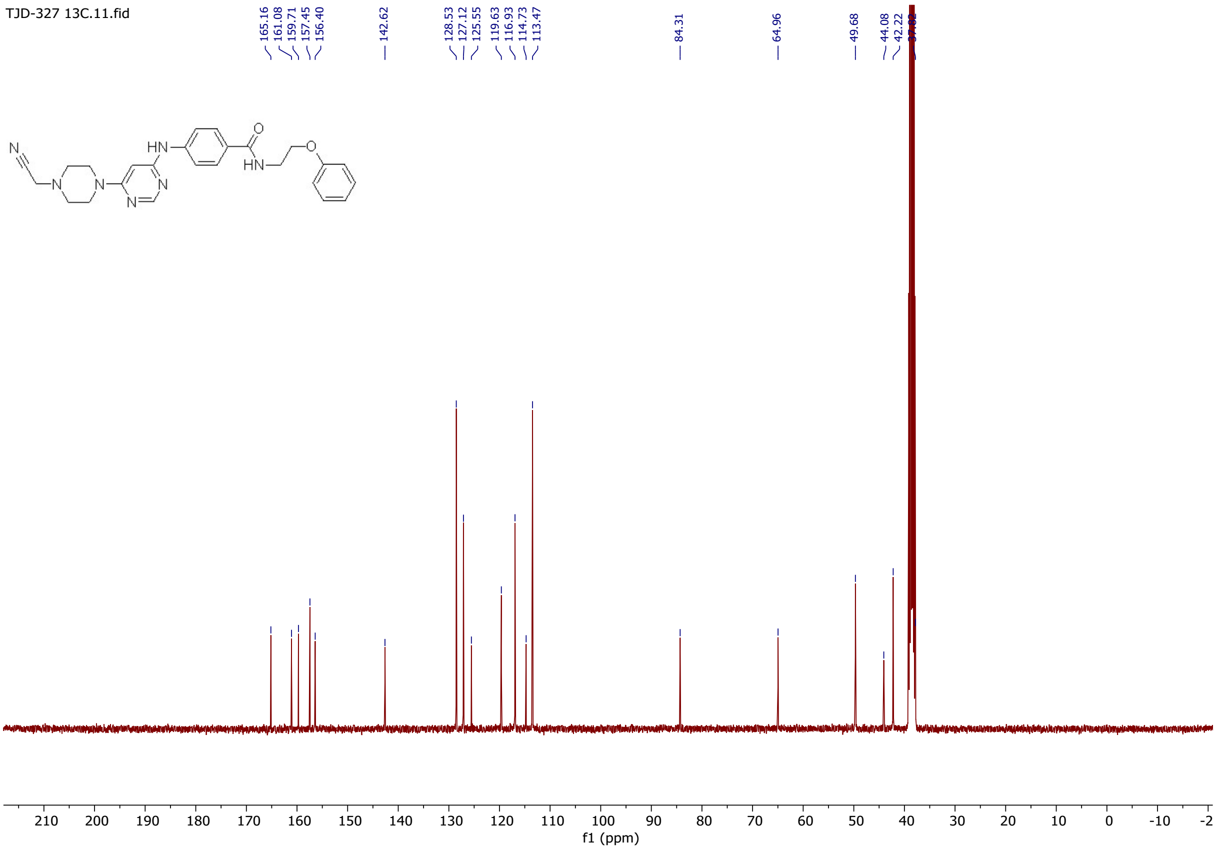

Literature

[1] L. Wang, J. Jiang, L. Zhang, Q. Zhang, J. Zhou, L. Li, X. Xu, Q. You, *J. Med. Chem.* **2020**, *63*, 1281–1297.
